# Supplementary figures and images for: Computational discovery of hidden breaks in 28S ribosomal RNAs across eukaryotes and consequences for RNA Integrity Numbers (part 1 of 3)
Source: Sci Rep. 2019 Dec 20;9:19477. doi: 10.1038/s41598-019-55573-1 (PMC6925239; doi:10.1038/s41598-019-55573-1)

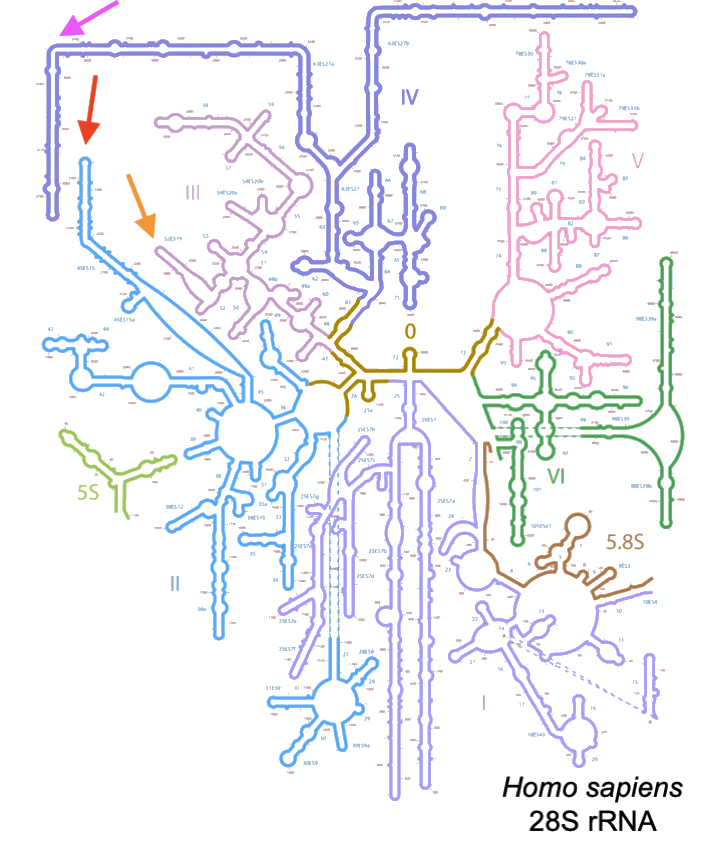

Supplement: Supplementary file 1 — Supplementary information [file 41598_2019_55573_MOESM1_ESM.png]

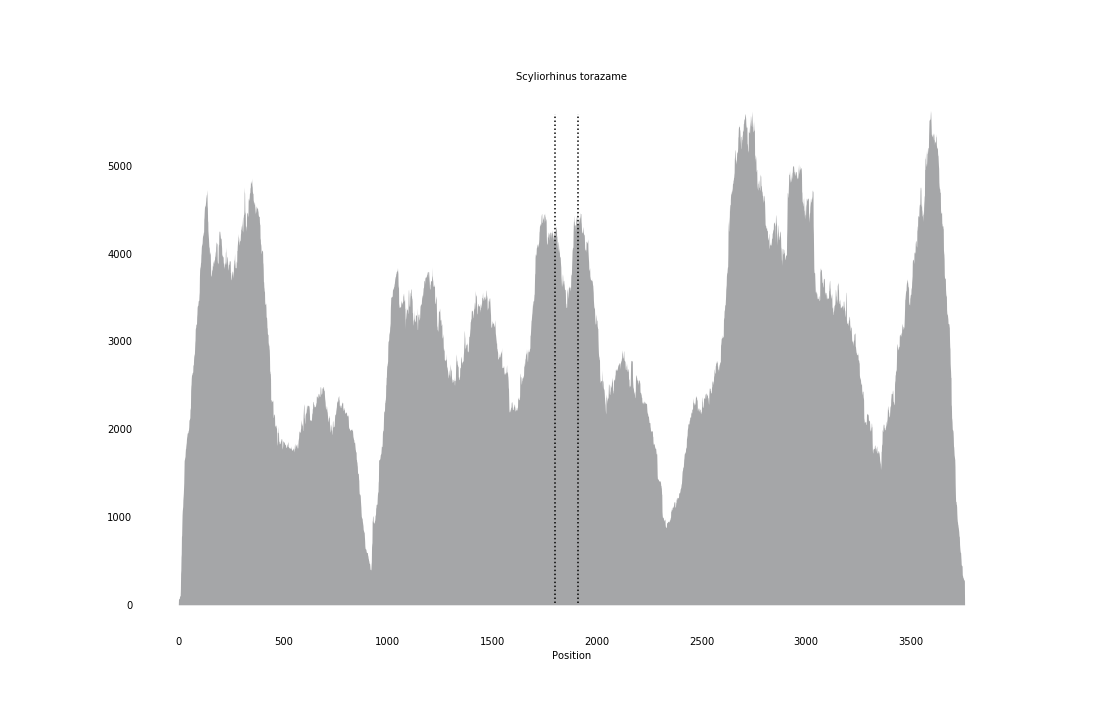

Supplement: Supplementary file 2 — Supplementary information [file 41598_2019_55573_MOESM2_ESM.zip › SupplementaryFile1/Metazoa/Deuterostomia/Scyliorhinus_torazame_coverage_correct.png]

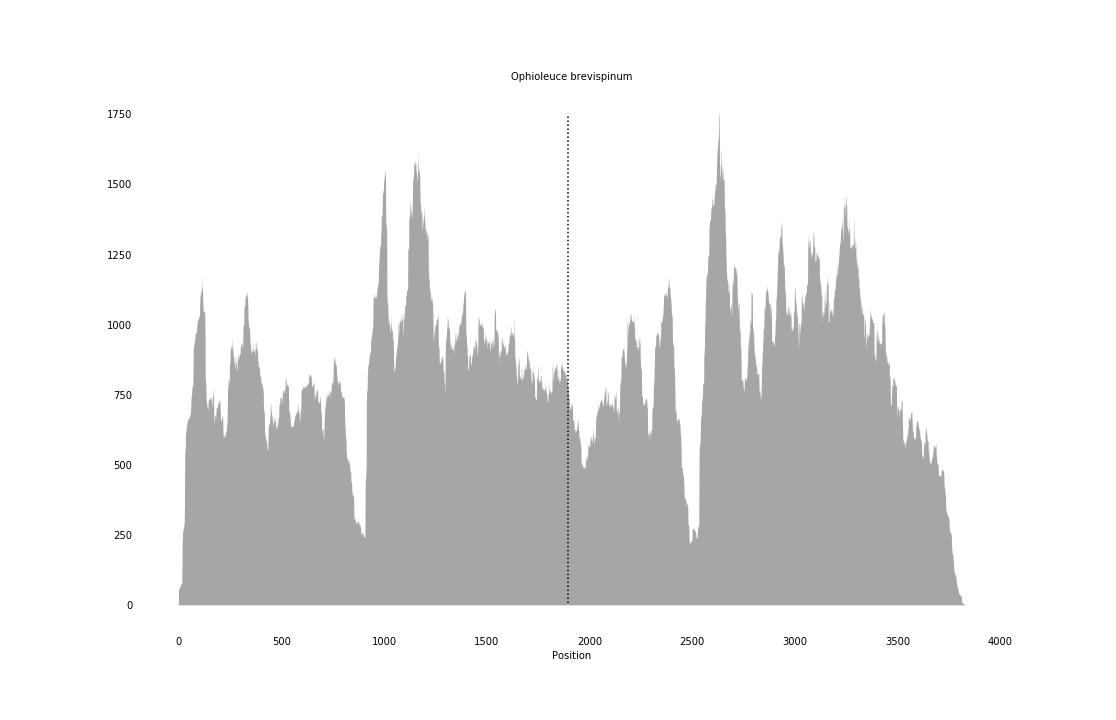

Supplement: Supplementary file 2 — Supplementary information [file 41598_2019_55573_MOESM2_ESM.zip › SupplementaryFile1/Metazoa/Deuterostomia/Ophioleuce_brevispinum_coverage.png]

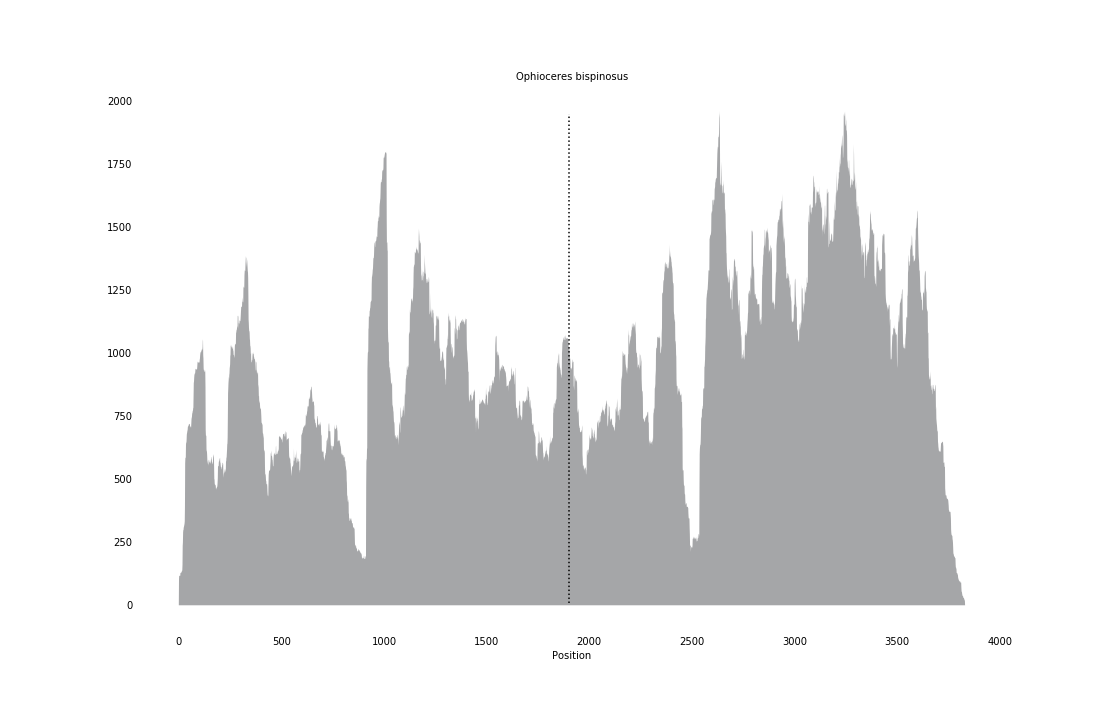

Supplement: Supplementary file 2 — Supplementary information [file 41598_2019_55573_MOESM2_ESM.zip › SupplementaryFile1/Metazoa/Deuterostomia/Ophioceres_bispinosus_coverage.png]

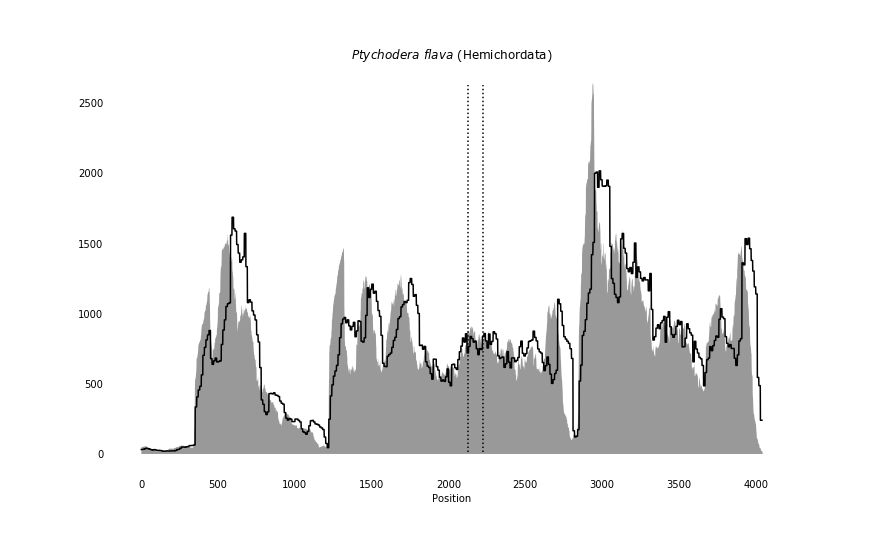

Supplement: Supplementary file 2 — Supplementary information [file 41598_2019_55573_MOESM2_ESM.zip › SupplementaryFile1/Metazoa/Deuterostomia/Ptychodera_flava_coverage.png]

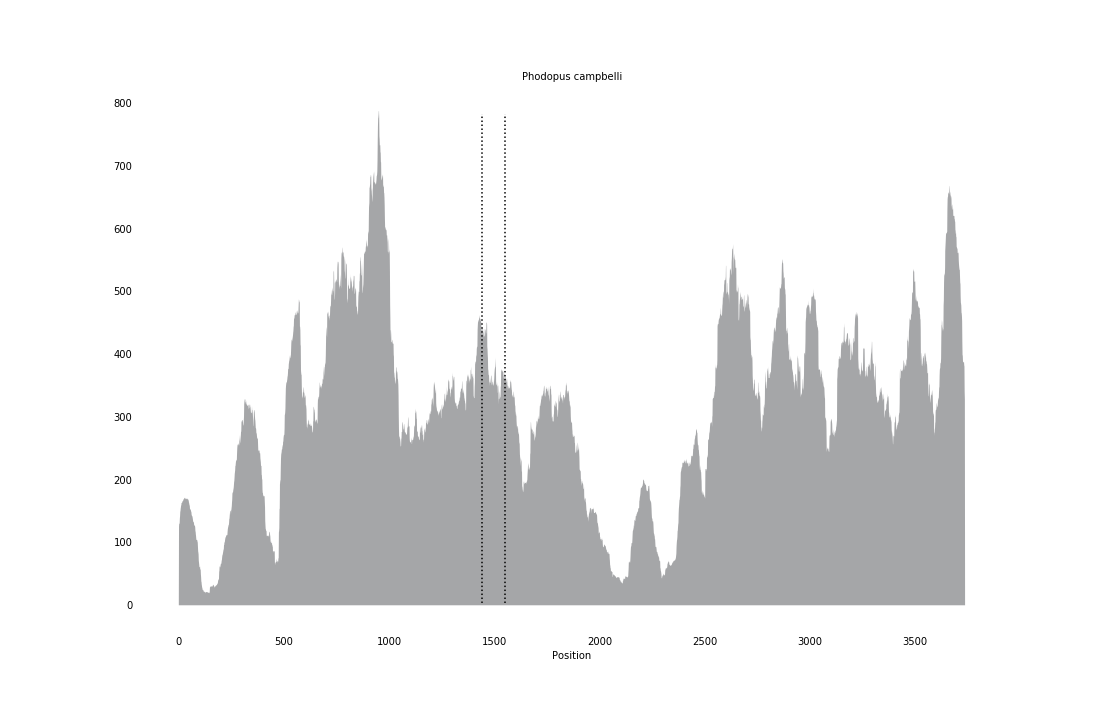

Supplement: Supplementary file 2 — Supplementary information [file 41598_2019_55573_MOESM2_ESM.zip › SupplementaryFile1/Metazoa/Deuterostomia/Phodopus_campbelli_coverage_correct.png]

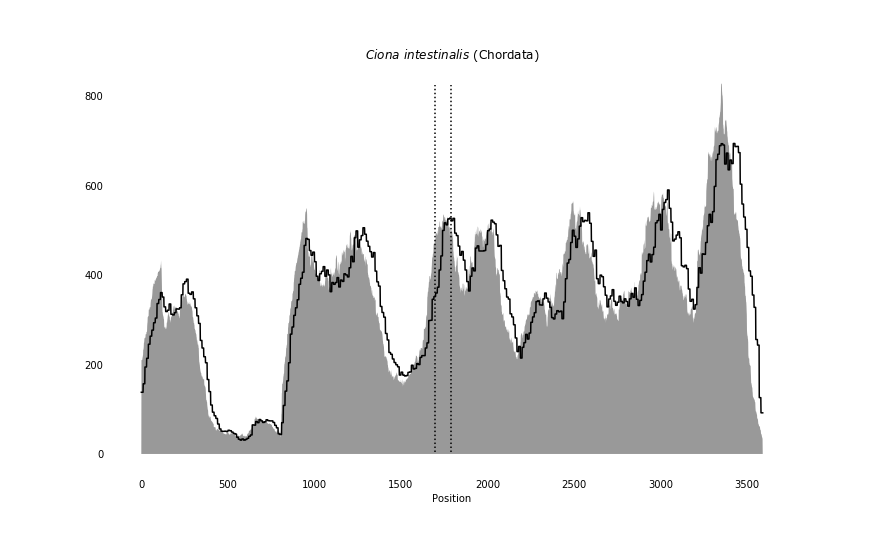

Supplement: Supplementary file 2 — Supplementary information [file 41598_2019_55573_MOESM2_ESM.zip › SupplementaryFile1/Metazoa/Deuterostomia/Ciona_intestinalis_coverage.png]

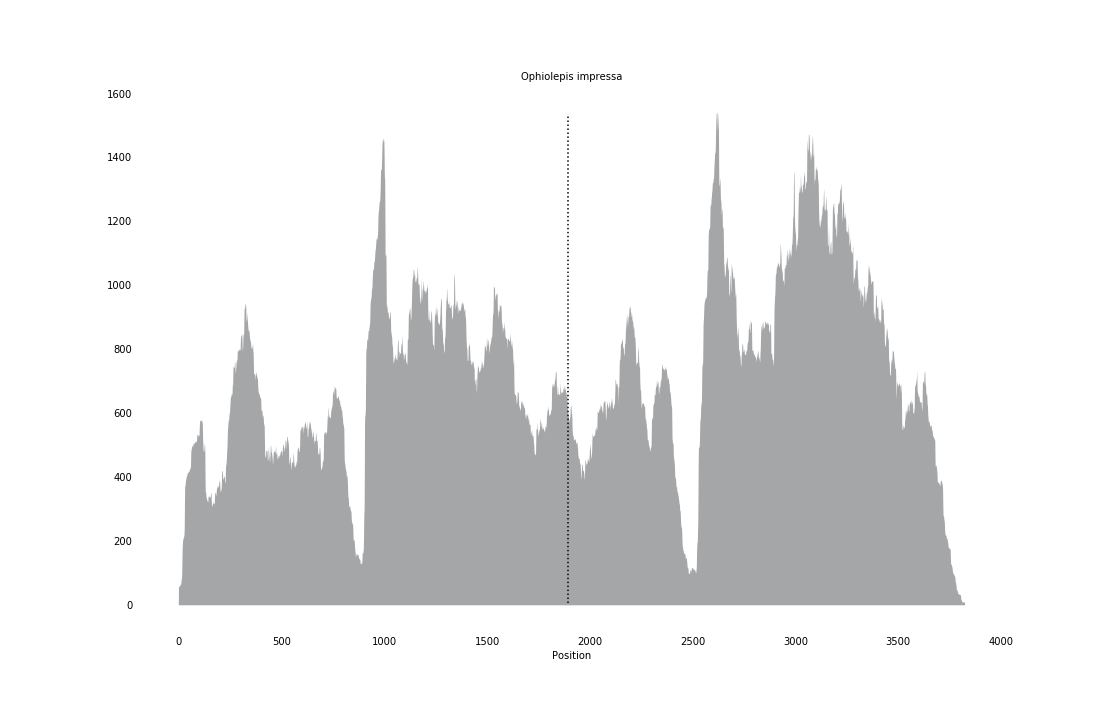

Supplement: Supplementary file 2 — Supplementary information [file 41598_2019_55573_MOESM2_ESM.zip › SupplementaryFile1/Metazoa/Deuterostomia/Ophiolepis_impressa_coverage.png]

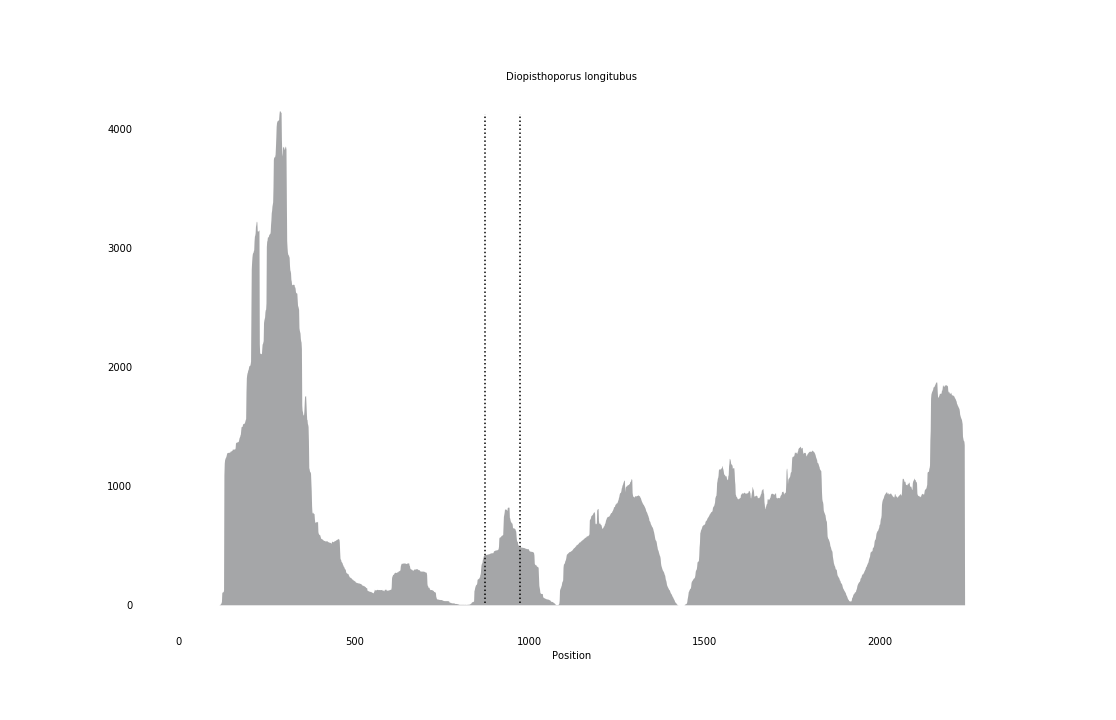

Supplement: Supplementary file 2 — Supplementary information [file 41598_2019_55573_MOESM2_ESM.zip › SupplementaryFile1/Metazoa/Deuterostomia/Diopisthoporus_longitubus_coverage_correct.png]

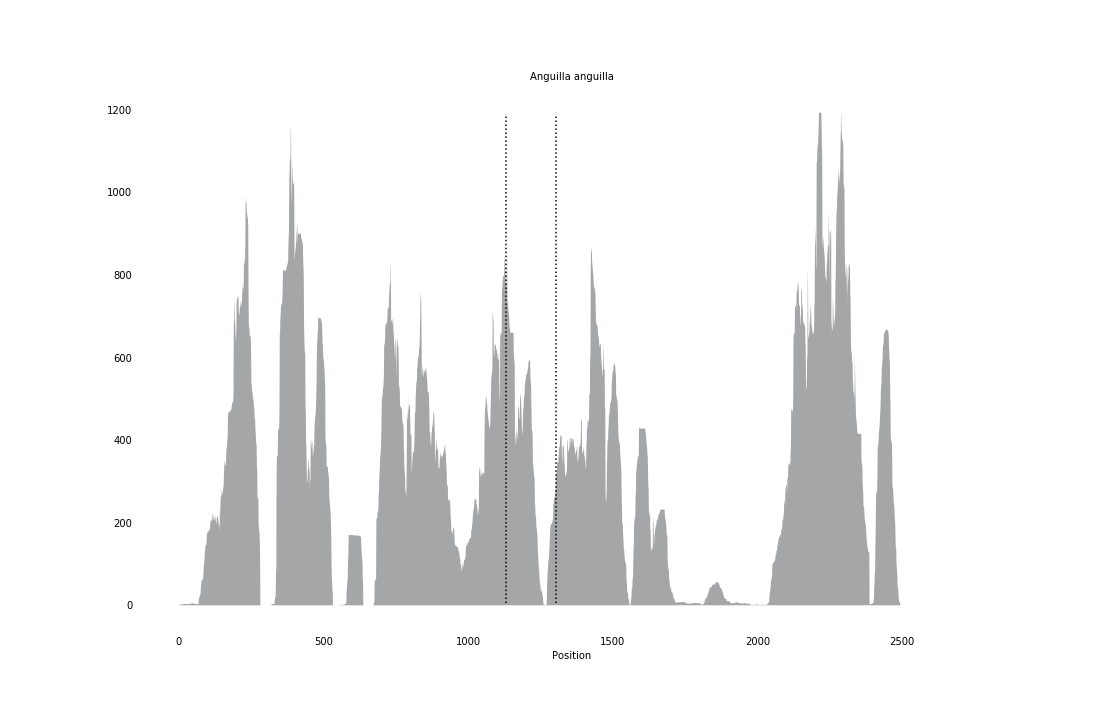

Supplement: Supplementary file 2 — Supplementary information [file 41598_2019_55573_MOESM2_ESM.zip › SupplementaryFile1/Metazoa/Deuterostomia/Anguilla_anguilla_coverage_correct.png]

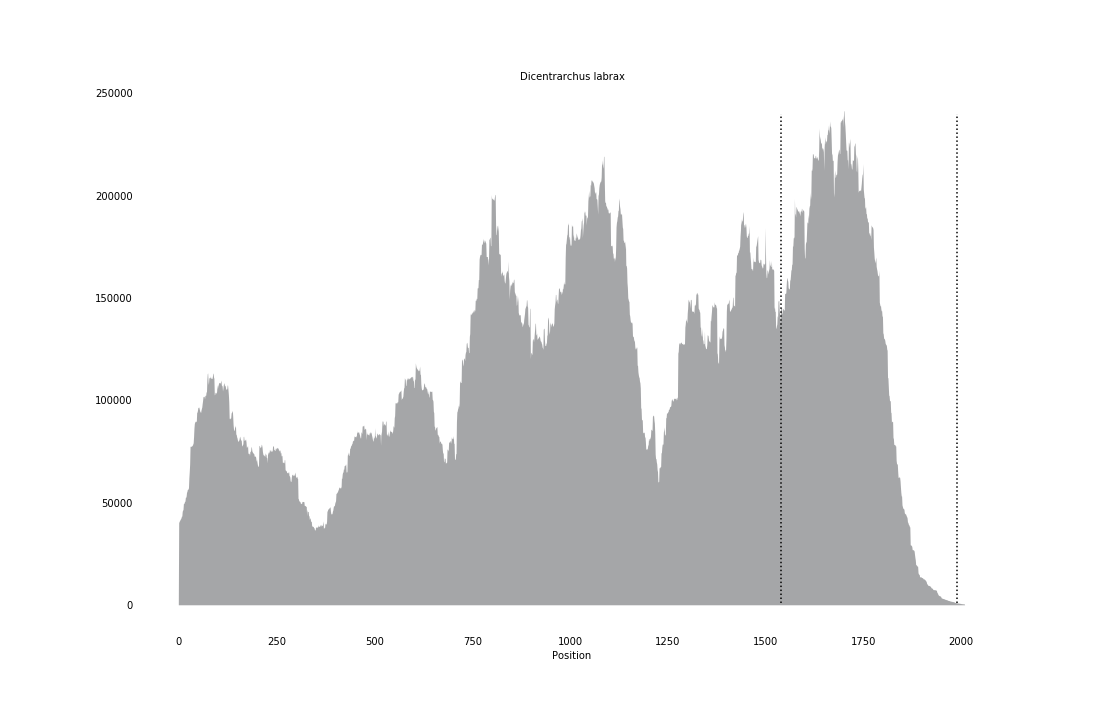

Supplement: Supplementary file 2 — Supplementary information [file 41598_2019_55573_MOESM2_ESM.zip › SupplementaryFile1/Metazoa/Deuterostomia/Dicentrarchus_labrax_coverage_correct.png]

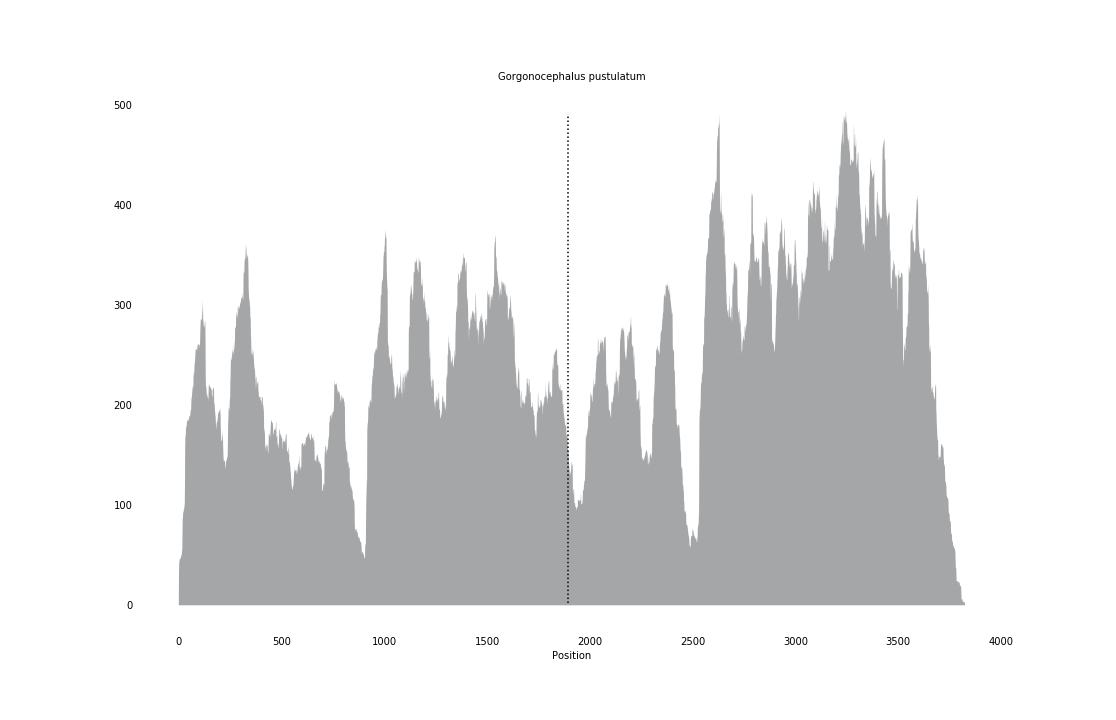

Supplement: Supplementary file 2 — Supplementary information [file 41598_2019_55573_MOESM2_ESM.zip › SupplementaryFile1/Metazoa/Deuterostomia/Gorgonocephalus_pustulatum_coverage.png]

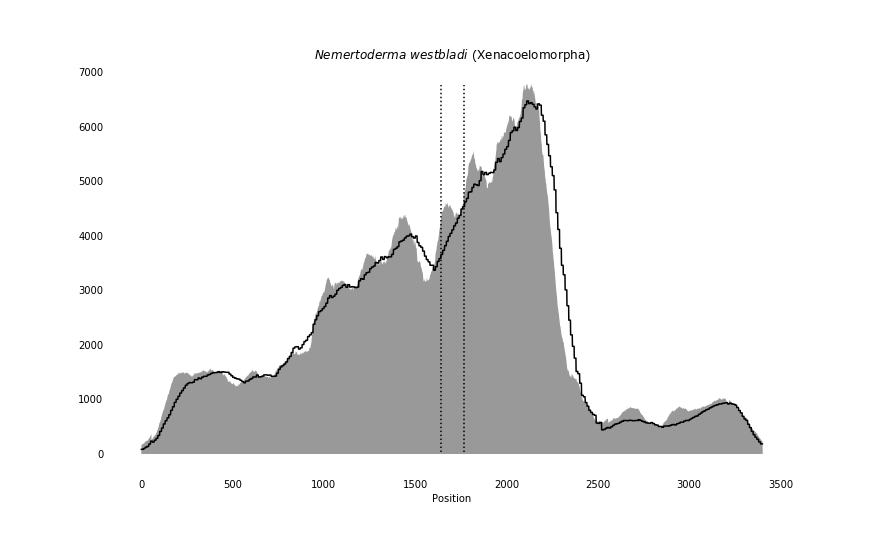

Supplement: Supplementary file 2 — Supplementary information [file 41598_2019_55573_MOESM2_ESM.zip › SupplementaryFile1/Metazoa/Deuterostomia/Nemertoderma_westbladi_coverage.png]

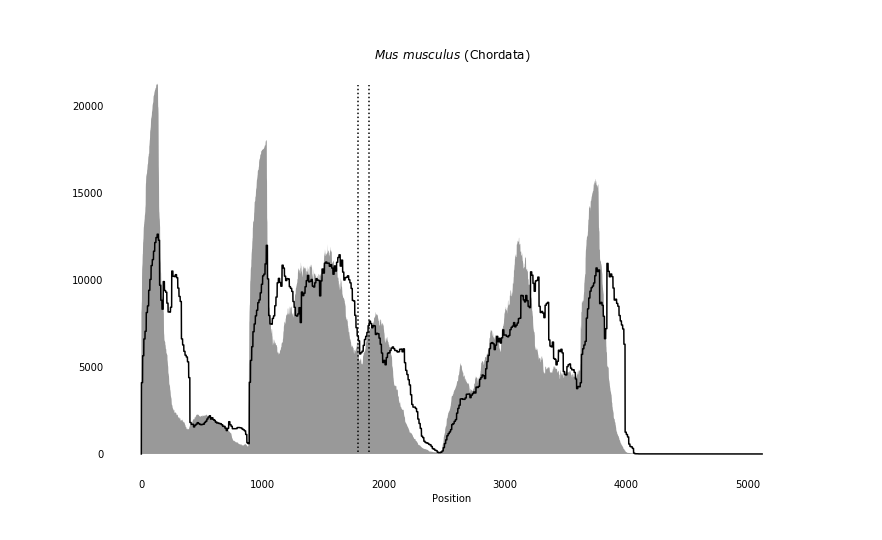

Supplement: Supplementary file 2 — Supplementary information [file 41598_2019_55573_MOESM2_ESM.zip › SupplementaryFile1/Metazoa/Deuterostomia/Cyprinus_carpio_coverage.png]

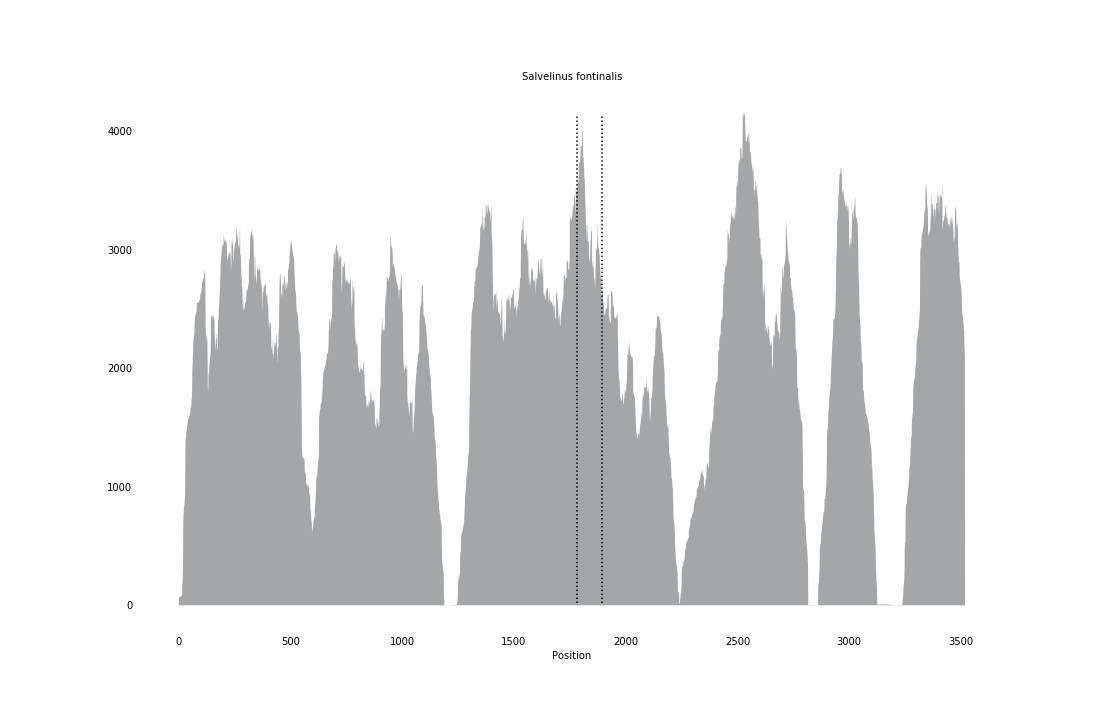

Supplement: Supplementary file 2 — Supplementary information [file 41598_2019_55573_MOESM2_ESM.zip › SupplementaryFile1/Metazoa/Deuterostomia/Salvelinus_fontinalis_coverage_correct.png]

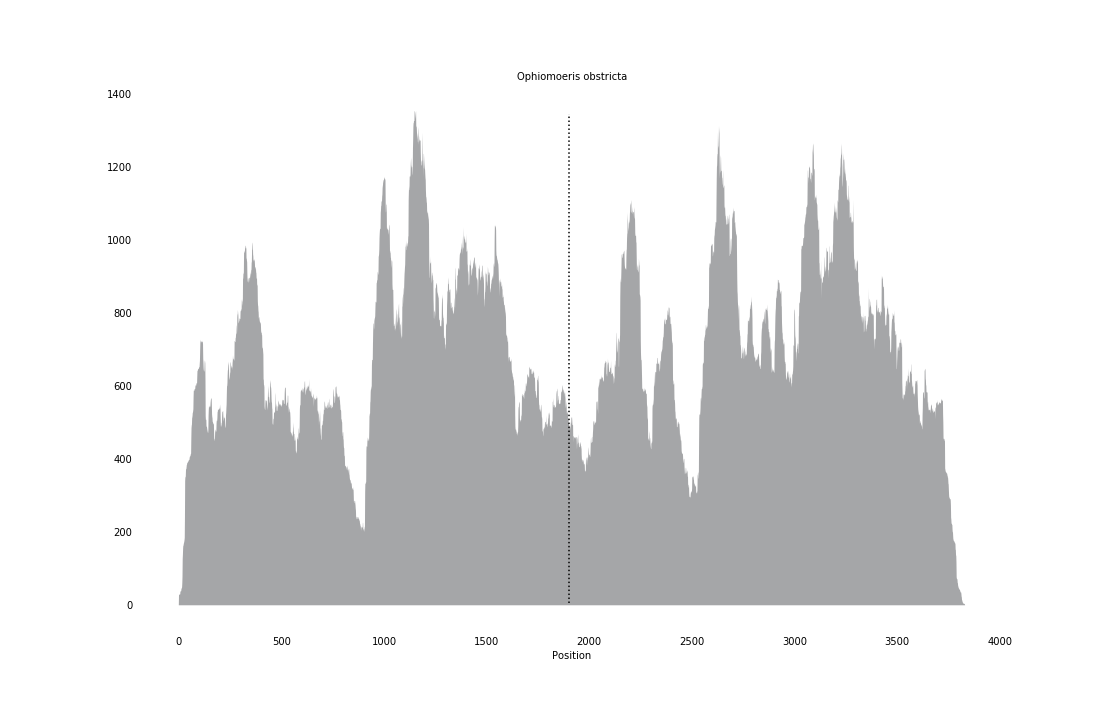

Supplement: Supplementary file 2 — Supplementary information [file 41598_2019_55573_MOESM2_ESM.zip › SupplementaryFile1/Metazoa/Deuterostomia/Ophiomoeris_obstricta_coverage.png]

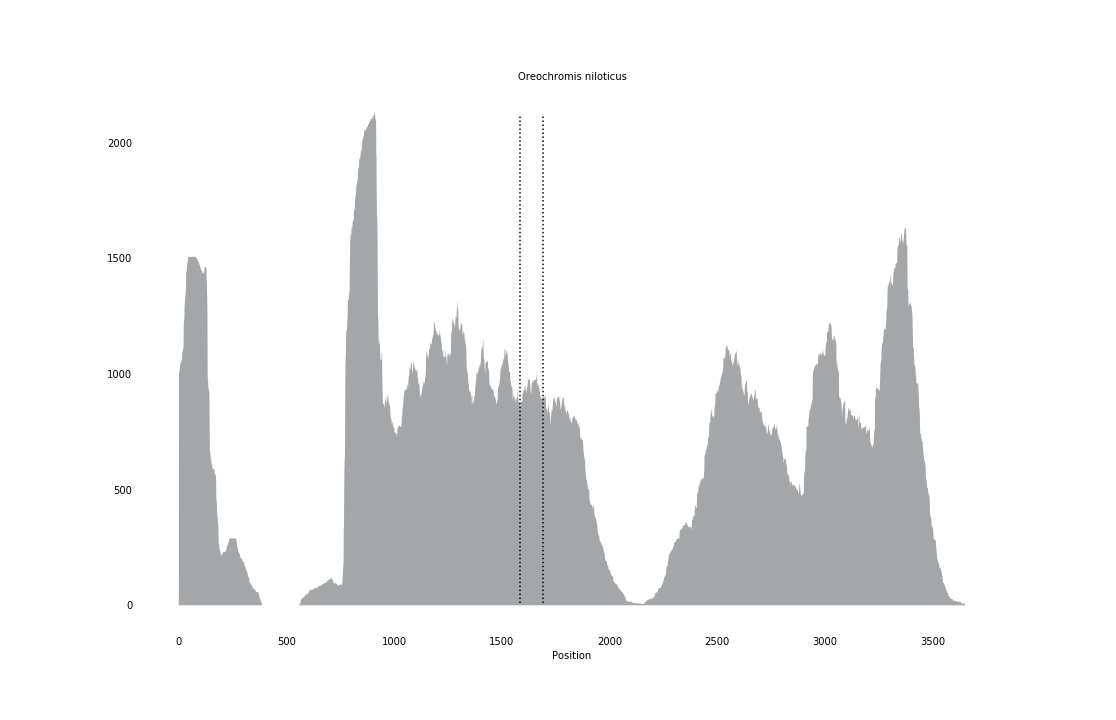

Supplement: Supplementary file 2 — Supplementary information [file 41598_2019_55573_MOESM2_ESM.zip › SupplementaryFile1/Metazoa/Deuterostomia/Oreochromis_niloticus_coverage_correct.png]

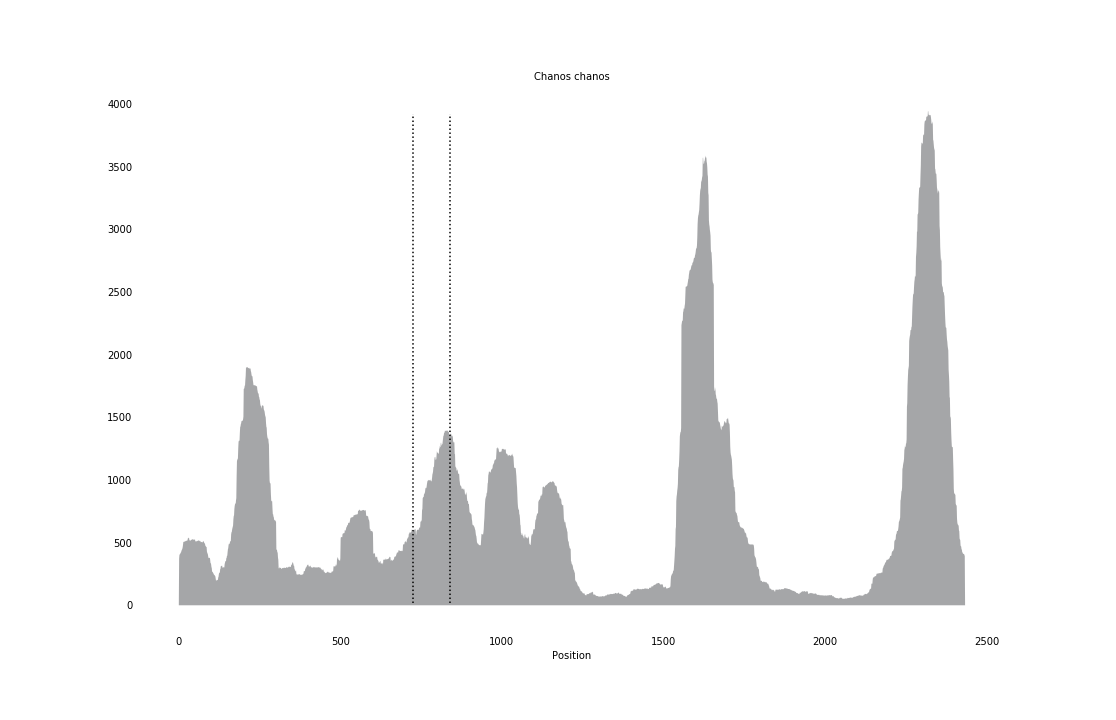

Supplement: Supplementary file 2 — Supplementary information [file 41598_2019_55573_MOESM2_ESM.zip › SupplementaryFile1/Metazoa/Deuterostomia/Chanos_chanos_coverage_correct.png]

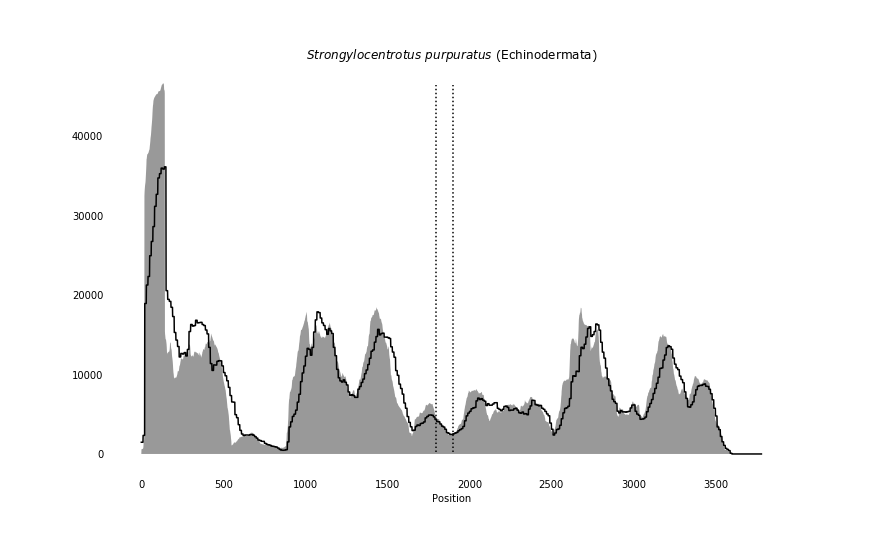

Supplement: Supplementary file 2 — Supplementary information [file 41598_2019_55573_MOESM2_ESM.zip › SupplementaryFile1/Metazoa/Deuterostomia/Strongylocentrotus_purpuratus_coverage.png]

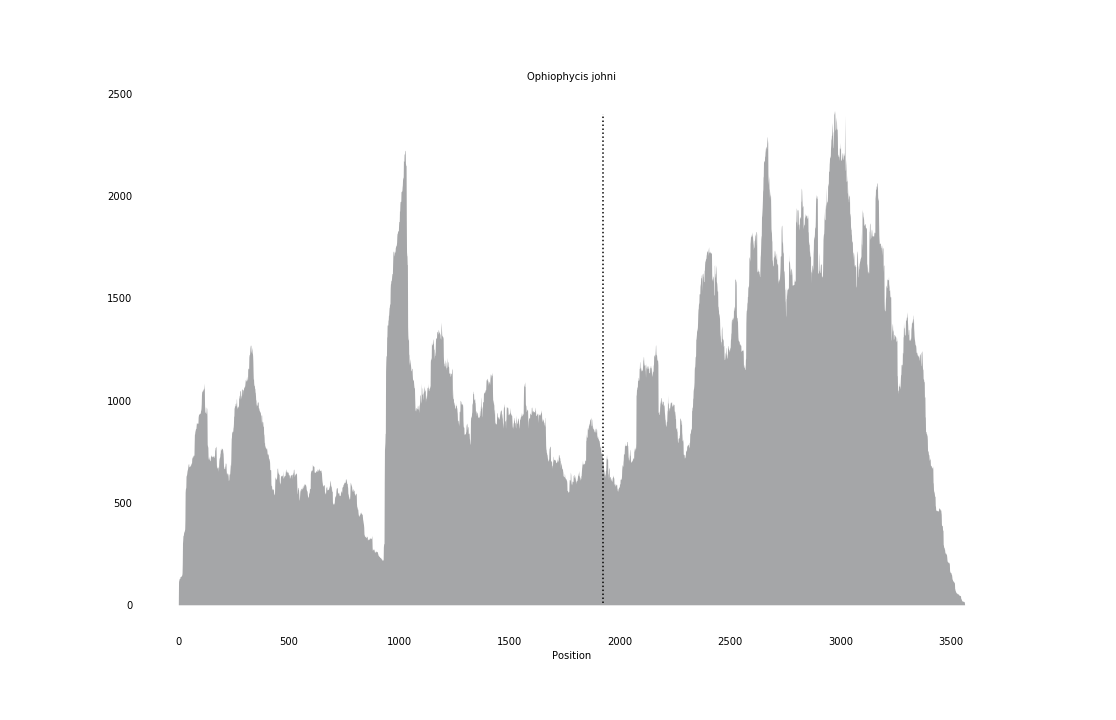

Supplement: Supplementary file 2 — Supplementary information [file 41598_2019_55573_MOESM2_ESM.zip › SupplementaryFile1/Metazoa/Deuterostomia/Ophiophycis_johni_coverage.png]

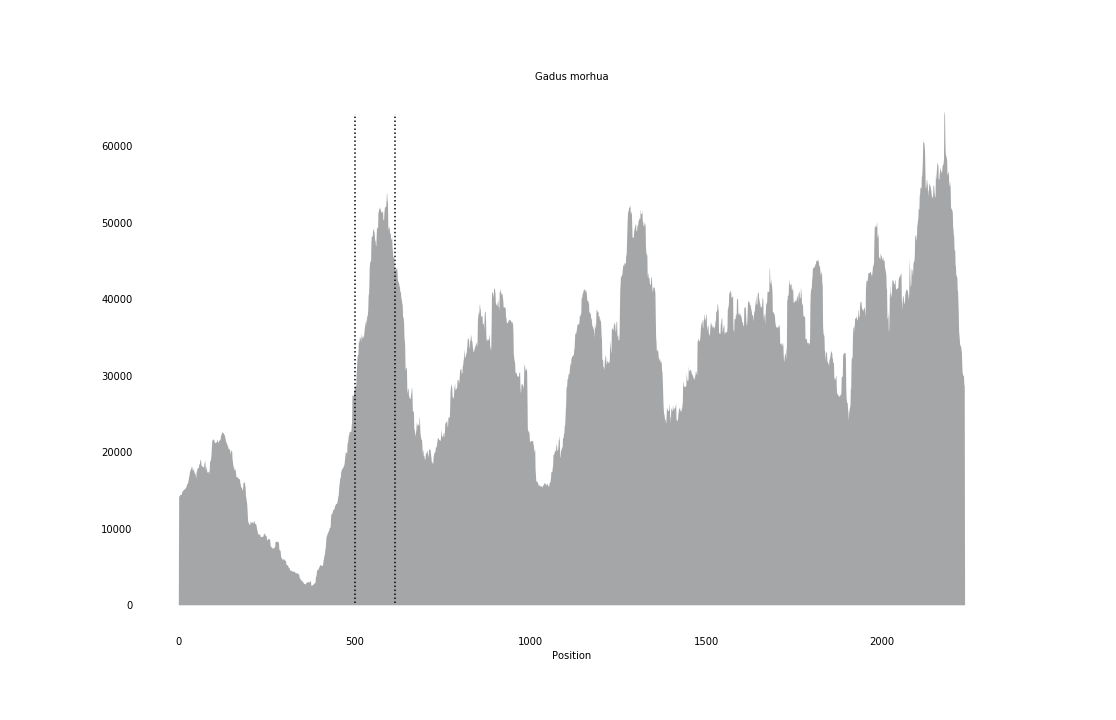

Supplement: Supplementary file 2 — Supplementary information [file 41598_2019_55573_MOESM2_ESM.zip › SupplementaryFile1/Metazoa/Deuterostomia/Gadus_morhua_coverage_correct.png]

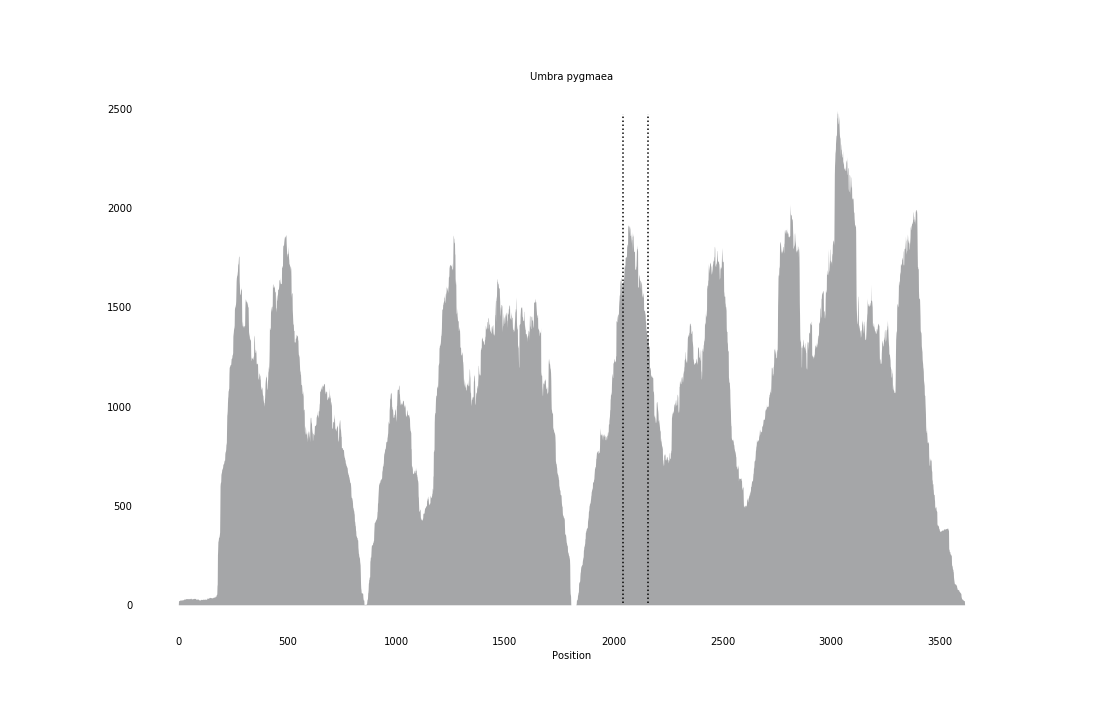

Supplement: Supplementary file 2 — Supplementary information [file 41598_2019_55573_MOESM2_ESM.zip › SupplementaryFile1/Metazoa/Deuterostomia/Umbra_pygmaea_coverage_correct.png]

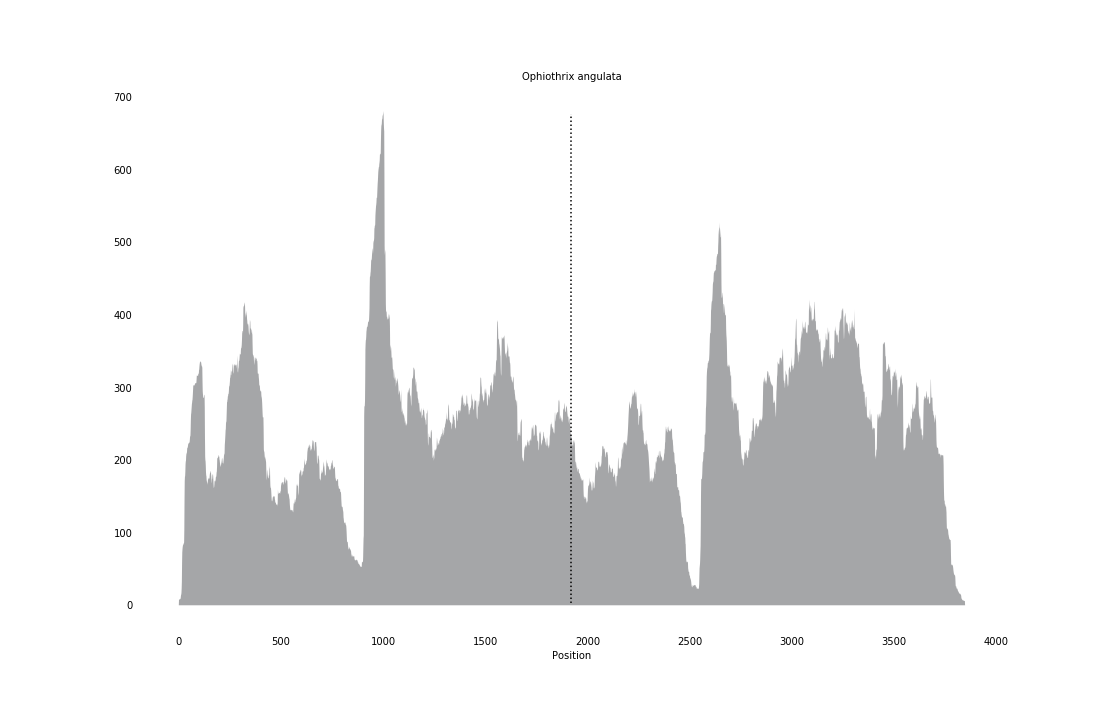

Supplement: Supplementary file 2 — Supplementary information [file 41598_2019_55573_MOESM2_ESM.zip › SupplementaryFile1/Metazoa/Deuterostomia/Ophiothrix_angulata_coverage.png]

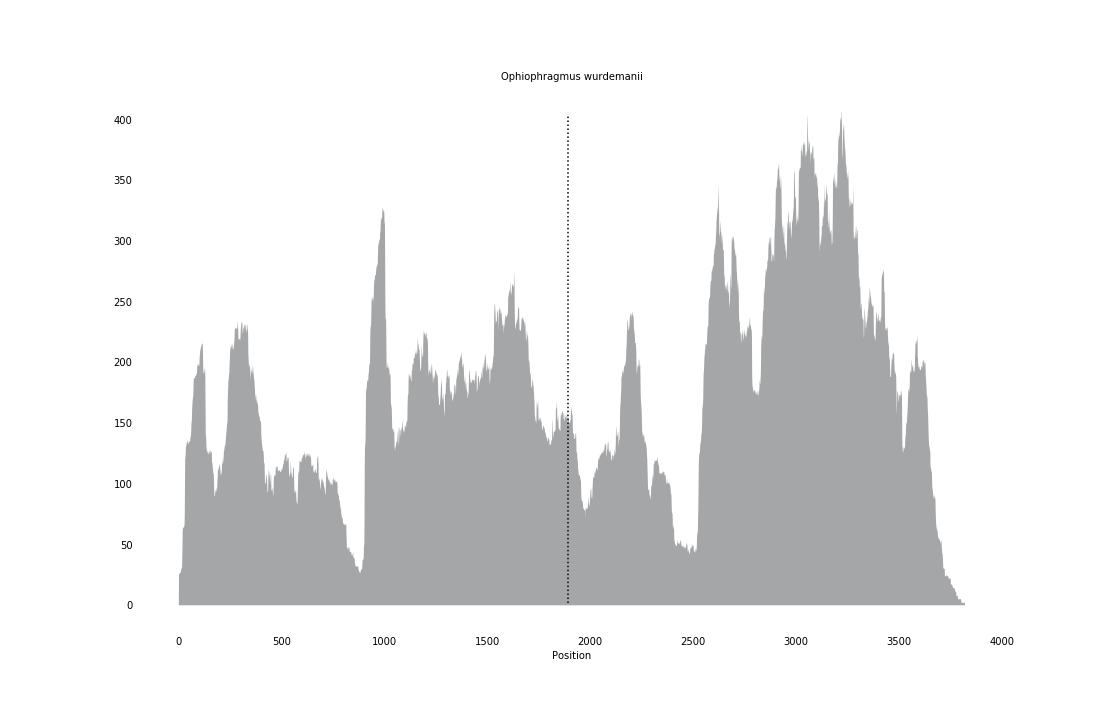

Supplement: Supplementary file 2 — Supplementary information [file 41598_2019_55573_MOESM2_ESM.zip › SupplementaryFile1/Metazoa/Deuterostomia/Ophiophragmus_wurdemanii_coverage.png]

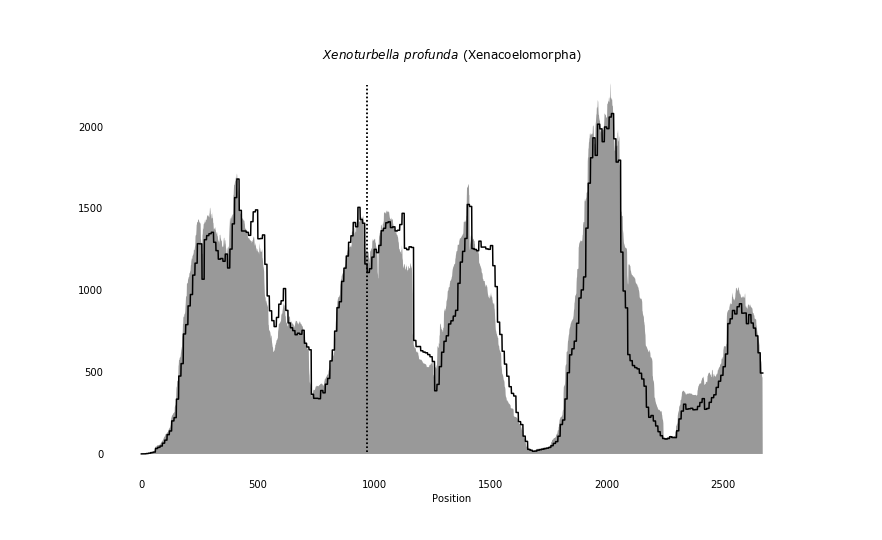

Supplement: Supplementary file 2 — Supplementary information [file 41598_2019_55573_MOESM2_ESM.zip › SupplementaryFile1/Metazoa/Deuterostomia/Xenoturbella_profunda_coverage.png]

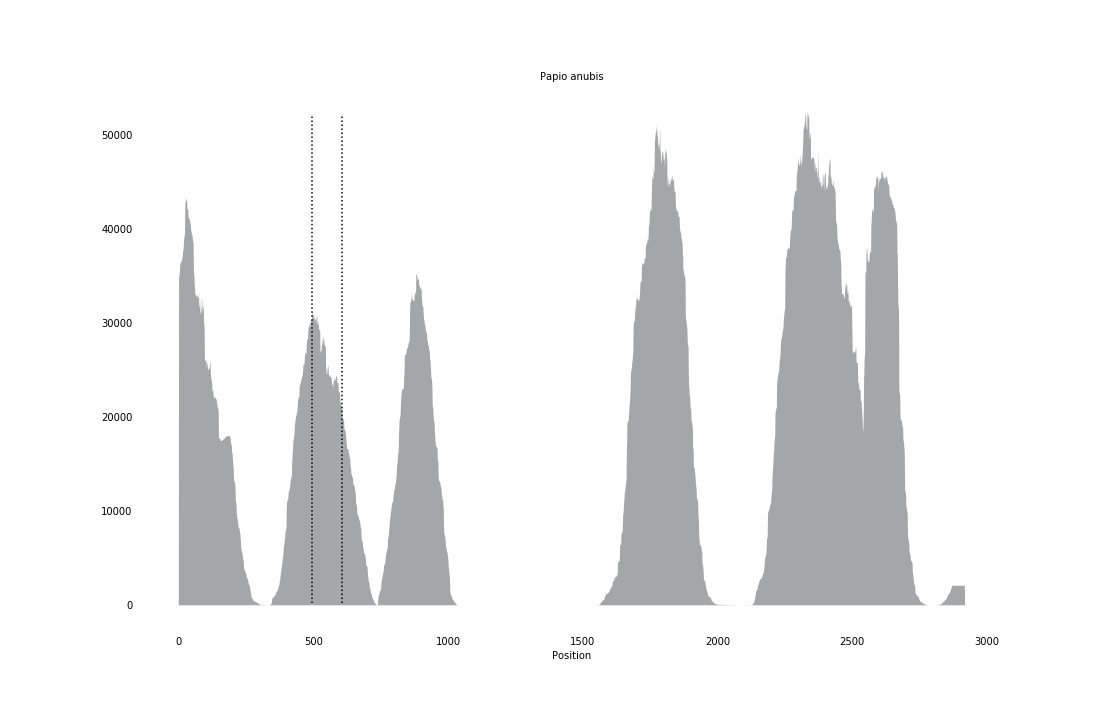

Supplement: Supplementary file 2 — Supplementary information [file 41598_2019_55573_MOESM2_ESM.zip › SupplementaryFile1/Metazoa/Deuterostomia/Papio_anubis_coverage_correct.png]

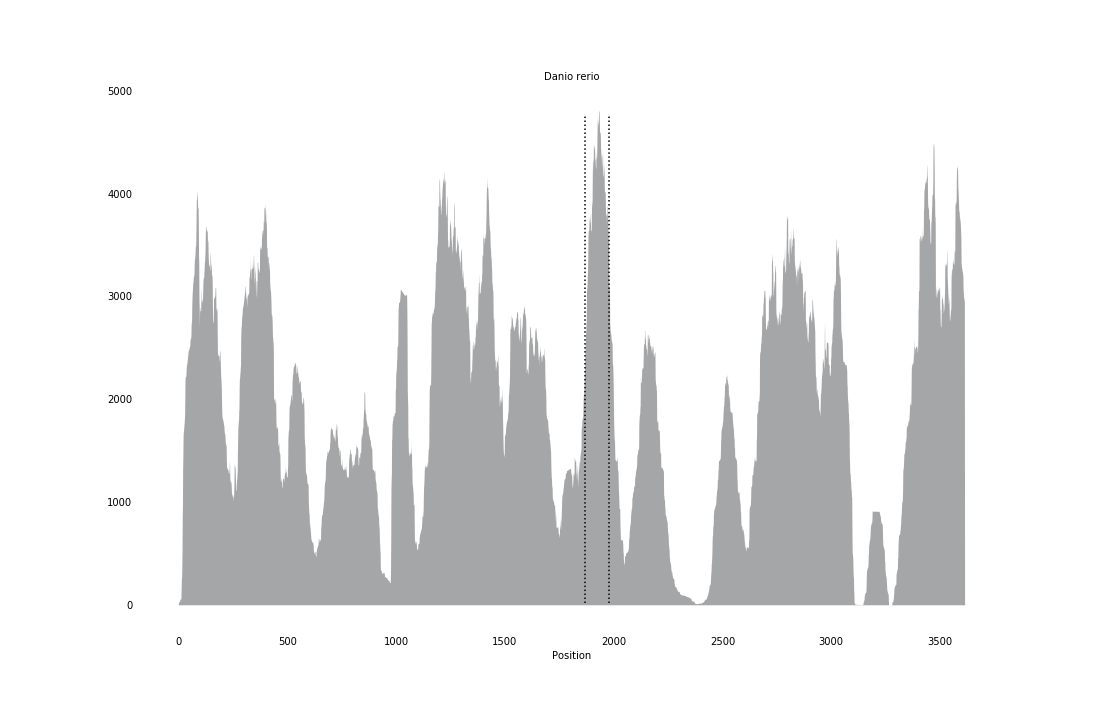

Supplement: Supplementary file 2 — Supplementary information [file 41598_2019_55573_MOESM2_ESM.zip › SupplementaryFile1/Metazoa/Deuterostomia/Danio_rerio_coverage_correct.png]

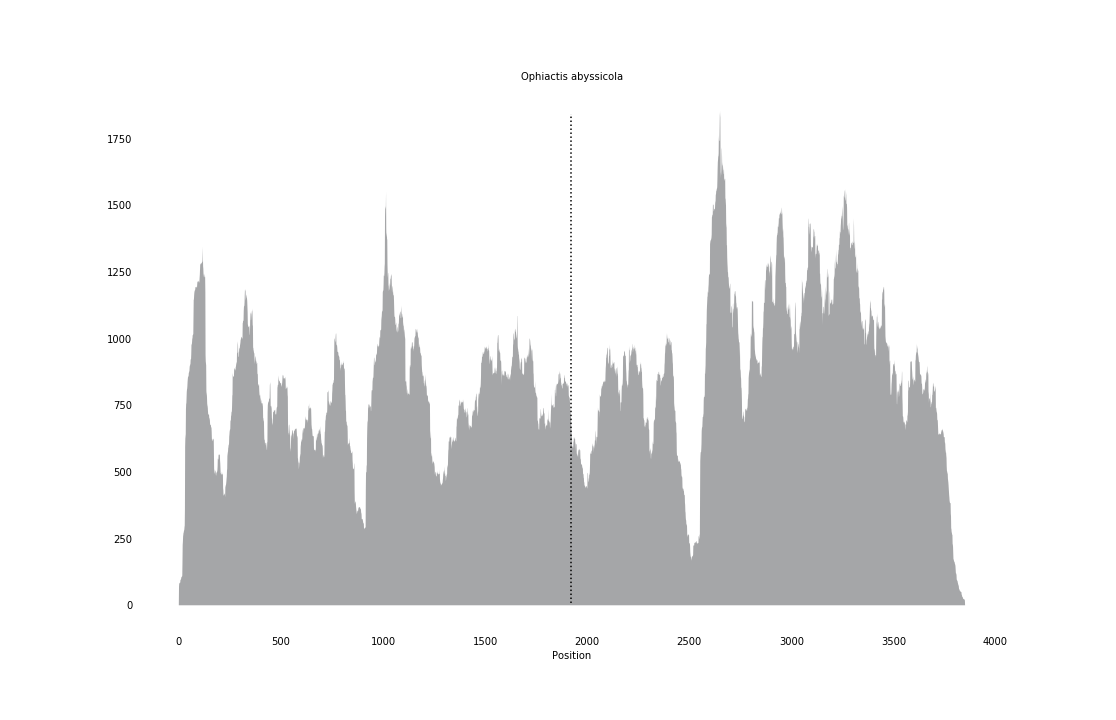

Supplement: Supplementary file 2 — Supplementary information [file 41598_2019_55573_MOESM2_ESM.zip › SupplementaryFile1/Metazoa/Deuterostomia/Ophiactis_abyssicola_coverage.png]

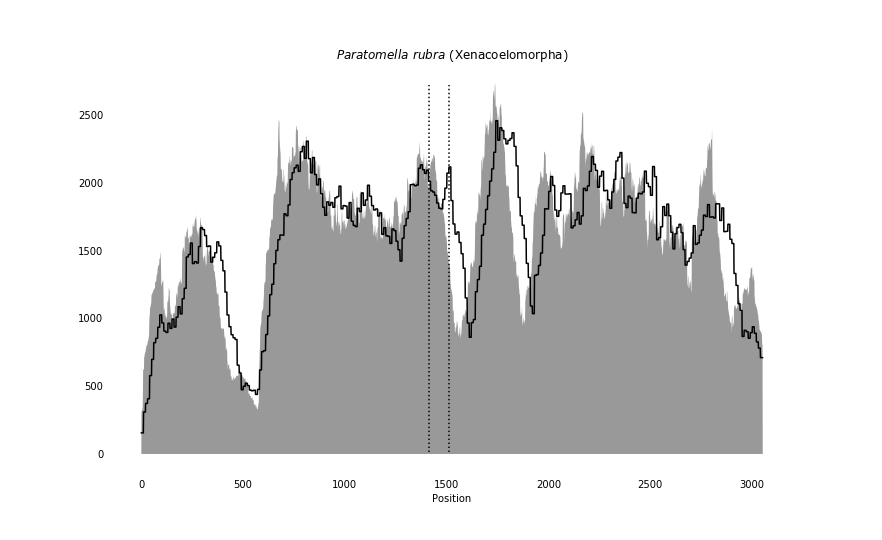

Supplement: Supplementary file 2 — Supplementary information [file 41598_2019_55573_MOESM2_ESM.zip › SupplementaryFile1/Metazoa/Deuterostomia/Paratomella_rubra_coverage.png]

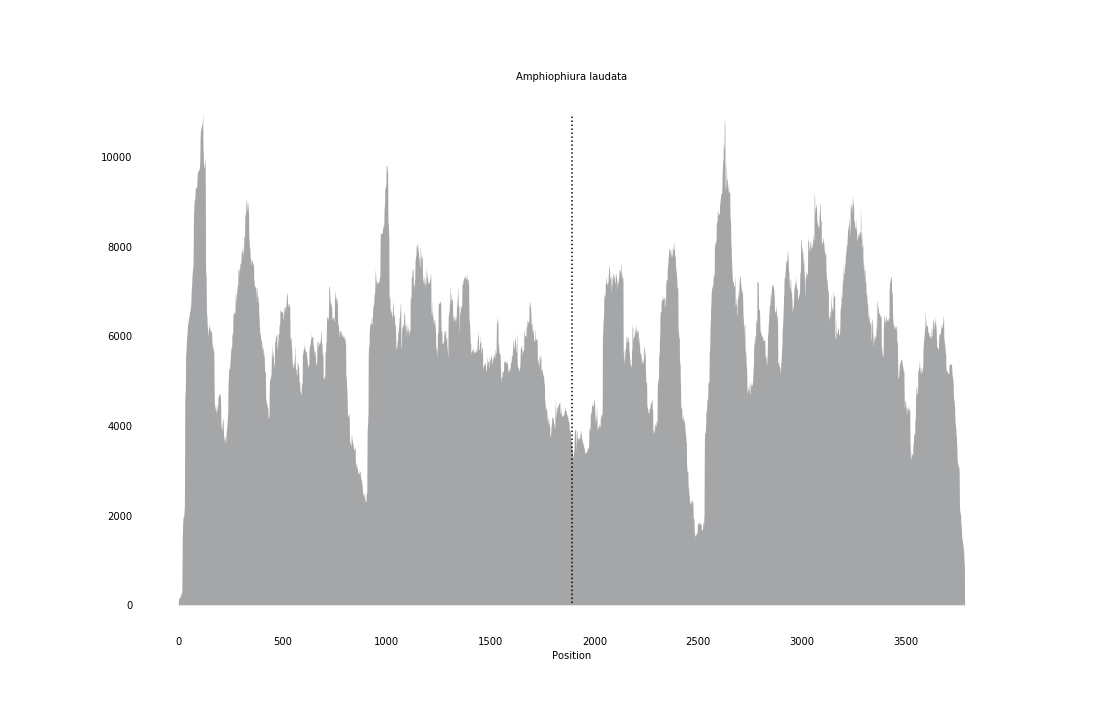

Supplement: Supplementary file 2 — Supplementary information [file 41598_2019_55573_MOESM2_ESM.zip › SupplementaryFile1/Metazoa/Deuterostomia/Amphiophiura_laudata_coverage.png]

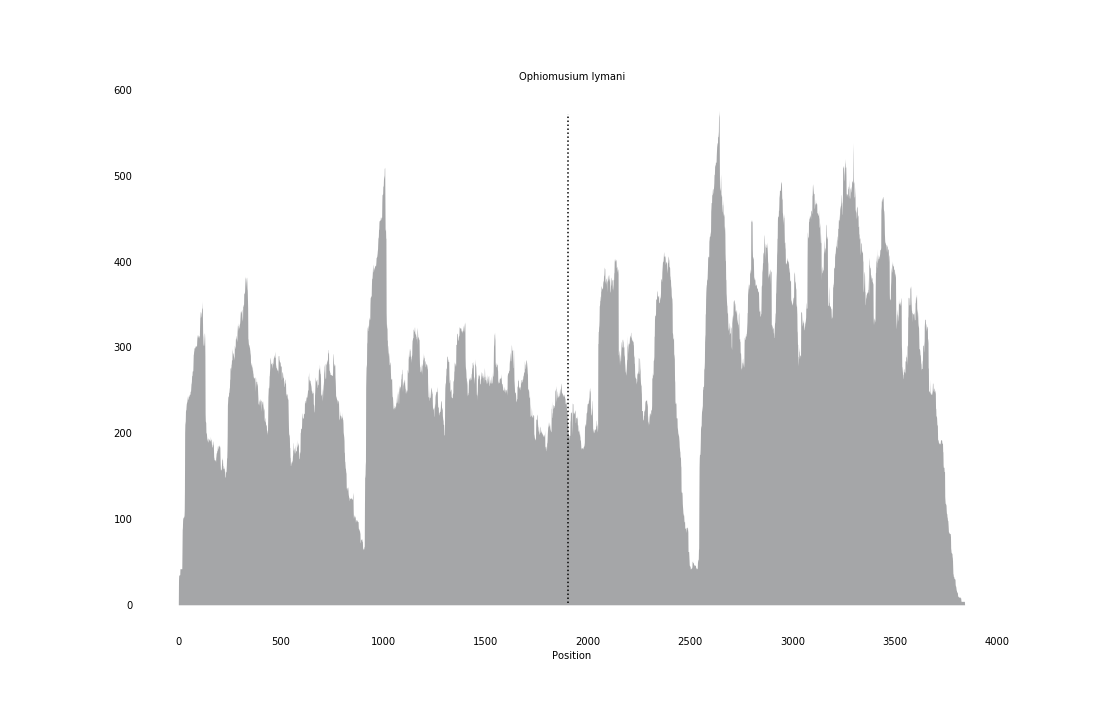

Supplement: Supplementary file 2 — Supplementary information [file 41598_2019_55573_MOESM2_ESM.zip › SupplementaryFile1/Metazoa/Deuterostomia/Ophiomusium_lymani_coverage.png]

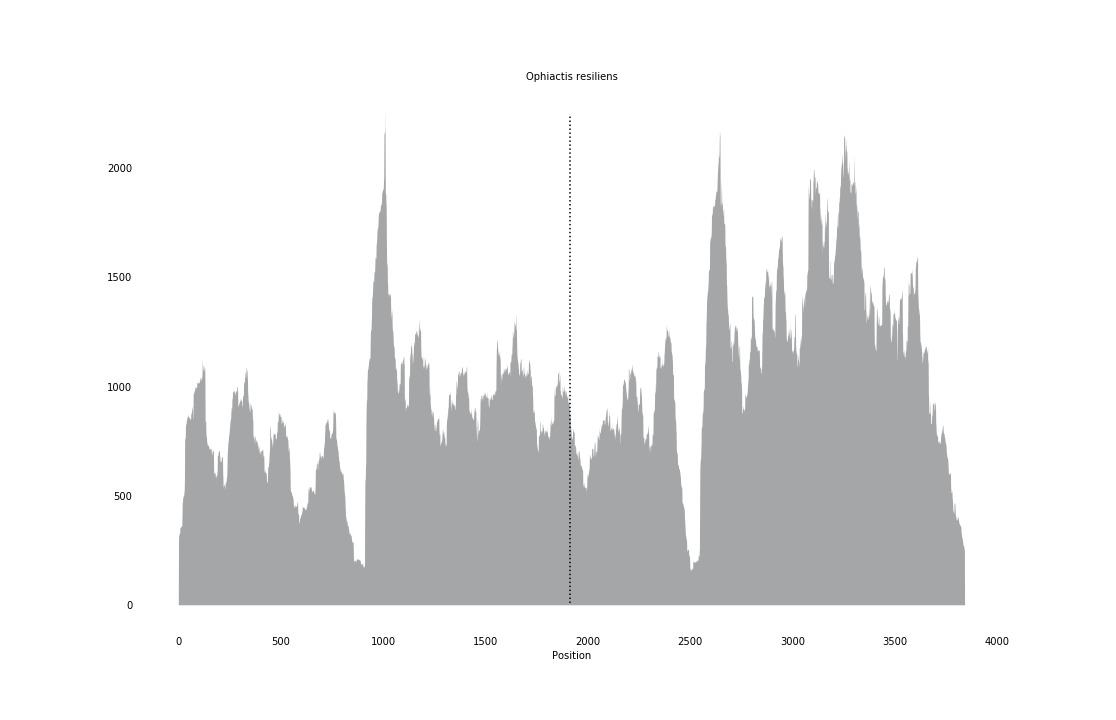

Supplement: Supplementary file 2 — Supplementary information [file 41598_2019_55573_MOESM2_ESM.zip › SupplementaryFile1/Metazoa/Deuterostomia/Ophiactis_resiliens_coverage.png]

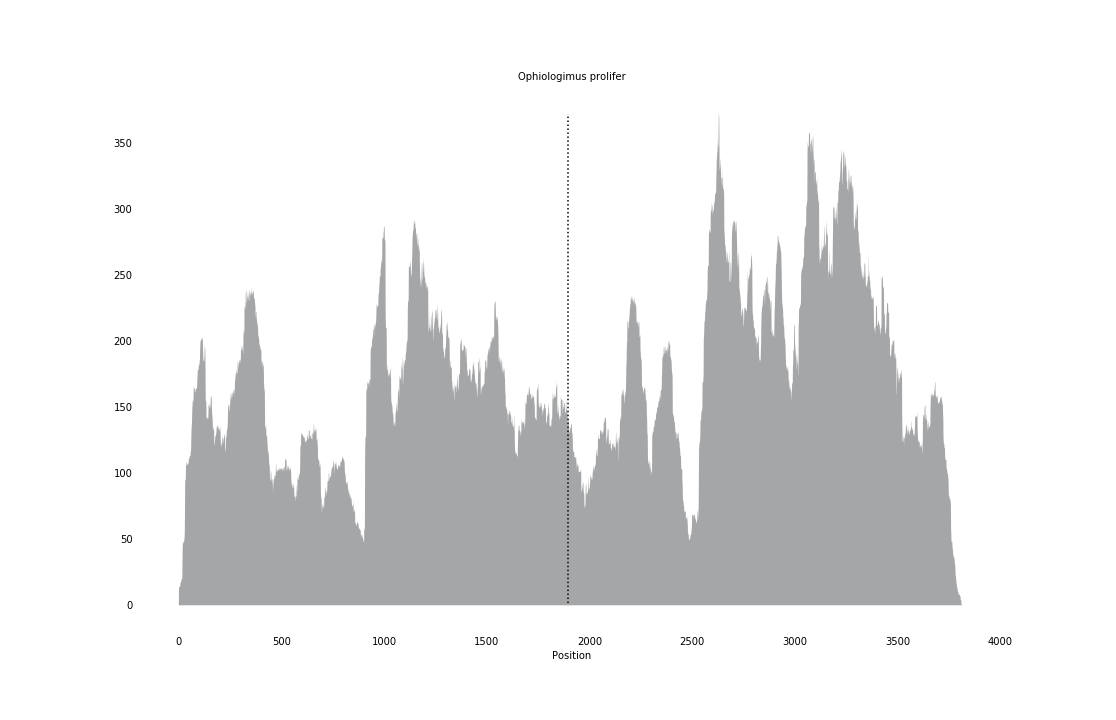

Supplement: Supplementary file 2 — Supplementary information [file 41598_2019_55573_MOESM2_ESM.zip › SupplementaryFile1/Metazoa/Deuterostomia/Ophiologimus_prolifer_coverage.png]

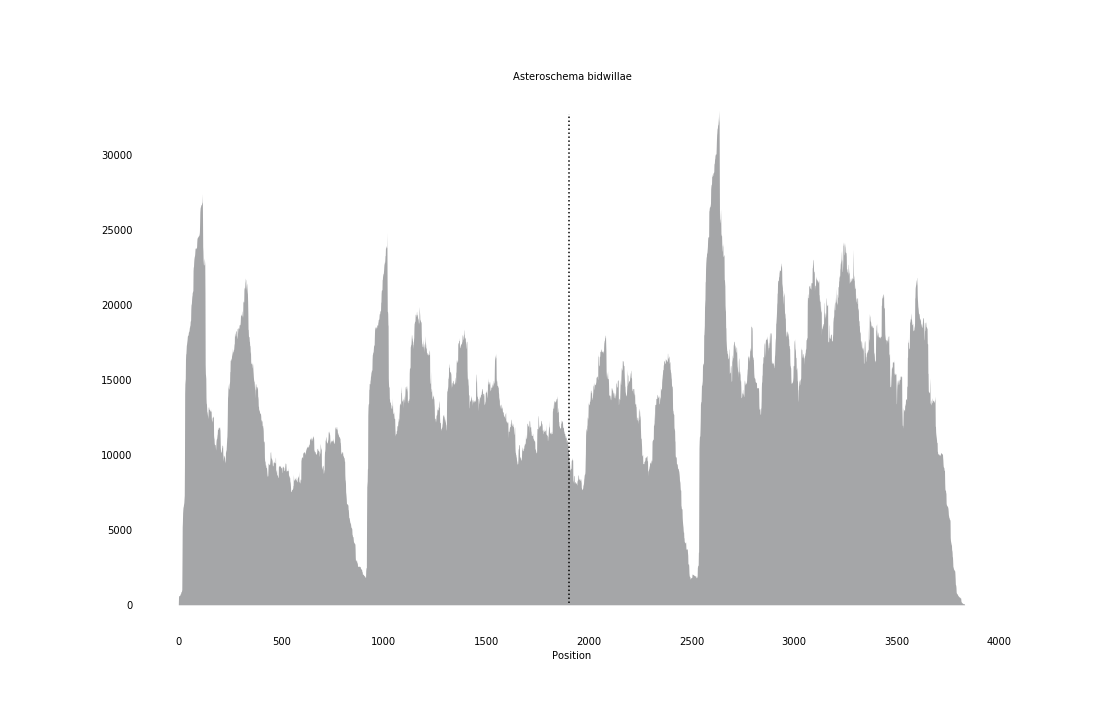

Supplement: Supplementary file 2 — Supplementary information [file 41598_2019_55573_MOESM2_ESM.zip › SupplementaryFile1/Metazoa/Deuterostomia/Asteroschema_bidwillae_coverage.png]

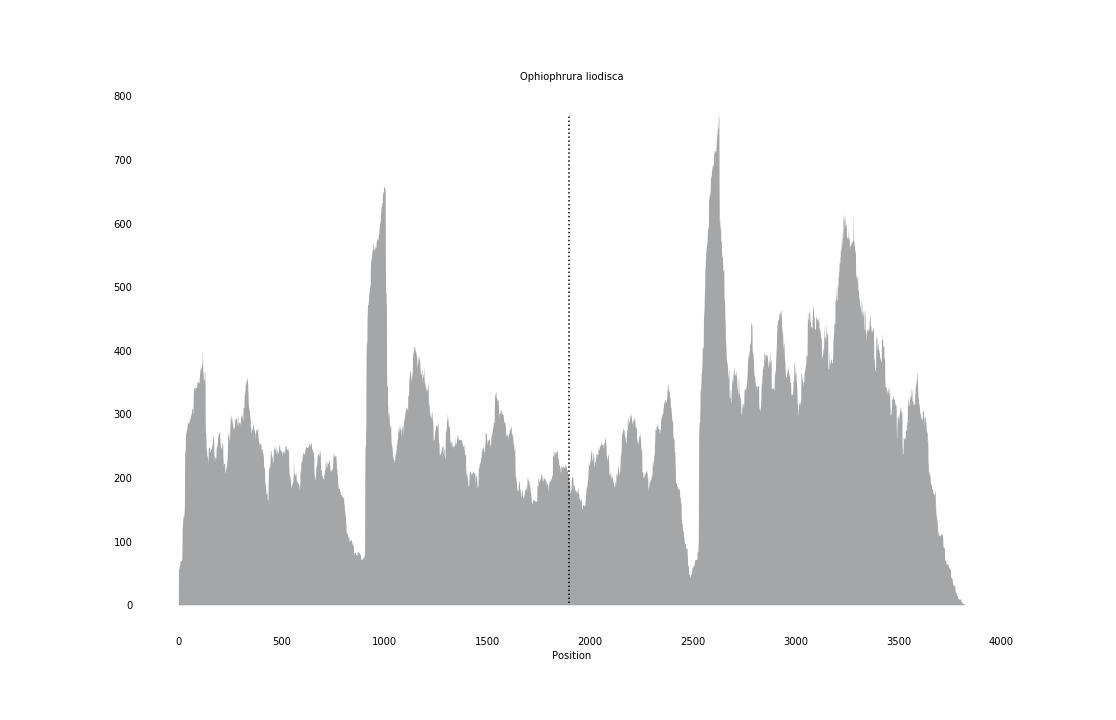

Supplement: Supplementary file 2 — Supplementary information [file 41598_2019_55573_MOESM2_ESM.zip › SupplementaryFile1/Metazoa/Deuterostomia/Ophiophrura_liodisca_coverage.png]

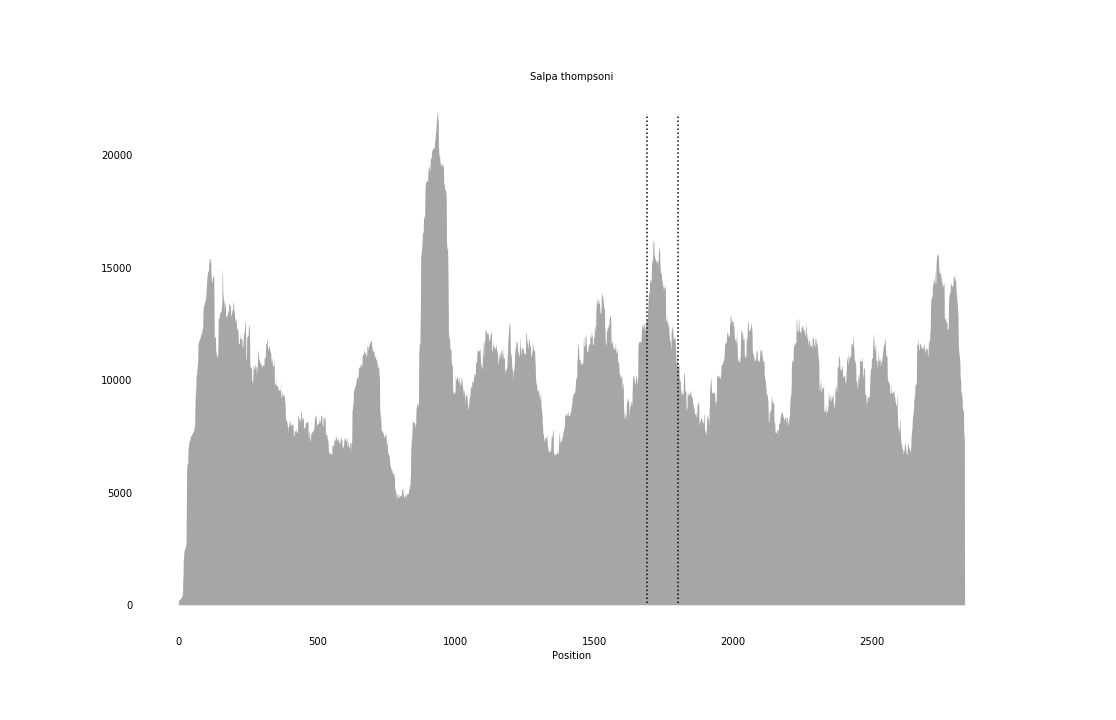

Supplement: Supplementary file 2 — Supplementary information [file 41598_2019_55573_MOESM2_ESM.zip › SupplementaryFile1/Metazoa/Deuterostomia/Salpa_thompsoni_coverage_correct.png]

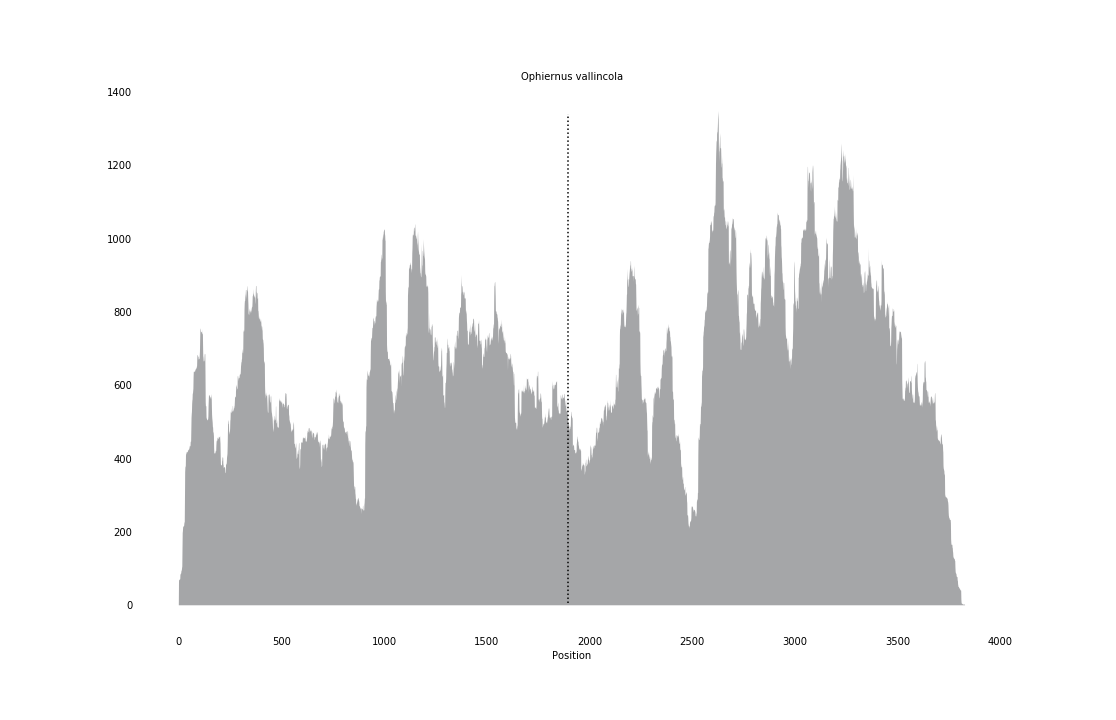

Supplement: Supplementary file 2 — Supplementary information [file 41598_2019_55573_MOESM2_ESM.zip › SupplementaryFile1/Metazoa/Deuterostomia/Ophiernus_vallincola_coverage.png]

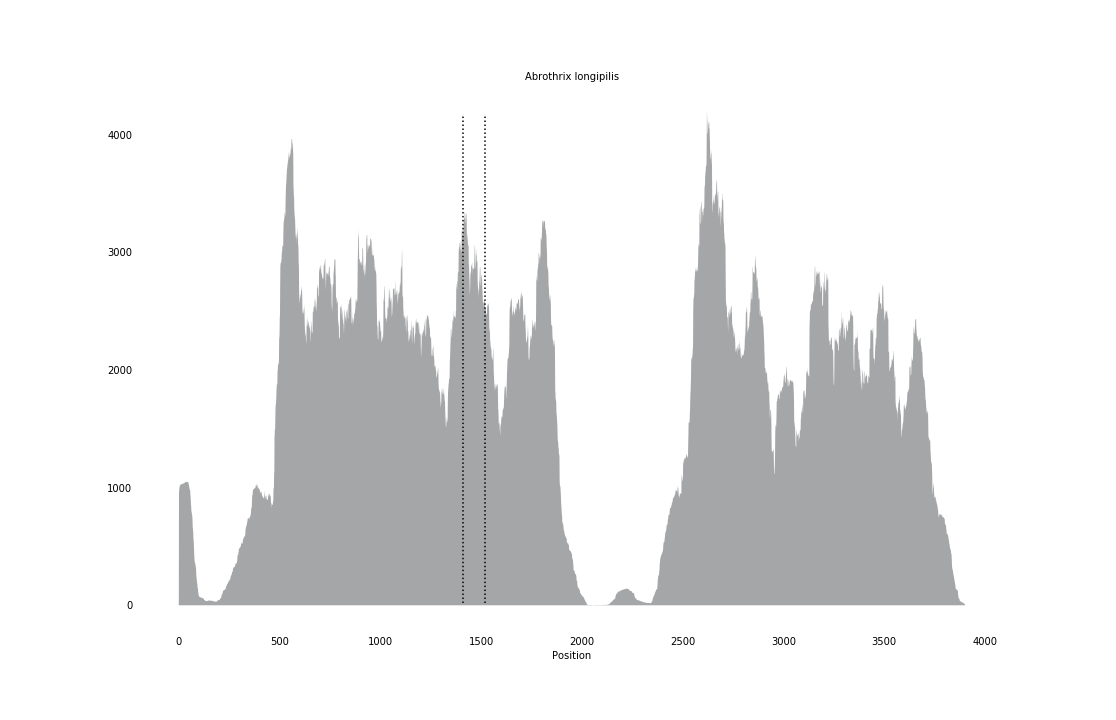

Supplement: Supplementary file 2 — Supplementary information [file 41598_2019_55573_MOESM2_ESM.zip › SupplementaryFile1/Metazoa/Deuterostomia/Abrothrix_longipilis_coverage_correct.png]

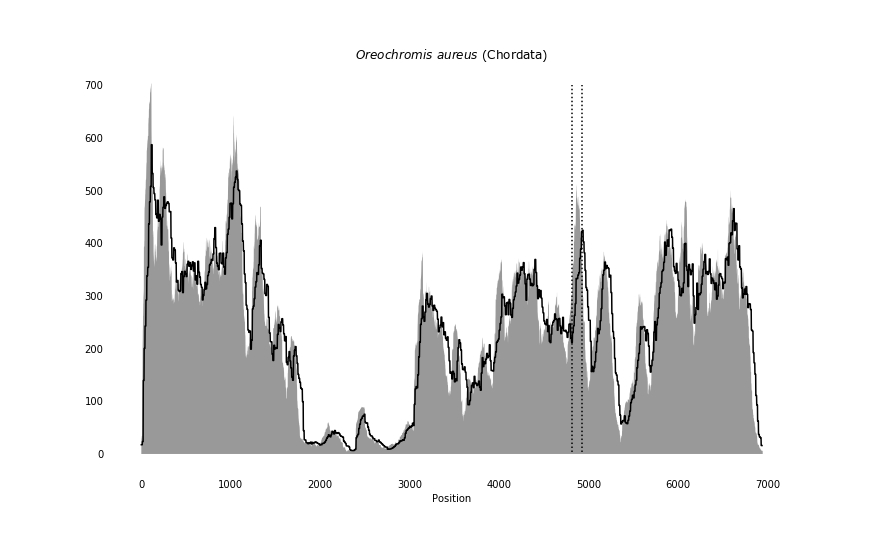

Supplement: Supplementary file 2 — Supplementary information [file 41598_2019_55573_MOESM2_ESM.zip › SupplementaryFile1/Metazoa/Deuterostomia/Oreochromis_aureus_coverage.png]

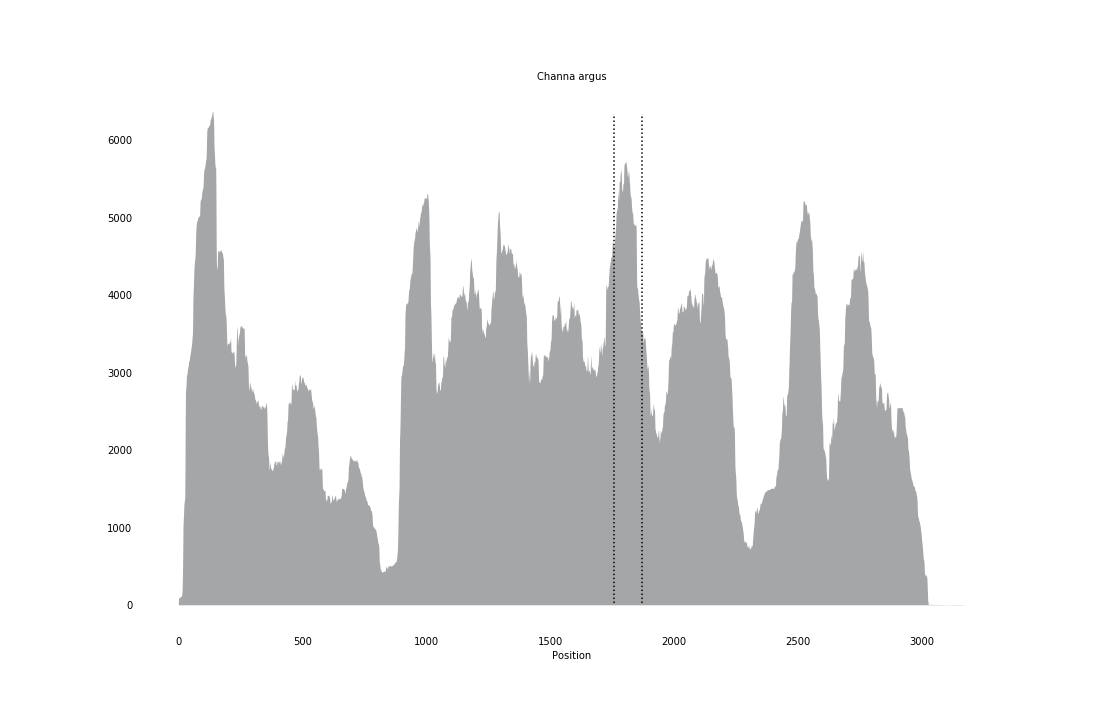

Supplement: Supplementary file 2 — Supplementary information [file 41598_2019_55573_MOESM2_ESM.zip › SupplementaryFile1/Metazoa/Deuterostomia/Channa_argus_coverage_correct.png]

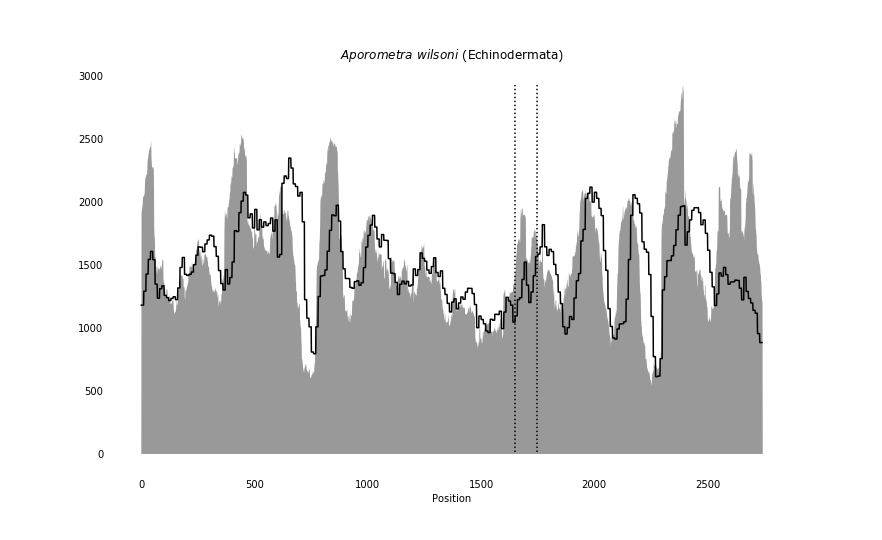

Supplement: Supplementary file 2 — Supplementary information [file 41598_2019_55573_MOESM2_ESM.zip › SupplementaryFile1/Metazoa/Deuterostomia/Aporometra_wilsoni_coverage.png]

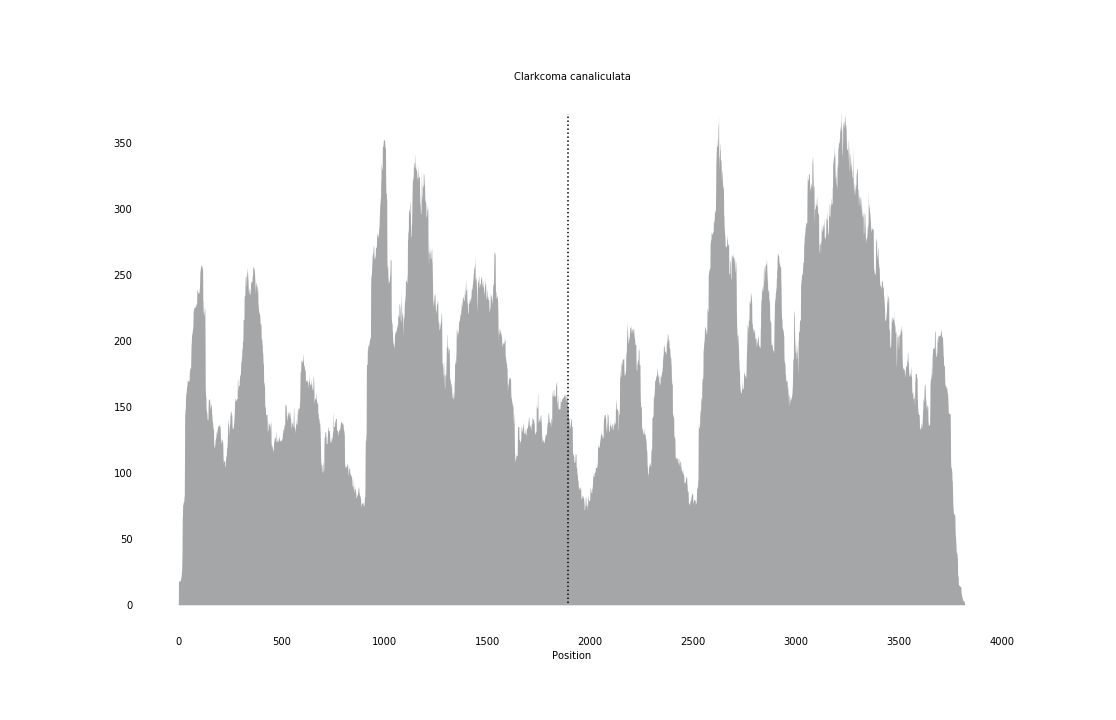

Supplement: Supplementary file 2 — Supplementary information [file 41598_2019_55573_MOESM2_ESM.zip › SupplementaryFile1/Metazoa/Deuterostomia/Clarkcoma_canaliculata_coverage.png]

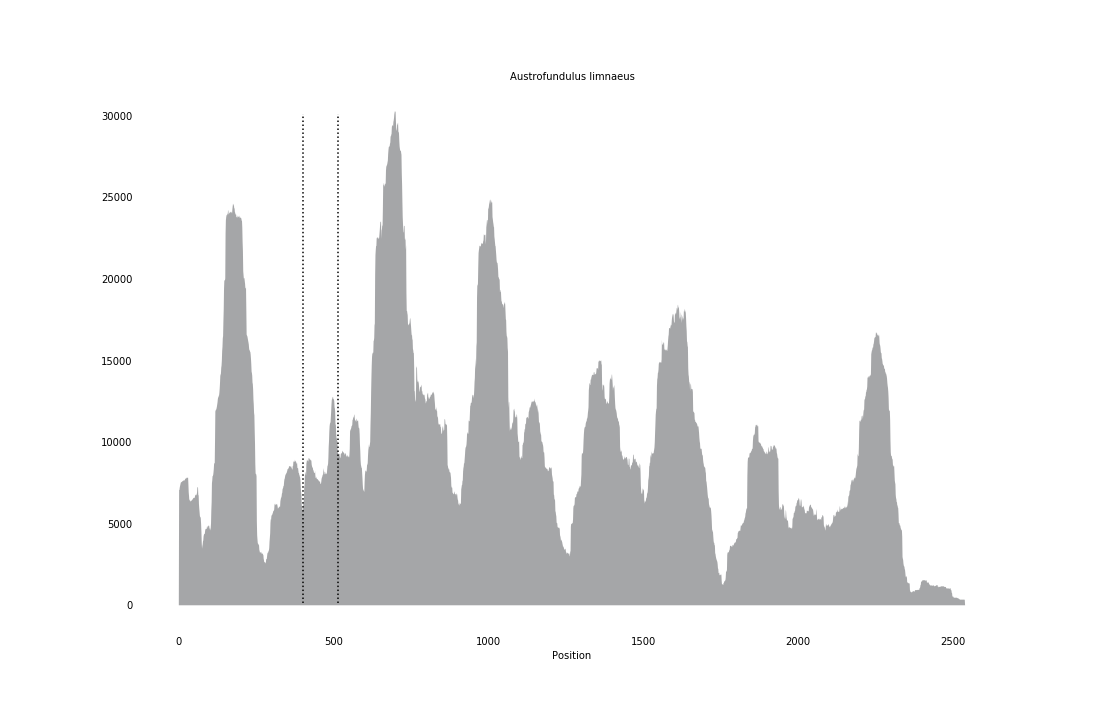

Supplement: Supplementary file 2 — Supplementary information [file 41598_2019_55573_MOESM2_ESM.zip › SupplementaryFile1/Metazoa/Deuterostomia/Austrofundulus_limnaeus_coverage_correct.png]

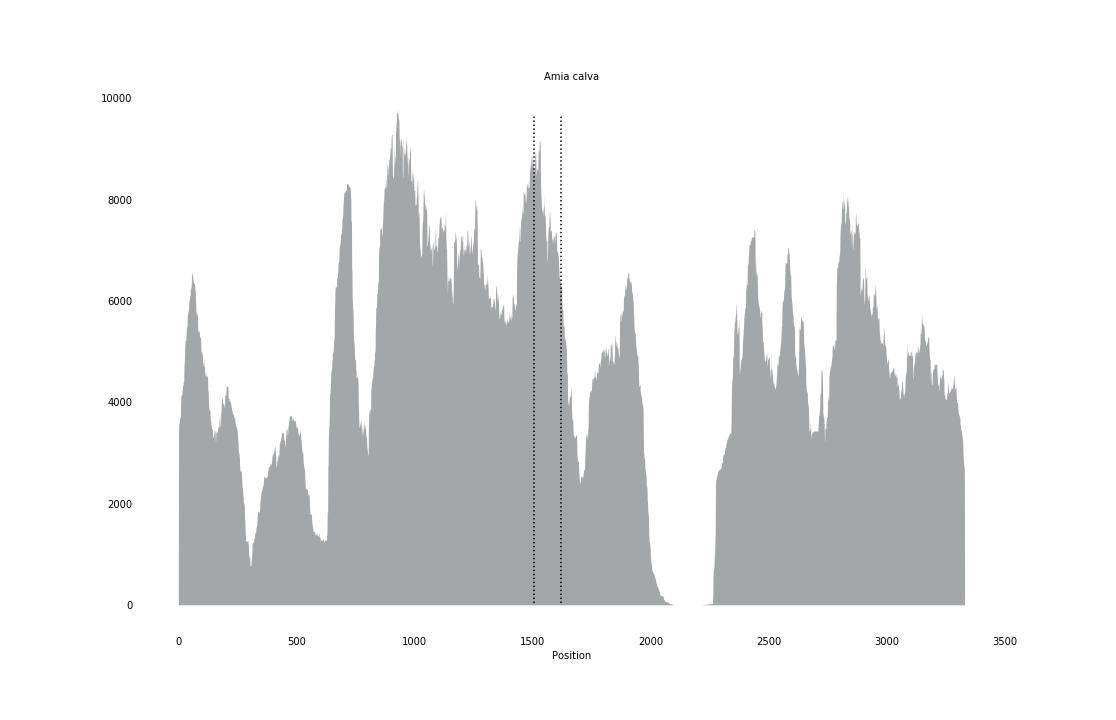

Supplement: Supplementary file 2 — Supplementary information [file 41598_2019_55573_MOESM2_ESM.zip › SupplementaryFile1/Metazoa/Deuterostomia/Amia_calva_coverage_correct.png]

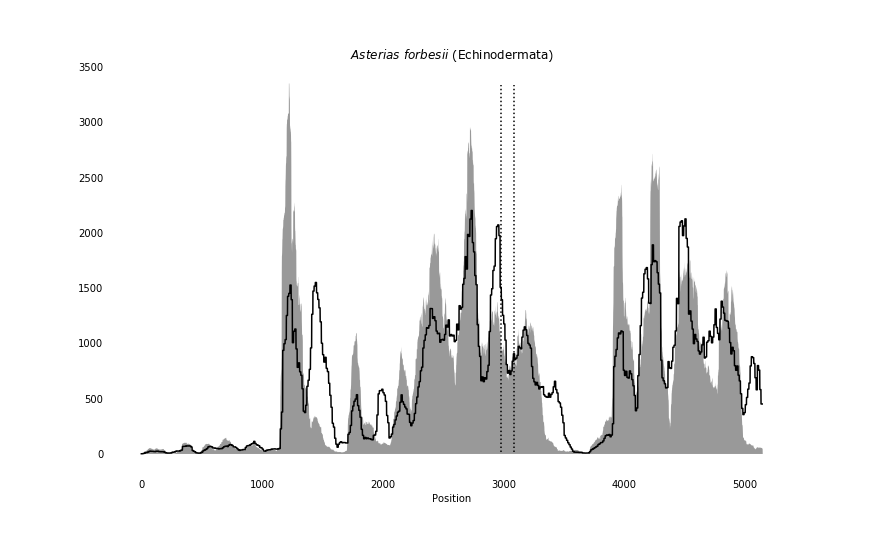

Supplement: Supplementary file 2 — Supplementary information [file 41598_2019_55573_MOESM2_ESM.zip › SupplementaryFile1/Metazoa/Deuterostomia/Asterias_forbesii_coverage.png]

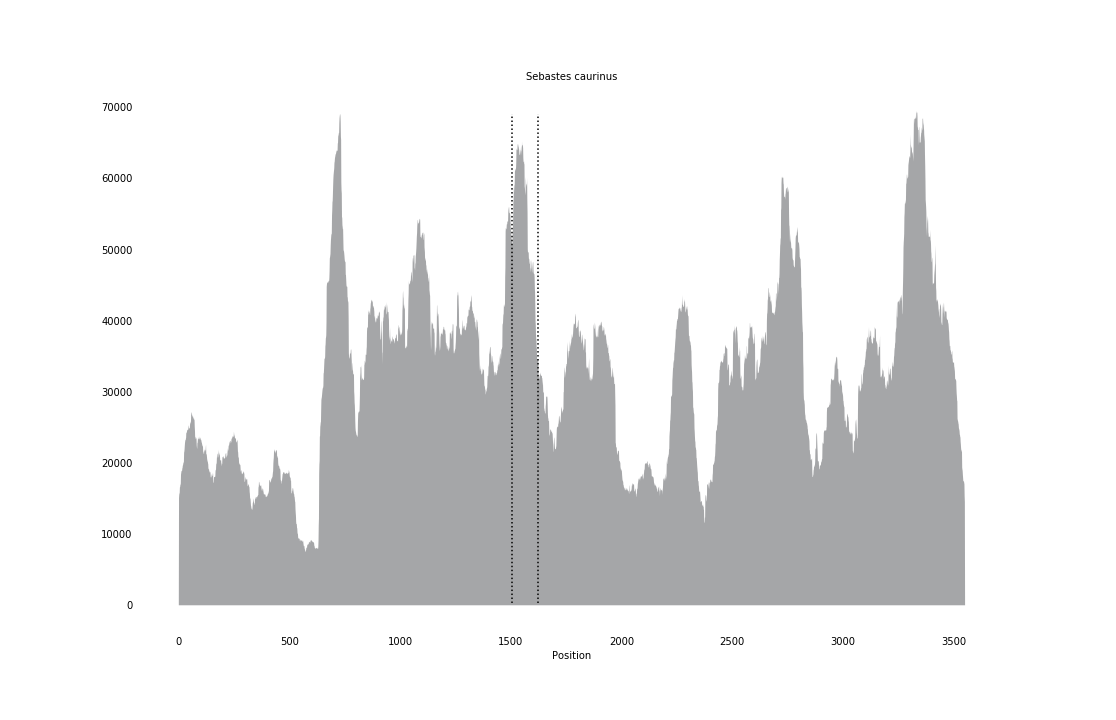

Supplement: Supplementary file 2 — Supplementary information [file 41598_2019_55573_MOESM2_ESM.zip › SupplementaryFile1/Metazoa/Deuterostomia/Sebastes_caurinus_coverage_correct.png]

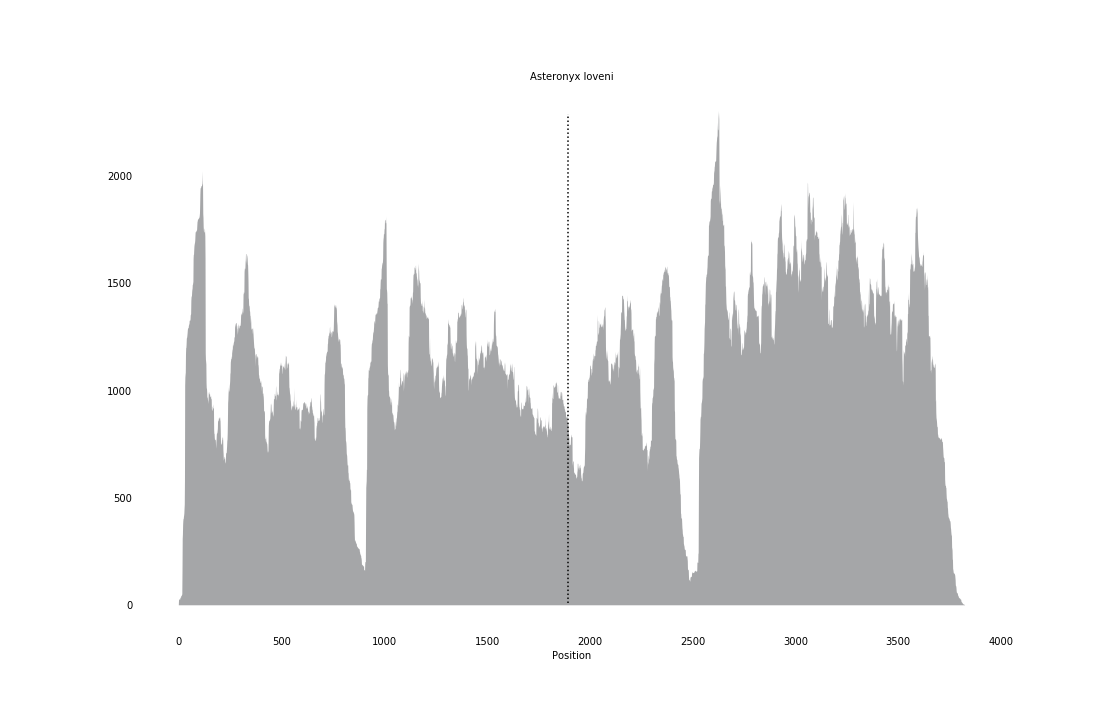

Supplement: Supplementary file 2 — Supplementary information [file 41598_2019_55573_MOESM2_ESM.zip › SupplementaryFile1/Metazoa/Deuterostomia/Asteronyx_loveni_coverage.png]

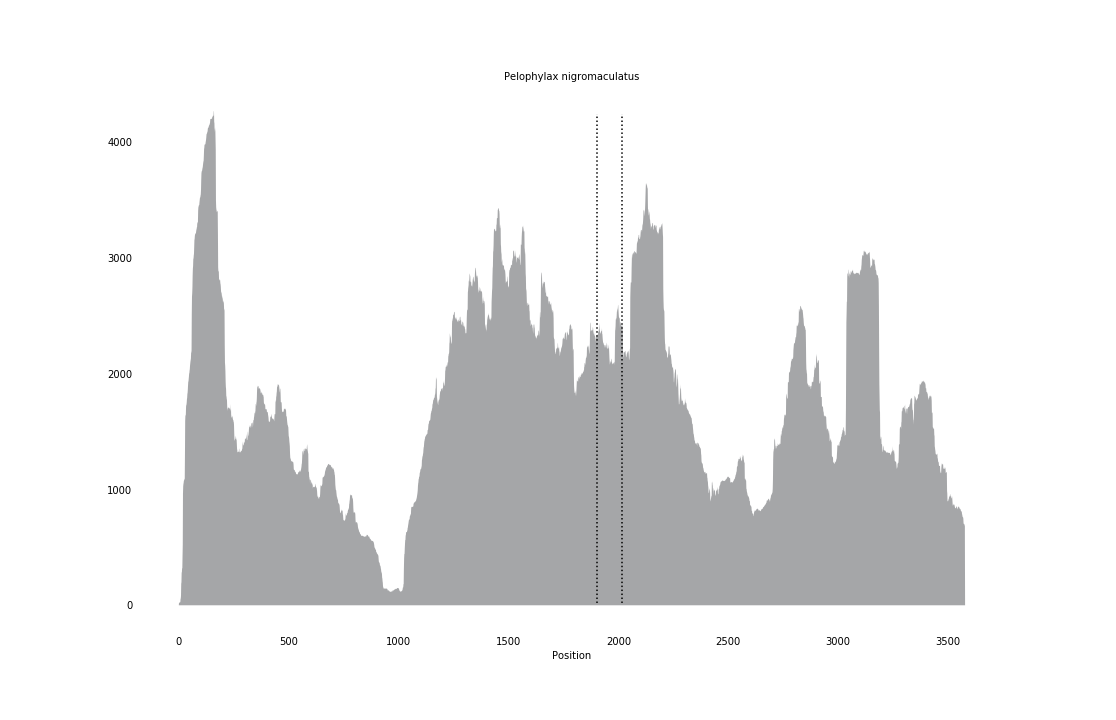

Supplement: Supplementary file 2 — Supplementary information [file 41598_2019_55573_MOESM2_ESM.zip › SupplementaryFile1/Metazoa/Deuterostomia/Pelophylax_nigromaculatus_coverage_correct.png]

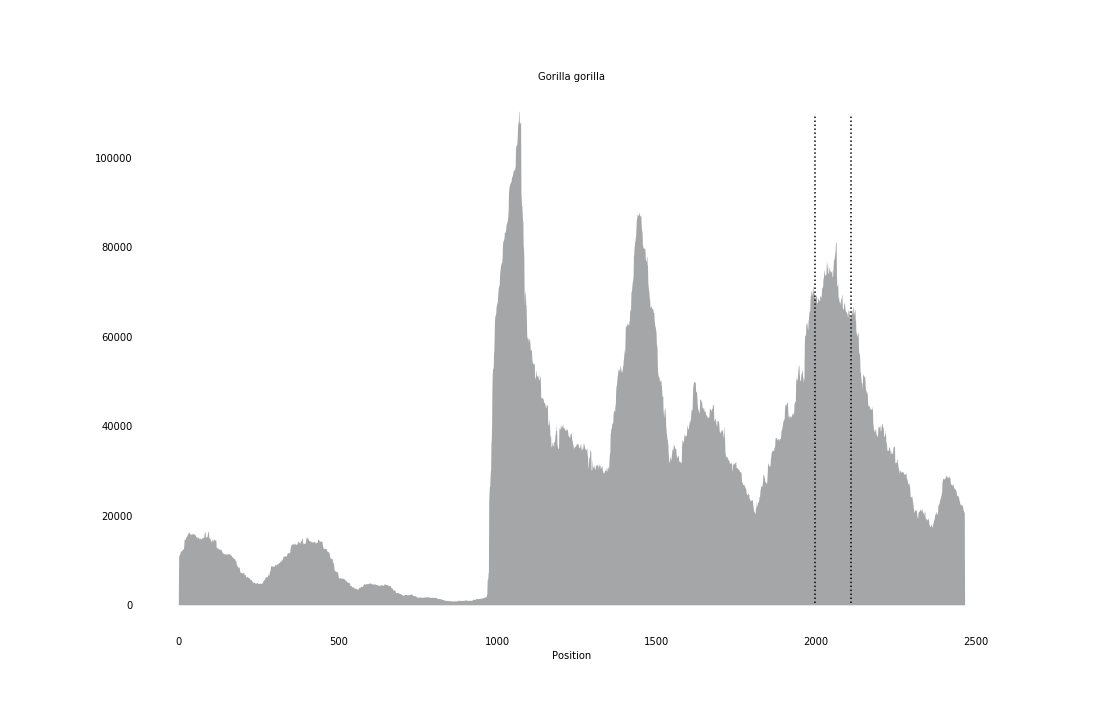

Supplement: Supplementary file 2 — Supplementary information [file 41598_2019_55573_MOESM2_ESM.zip › SupplementaryFile1/Metazoa/Deuterostomia/Gorilla_gorilla_coverage_correct.png]

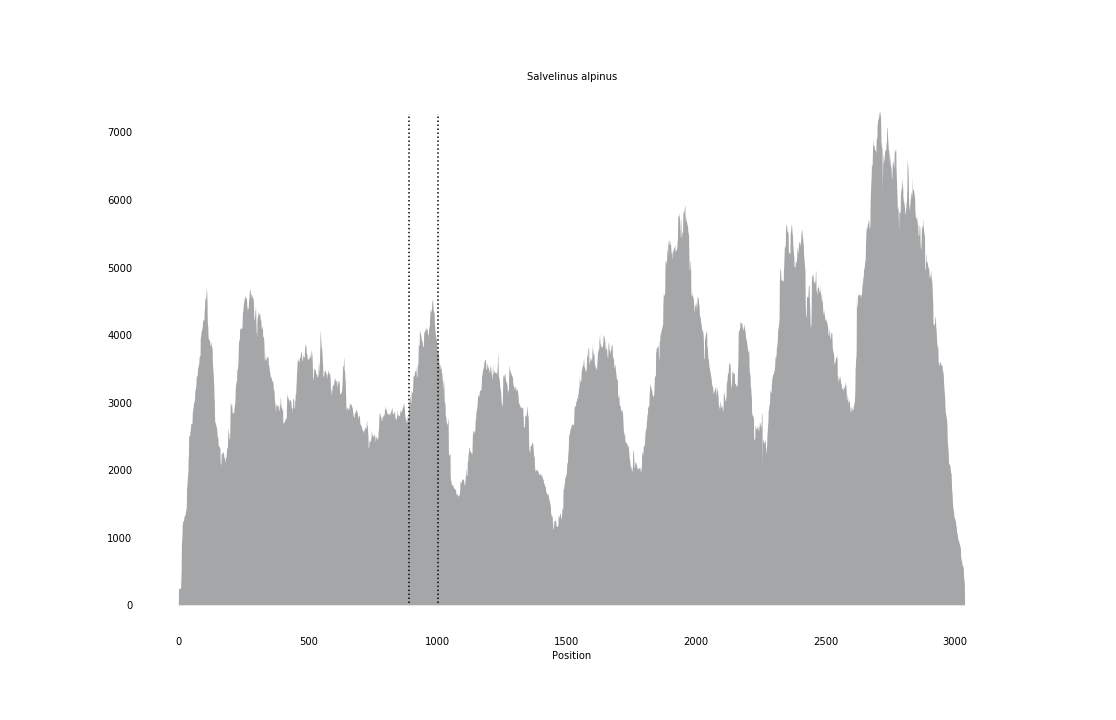

Supplement: Supplementary file 2 — Supplementary information [file 41598_2019_55573_MOESM2_ESM.zip › SupplementaryFile1/Metazoa/Deuterostomia/Salvelinus_alpinus_coverage_correct.png]

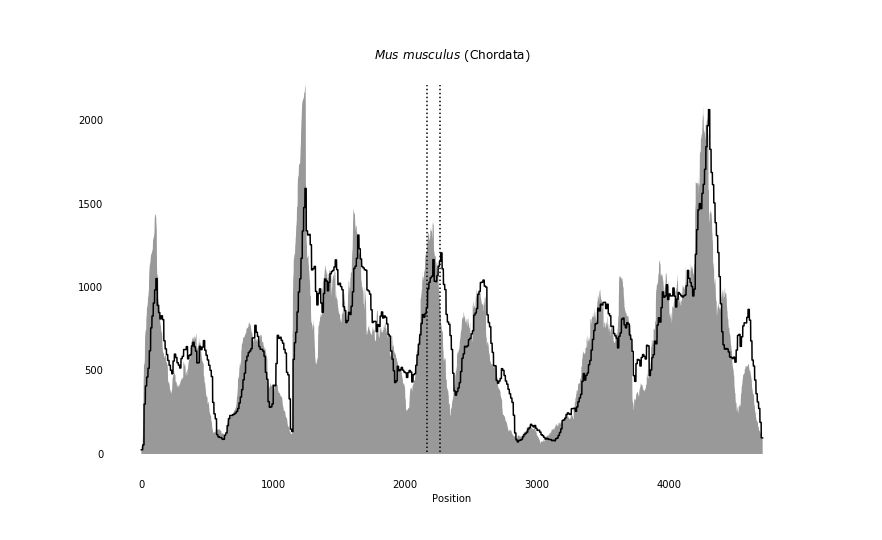

Supplement: Supplementary file 2 — Supplementary information [file 41598_2019_55573_MOESM2_ESM.zip › SupplementaryFile1/Metazoa/Deuterostomia/Mus_musculus_coverage.png]

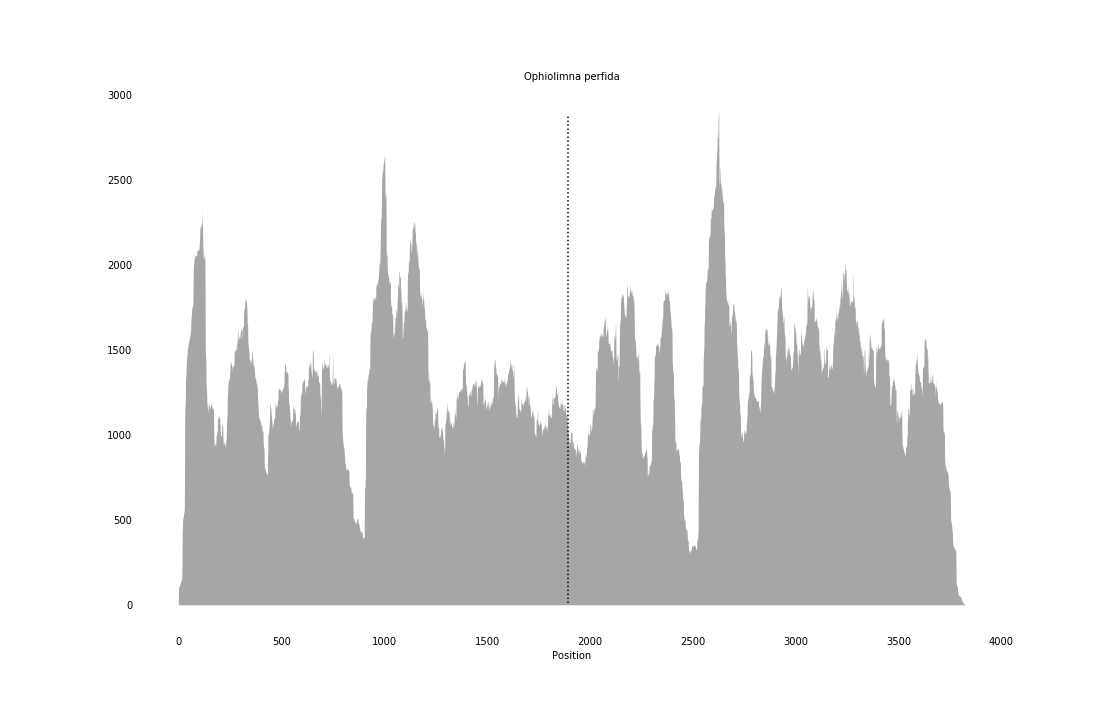

Supplement: Supplementary file 2 — Supplementary information [file 41598_2019_55573_MOESM2_ESM.zip › SupplementaryFile1/Metazoa/Deuterostomia/Ophiolimna_perfida_coverage.png]

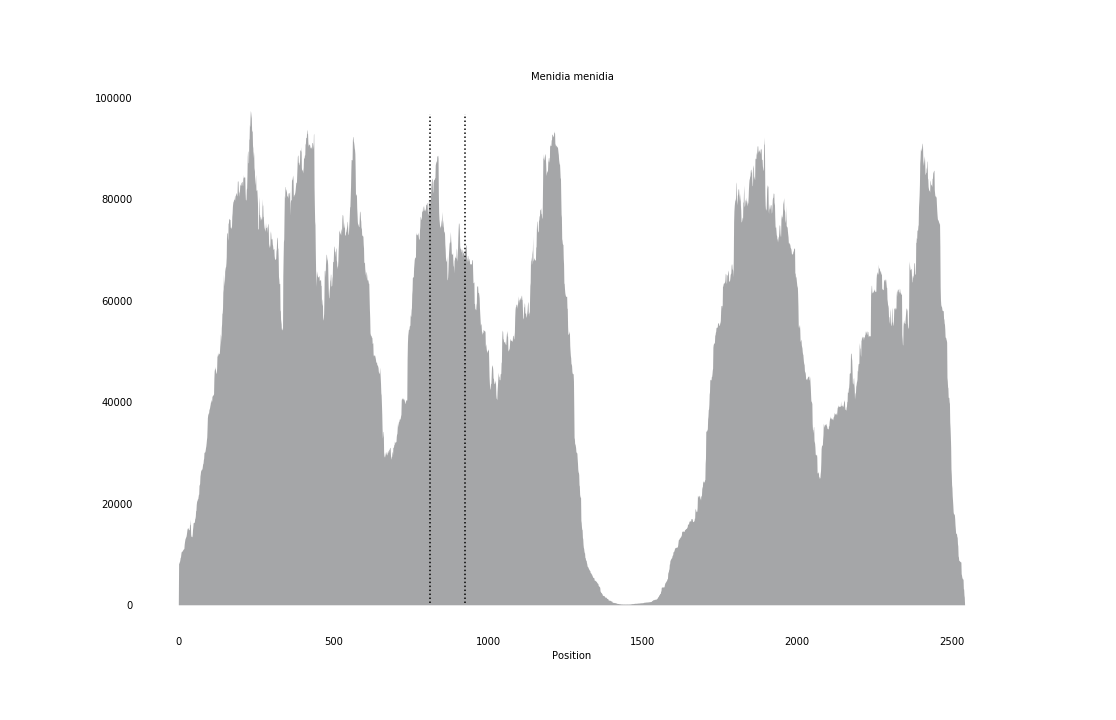

Supplement: Supplementary file 2 — Supplementary information [file 41598_2019_55573_MOESM2_ESM.zip › SupplementaryFile1/Metazoa/Deuterostomia/Menidia_menidia_coverage_correct.png]

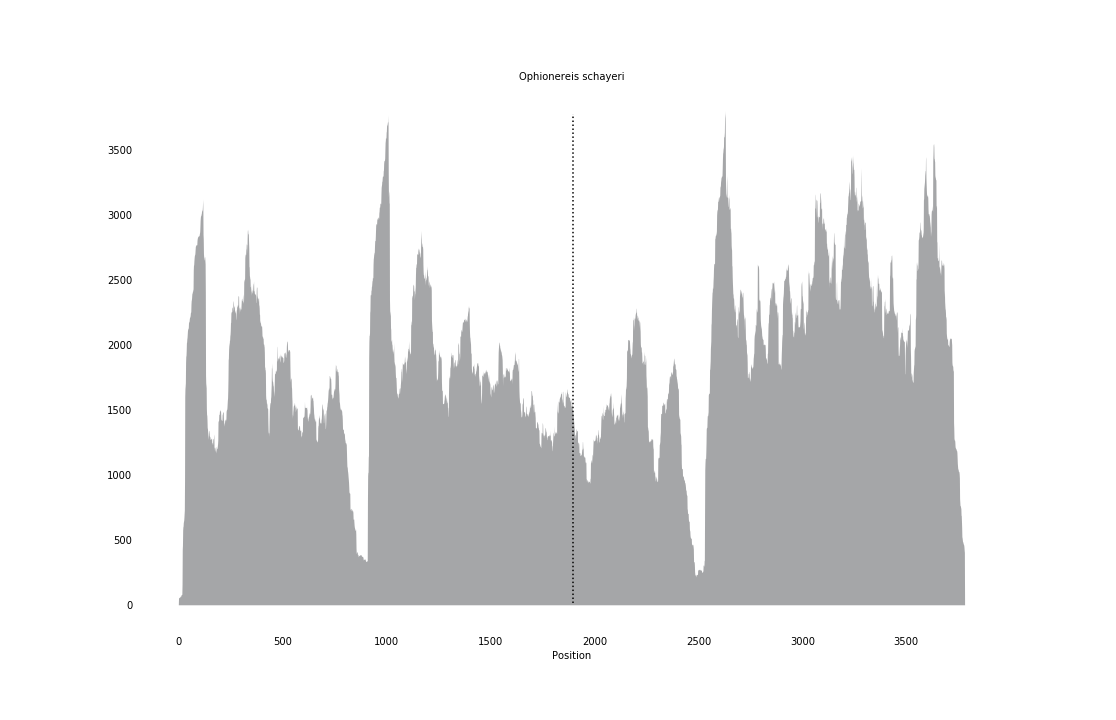

Supplement: Supplementary file 2 — Supplementary information [file 41598_2019_55573_MOESM2_ESM.zip › SupplementaryFile1/Metazoa/Deuterostomia/Ophionereis_schayeri_coverage.png]

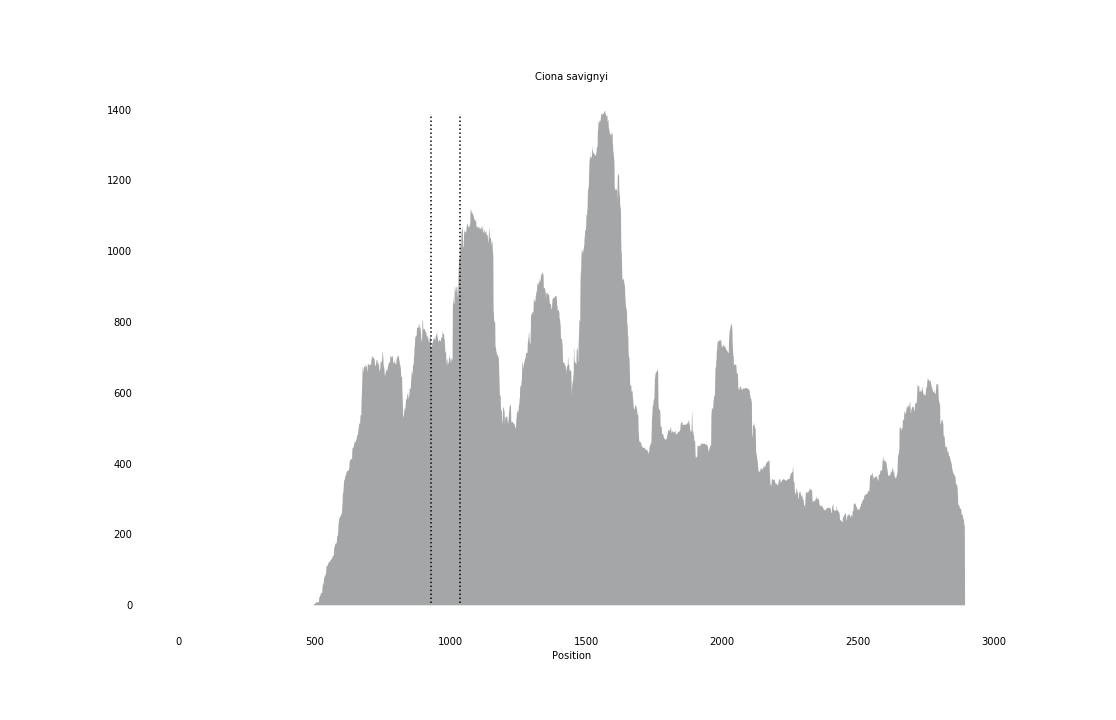

Supplement: Supplementary file 2 — Supplementary information [file 41598_2019_55573_MOESM2_ESM.zip › SupplementaryFile1/Metazoa/Deuterostomia/Ciona_savignyi_coverage_correct.png]

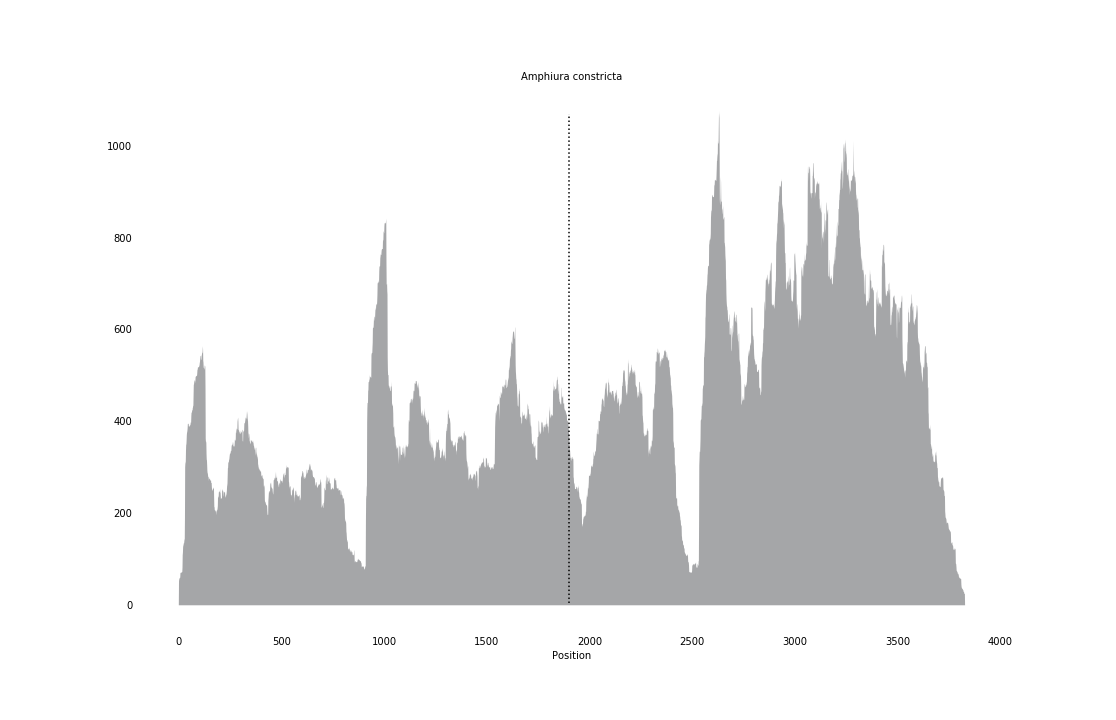

Supplement: Supplementary file 2 — Supplementary information [file 41598_2019_55573_MOESM2_ESM.zip › SupplementaryFile1/Metazoa/Deuterostomia/Amphiura_constricta_coverage.png]

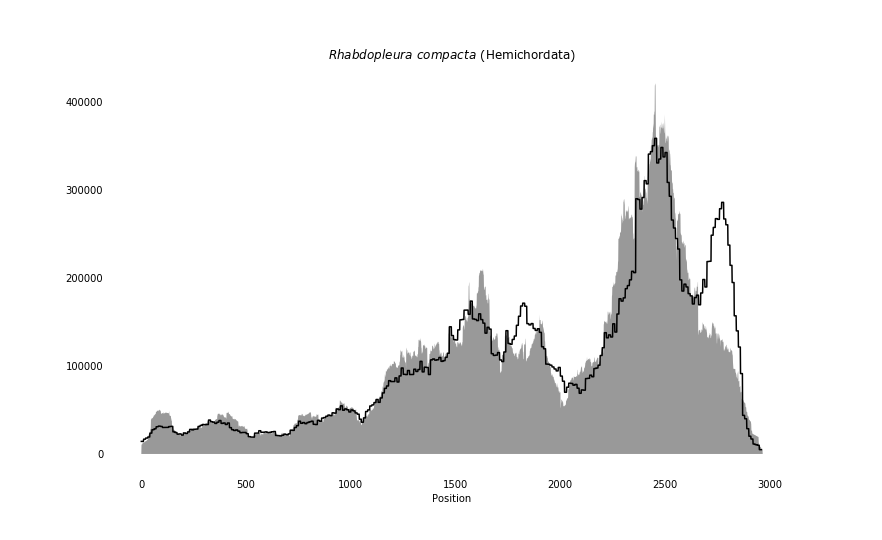

Supplement: Supplementary file 2 — Supplementary information [file 41598_2019_55573_MOESM2_ESM.zip › SupplementaryFile1/Metazoa/Deuterostomia/Rhabdopleura_compacta_coverage.png]

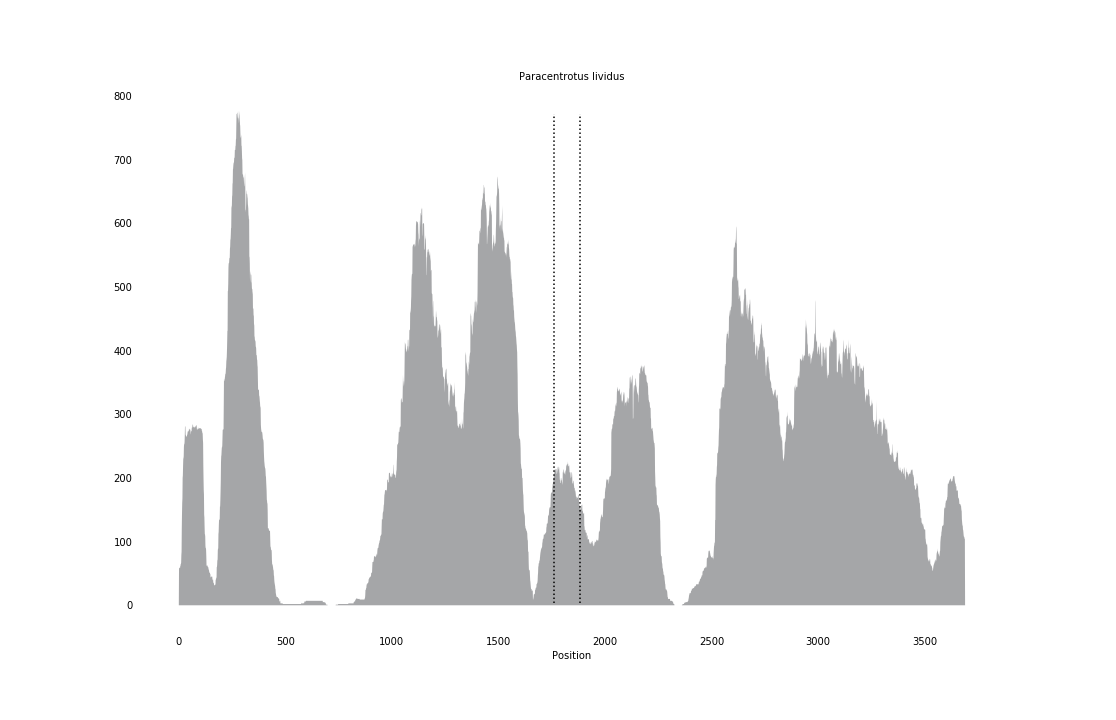

Supplement: Supplementary file 2 — Supplementary information [file 41598_2019_55573_MOESM2_ESM.zip › SupplementaryFile1/Metazoa/Deuterostomia/Paracentrotus_lividus_coverage_correct.png]

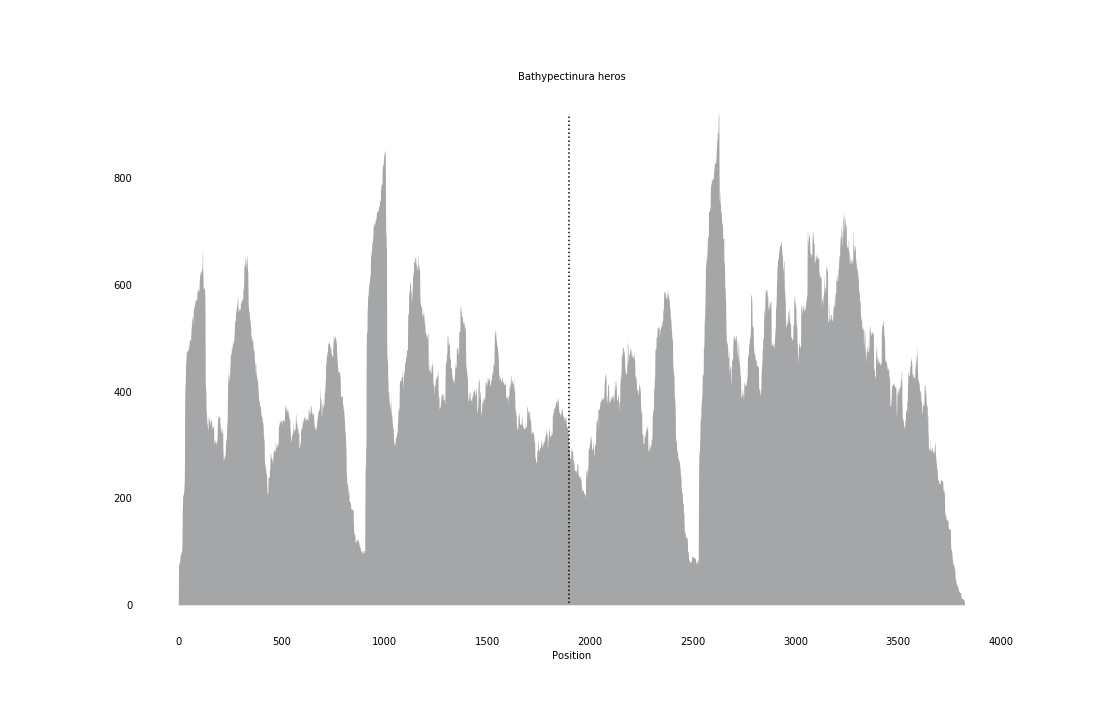

Supplement: Supplementary file 2 — Supplementary information [file 41598_2019_55573_MOESM2_ESM.zip › SupplementaryFile1/Metazoa/Deuterostomia/Bathypectinura_heros_coverage.png]

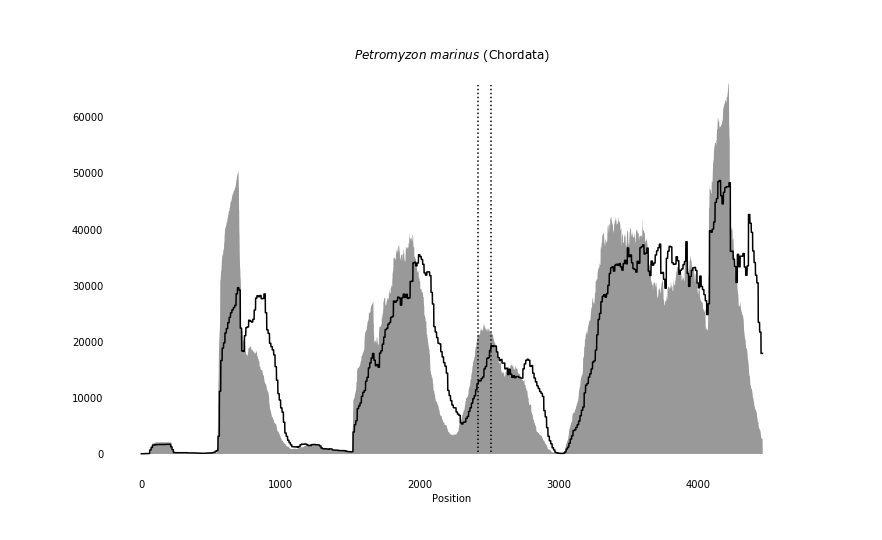

Supplement: Supplementary file 2 — Supplementary information [file 41598_2019_55573_MOESM2_ESM.zip › SupplementaryFile1/Metazoa/Deuterostomia/Petromyzon_marinus_coverage.png]

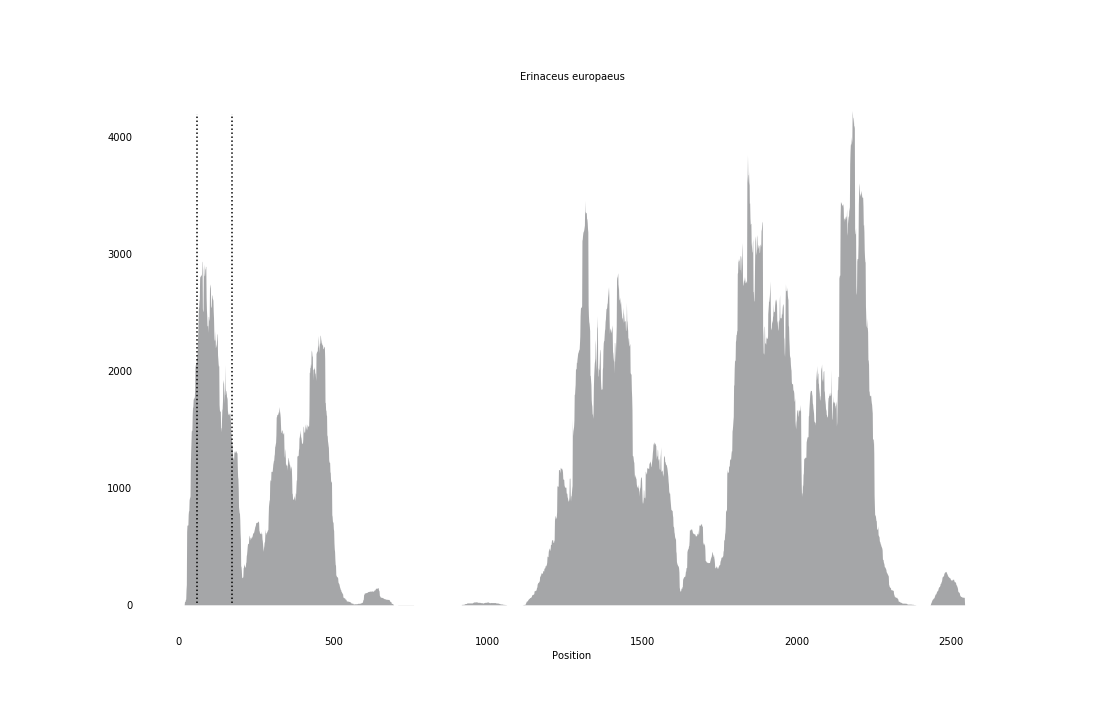

Supplement: Supplementary file 2 — Supplementary information [file 41598_2019_55573_MOESM2_ESM.zip › SupplementaryFile1/Metazoa/Deuterostomia/Erinaceus_europaeus_coverage_correct.png]

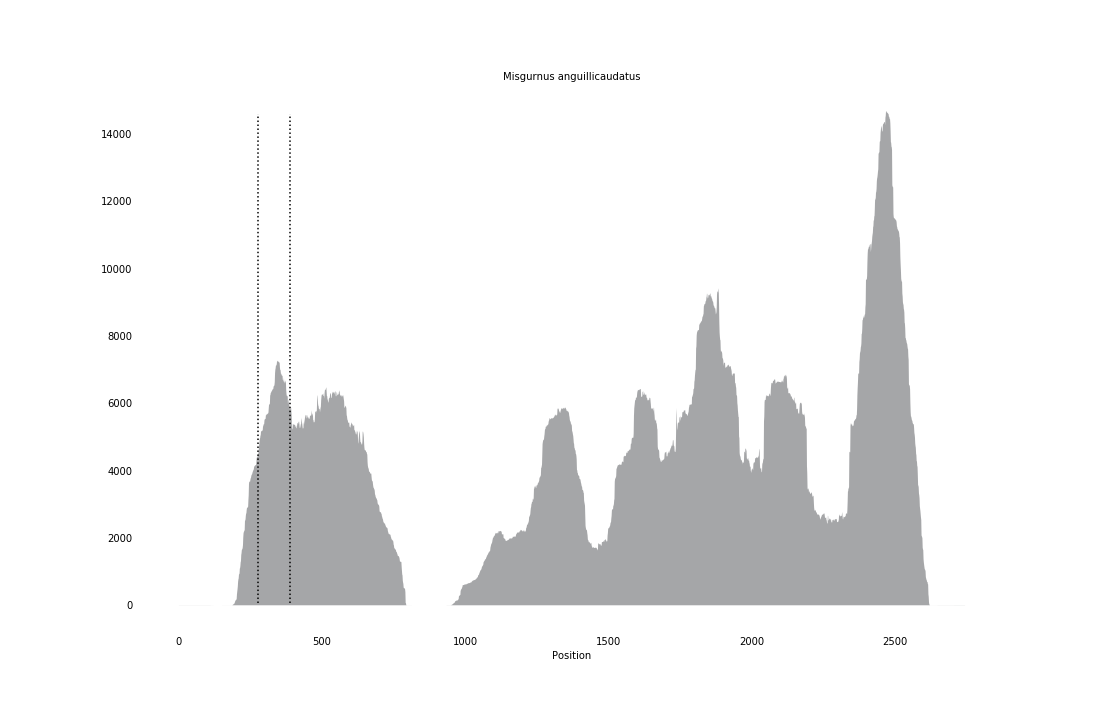

Supplement: Supplementary file 2 — Supplementary information [file 41598_2019_55573_MOESM2_ESM.zip › SupplementaryFile1/Metazoa/Deuterostomia/Misgurnus_anguillicaudatus_coverage_correct.png]

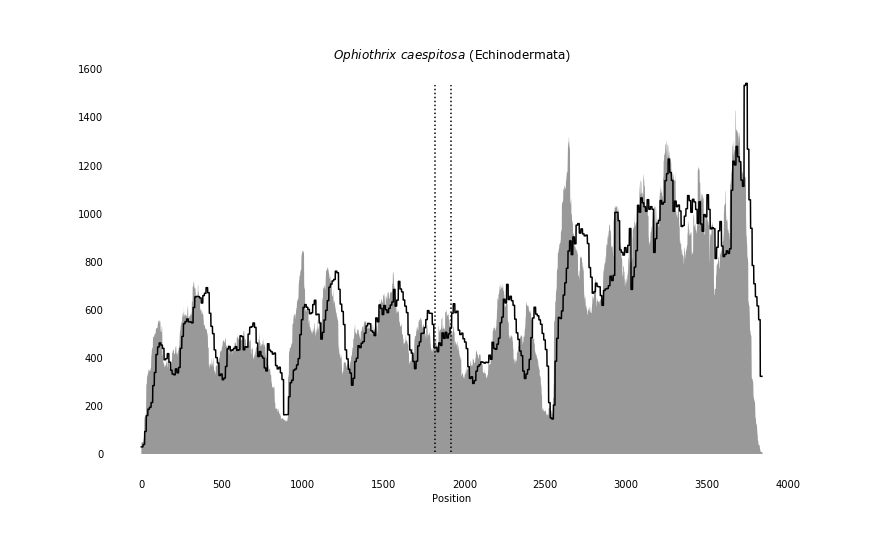

Supplement: Supplementary file 2 — Supplementary information [file 41598_2019_55573_MOESM2_ESM.zip › SupplementaryFile1/Metazoa/Deuterostomia/Ophiothrix_caespitosa_coverage.png]

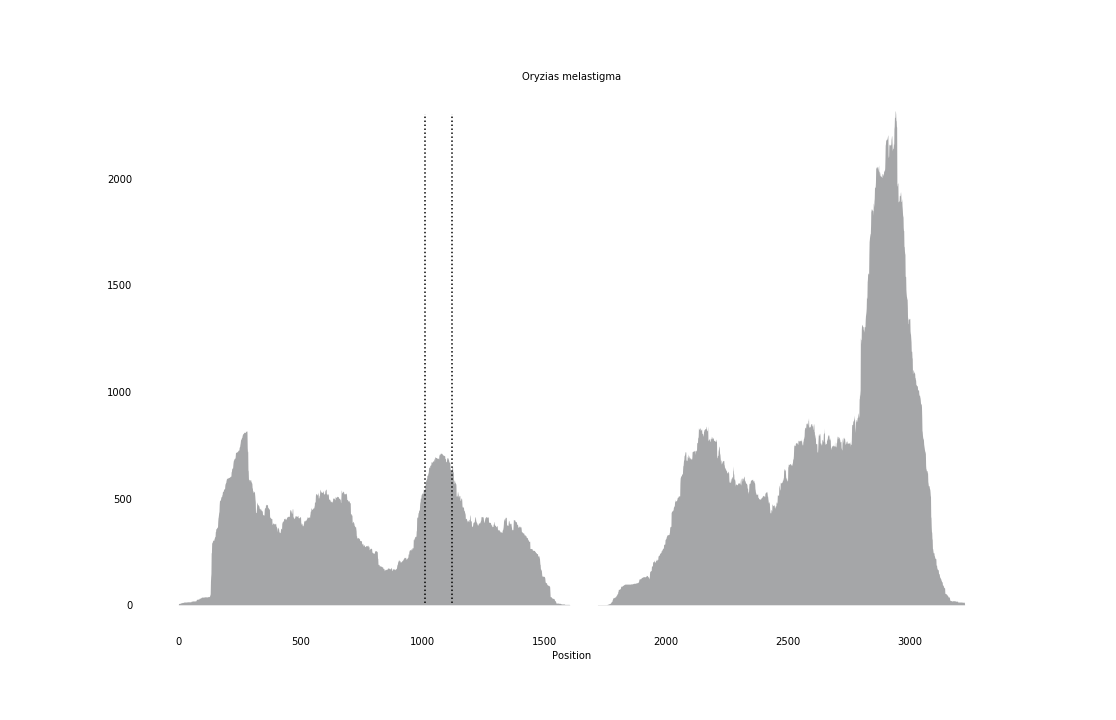

Supplement: Supplementary file 2 — Supplementary information [file 41598_2019_55573_MOESM2_ESM.zip › SupplementaryFile1/Metazoa/Deuterostomia/Oryzias_melastigma_coverage_correct.png]

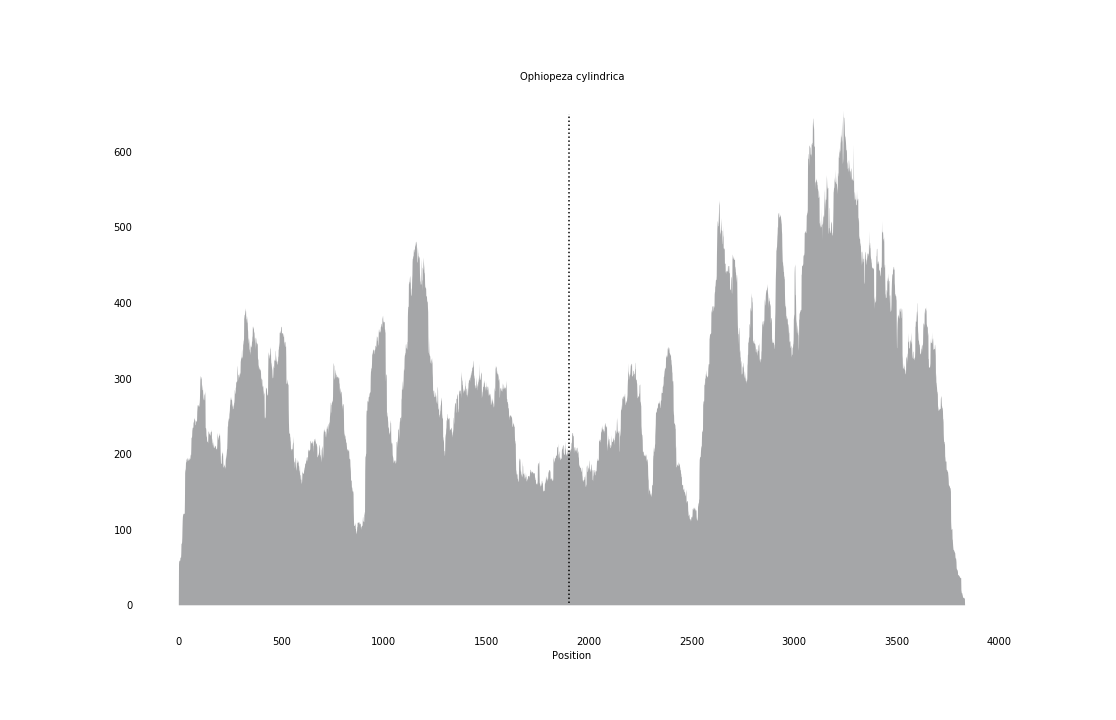

Supplement: Supplementary file 2 — Supplementary information [file 41598_2019_55573_MOESM2_ESM.zip › SupplementaryFile1/Metazoa/Deuterostomia/Ophiopeza_cylindrica_coverage.png]

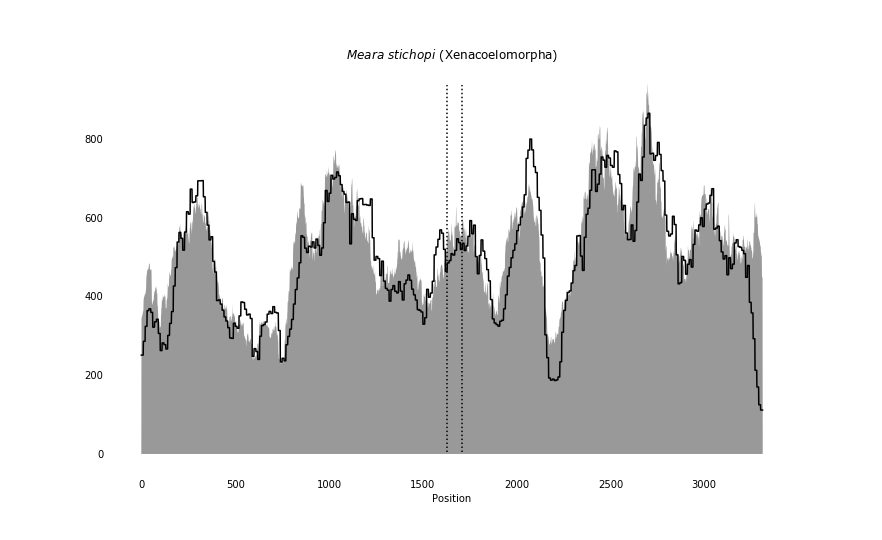

Supplement: Supplementary file 2 — Supplementary information [file 41598_2019_55573_MOESM2_ESM.zip › SupplementaryFile1/Metazoa/Deuterostomia/Meara_stichopi_coverage.png]

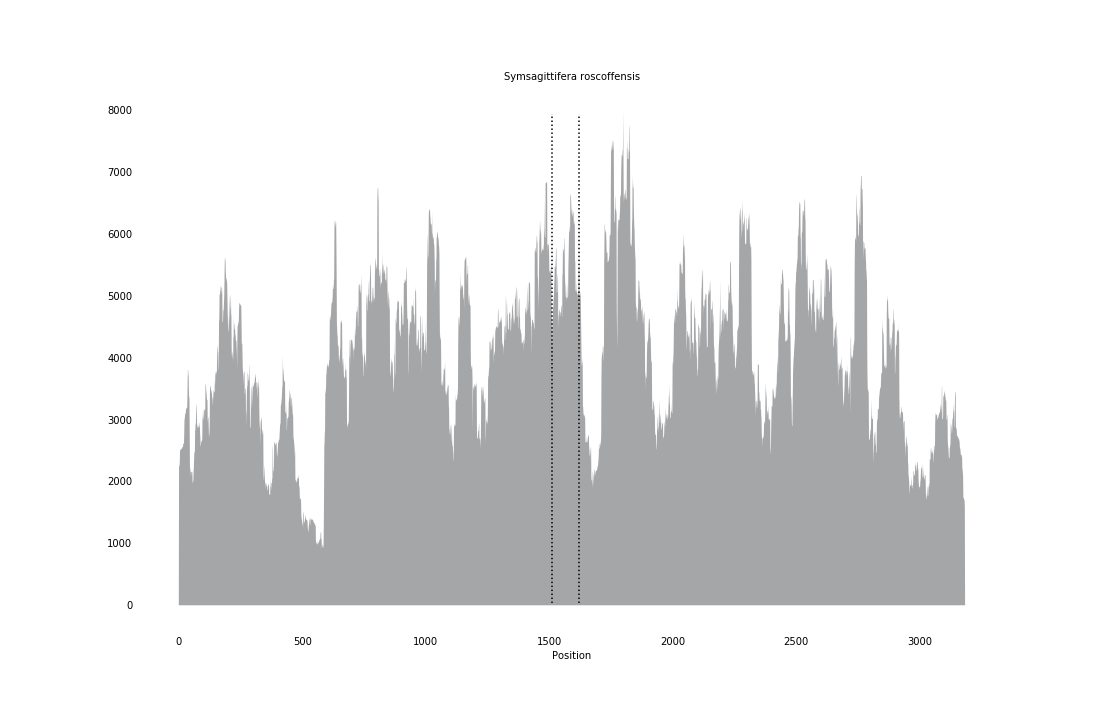

Supplement: Supplementary file 2 — Supplementary information [file 41598_2019_55573_MOESM2_ESM.zip › SupplementaryFile1/Metazoa/Deuterostomia/Symsagittifera_roscoffensis_coverage_correct.png]

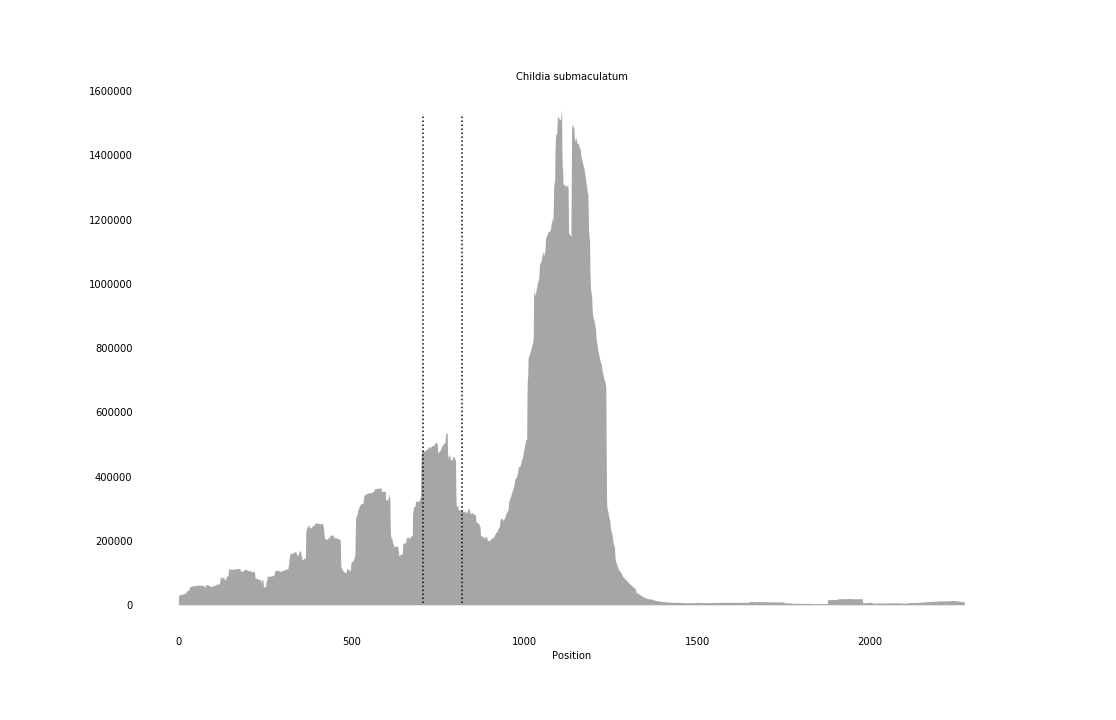

Supplement: Supplementary file 2 — Supplementary information [file 41598_2019_55573_MOESM2_ESM.zip › SupplementaryFile1/Metazoa/Deuterostomia/Childia_submaculatum_coverage_correct.png]

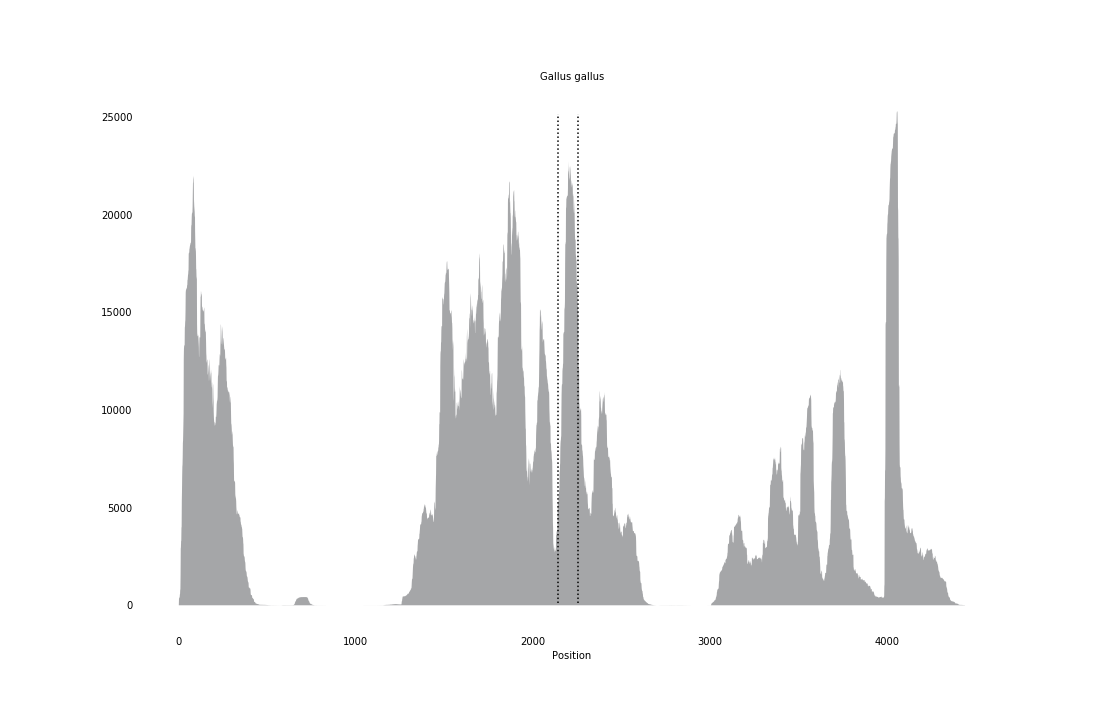

Supplement: Supplementary file 2 — Supplementary information [file 41598_2019_55573_MOESM2_ESM.zip › SupplementaryFile1/Metazoa/Deuterostomia/Gallus_gallus_coverage_correct.png]

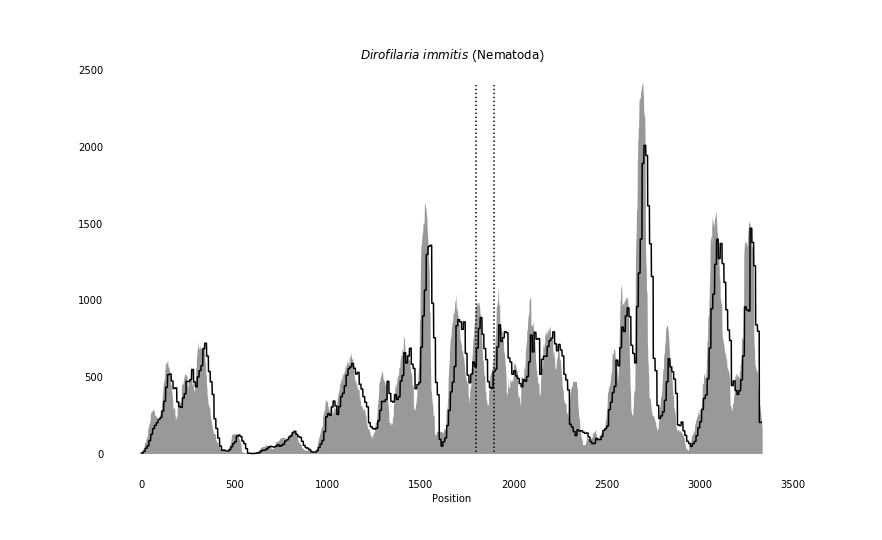

Supplement: Supplementary file 2 — Supplementary information [file 41598_2019_55573_MOESM2_ESM.zip › SupplementaryFile1/Metazoa/Protostomia/Nematoda/Dirofilaria_immitis_coverage.png]

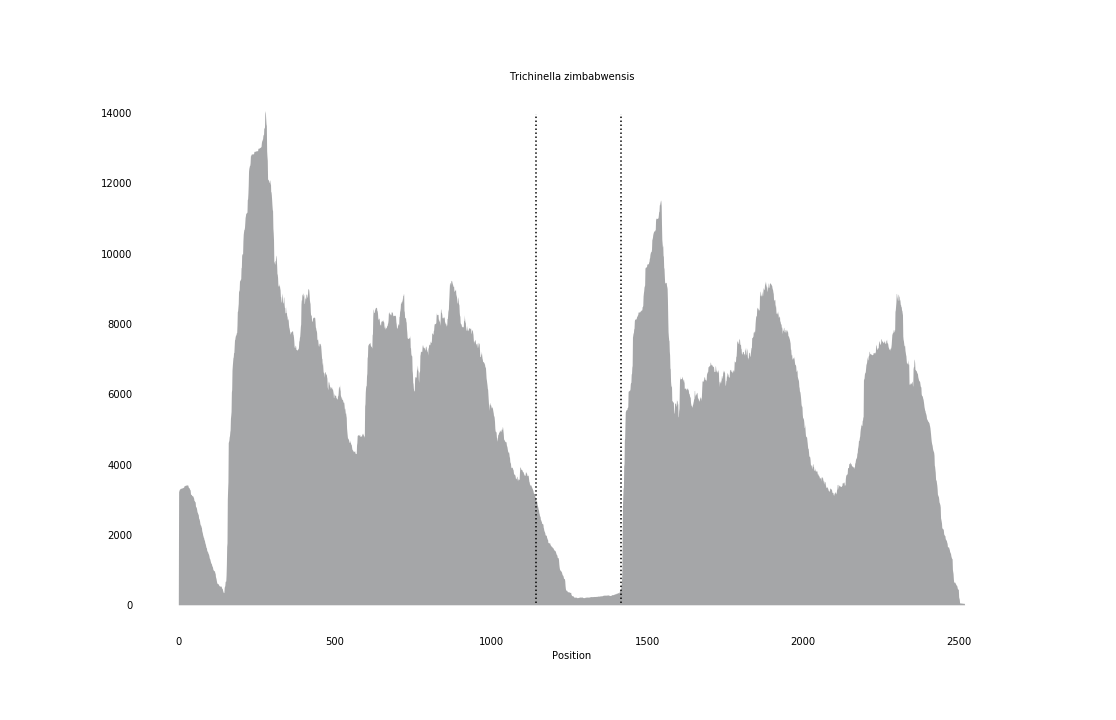

Supplement: Supplementary file 2 — Supplementary information [file 41598_2019_55573_MOESM2_ESM.zip › SupplementaryFile1/Metazoa/Protostomia/Nematoda/Trichinella_zimbabwensis_coverage_correct.png]

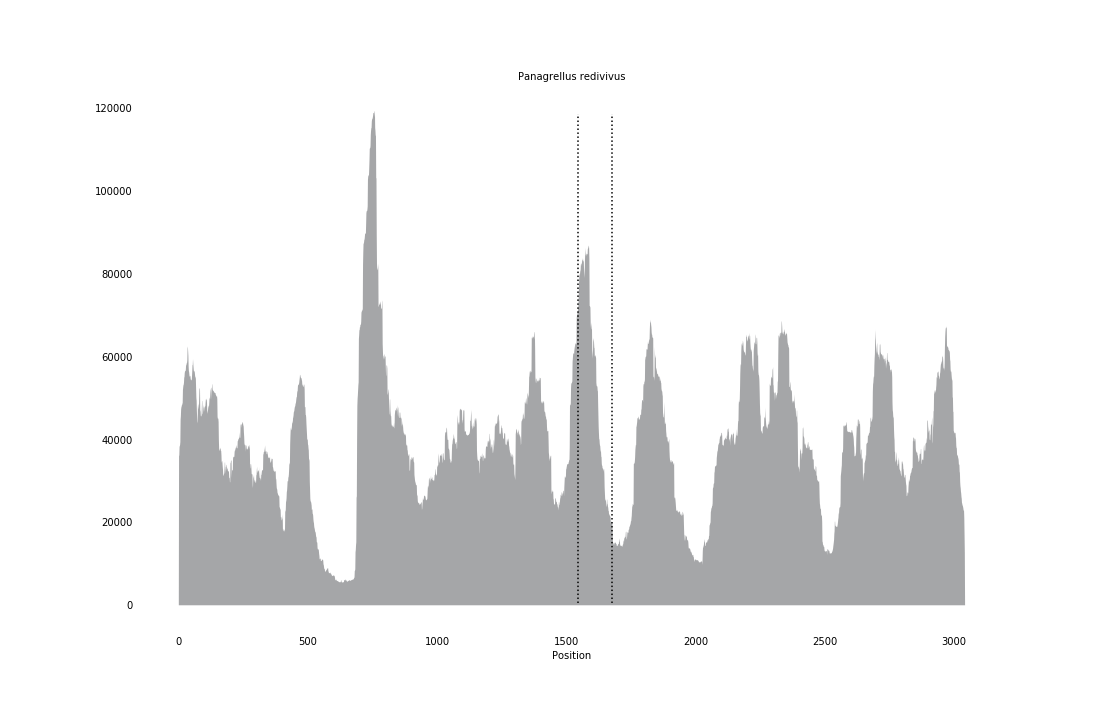

Supplement: Supplementary file 2 — Supplementary information [file 41598_2019_55573_MOESM2_ESM.zip › SupplementaryFile1/Metazoa/Protostomia/Nematoda/Panagrellus_redivivus_coverage_correct.png]

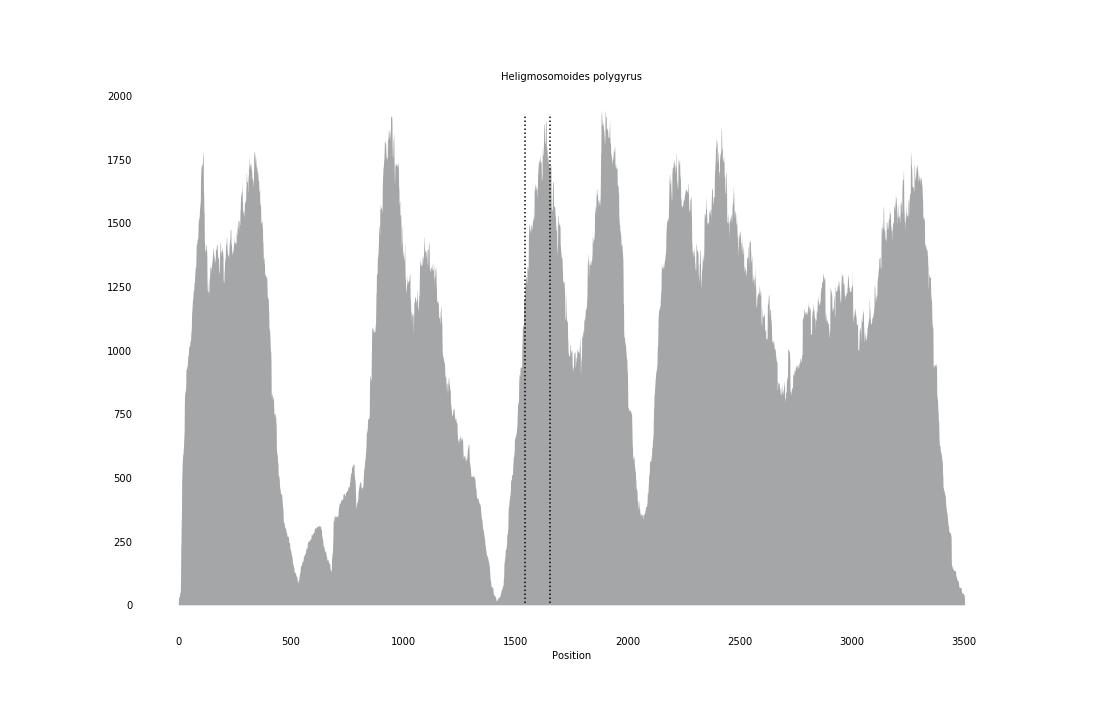

Supplement: Supplementary file 2 — Supplementary information [file 41598_2019_55573_MOESM2_ESM.zip › SupplementaryFile1/Metazoa/Protostomia/Nematoda/Heligmosomoides_polygyrus_coverage_correct.png]

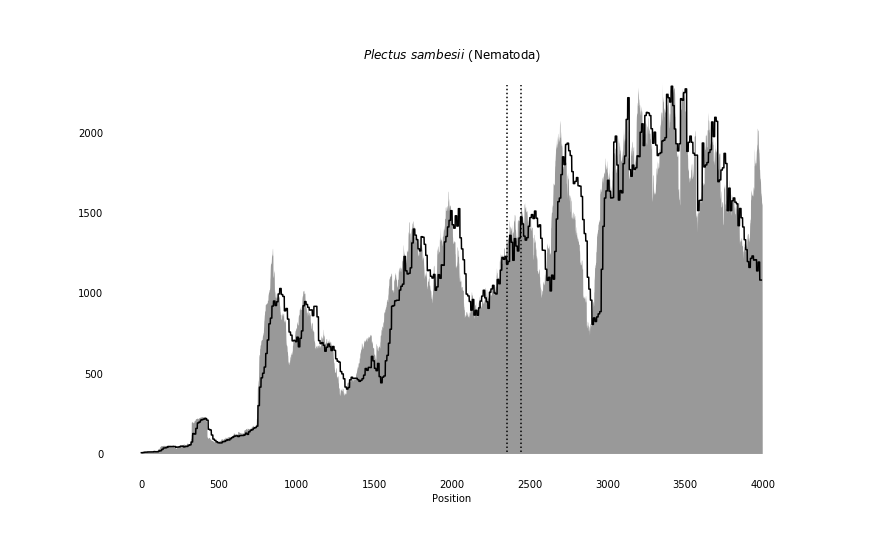

Supplement: Supplementary file 2 — Supplementary information [file 41598_2019_55573_MOESM2_ESM.zip › SupplementaryFile1/Metazoa/Protostomia/Nematoda/Plectus_sambesii_coverage.png]

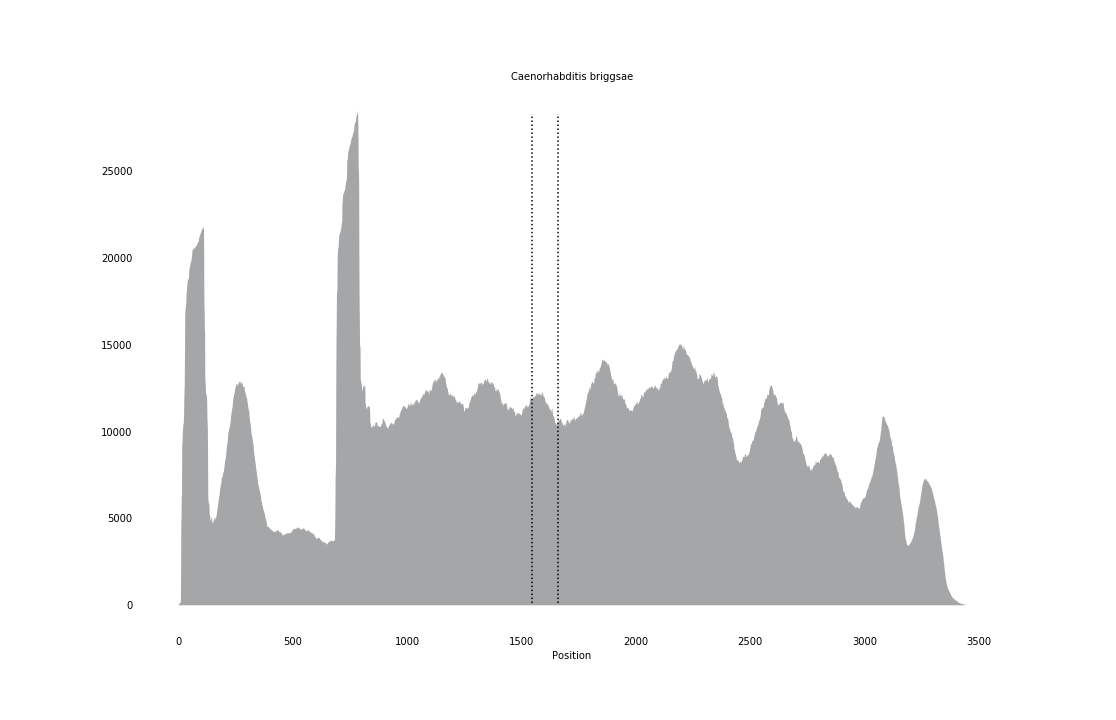

Supplement: Supplementary file 2 — Supplementary information [file 41598_2019_55573_MOESM2_ESM.zip › SupplementaryFile1/Metazoa/Protostomia/Nematoda/Caenorhabditis_briggsae_coverage_correct.png]

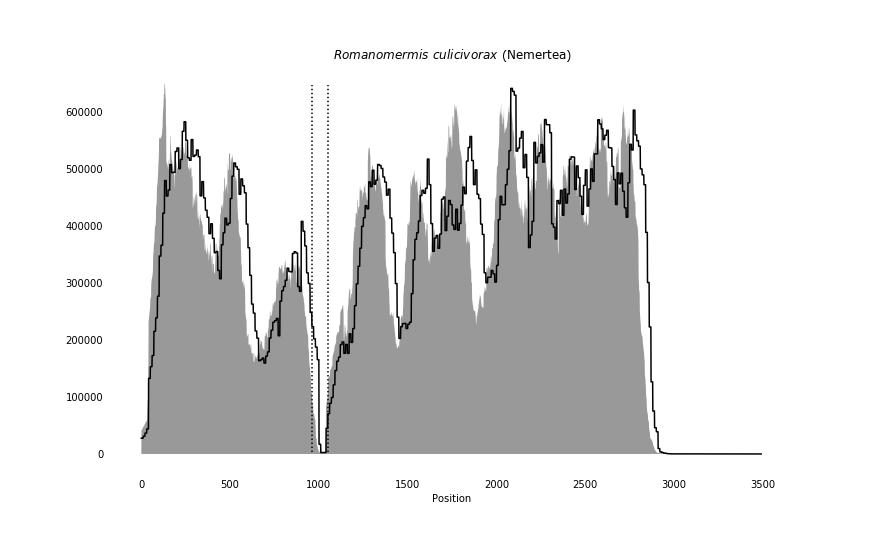

Supplement: Supplementary file 2 — Supplementary information [file 41598_2019_55573_MOESM2_ESM.zip › SupplementaryFile1/Metazoa/Protostomia/Nematoda/Romanomermis_culicivorax_coverage.png]

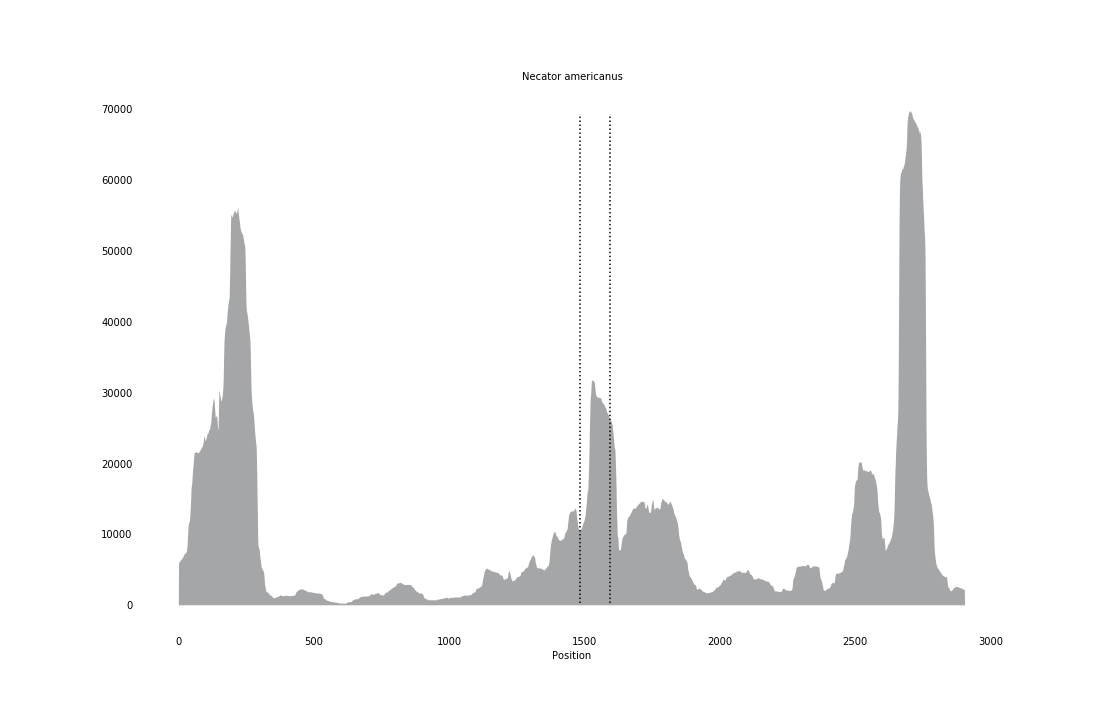

Supplement: Supplementary file 2 — Supplementary information [file 41598_2019_55573_MOESM2_ESM.zip › SupplementaryFile1/Metazoa/Protostomia/Nematoda/Necator_americanus_coverage_correct.png]

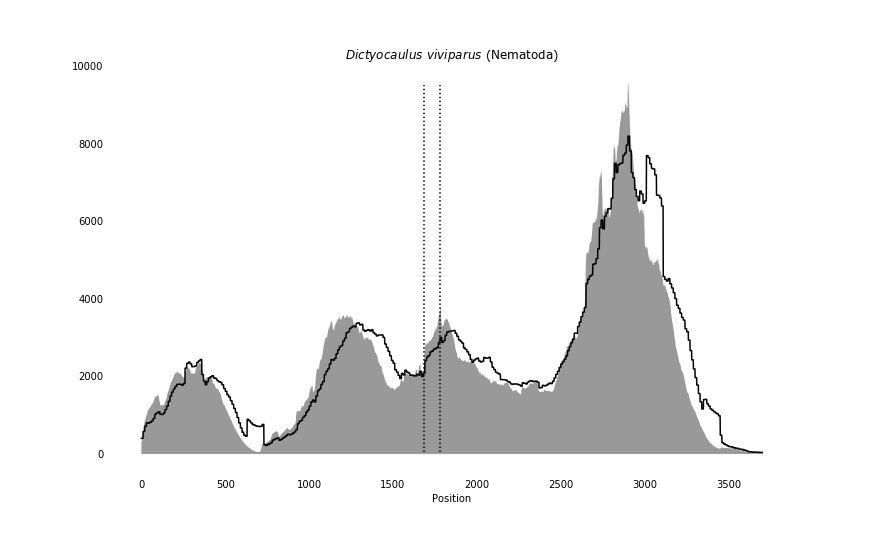

Supplement: Supplementary file 2 — Supplementary information [file 41598_2019_55573_MOESM2_ESM.zip › SupplementaryFile1/Metazoa/Protostomia/Nematoda/Dictyocaulus_viviparus_coverage.png]

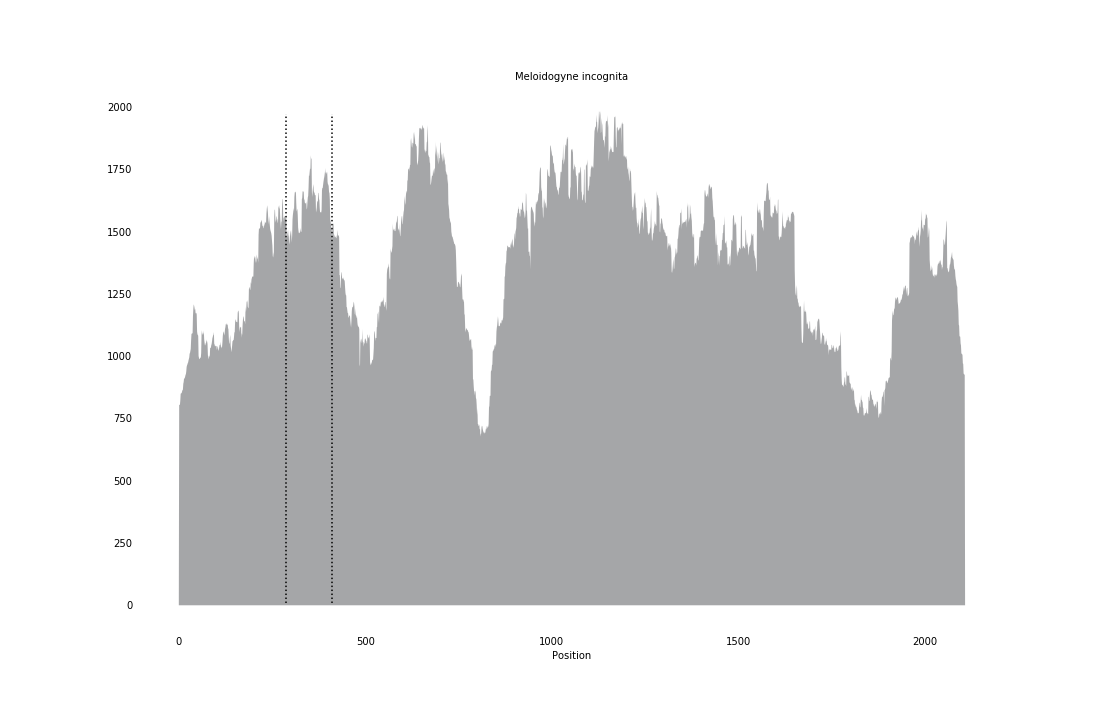

Supplement: Supplementary file 2 — Supplementary information [file 41598_2019_55573_MOESM2_ESM.zip › SupplementaryFile1/Metazoa/Protostomia/Nematoda/Meloidogyne_incognita_coverage_correct.png]

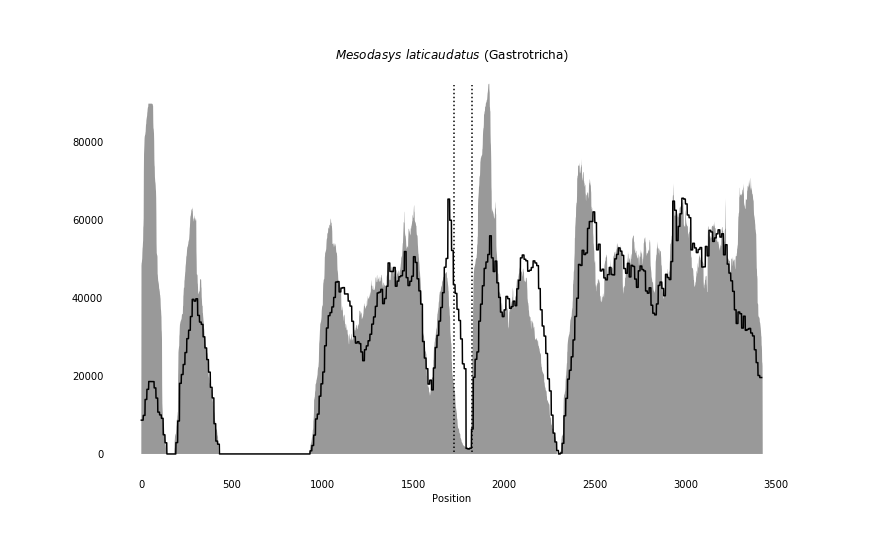

Supplement: Supplementary file 2 — Supplementary information [file 41598_2019_55573_MOESM2_ESM.zip › SupplementaryFile1/Metazoa/Protostomia/Mesodasys_laticaudatus_coverage.png]

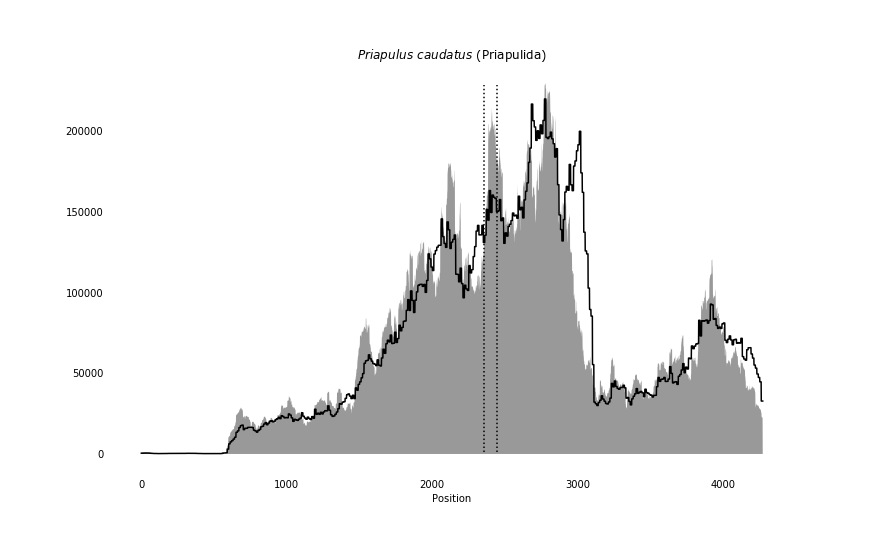

Supplement: Supplementary file 2 — Supplementary information [file 41598_2019_55573_MOESM2_ESM.zip › SupplementaryFile1/Metazoa/Protostomia/Priapulus_caudatus_coverage.png]

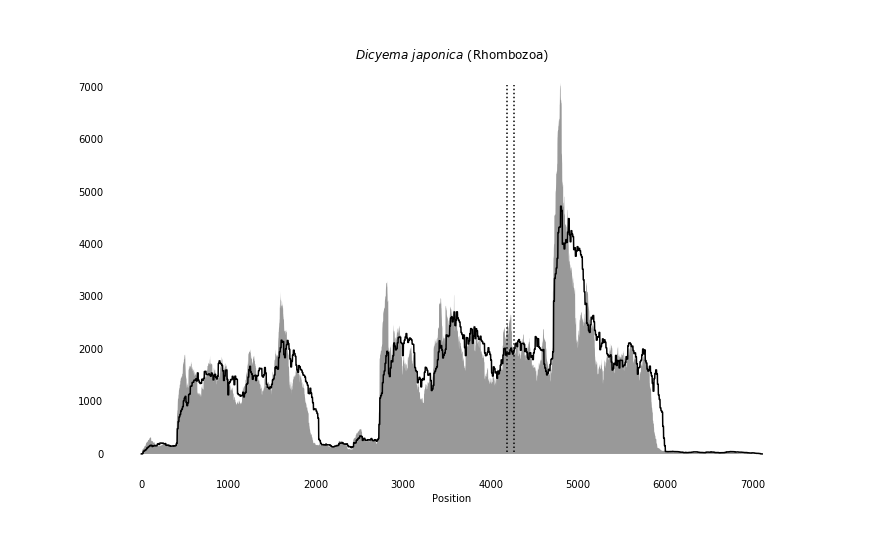

Supplement: Supplementary file 2 — Supplementary information [file 41598_2019_55573_MOESM2_ESM.zip › SupplementaryFile1/Metazoa/Protostomia/Dicyema_japonica_coverage.png]

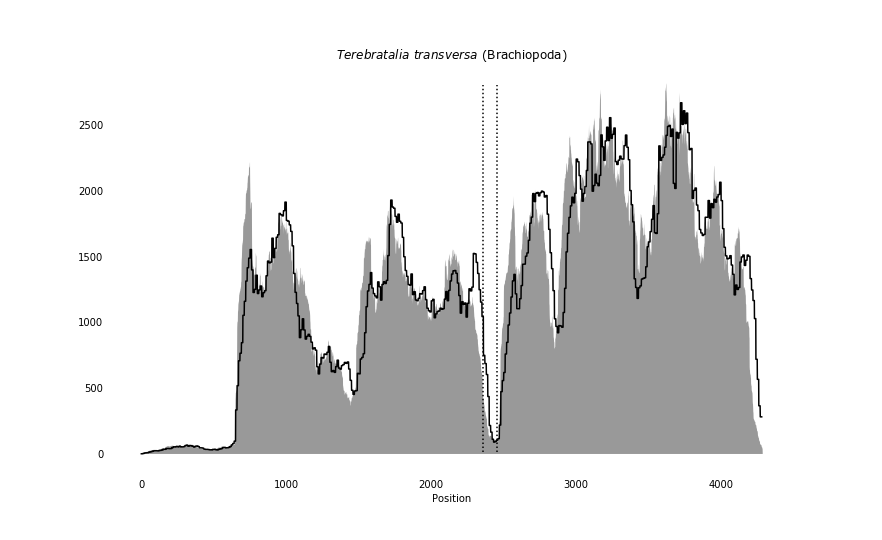

Supplement: Supplementary file 2 — Supplementary information [file 41598_2019_55573_MOESM2_ESM.zip › SupplementaryFile1/Metazoa/Protostomia/Terebratalia_transversa_coverage.png]

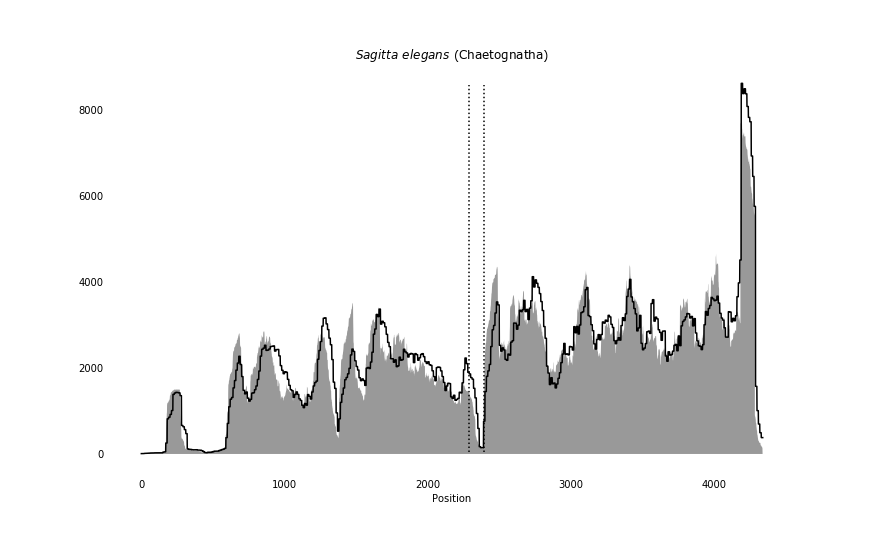

Supplement: Supplementary file 2 — Supplementary information [file 41598_2019_55573_MOESM2_ESM.zip › SupplementaryFile1/Metazoa/Protostomia/Sagitta_elegans_coverage.png]

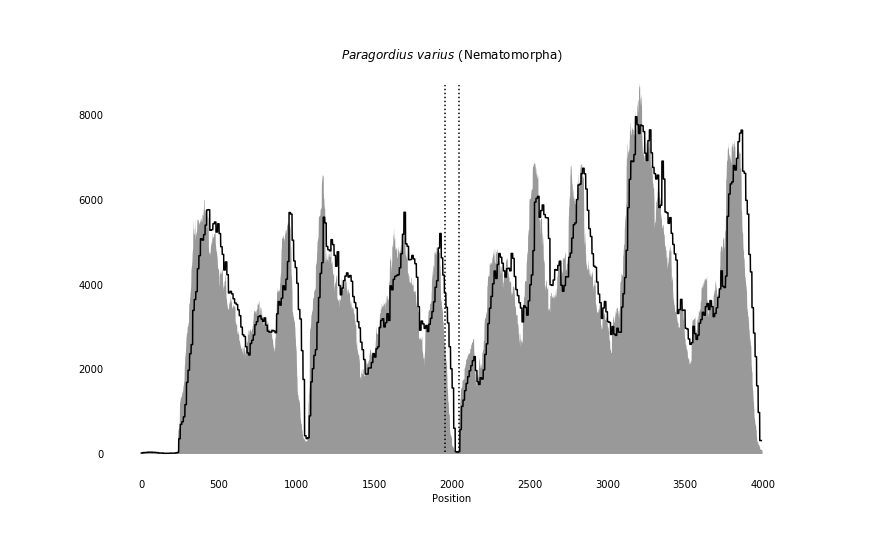

Supplement: Supplementary file 2 — Supplementary information [file 41598_2019_55573_MOESM2_ESM.zip › SupplementaryFile1/Metazoa/Protostomia/Paragordius_varius_coverage.png]

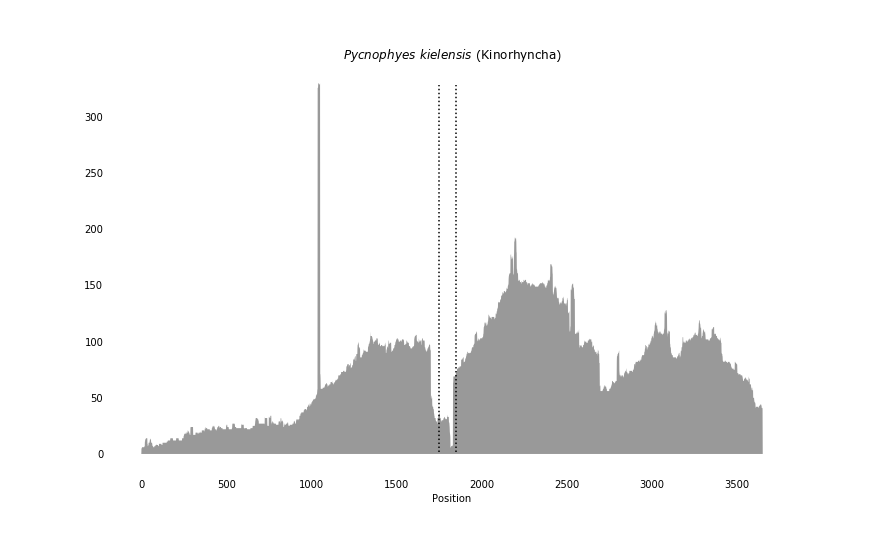

Supplement: Supplementary file 2 — Supplementary information [file 41598_2019_55573_MOESM2_ESM.zip › SupplementaryFile1/Metazoa/Protostomia/Pycnophyes_kielensis_coverage.png]

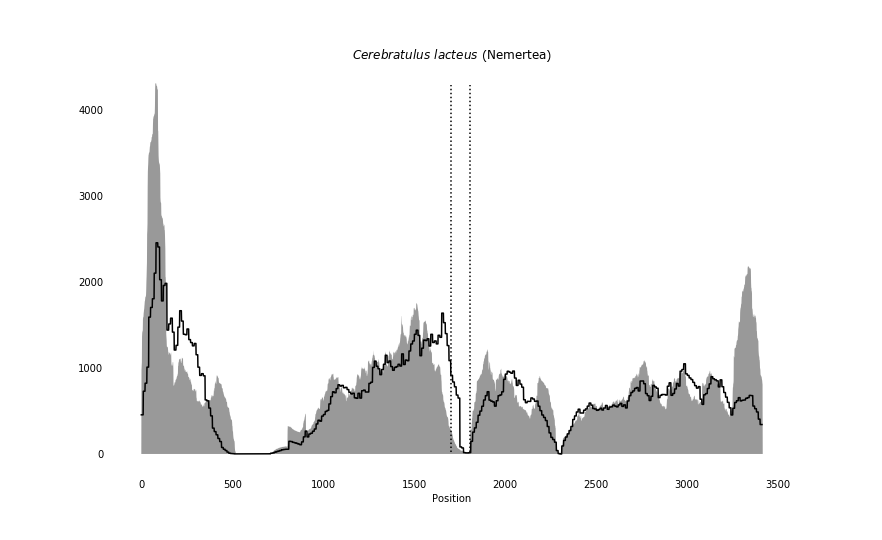

Supplement: Supplementary file 2 — Supplementary information [file 41598_2019_55573_MOESM2_ESM.zip › SupplementaryFile1/Metazoa/Protostomia/Cerebratulus_lacteus_coverage.png]

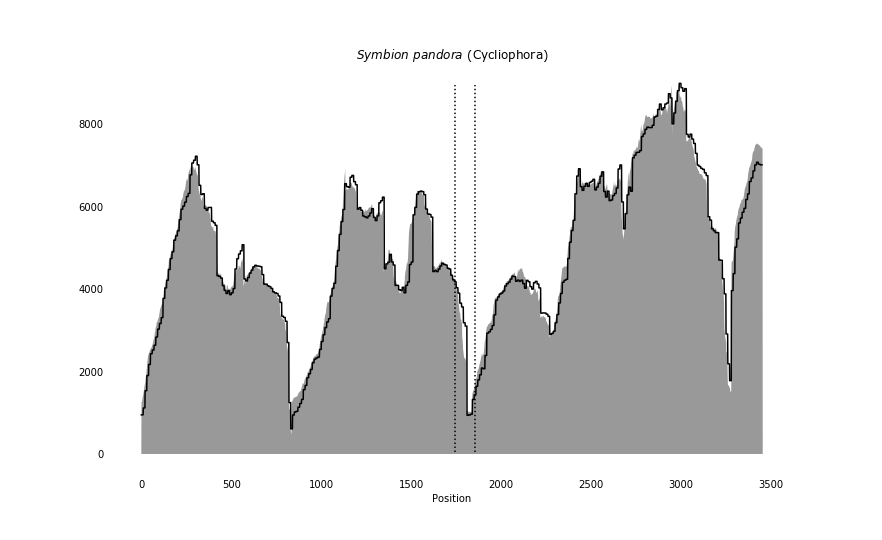

Supplement: Supplementary file 2 — Supplementary information [file 41598_2019_55573_MOESM2_ESM.zip › SupplementaryFile1/Metazoa/Protostomia/Symbion_pandora_coverage.png]

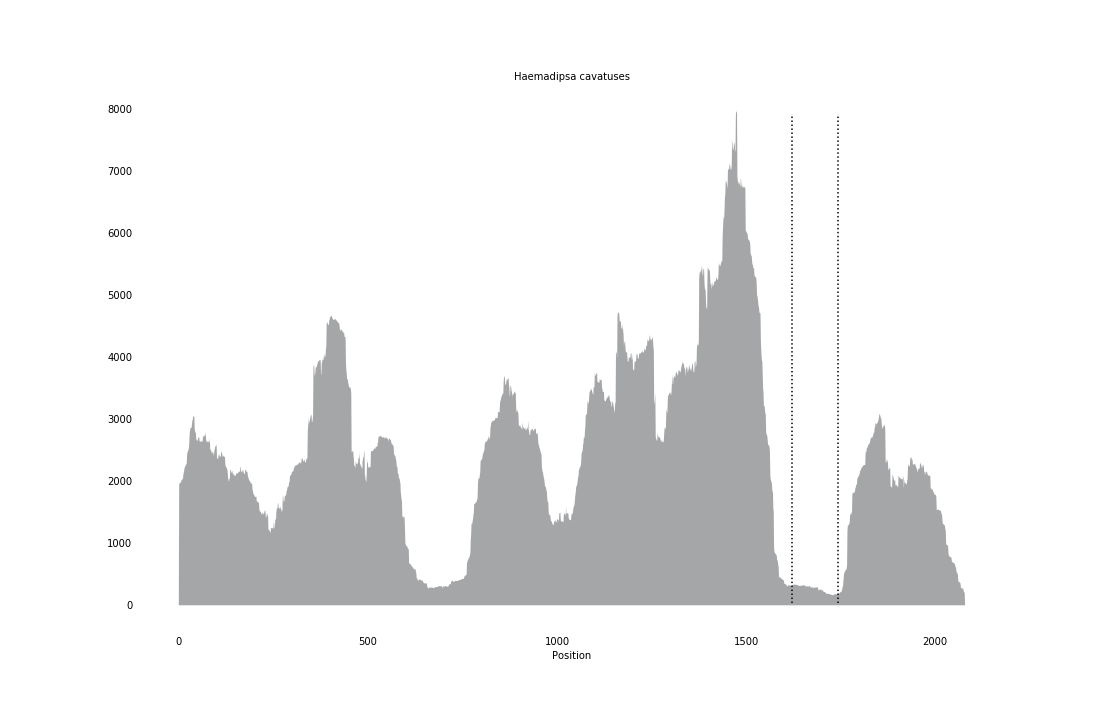

Supplement: Supplementary file 2 — Supplementary information [file 41598_2019_55573_MOESM2_ESM.zip › SupplementaryFile1/Metazoa/Protostomia/Annelida/Haemadipsa_cavatuses_coverage_correct.png]

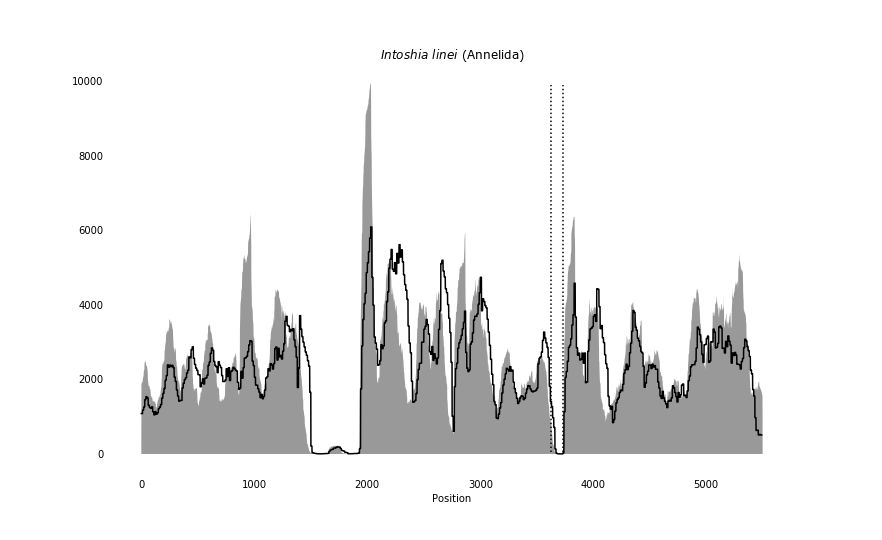

Supplement: Supplementary file 2 — Supplementary information [file 41598_2019_55573_MOESM2_ESM.zip › SupplementaryFile1/Metazoa/Protostomia/Annelida/Intoshia_linei_coverage.png]

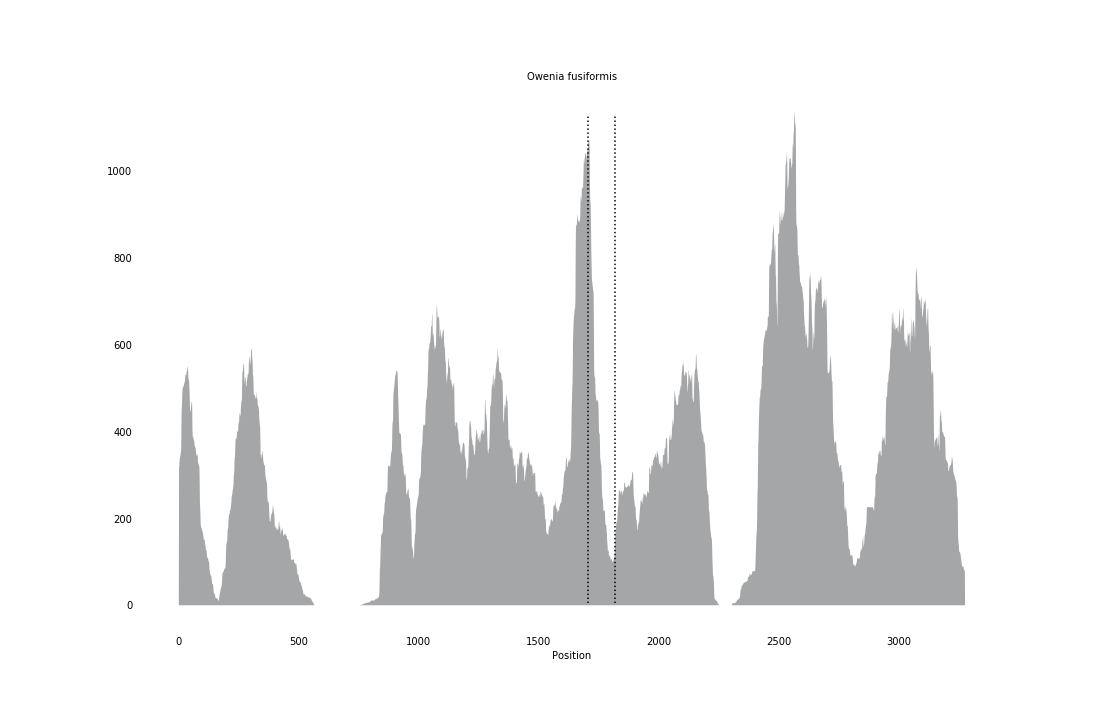

Supplement: Supplementary file 2 — Supplementary information [file 41598_2019_55573_MOESM2_ESM.zip › SupplementaryFile1/Metazoa/Protostomia/Annelida/Owenia_fusiformis_coverage_correct.png]

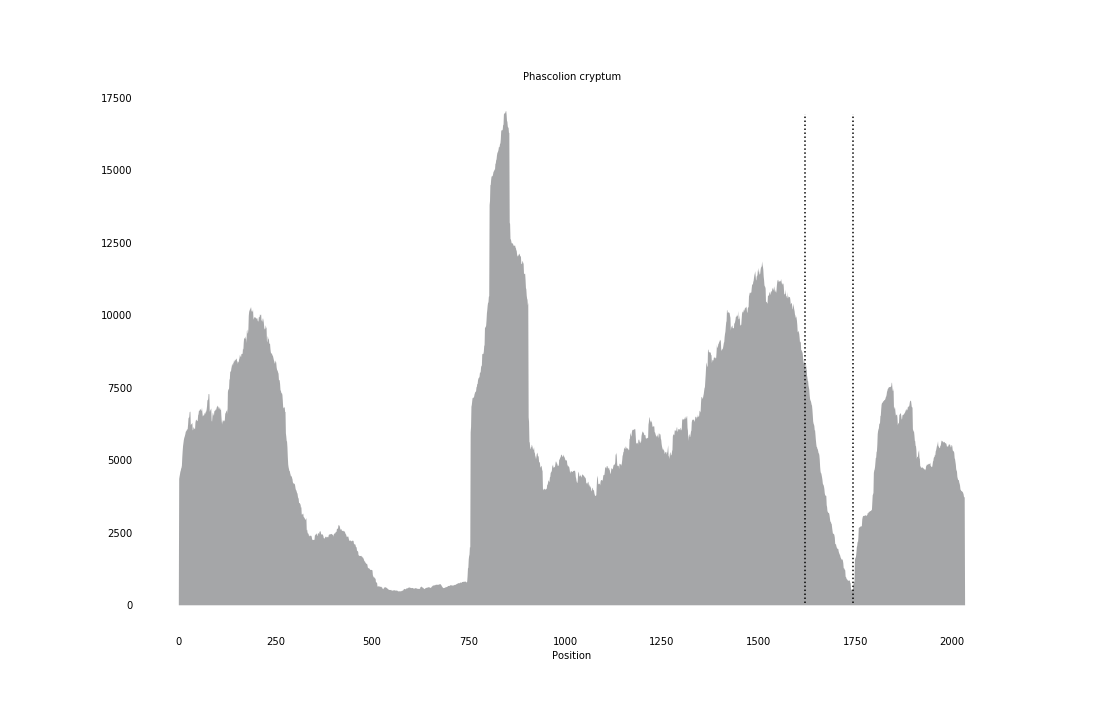

Supplement: Supplementary file 2 — Supplementary information [file 41598_2019_55573_MOESM2_ESM.zip › SupplementaryFile1/Metazoa/Protostomia/Annelida/Phascolion_cryptum_coverage_correct.png]

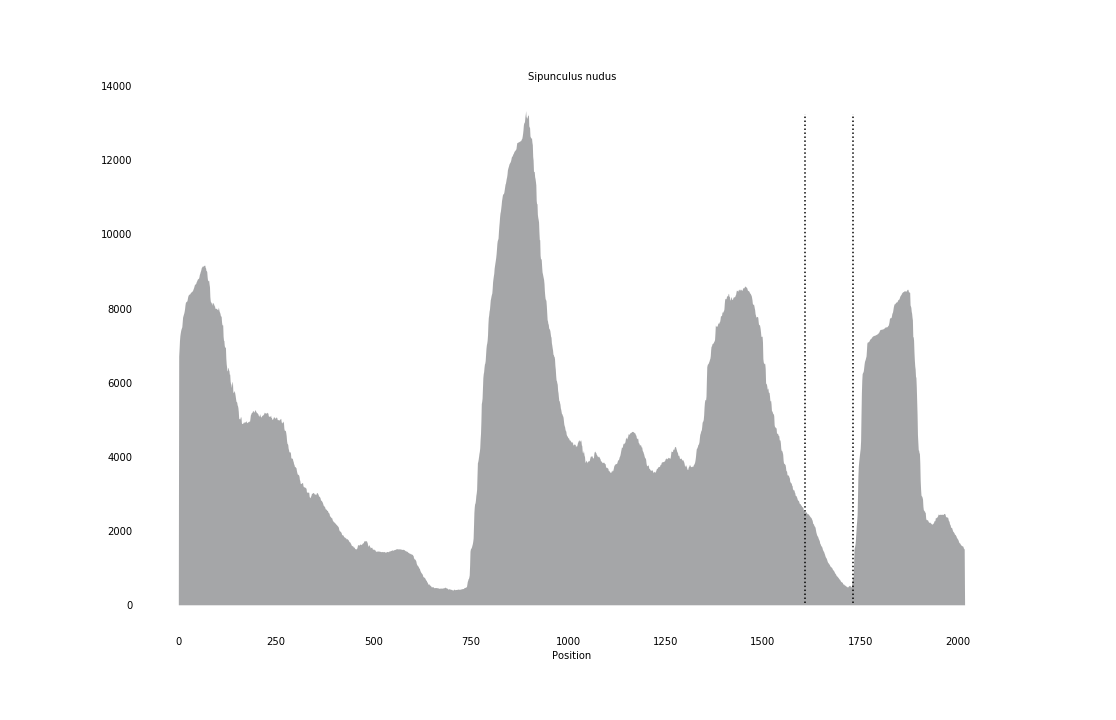

Supplement: Supplementary file 2 — Supplementary information [file 41598_2019_55573_MOESM2_ESM.zip › SupplementaryFile1/Metazoa/Protostomia/Annelida/Sipunculus_nudus_coverage_correct.png]

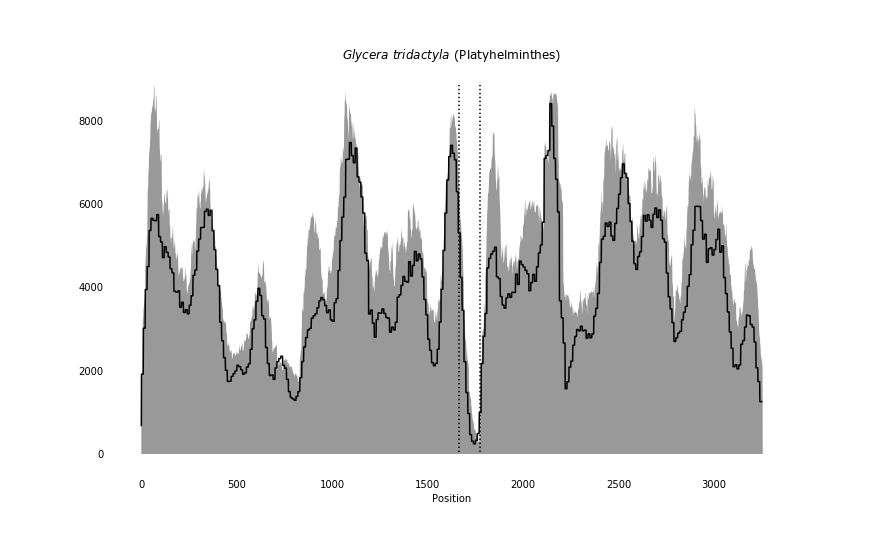

Supplement: Supplementary file 2 — Supplementary information [file 41598_2019_55573_MOESM2_ESM.zip › SupplementaryFile1/Metazoa/Protostomia/Annelida/Glycera_tridactyla_coverage.png]

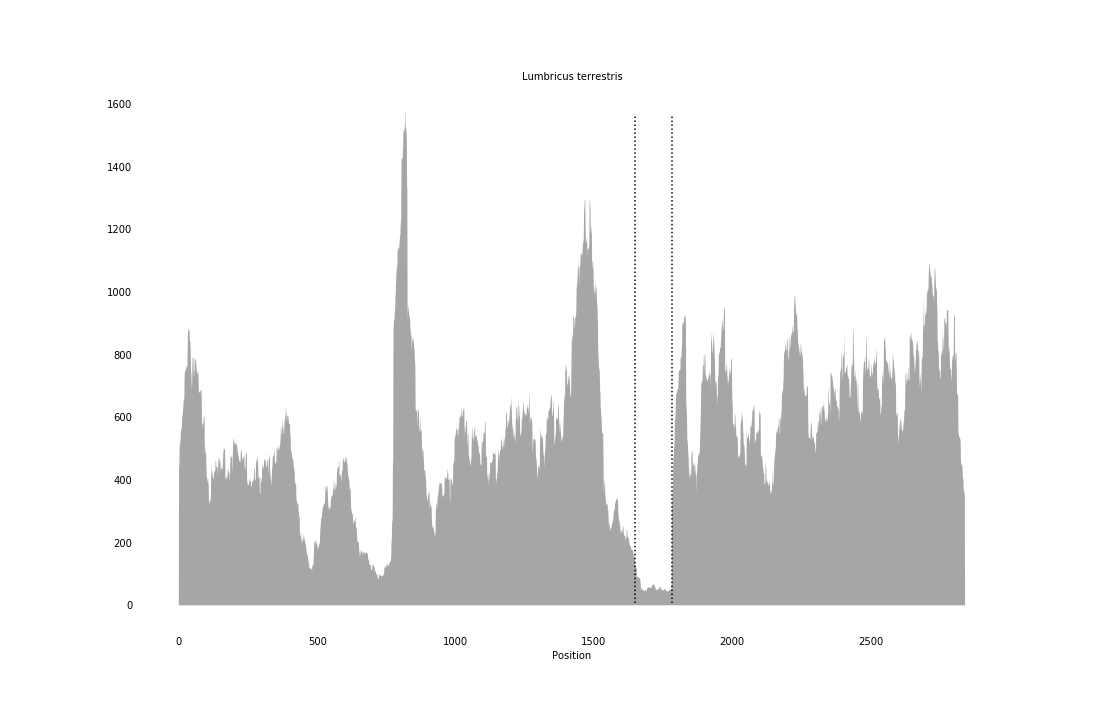

Supplement: Supplementary file 2 — Supplementary information [file 41598_2019_55573_MOESM2_ESM.zip › SupplementaryFile1/Metazoa/Protostomia/Annelida/Lumbricus_terrestris_coverage_correct.png]

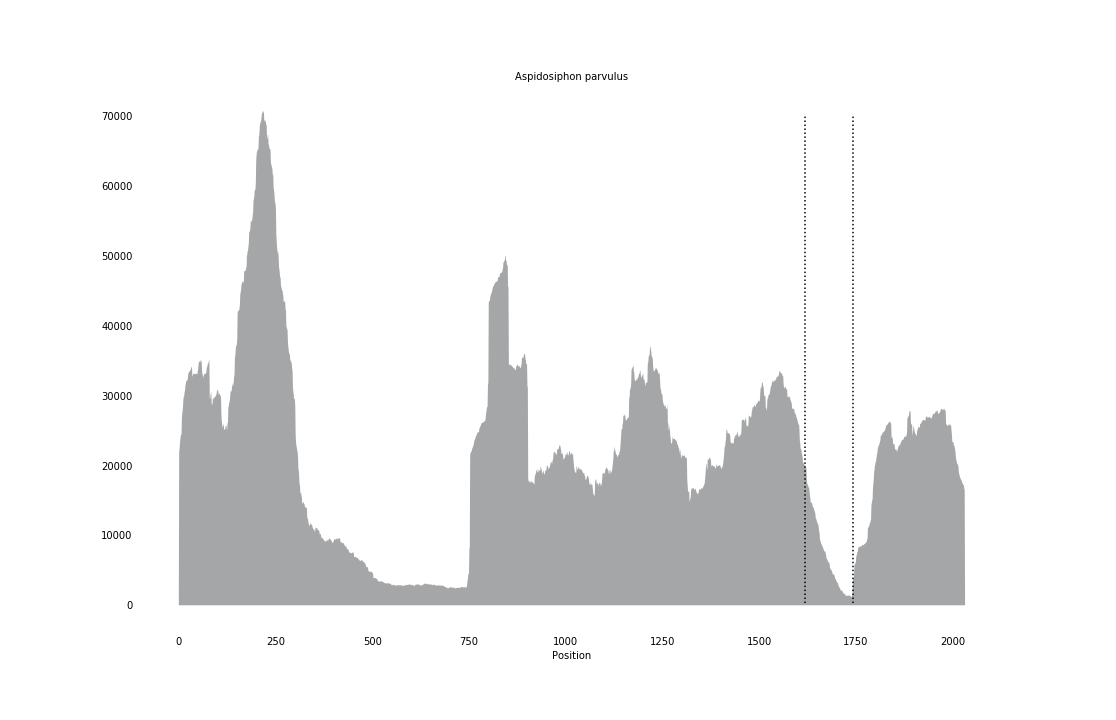

Supplement: Supplementary file 2 — Supplementary information [file 41598_2019_55573_MOESM2_ESM.zip › SupplementaryFile1/Metazoa/Protostomia/Annelida/Aspidosiphon_parvulus_coverage_correct.png]

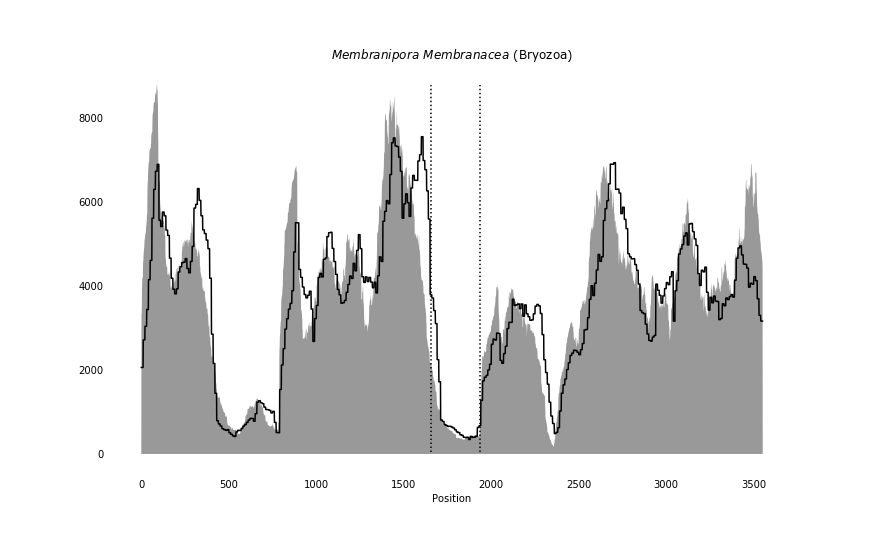

Supplement: Supplementary file 2 — Supplementary information [file 41598_2019_55573_MOESM2_ESM.zip › SupplementaryFile1/Metazoa/Protostomia/Membranipora_membranacea_coverage.png]

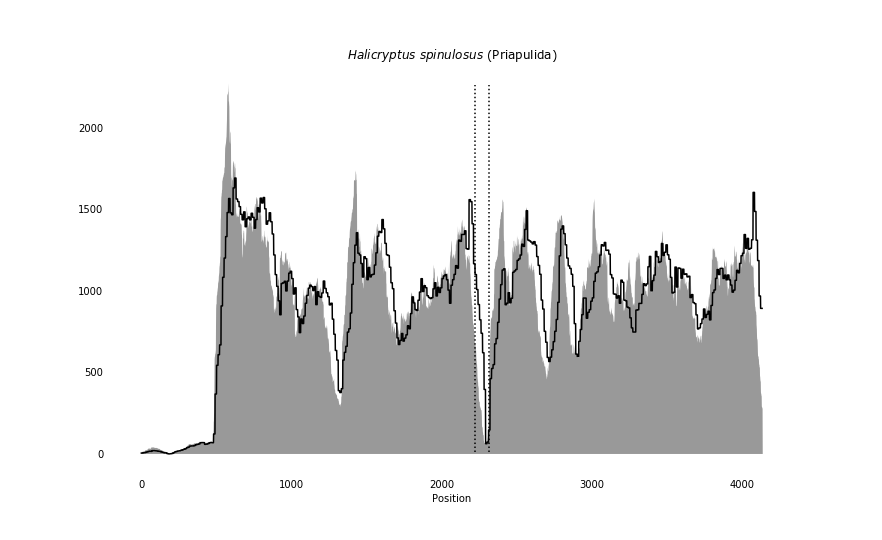

Supplement: Supplementary file 2 — Supplementary information [file 41598_2019_55573_MOESM2_ESM.zip › SupplementaryFile1/Metazoa/Protostomia/Halicryptus_spinulosus_coverage.png]

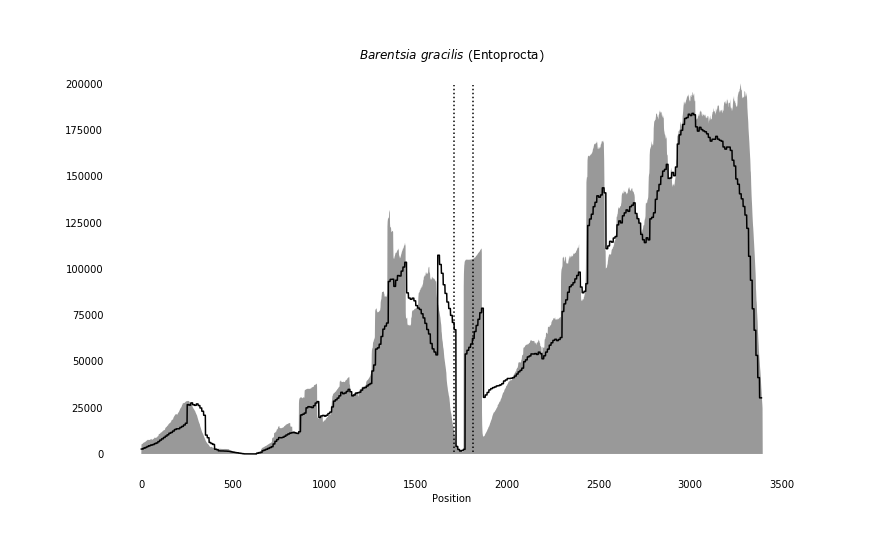

Supplement: Supplementary file 2 — Supplementary information [file 41598_2019_55573_MOESM2_ESM.zip › SupplementaryFile1/Metazoa/Protostomia/Barentsia_gracilis_overage.png]

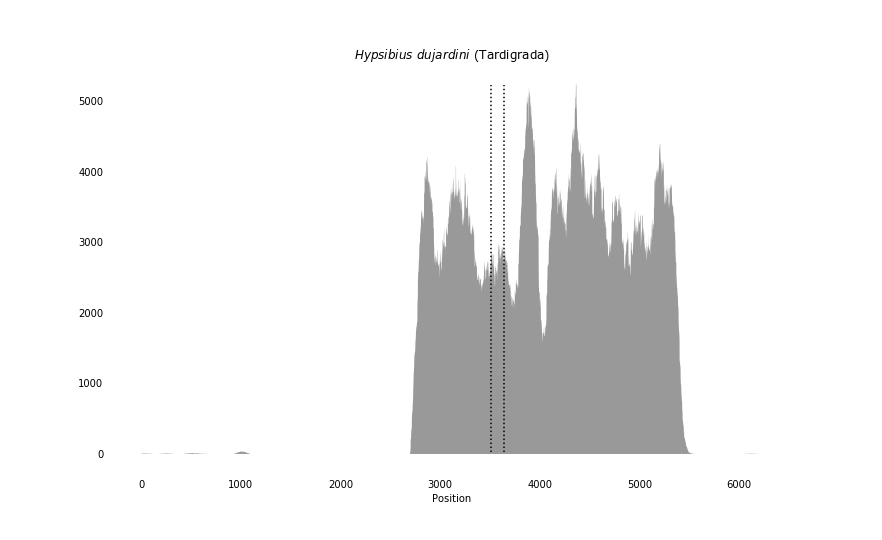

Supplement: Supplementary file 2 — Supplementary information [file 41598_2019_55573_MOESM2_ESM.zip › SupplementaryFile1/Metazoa/Protostomia/Hypsibius_dujardini_coverage.png]
