# Supplementary figures and images for: Computational discovery of hidden breaks in 28S ribosomal RNAs across eukaryotes and consequences for RNA Integrity Numbers (part 3 of 3)
Source: Sci Rep. 2019 Dec 20;9:19477. doi: 10.1038/s41598-019-55573-1 (PMC6925239; doi:10.1038/s41598-019-55573-1)

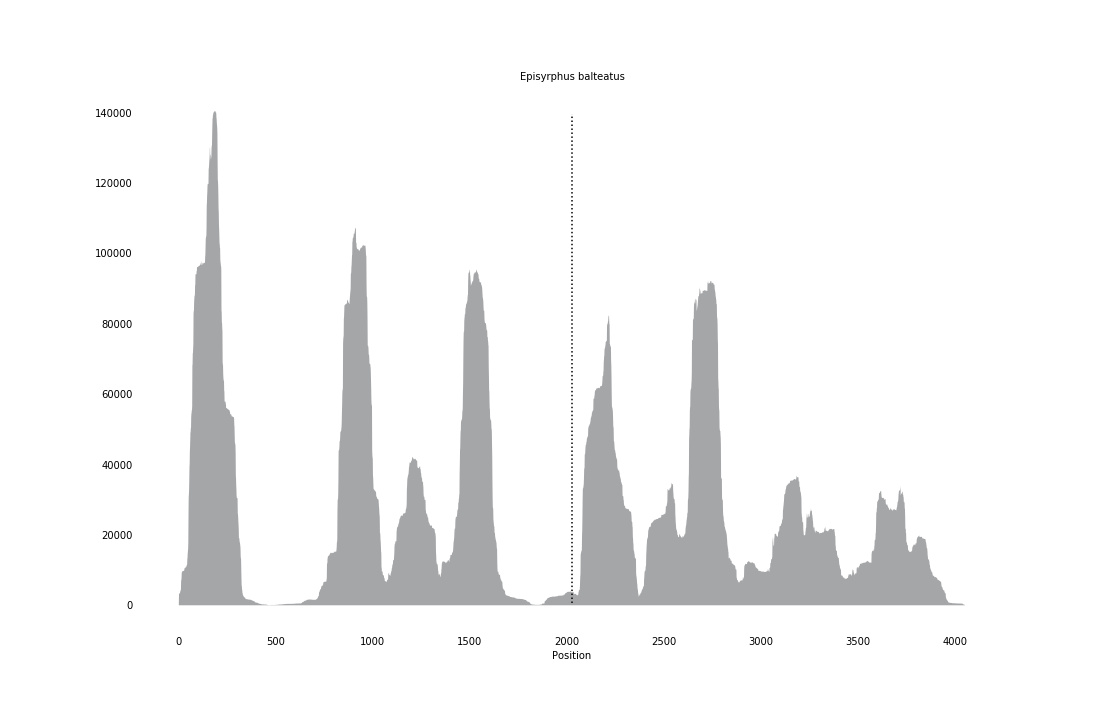

Supplement: Supplementary file 2 — Supplementary information [file 41598_2019_55573_MOESM2_ESM.zip › SupplementaryFile1/Metazoa/Protostomia/Arthropoda/Insecta/Episyrphus_balteatus_coverage.png]

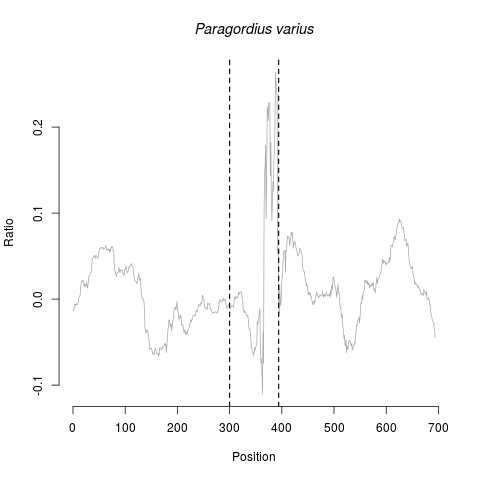

Supplement: Supplementary file 3 — Supplementary information [file 41598_2019_55573_MOESM3_ESM.zip › SupplementaryFile2/WITH_BREAK/paragordius_log_rf.png]

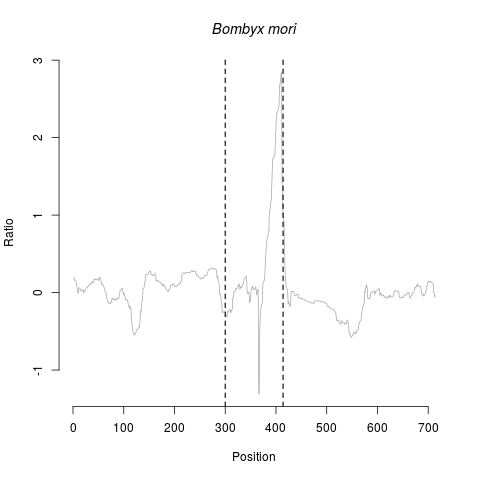

Supplement: Supplementary file 3 — Supplementary information [file 41598_2019_55573_MOESM3_ESM.zip › SupplementaryFile2/WITH_BREAK/bombyx_log_fr.png]

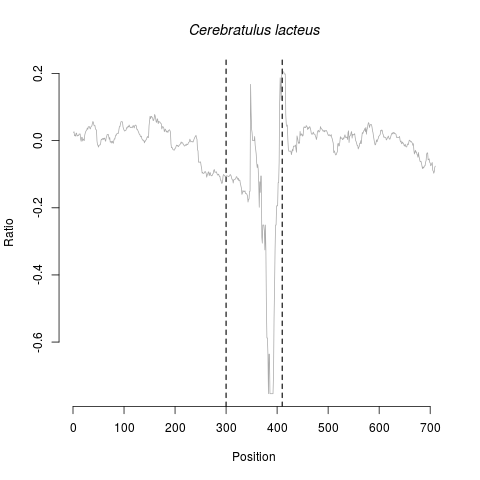

Supplement: Supplementary file 3 — Supplementary information [file 41598_2019_55573_MOESM3_ESM.zip › SupplementaryFile2/WITH_BREAK/cerebratulus_log_rf.png]

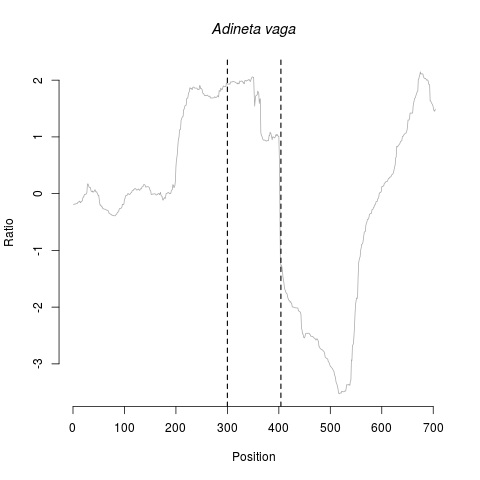

Supplement: Supplementary file 3 — Supplementary information [file 41598_2019_55573_MOESM3_ESM.zip › SupplementaryFile2/WITH_BREAK/adineta_log_fr.png]

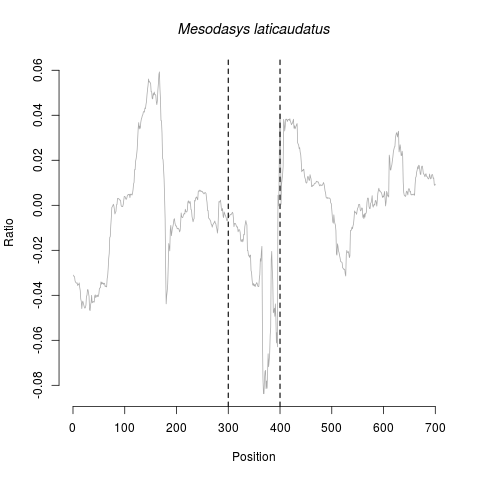

Supplement: Supplementary file 3 — Supplementary information [file 41598_2019_55573_MOESM3_ESM.zip › SupplementaryFile2/WITH_BREAK/mesodasys_log_fr.png]

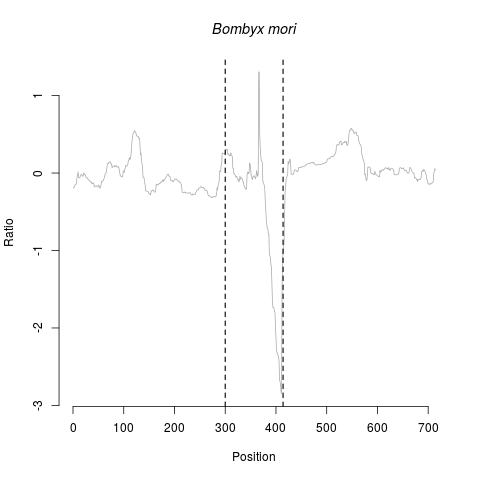

Supplement: Supplementary file 3 — Supplementary information [file 41598_2019_55573_MOESM3_ESM.zip › SupplementaryFile2/WITH_BREAK/bombyx_log_rf.png]

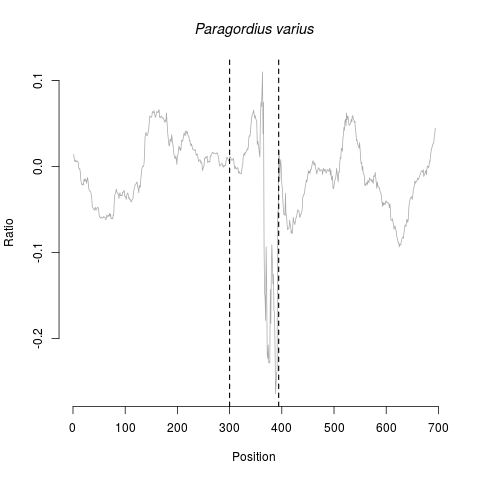

Supplement: Supplementary file 3 — Supplementary information [file 41598_2019_55573_MOESM3_ESM.zip › SupplementaryFile2/WITH_BREAK/paragordius_log_fr.png]

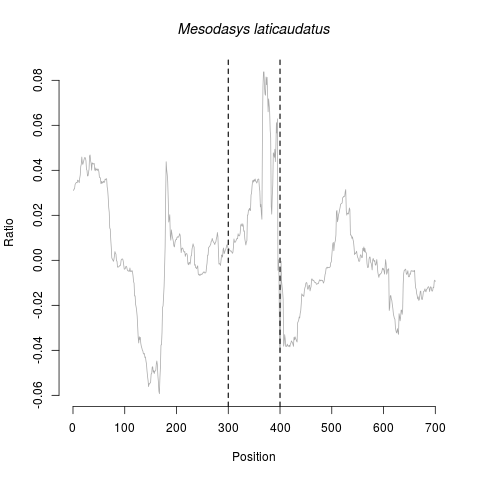

Supplement: Supplementary file 3 — Supplementary information [file 41598_2019_55573_MOESM3_ESM.zip › SupplementaryFile2/WITH_BREAK/mesodasys_log_rf.png]

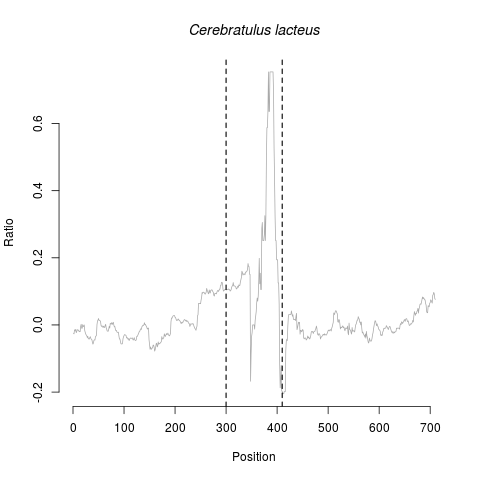

Supplement: Supplementary file 3 — Supplementary information [file 41598_2019_55573_MOESM3_ESM.zip › SupplementaryFile2/WITH_BREAK/cerebratulus_log_fr.png]

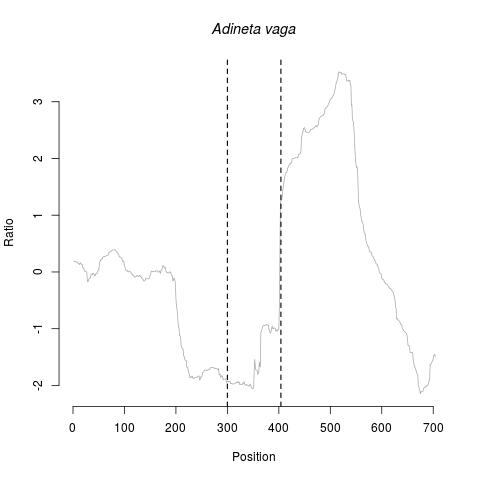

Supplement: Supplementary file 3 — Supplementary information [file 41598_2019_55573_MOESM3_ESM.zip › SupplementaryFile2/WITH_BREAK/adineta_log_rf.png]

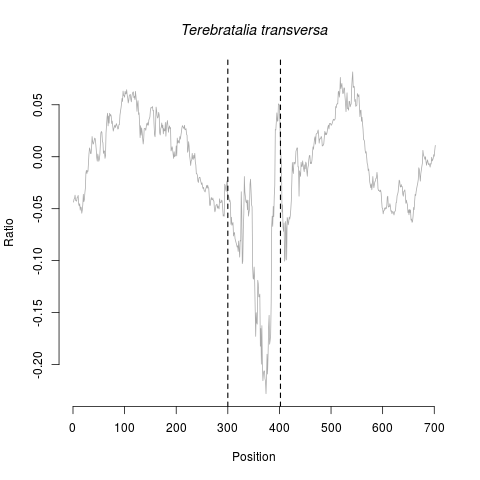

Supplement: Supplementary file 3 — Supplementary information [file 41598_2019_55573_MOESM3_ESM.zip › SupplementaryFile2/WITH_BREAK/terebratalia_log_rf.png]

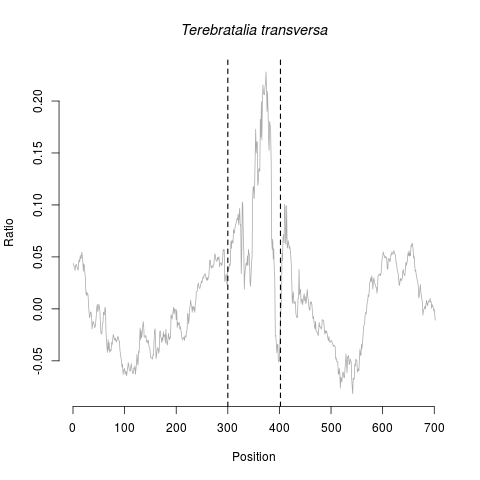

Supplement: Supplementary file 3 — Supplementary information [file 41598_2019_55573_MOESM3_ESM.zip › SupplementaryFile2/WITH_BREAK/terebratalia_log_fr.png]

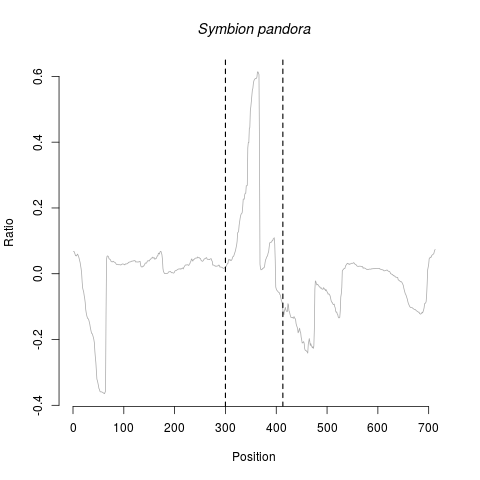

Supplement: Supplementary file 3 — Supplementary information [file 41598_2019_55573_MOESM3_ESM.zip › SupplementaryFile2/WITH_BREAK/symbion_log_rf.png]

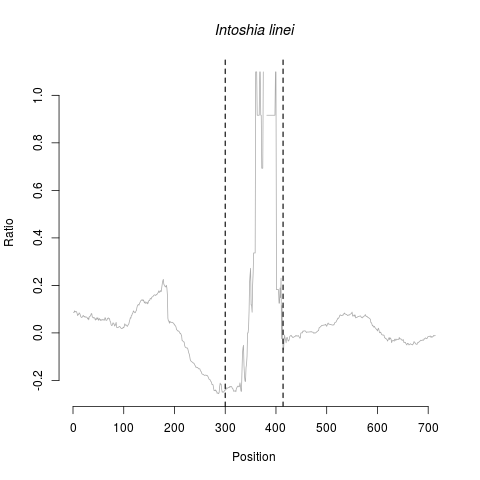

Supplement: Supplementary file 3 — Supplementary information [file 41598_2019_55573_MOESM3_ESM.zip › SupplementaryFile2/WITH_BREAK/intoshia_log_fr.png]

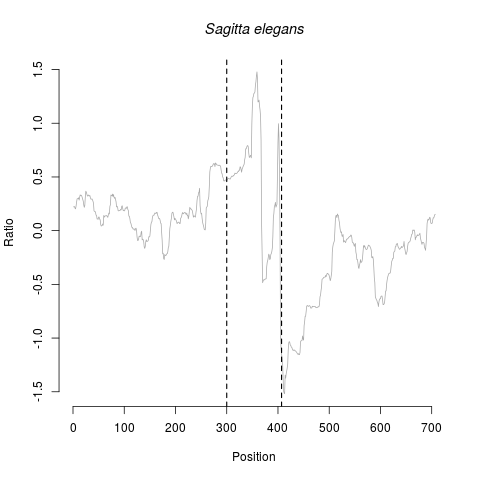

Supplement: Supplementary file 3 — Supplementary information [file 41598_2019_55573_MOESM3_ESM.zip › SupplementaryFile2/WITH_BREAK/sagitta_log_rf.png]

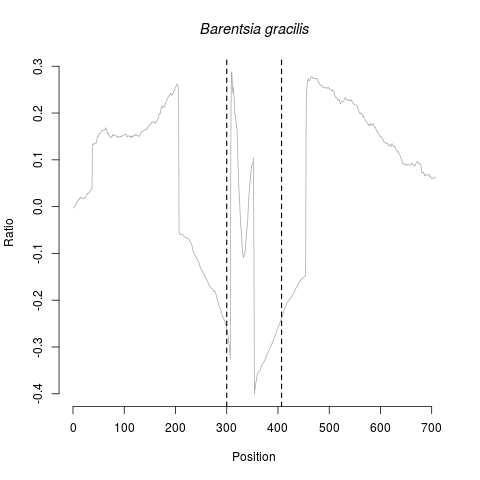

Supplement: Supplementary file 3 — Supplementary information [file 41598_2019_55573_MOESM3_ESM.zip › SupplementaryFile2/WITH_BREAK/barentsia_log_fr.png]

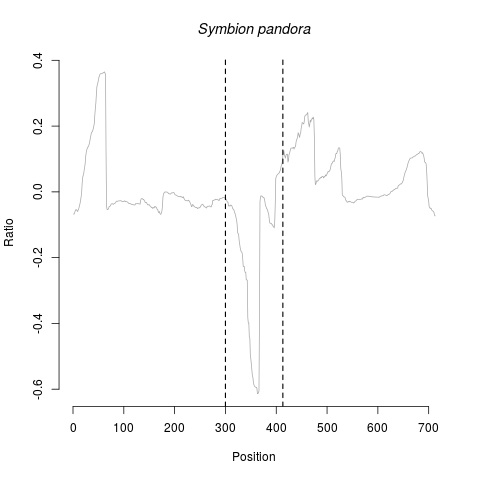

Supplement: Supplementary file 3 — Supplementary information [file 41598_2019_55573_MOESM3_ESM.zip › SupplementaryFile2/WITH_BREAK/symbion_log_fr.png]

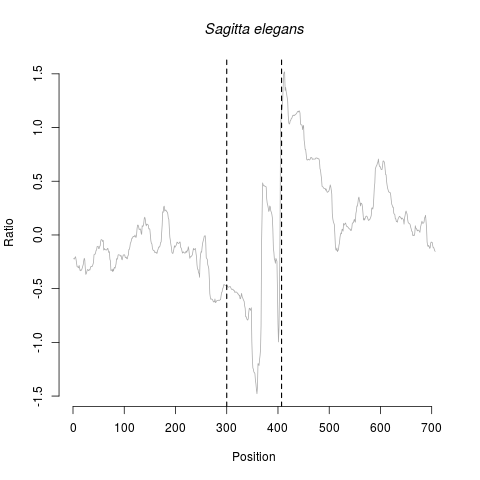

Supplement: Supplementary file 3 — Supplementary information [file 41598_2019_55573_MOESM3_ESM.zip › SupplementaryFile2/WITH_BREAK/sagitta_log_fr.png]

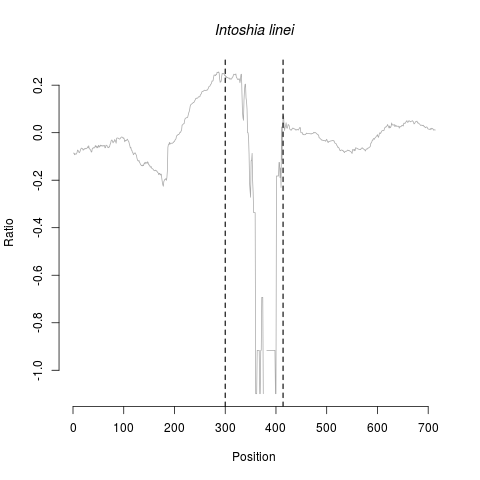

Supplement: Supplementary file 3 — Supplementary information [file 41598_2019_55573_MOESM3_ESM.zip › SupplementaryFile2/WITH_BREAK/intoshia_log_rf.png]

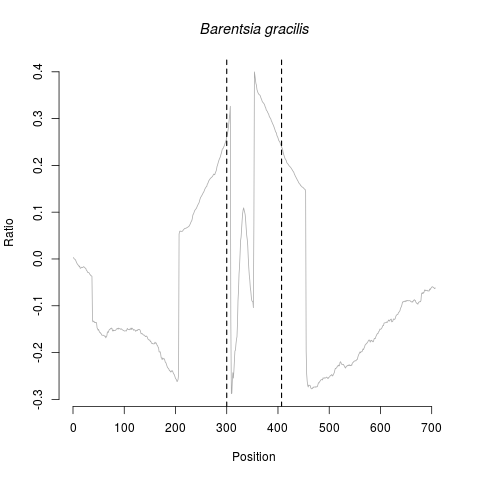

Supplement: Supplementary file 3 — Supplementary information [file 41598_2019_55573_MOESM3_ESM.zip › SupplementaryFile2/WITH_BREAK/barentsia_log_rf.png]

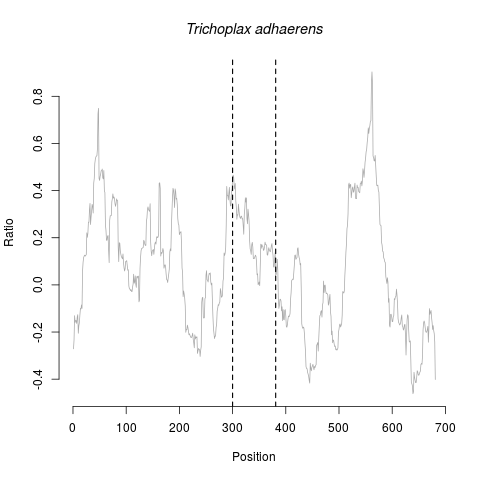

Supplement: Supplementary file 3 — Supplementary information [file 41598_2019_55573_MOESM3_ESM.zip › SupplementaryFile2/WITHOUT_BREAK/trichoplax_log_fr.png]

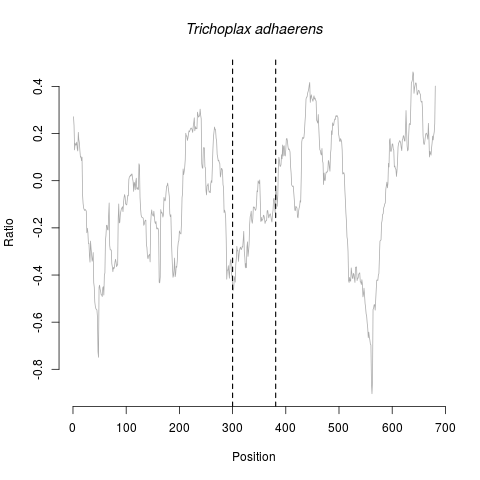

Supplement: Supplementary file 3 — Supplementary information [file 41598_2019_55573_MOESM3_ESM.zip › SupplementaryFile2/WITHOUT_BREAK/trichoplax_log_rf.png]

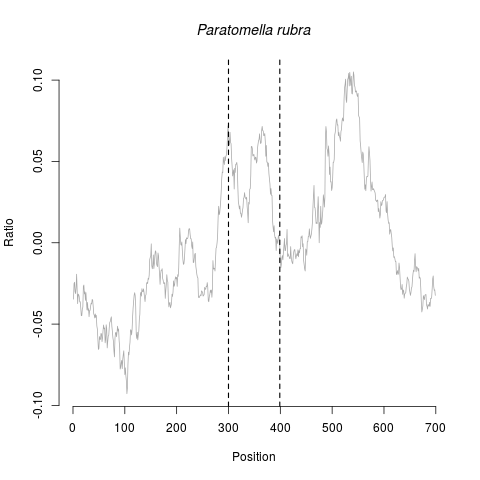

Supplement: Supplementary file 3 — Supplementary information [file 41598_2019_55573_MOESM3_ESM.zip › SupplementaryFile2/WITHOUT_BREAK/paratomella_log_fr.png]

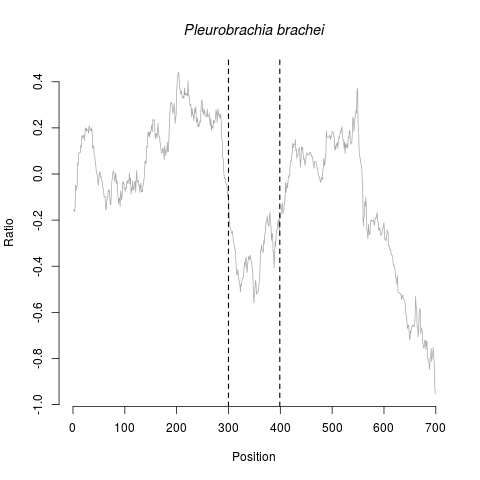

Supplement: Supplementary file 3 — Supplementary information [file 41598_2019_55573_MOESM3_ESM.zip › SupplementaryFile2/WITHOUT_BREAK/pleurobrachia_log_rf.png]

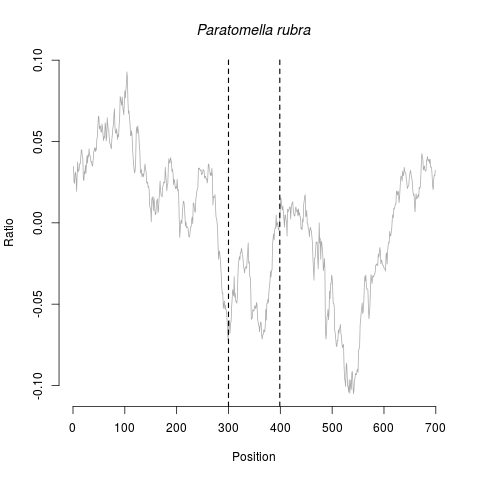

Supplement: Supplementary file 3 — Supplementary information [file 41598_2019_55573_MOESM3_ESM.zip › SupplementaryFile2/WITHOUT_BREAK/paratomella_log_rf.png]

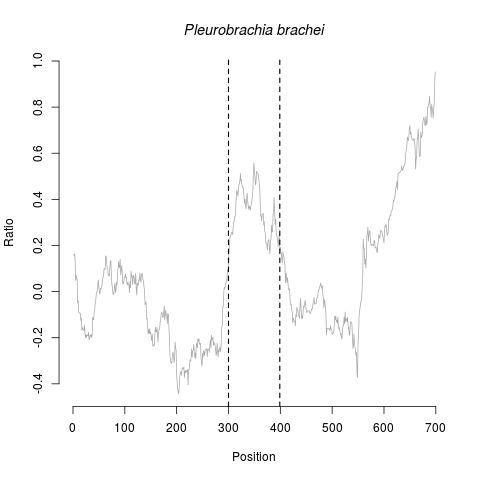

Supplement: Supplementary file 3 — Supplementary information [file 41598_2019_55573_MOESM3_ESM.zip › SupplementaryFile2/WITHOUT_BREAK/pleurobrachia_log_fr.png]

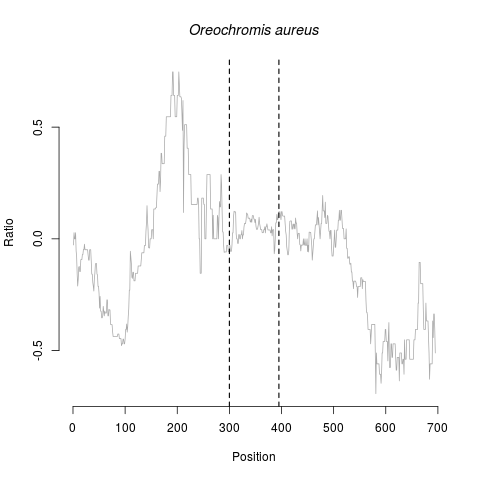

Supplement: Supplementary file 3 — Supplementary information [file 41598_2019_55573_MOESM3_ESM.zip › SupplementaryFile2/WITHOUT_BREAK/oreochromis_log_fr.png]

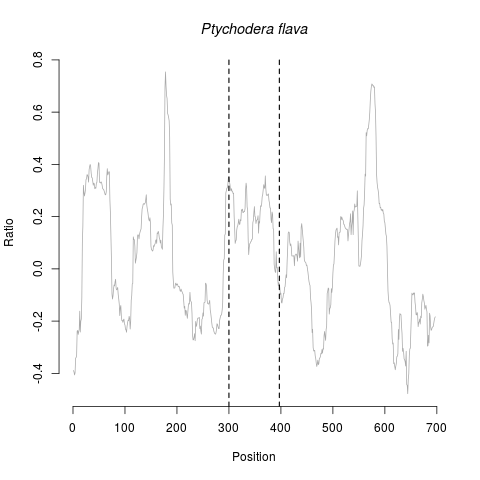

Supplement: Supplementary file 3 — Supplementary information [file 41598_2019_55573_MOESM3_ESM.zip › SupplementaryFile2/WITHOUT_BREAK/ptychodera_log_rf.png]

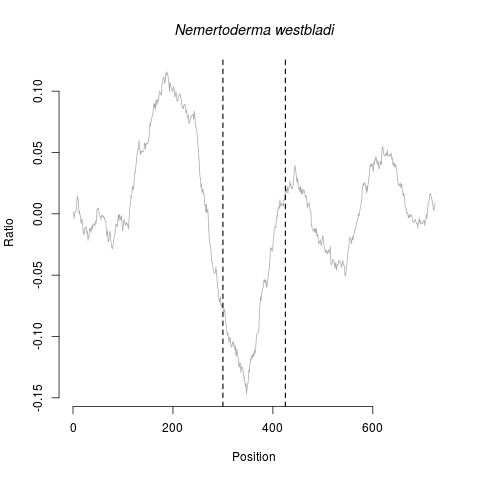

Supplement: Supplementary file 3 — Supplementary information [file 41598_2019_55573_MOESM3_ESM.zip › SupplementaryFile2/WITHOUT_BREAK/nemertoderma_log_fr.png]

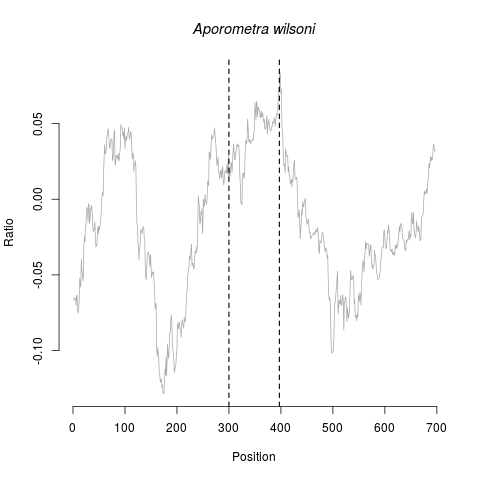

Supplement: Supplementary file 3 — Supplementary information [file 41598_2019_55573_MOESM3_ESM.zip › SupplementaryFile2/WITHOUT_BREAK/aporometra_log_rf.png]

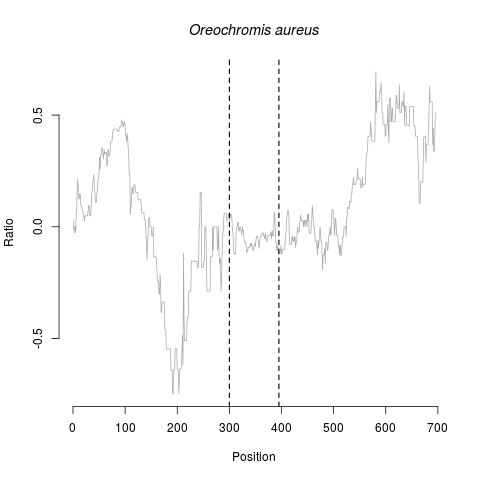

Supplement: Supplementary file 3 — Supplementary information [file 41598_2019_55573_MOESM3_ESM.zip › SupplementaryFile2/WITHOUT_BREAK/oreochromis_log_rf.png]

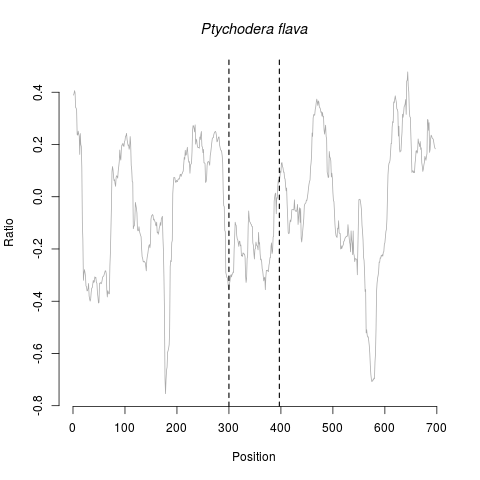

Supplement: Supplementary file 3 — Supplementary information [file 41598_2019_55573_MOESM3_ESM.zip › SupplementaryFile2/WITHOUT_BREAK/ptychodera_log_fr.png]

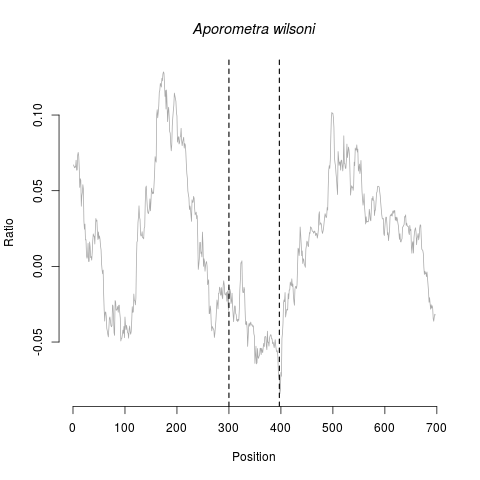

Supplement: Supplementary file 3 — Supplementary information [file 41598_2019_55573_MOESM3_ESM.zip › SupplementaryFile2/WITHOUT_BREAK/aporometra_log_fr.png]

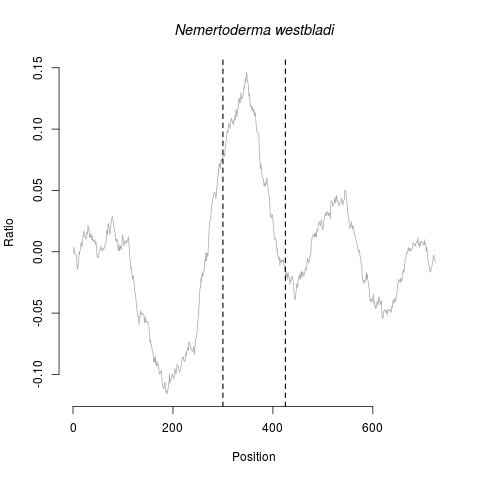

Supplement: Supplementary file 3 — Supplementary information [file 41598_2019_55573_MOESM3_ESM.zip › SupplementaryFile2/WITHOUT_BREAK/nemertoderma_log_rf.png]
