# Supplementary figures and images for: Computational discovery of hidden breaks in 28S ribosomal RNAs across eukaryotes and consequences for RNA Integrity Numbers (part 2 of 3)
Source: Sci Rep. 2019 Dec 20;9:19477. doi: 10.1038/s41598-019-55573-1 (PMC6925239; doi:10.1038/s41598-019-55573-1)

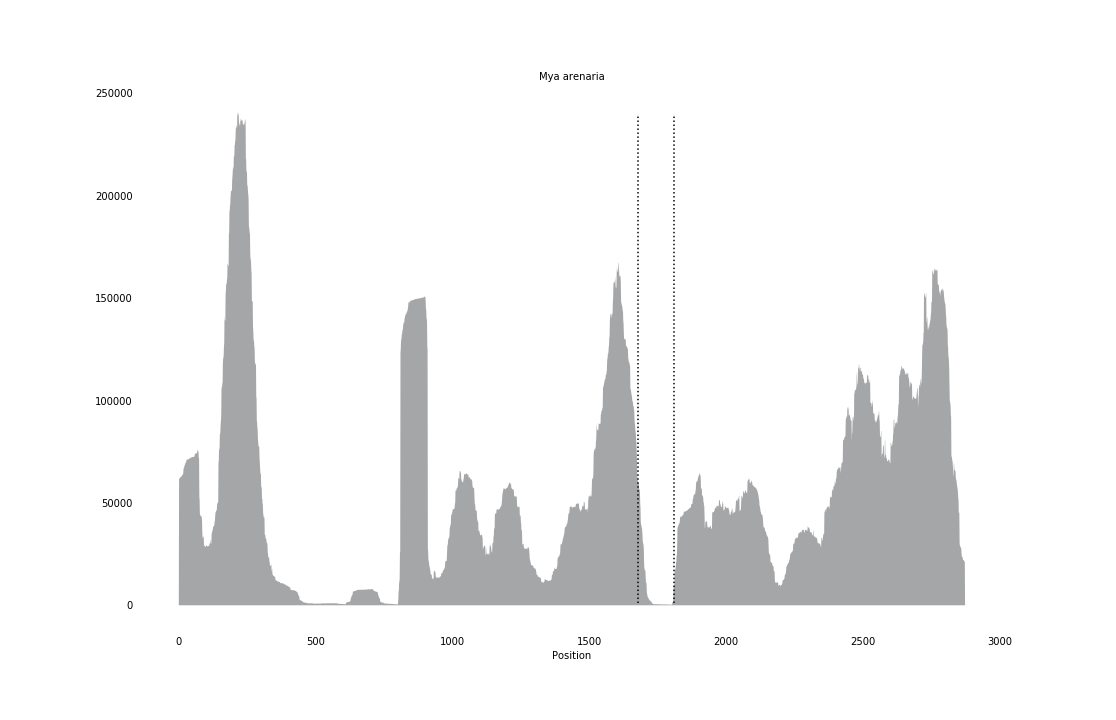

Supplement: Supplementary file 2 — Supplementary information [file 41598_2019_55573_MOESM2_ESM.zip › SupplementaryFile1/Metazoa/Protostomia/Mollusca/Mya_arenaria_coverage_correct.png]

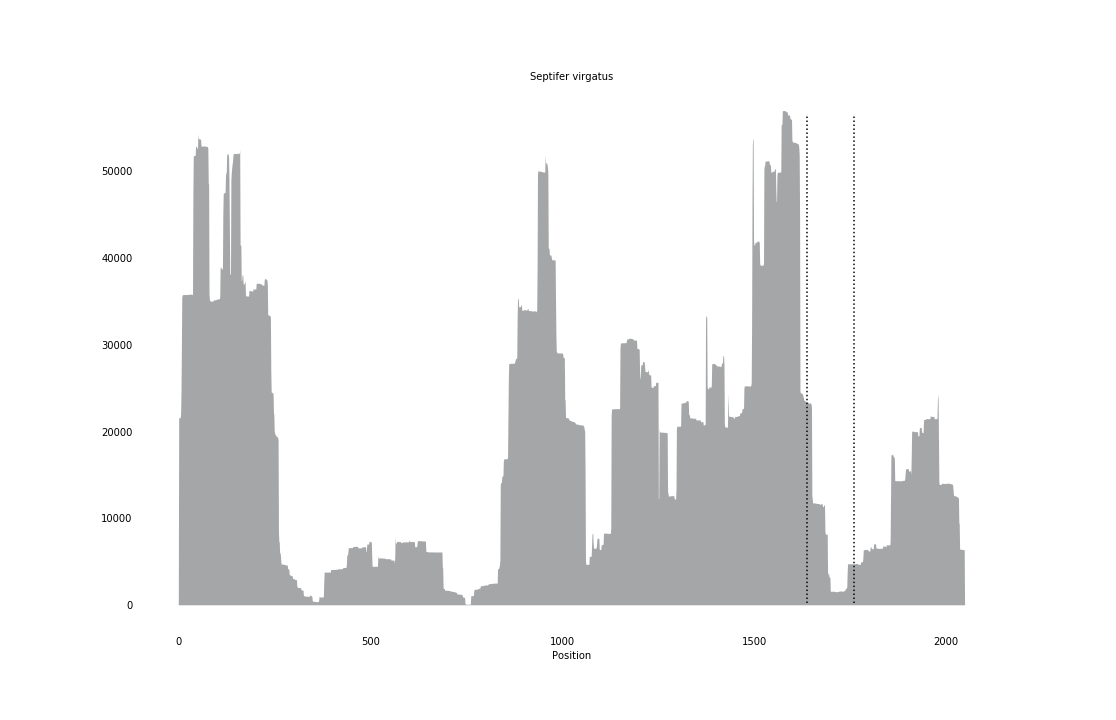

Supplement: Supplementary file 2 — Supplementary information [file 41598_2019_55573_MOESM2_ESM.zip › SupplementaryFile1/Metazoa/Protostomia/Mollusca/Septifer_virgatus_coverage_correct.png]

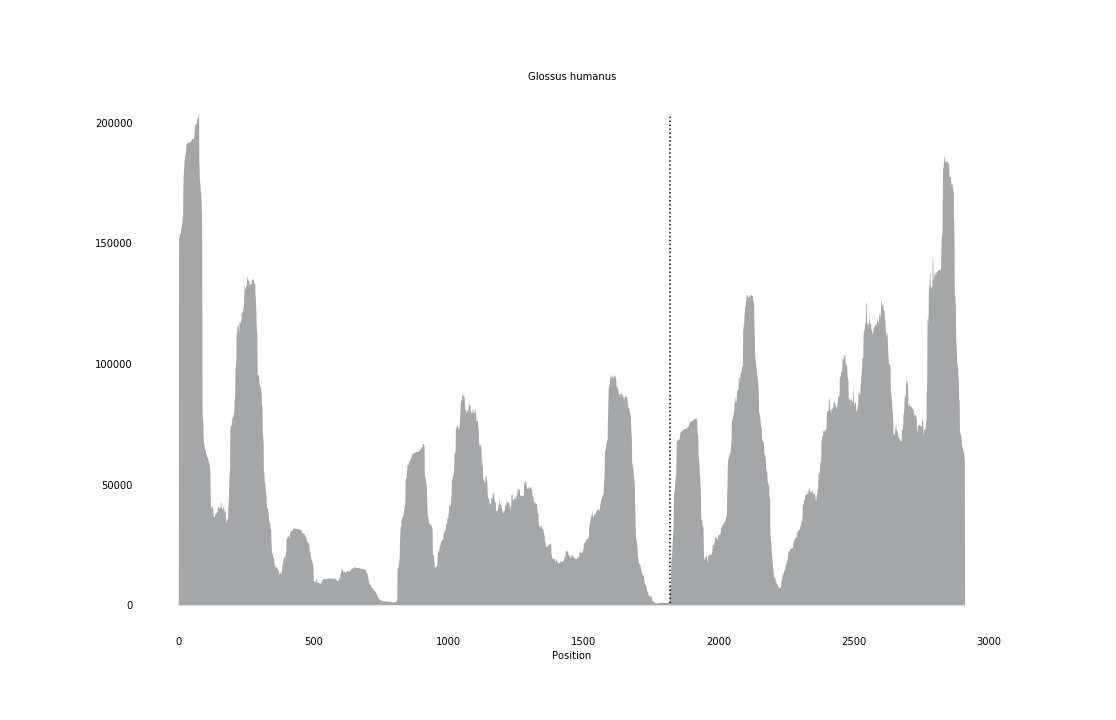

Supplement: Supplementary file 2 — Supplementary information [file 41598_2019_55573_MOESM2_ESM.zip › SupplementaryFile1/Metazoa/Protostomia/Mollusca/Glossus_humanus_coverage.png]

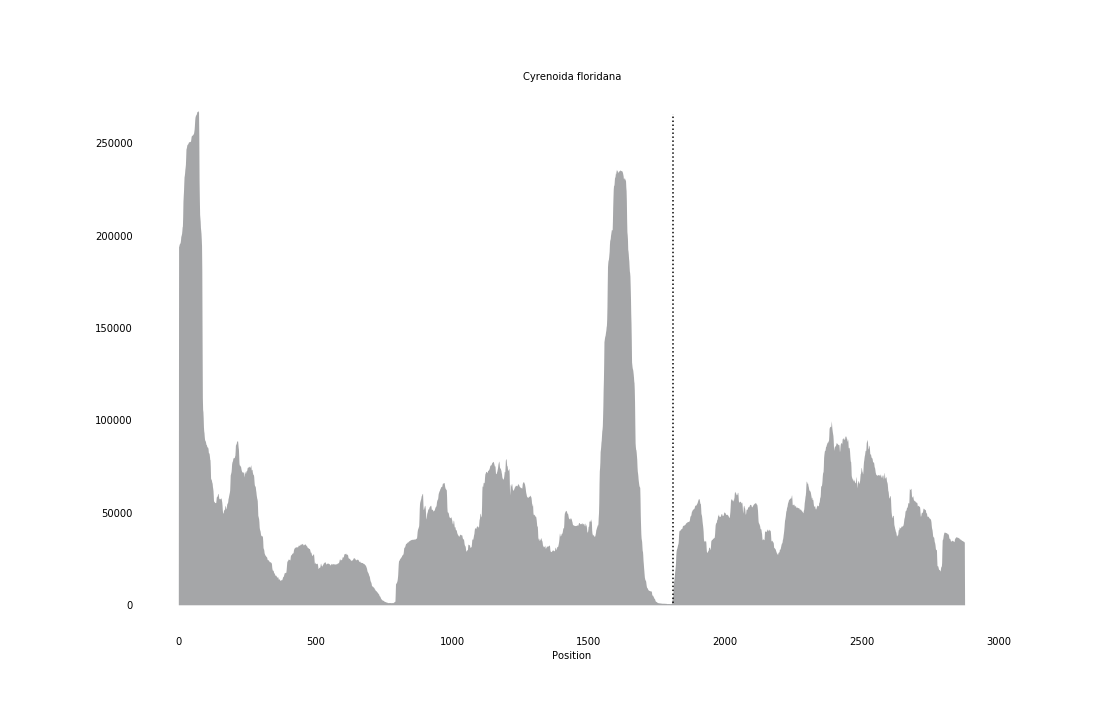

Supplement: Supplementary file 2 — Supplementary information [file 41598_2019_55573_MOESM2_ESM.zip › SupplementaryFile1/Metazoa/Protostomia/Mollusca/Cyrenoida_floridana_coverage.png]

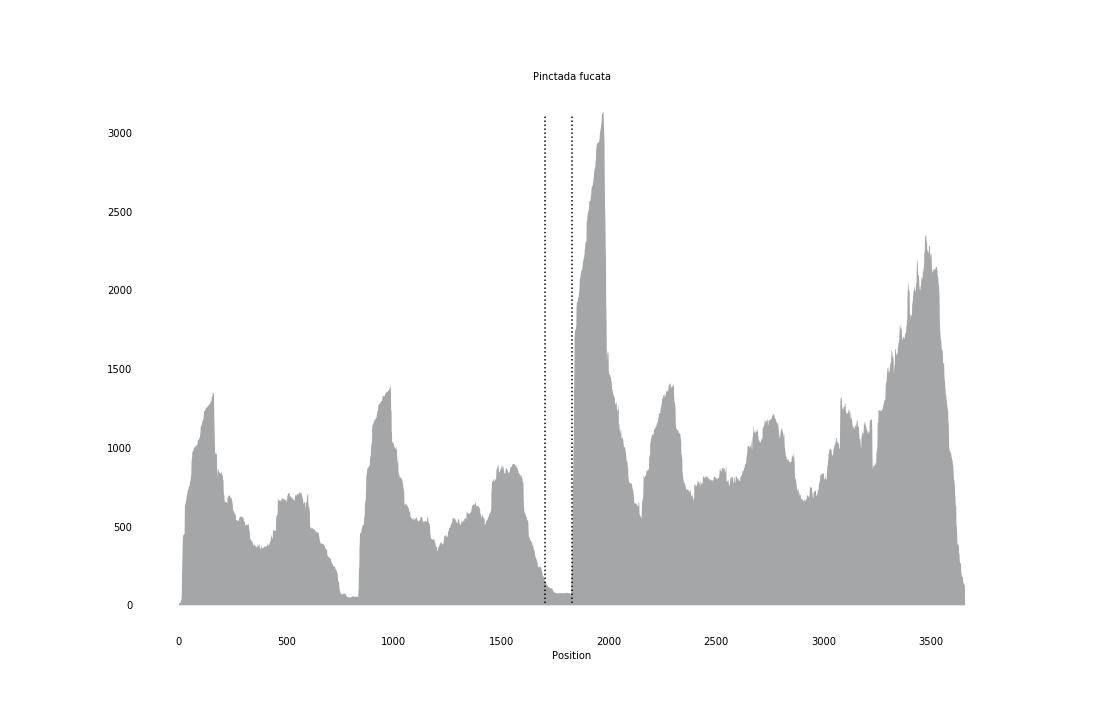

Supplement: Supplementary file 2 — Supplementary information [file 41598_2019_55573_MOESM2_ESM.zip › SupplementaryFile1/Metazoa/Protostomia/Mollusca/Pinctada_fucata_coverage_correct.png]

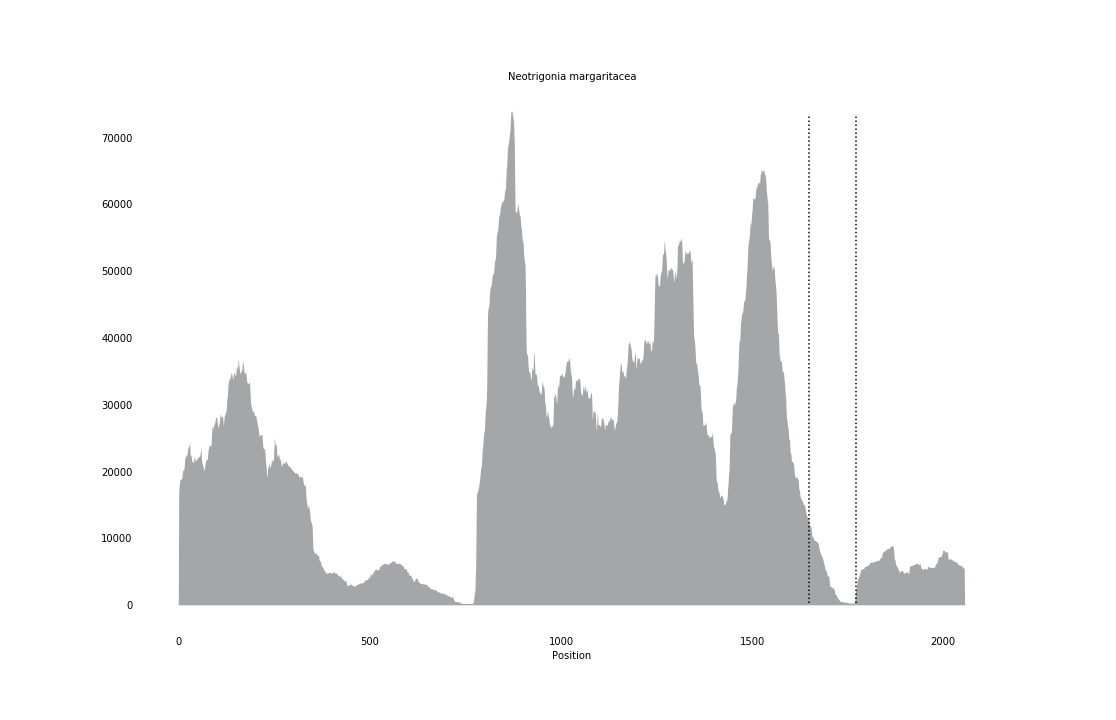

Supplement: Supplementary file 2 — Supplementary information [file 41598_2019_55573_MOESM2_ESM.zip › SupplementaryFile1/Metazoa/Protostomia/Mollusca/Neotrigonia_margaritacea_coverage_correct.png]

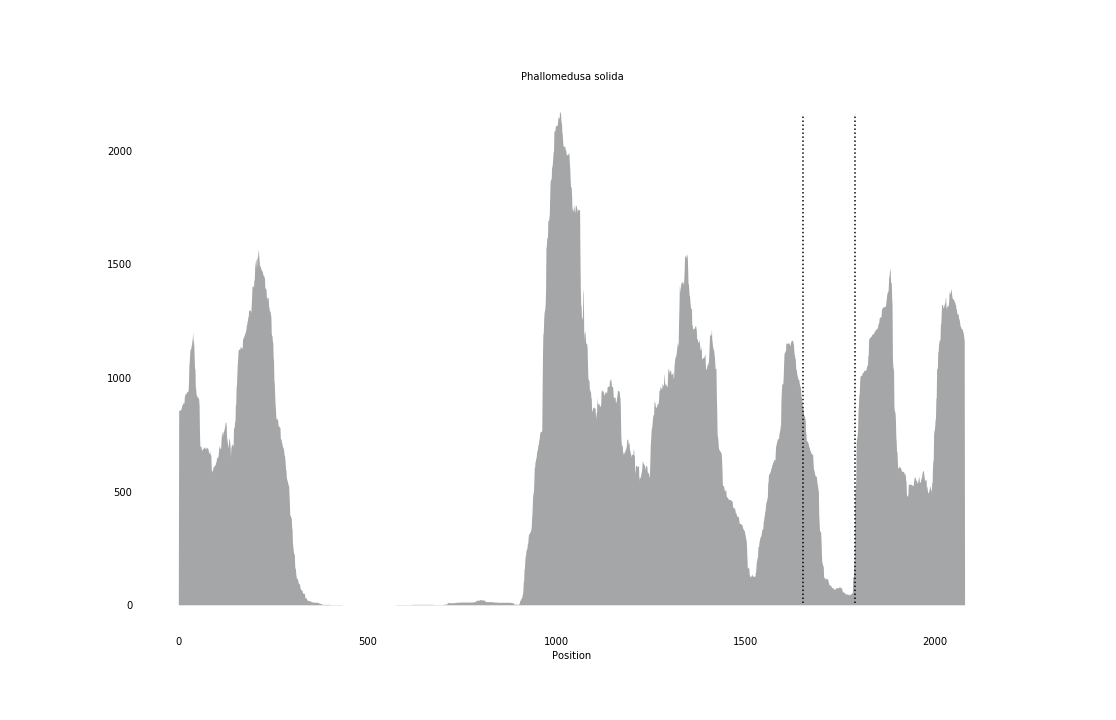

Supplement: Supplementary file 2 — Supplementary information [file 41598_2019_55573_MOESM2_ESM.zip › SupplementaryFile1/Metazoa/Protostomia/Mollusca/Phallomedusa_solida_coverage_correct.png]

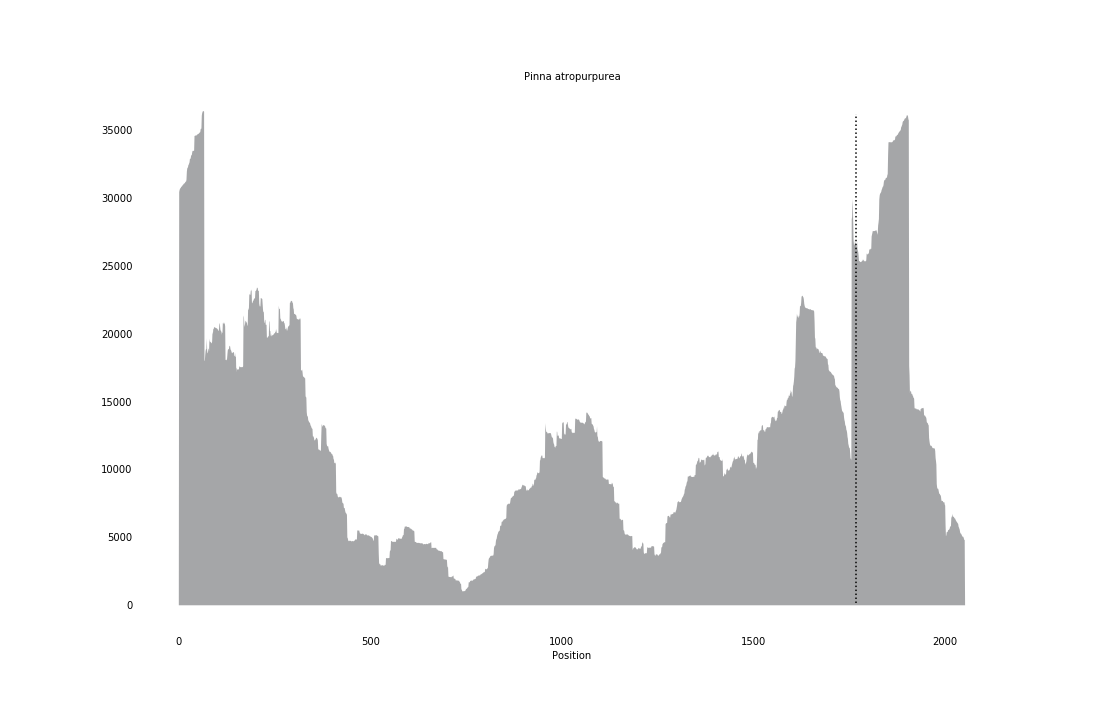

Supplement: Supplementary file 2 — Supplementary information [file 41598_2019_55573_MOESM2_ESM.zip › SupplementaryFile1/Metazoa/Protostomia/Mollusca/Pinna_atropurpurea_coverage.png]

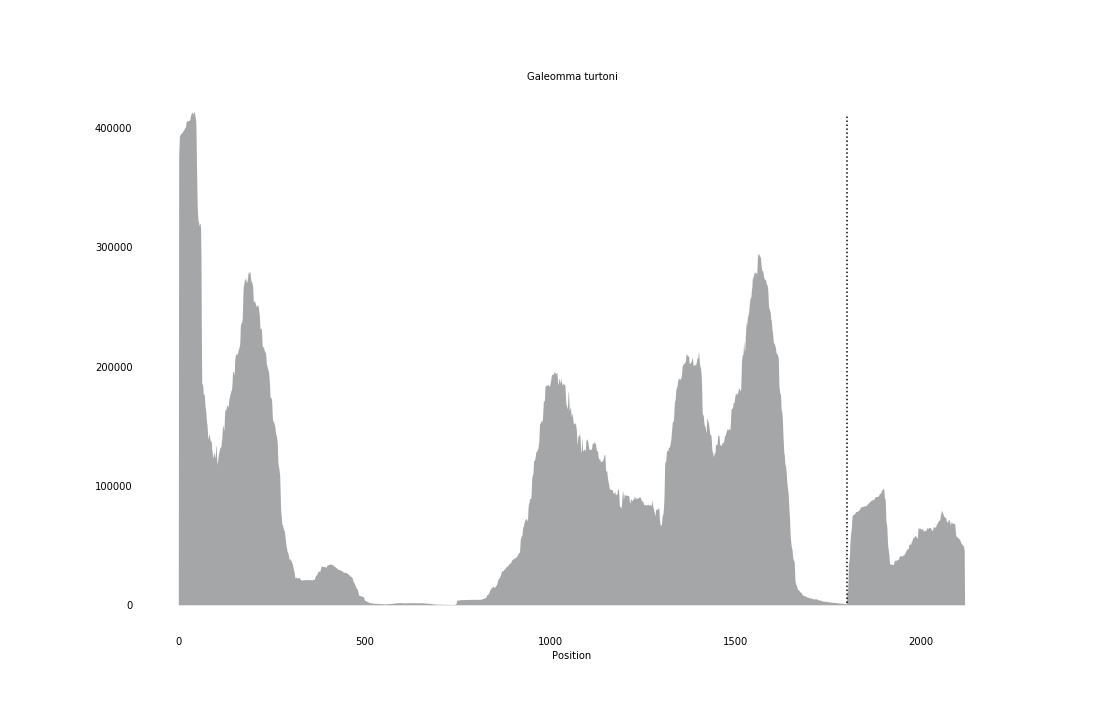

Supplement: Supplementary file 2 — Supplementary information [file 41598_2019_55573_MOESM2_ESM.zip › SupplementaryFile1/Metazoa/Protostomia/Mollusca/Galeomma_turtoni_coverage.png]

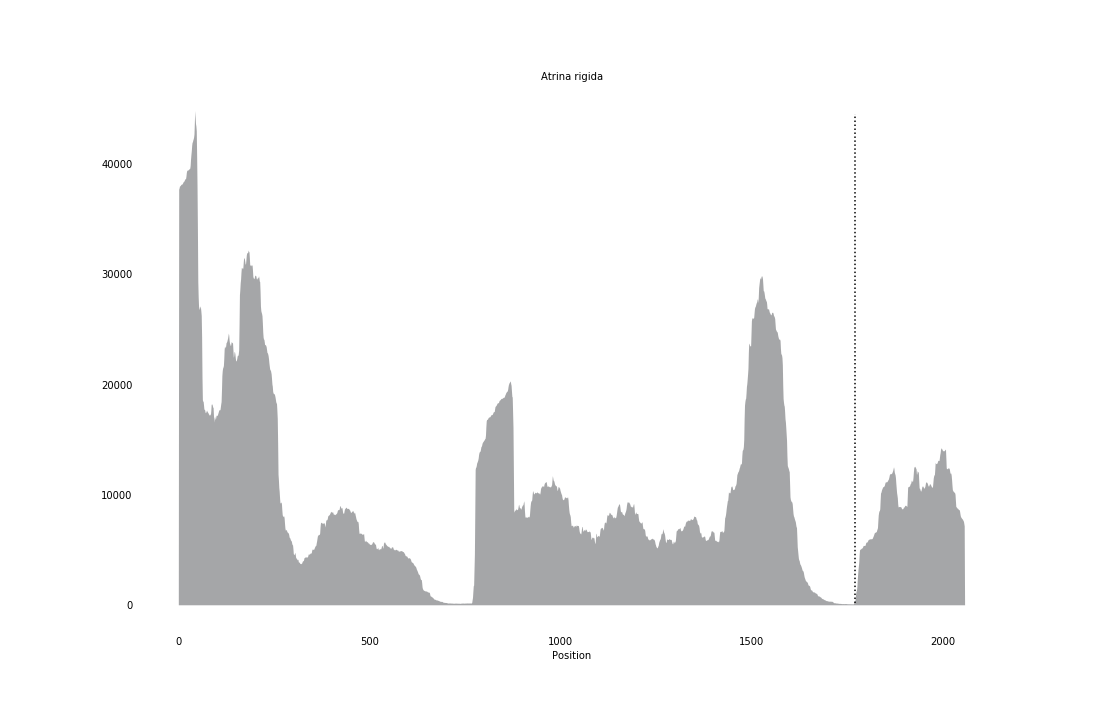

Supplement: Supplementary file 2 — Supplementary information [file 41598_2019_55573_MOESM2_ESM.zip › SupplementaryFile1/Metazoa/Protostomia/Mollusca/Atrina_rigida_coverage.png]

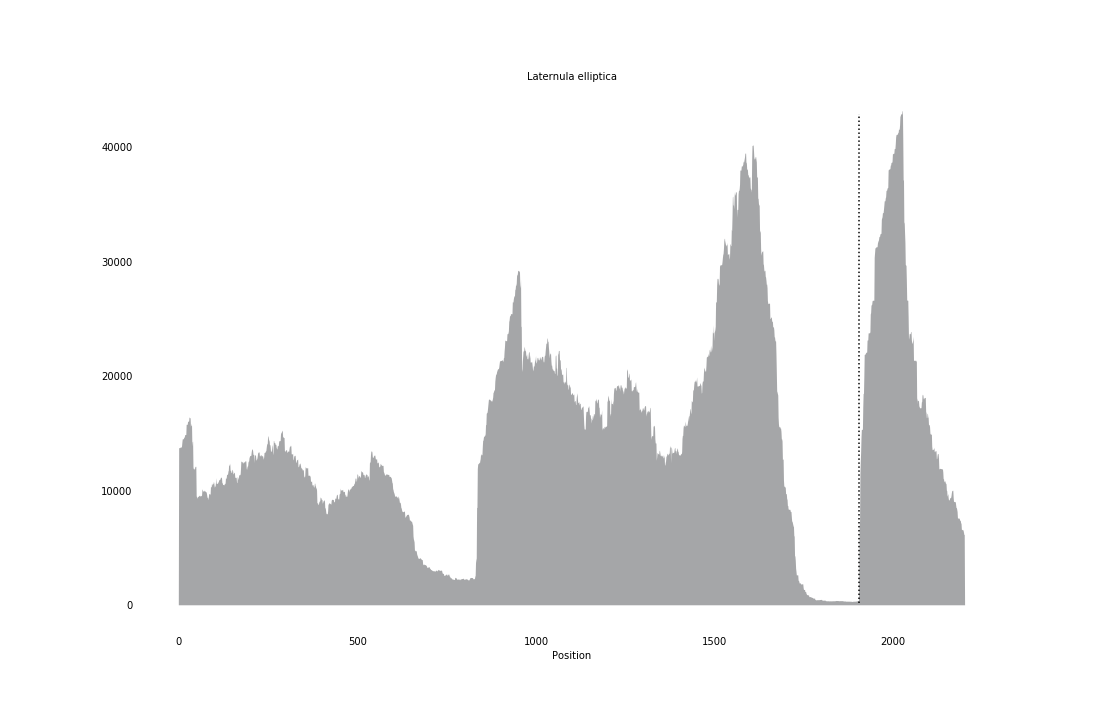

Supplement: Supplementary file 2 — Supplementary information [file 41598_2019_55573_MOESM2_ESM.zip › SupplementaryFile1/Metazoa/Protostomia/Mollusca/Laternula_elliptica_coverage.png]

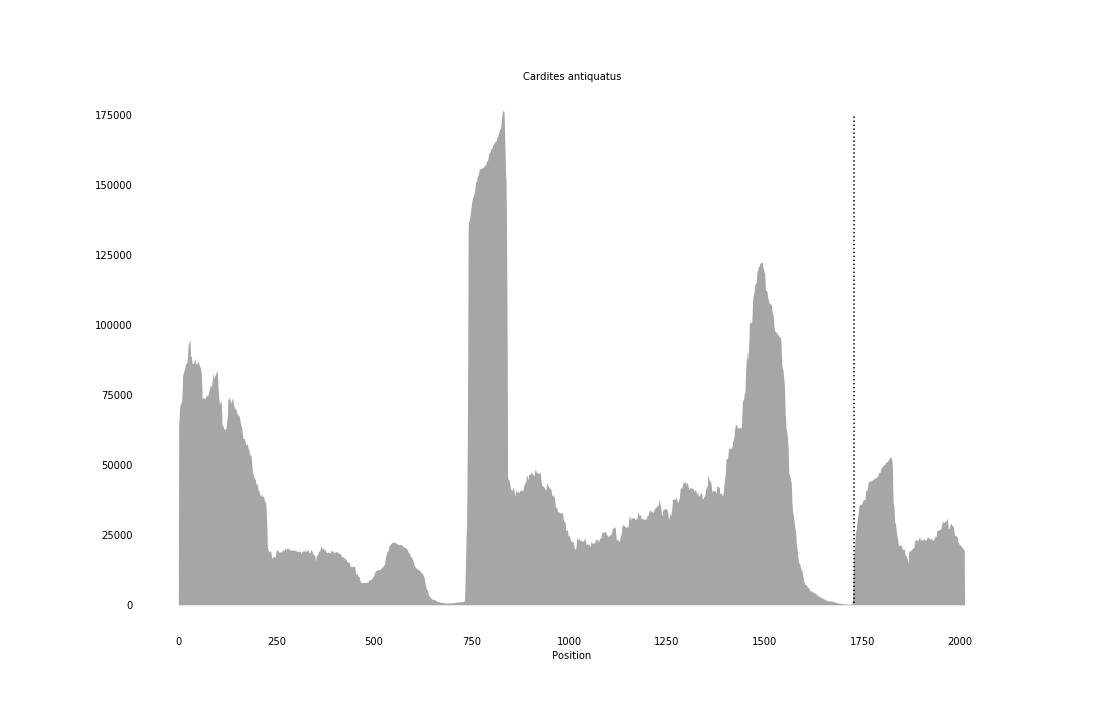

Supplement: Supplementary file 2 — Supplementary information [file 41598_2019_55573_MOESM2_ESM.zip › SupplementaryFile1/Metazoa/Protostomia/Mollusca/Cardites_antiquatus_coverage.png]

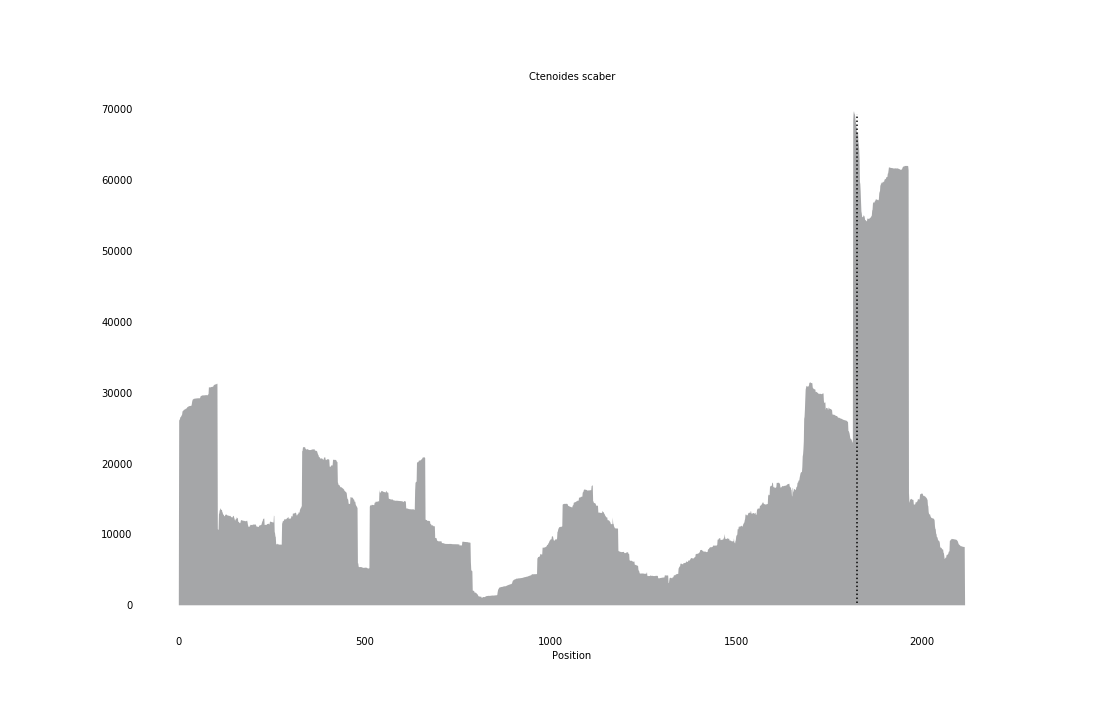

Supplement: Supplementary file 2 — Supplementary information [file 41598_2019_55573_MOESM2_ESM.zip › SupplementaryFile1/Metazoa/Protostomia/Mollusca/Ctenoides_scaber_coverage.png]

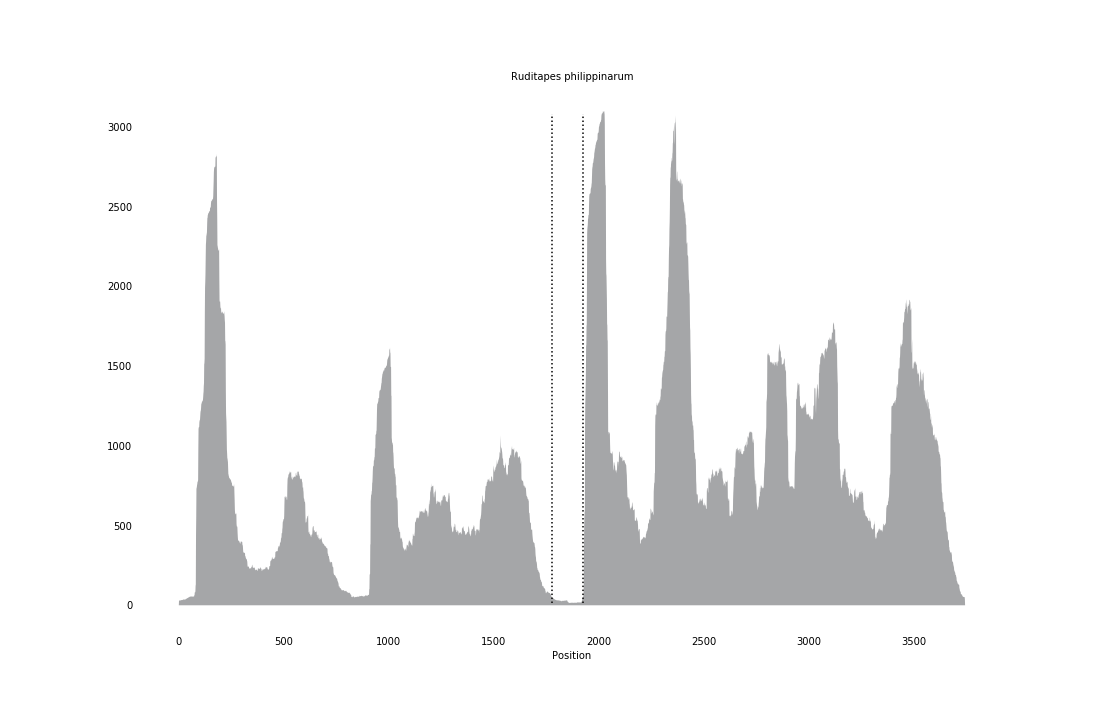

Supplement: Supplementary file 2 — Supplementary information [file 41598_2019_55573_MOESM2_ESM.zip › SupplementaryFile1/Metazoa/Protostomia/Mollusca/Ruditapes_philippinarum_coverage_correct.png]

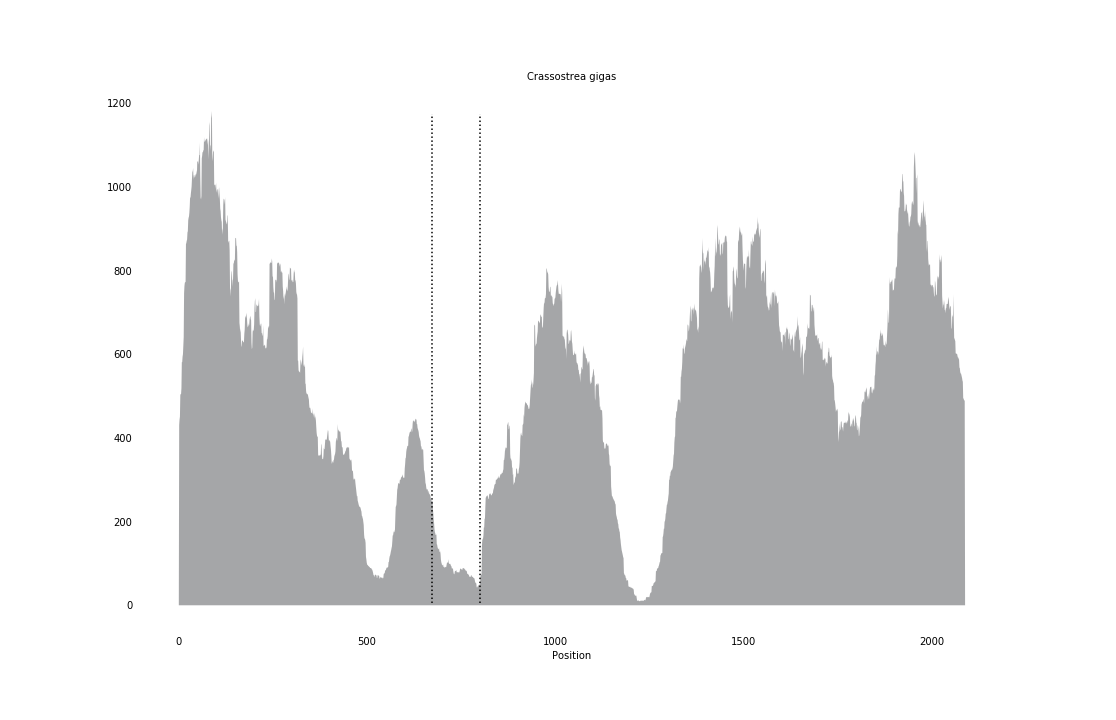

Supplement: Supplementary file 2 — Supplementary information [file 41598_2019_55573_MOESM2_ESM.zip › SupplementaryFile1/Metazoa/Protostomia/Mollusca/Crassostrea_gigas_coverage_correct.png]

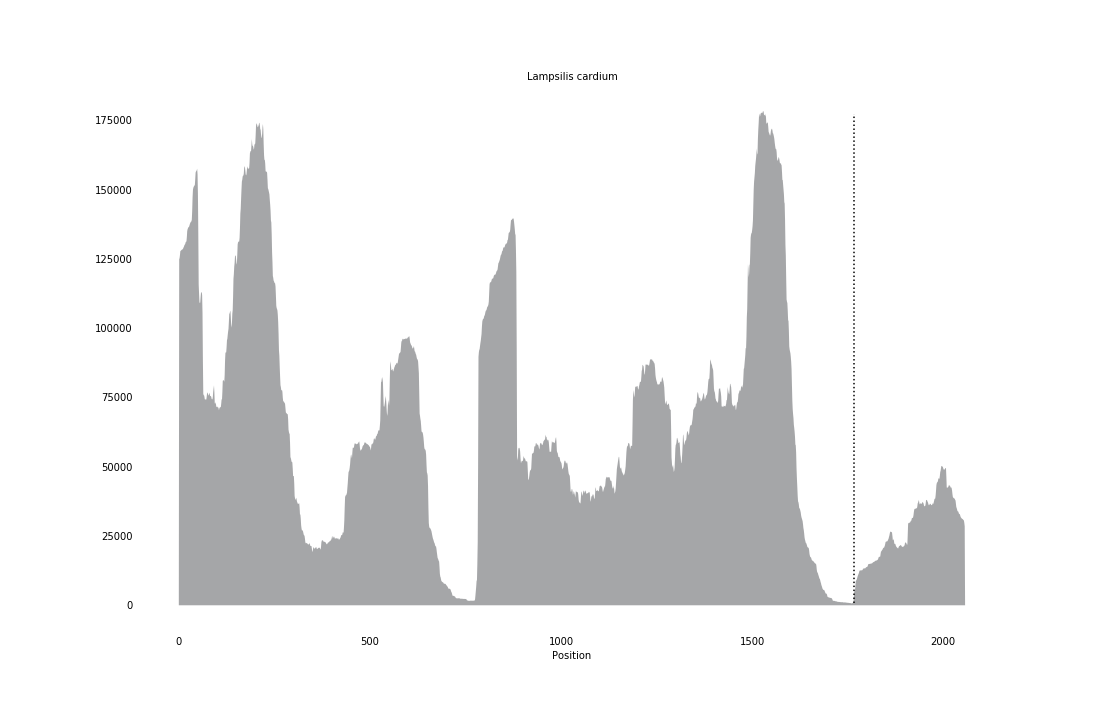

Supplement: Supplementary file 2 — Supplementary information [file 41598_2019_55573_MOESM2_ESM.zip › SupplementaryFile1/Metazoa/Protostomia/Mollusca/Lampsilis_cardium_coverage.png]

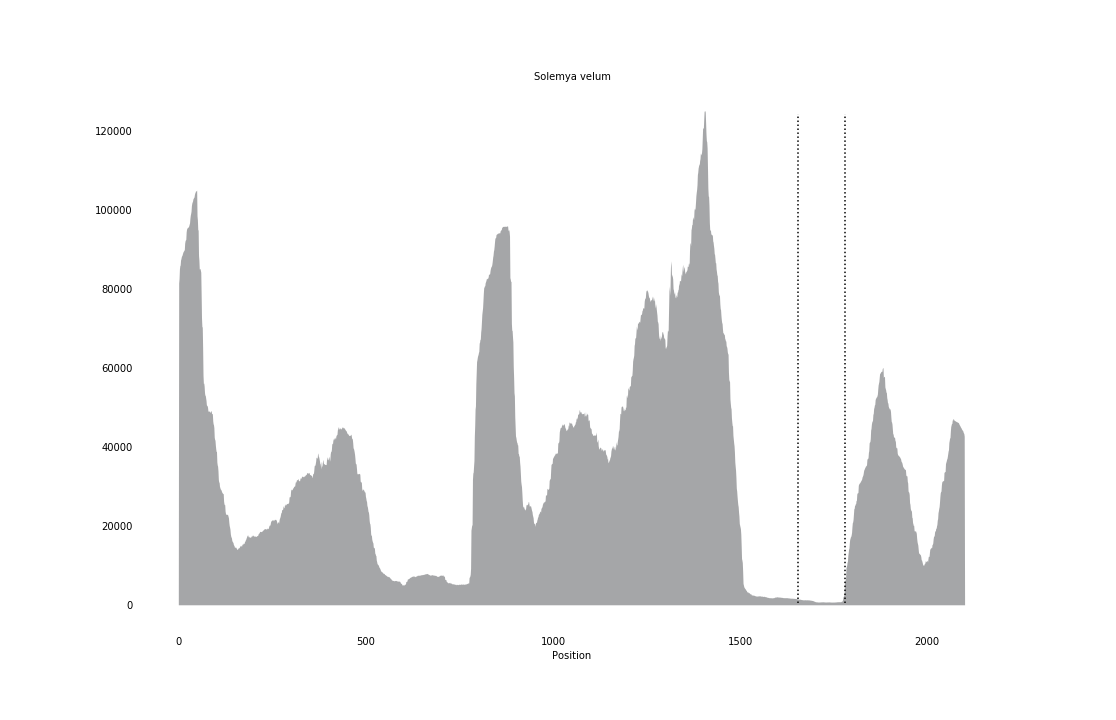

Supplement: Supplementary file 2 — Supplementary information [file 41598_2019_55573_MOESM2_ESM.zip › SupplementaryFile1/Metazoa/Protostomia/Mollusca/Solemya_velum_coverage_correct.png]

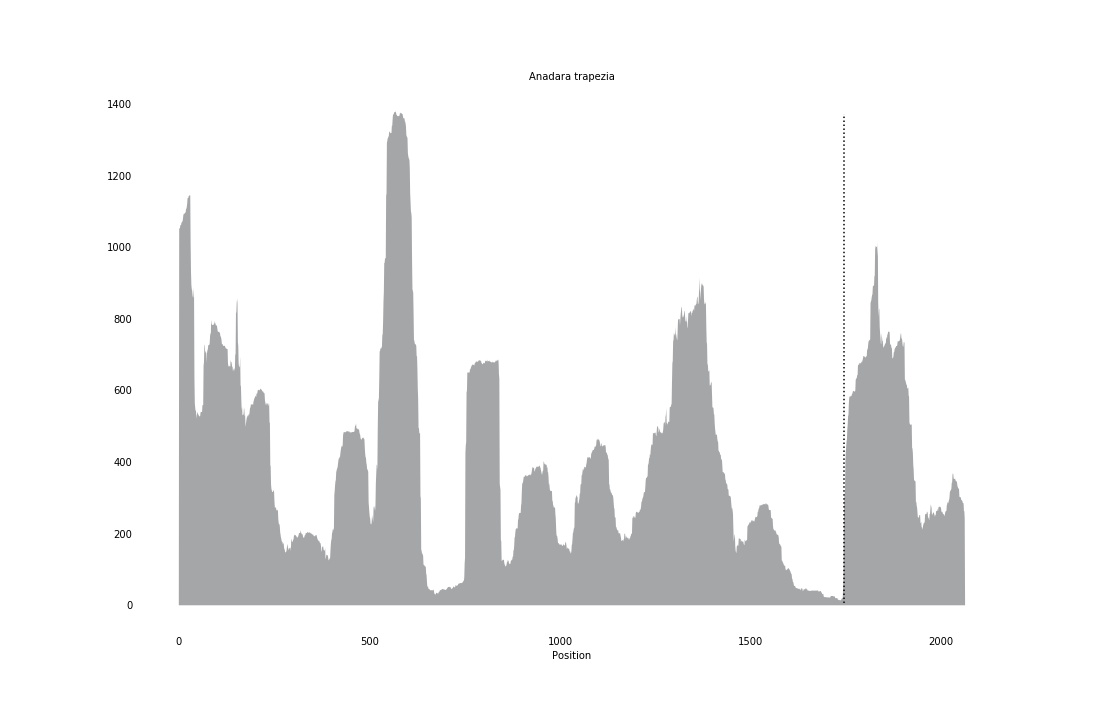

Supplement: Supplementary file 2 — Supplementary information [file 41598_2019_55573_MOESM2_ESM.zip › SupplementaryFile1/Metazoa/Protostomia/Mollusca/Anadara_trapezia_coverage.png]

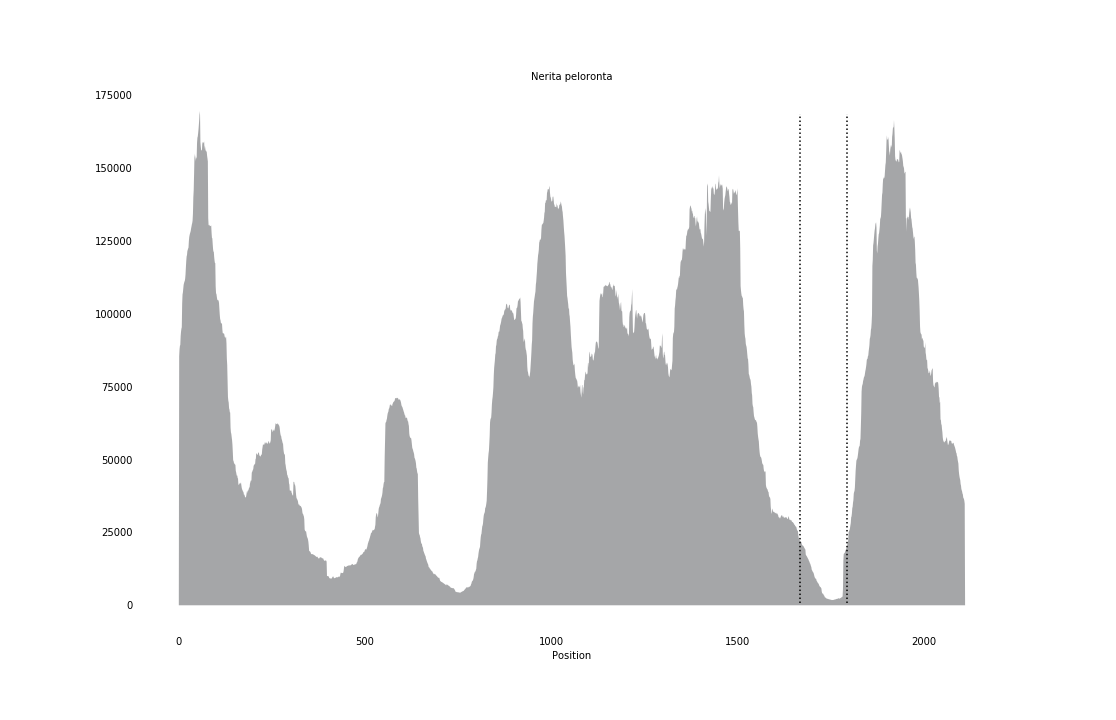

Supplement: Supplementary file 2 — Supplementary information [file 41598_2019_55573_MOESM2_ESM.zip › SupplementaryFile1/Metazoa/Protostomia/Mollusca/Nerita_peloronta_coverage_correct.png]

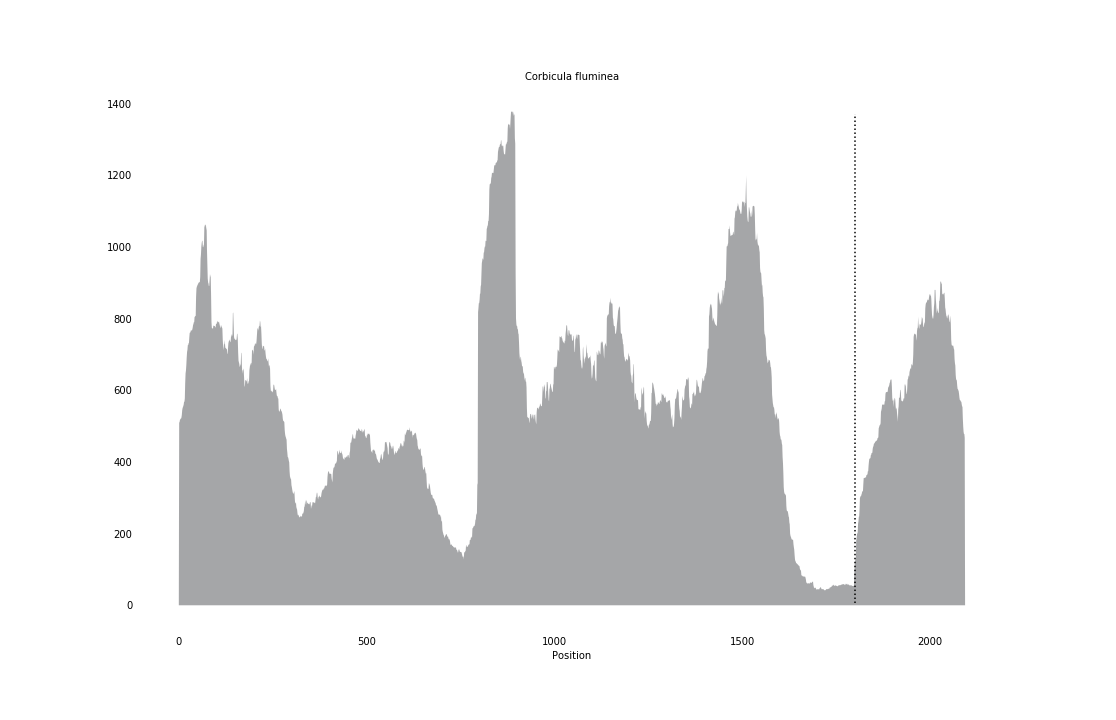

Supplement: Supplementary file 2 — Supplementary information [file 41598_2019_55573_MOESM2_ESM.zip › SupplementaryFile1/Metazoa/Protostomia/Mollusca/Corbicula_fluminea_coverage.png]

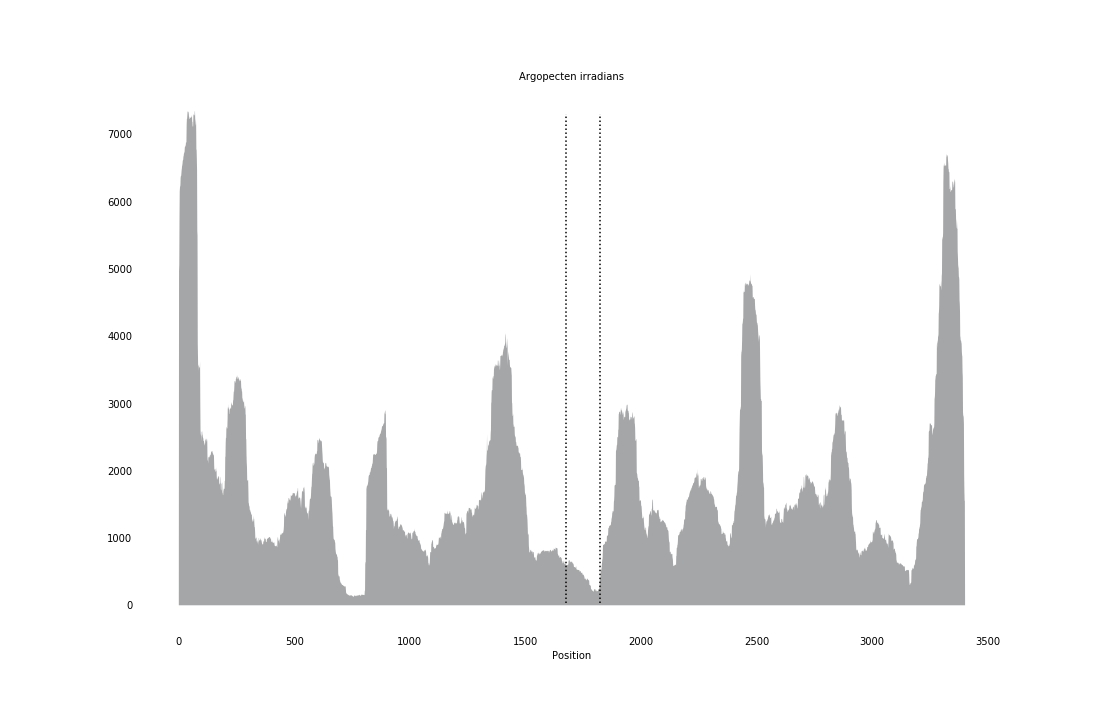

Supplement: Supplementary file 2 — Supplementary information [file 41598_2019_55573_MOESM2_ESM.zip › SupplementaryFile1/Metazoa/Protostomia/Mollusca/Argopecten_irradians_coverage_correct.png]

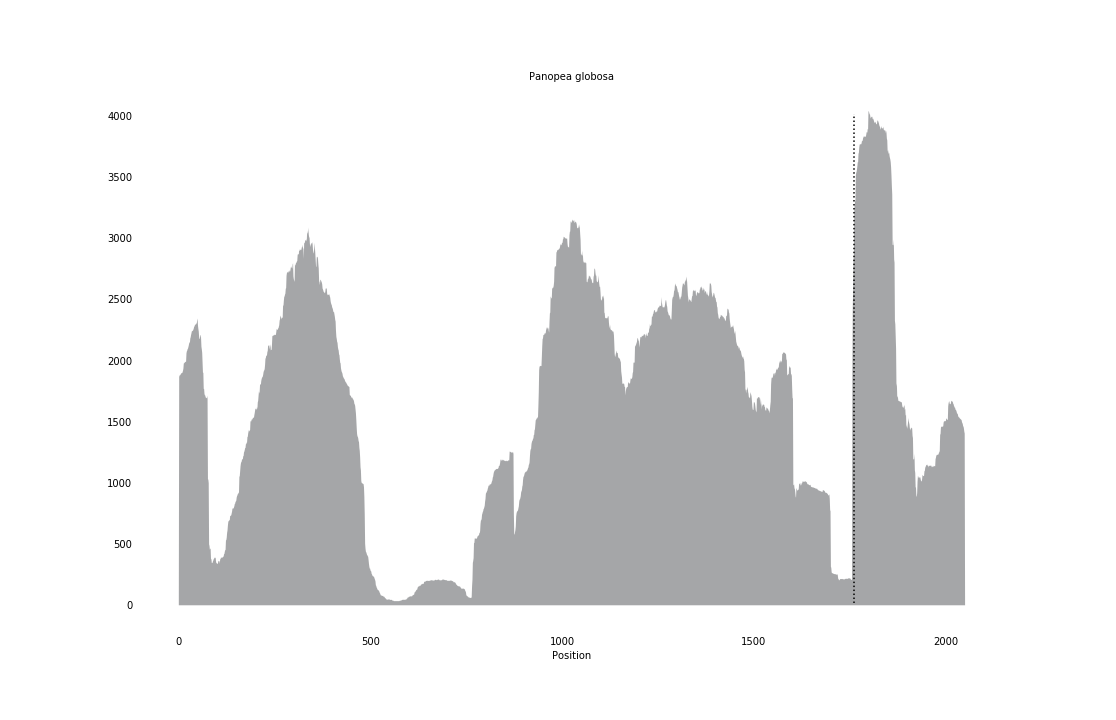

Supplement: Supplementary file 2 — Supplementary information [file 41598_2019_55573_MOESM2_ESM.zip › SupplementaryFile1/Metazoa/Protostomia/Mollusca/Panopea_globosa_coverage.png]

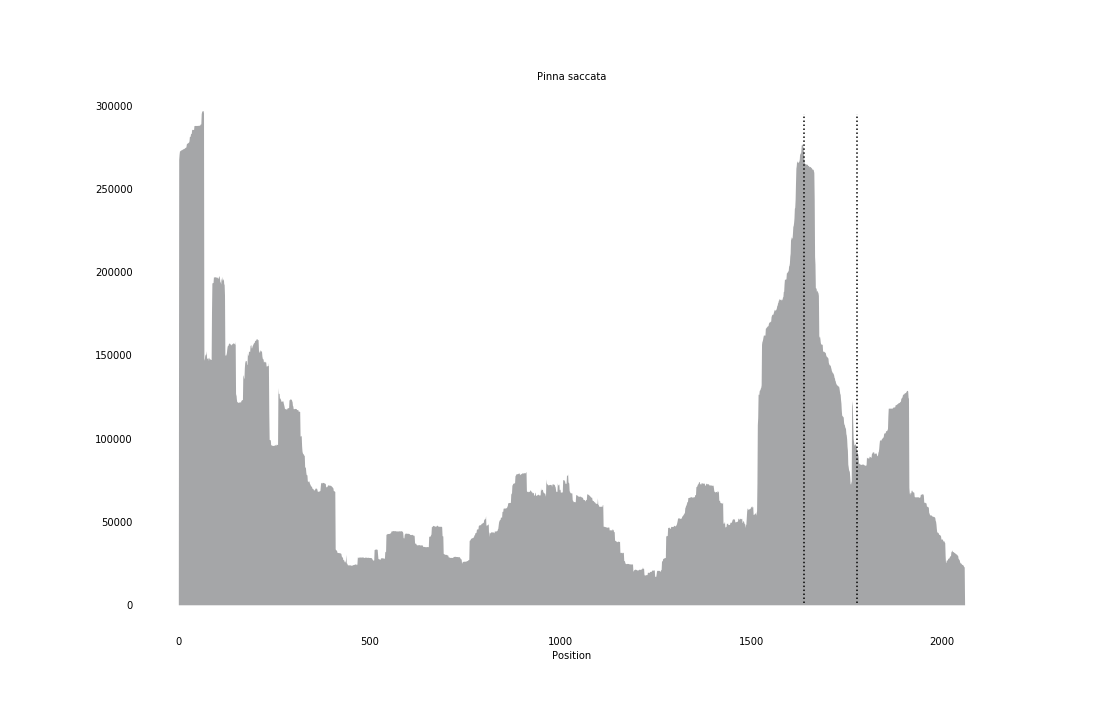

Supplement: Supplementary file 2 — Supplementary information [file 41598_2019_55573_MOESM2_ESM.zip › SupplementaryFile1/Metazoa/Protostomia/Mollusca/Pinna_saccata_coverage_correct.png]

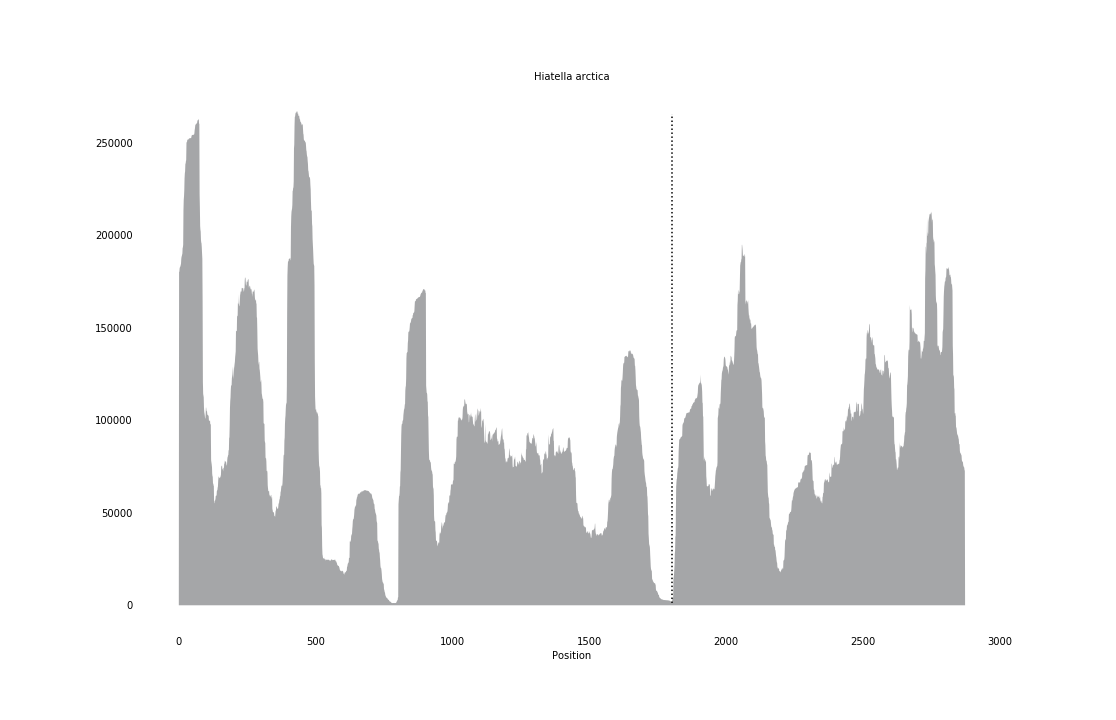

Supplement: Supplementary file 2 — Supplementary information [file 41598_2019_55573_MOESM2_ESM.zip › SupplementaryFile1/Metazoa/Protostomia/Mollusca/Hiatella_arctica_coverage.png]

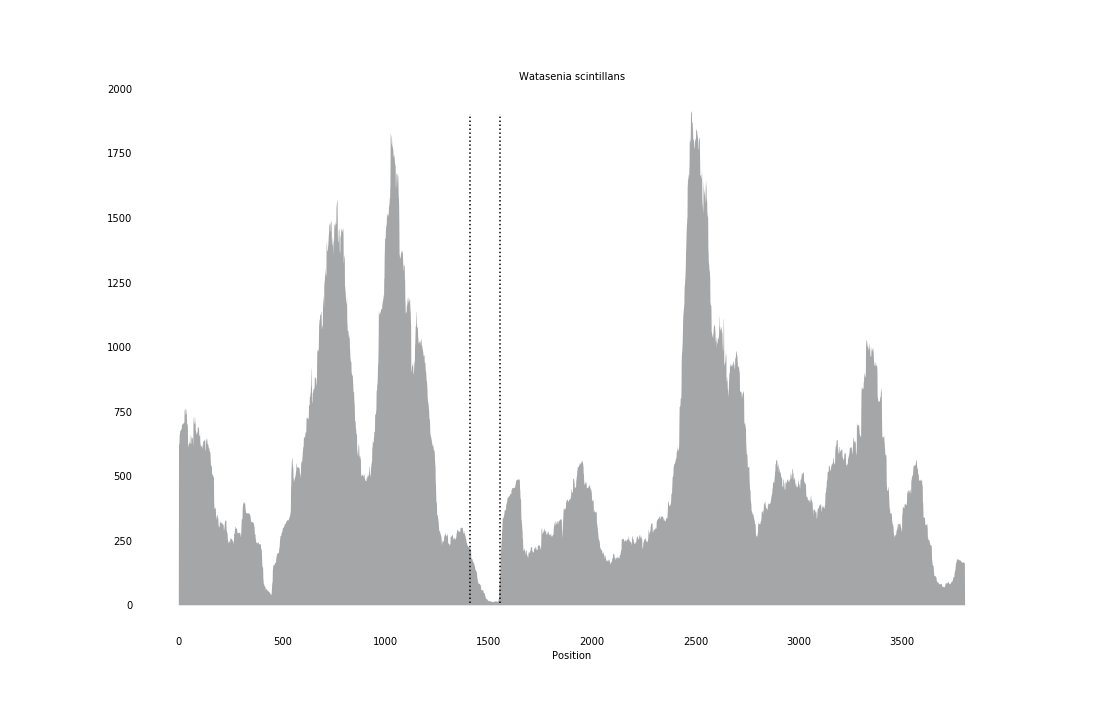

Supplement: Supplementary file 2 — Supplementary information [file 41598_2019_55573_MOESM2_ESM.zip › SupplementaryFile1/Metazoa/Protostomia/Mollusca/Watasenia_scintillans_coverage_correct.png]

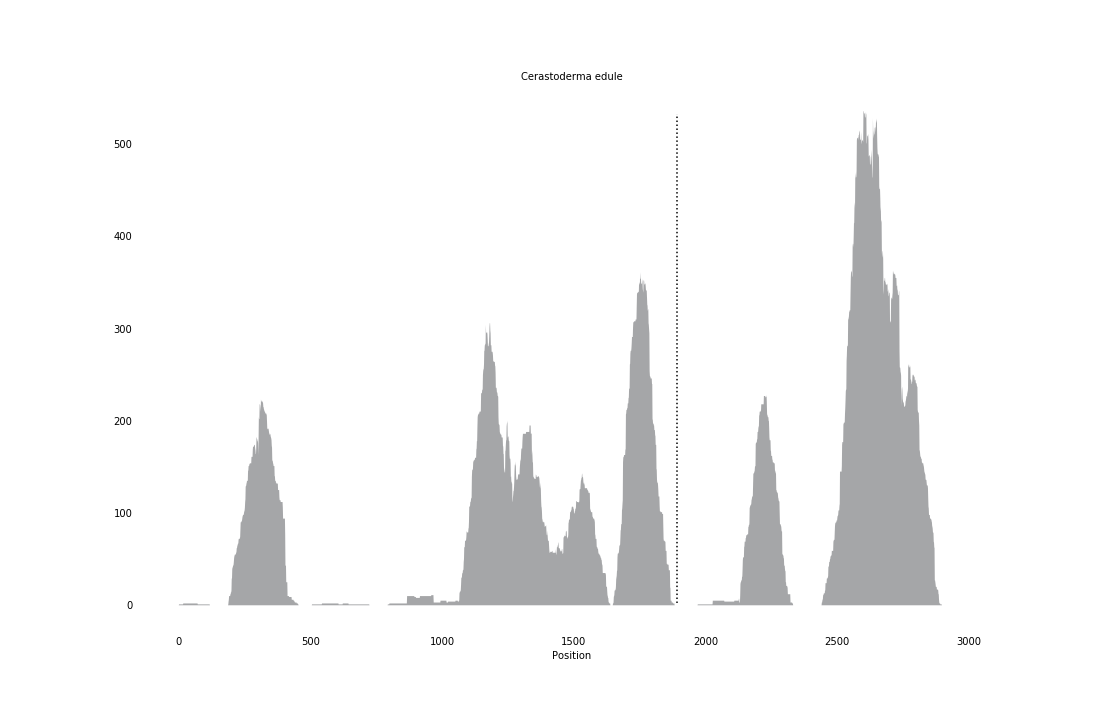

Supplement: Supplementary file 2 — Supplementary information [file 41598_2019_55573_MOESM2_ESM.zip › SupplementaryFile1/Metazoa/Protostomia/Mollusca/Cerastoderma_edule_coverage.png]

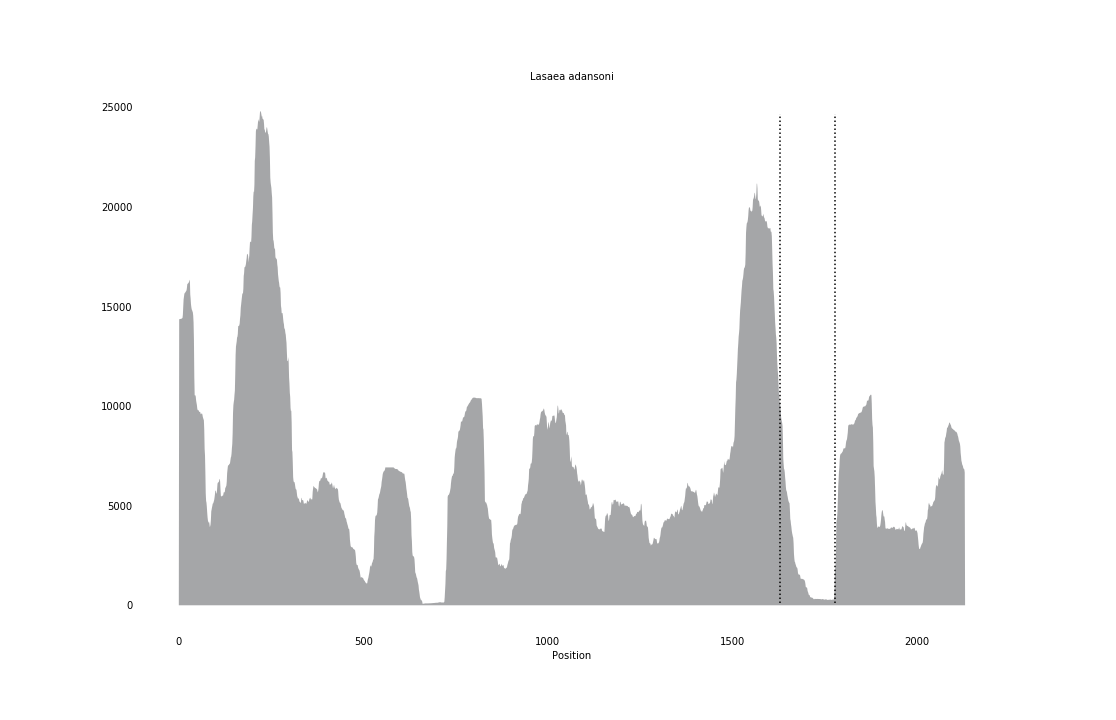

Supplement: Supplementary file 2 — Supplementary information [file 41598_2019_55573_MOESM2_ESM.zip › SupplementaryFile1/Metazoa/Protostomia/Mollusca/Lasaea_adansoni_coverage_correct.png]

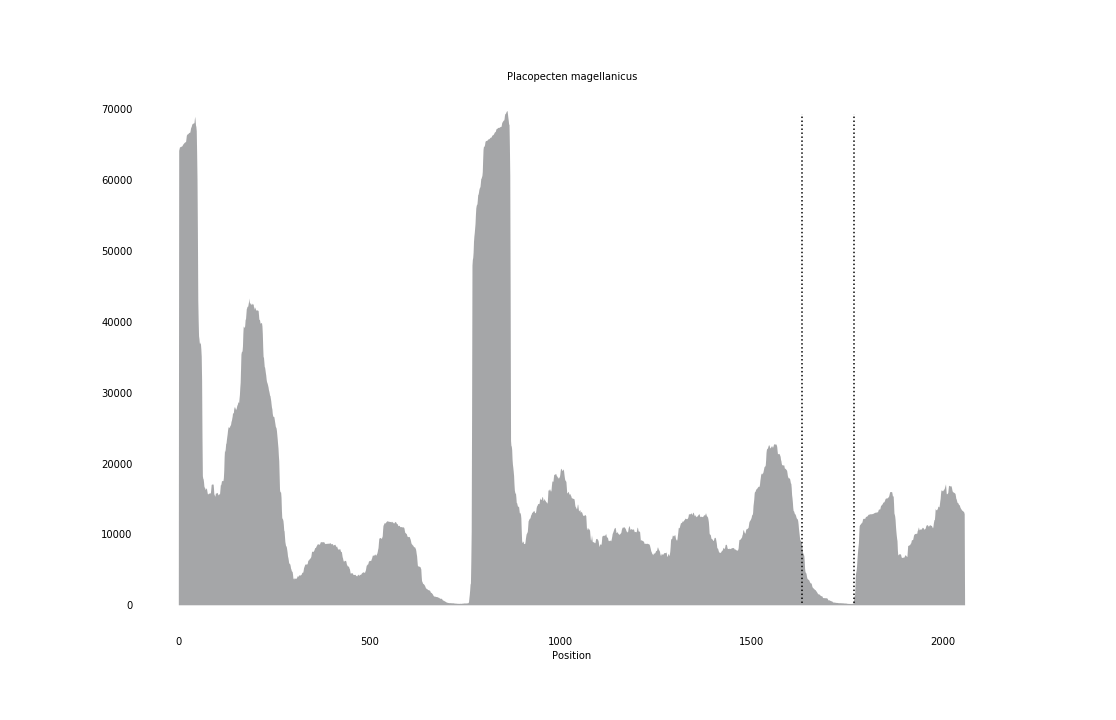

Supplement: Supplementary file 2 — Supplementary information [file 41598_2019_55573_MOESM2_ESM.zip › SupplementaryFile1/Metazoa/Protostomia/Mollusca/Placopecten_magellanicus_coverage_correct.png]

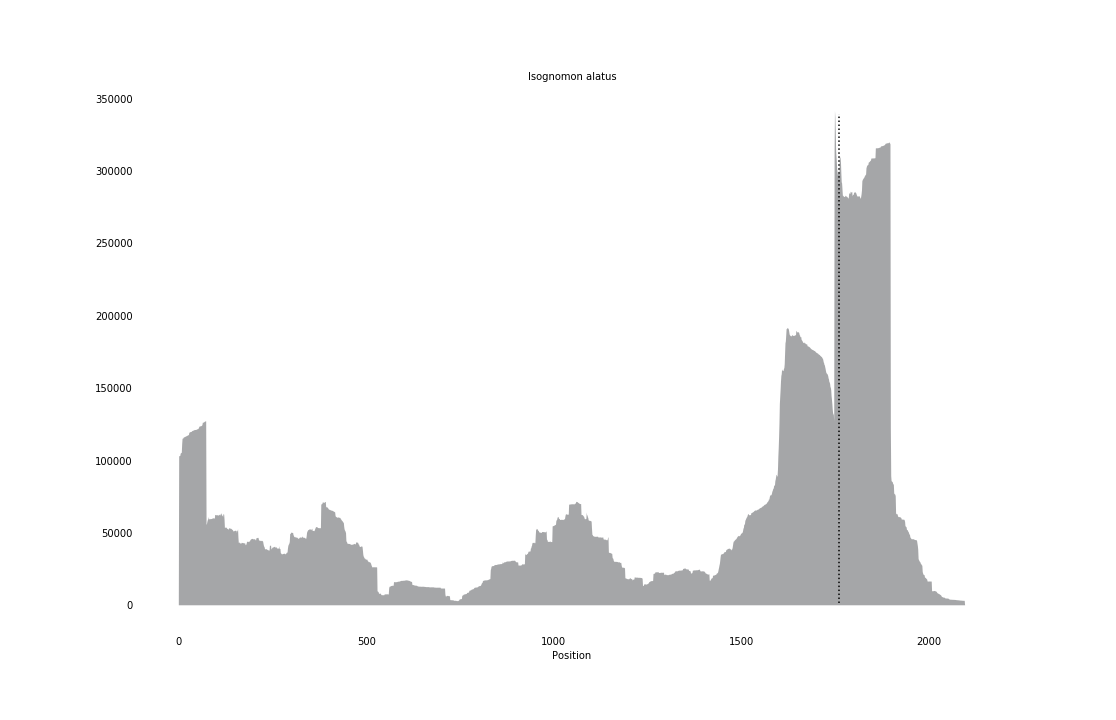

Supplement: Supplementary file 2 — Supplementary information [file 41598_2019_55573_MOESM2_ESM.zip › SupplementaryFile1/Metazoa/Protostomia/Mollusca/Isognomon_alatus_coverage.png]

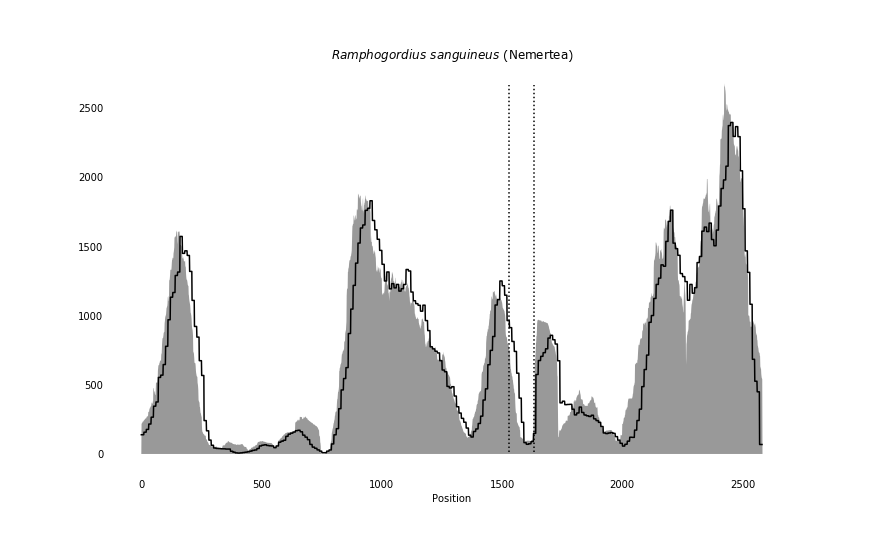

Supplement: Supplementary file 2 — Supplementary information [file 41598_2019_55573_MOESM2_ESM.zip › SupplementaryFile1/Metazoa/Protostomia/Ramphogordius_sanguineus_coverage.png]

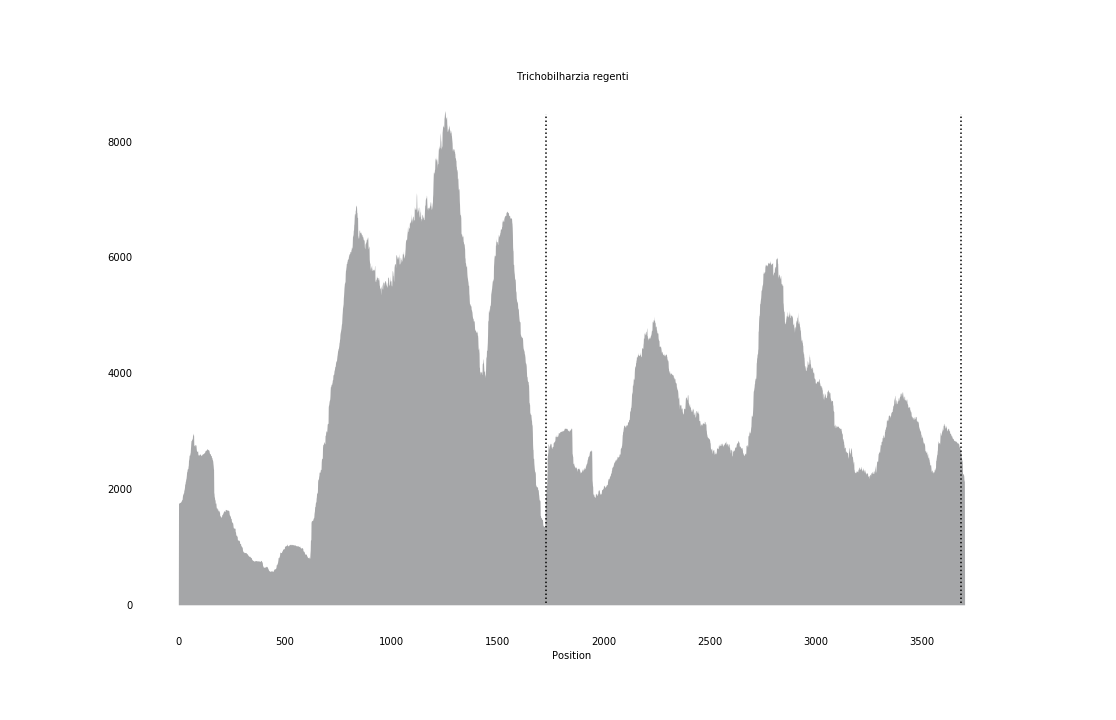

Supplement: Supplementary file 2 — Supplementary information [file 41598_2019_55573_MOESM2_ESM.zip › SupplementaryFile1/Metazoa/Protostomia/Platyhelminthes/Trichobilharzia_regenti_coverage_correct.png]

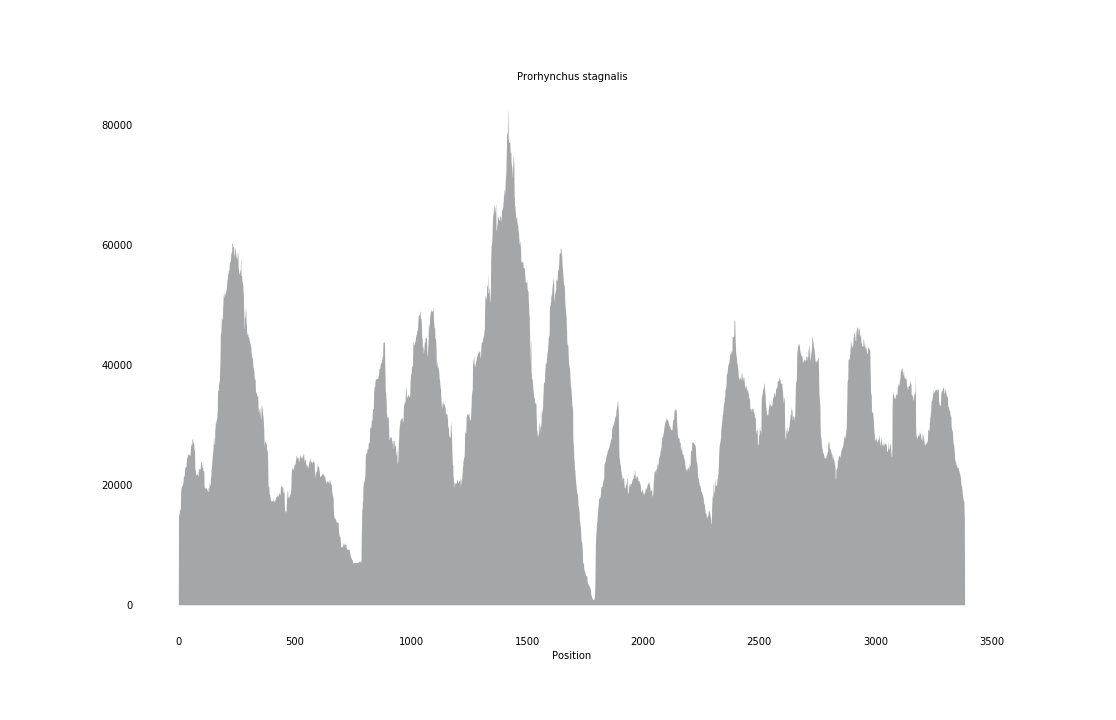

Supplement: Supplementary file 2 — Supplementary information [file 41598_2019_55573_MOESM2_ESM.zip › SupplementaryFile1/Metazoa/Protostomia/Platyhelminthes/Prorhynchus_stagnalis_coverage.png]

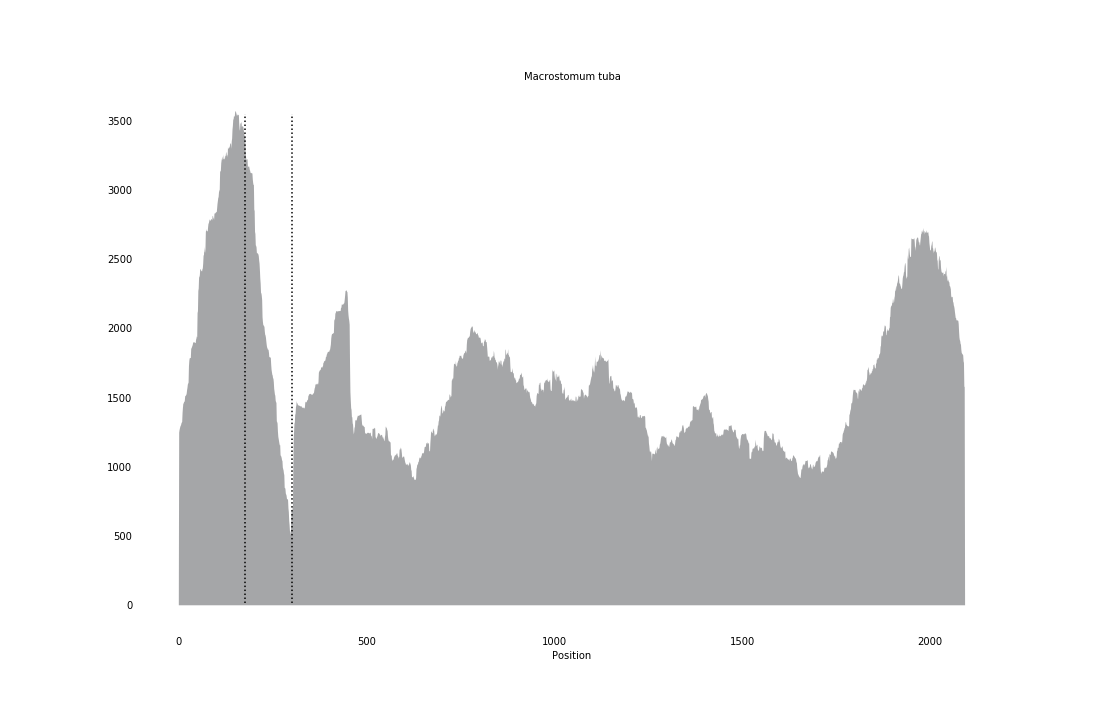

Supplement: Supplementary file 2 — Supplementary information [file 41598_2019_55573_MOESM2_ESM.zip › SupplementaryFile1/Metazoa/Protostomia/Platyhelminthes/Macrostomum_tuba_coverage_correct.png]

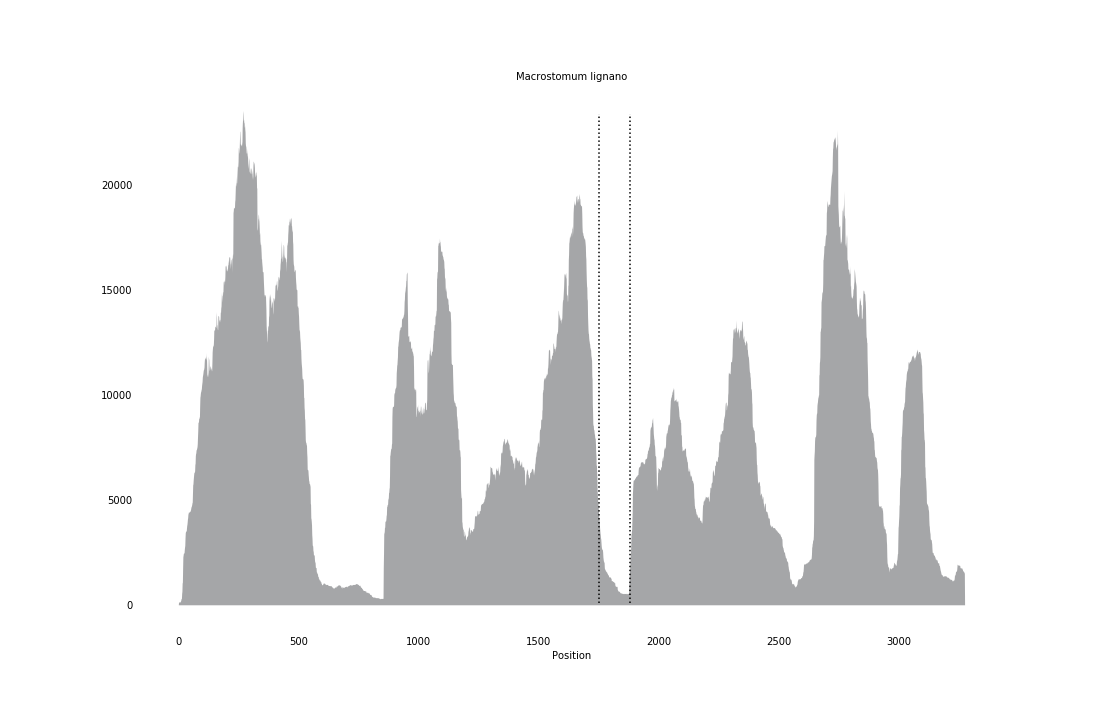

Supplement: Supplementary file 2 — Supplementary information [file 41598_2019_55573_MOESM2_ESM.zip › SupplementaryFile1/Metazoa/Protostomia/Platyhelminthes/Macrostomum_lignano_coverage_correct.png]

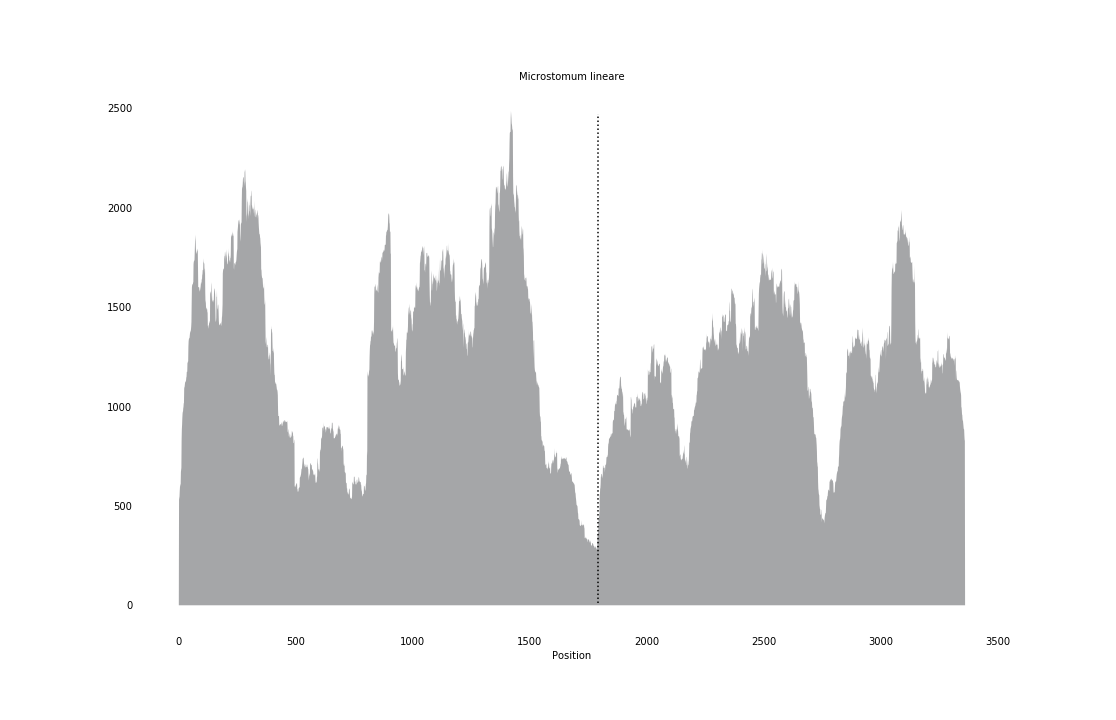

Supplement: Supplementary file 2 — Supplementary information [file 41598_2019_55573_MOESM2_ESM.zip › SupplementaryFile1/Metazoa/Protostomia/Platyhelminthes/Microstomum_lineare_coverage.png]

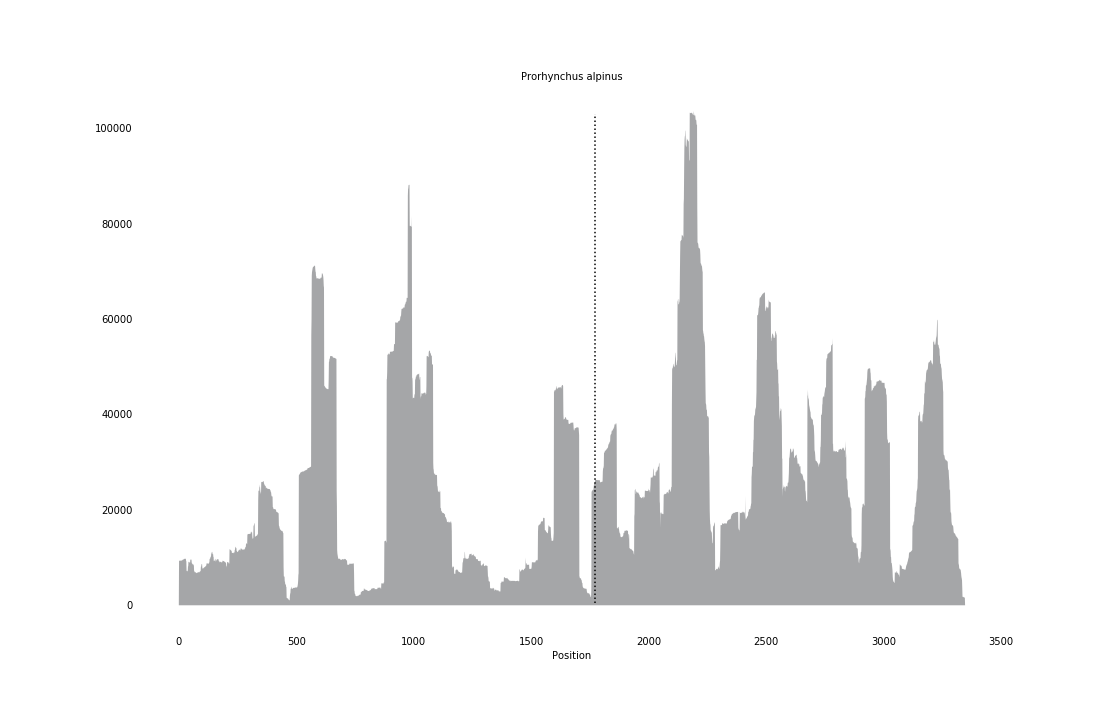

Supplement: Supplementary file 2 — Supplementary information [file 41598_2019_55573_MOESM2_ESM.zip › SupplementaryFile1/Metazoa/Protostomia/Platyhelminthes/Prorhynchus_alpinus_coverage.png]

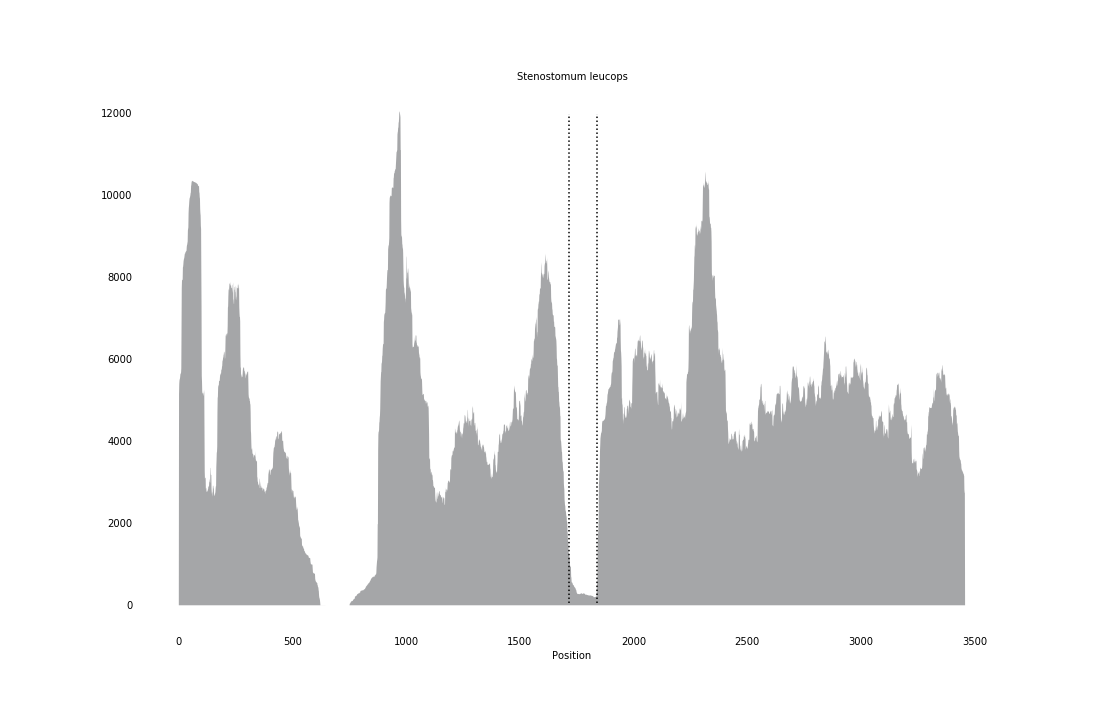

Supplement: Supplementary file 2 — Supplementary information [file 41598_2019_55573_MOESM2_ESM.zip › SupplementaryFile1/Metazoa/Protostomia/Platyhelminthes/Stenostomum_leucops_coverage_correct.png]

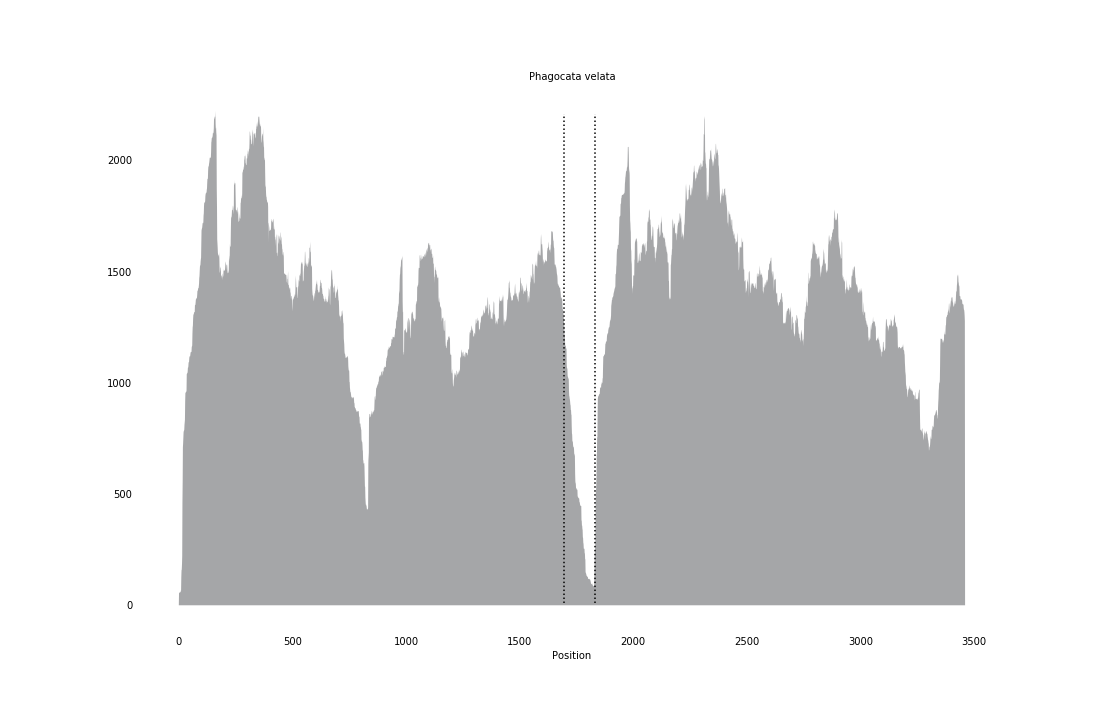

Supplement: Supplementary file 2 — Supplementary information [file 41598_2019_55573_MOESM2_ESM.zip › SupplementaryFile1/Metazoa/Protostomia/Platyhelminthes/Phagocata_velata_coverage_correct.png]

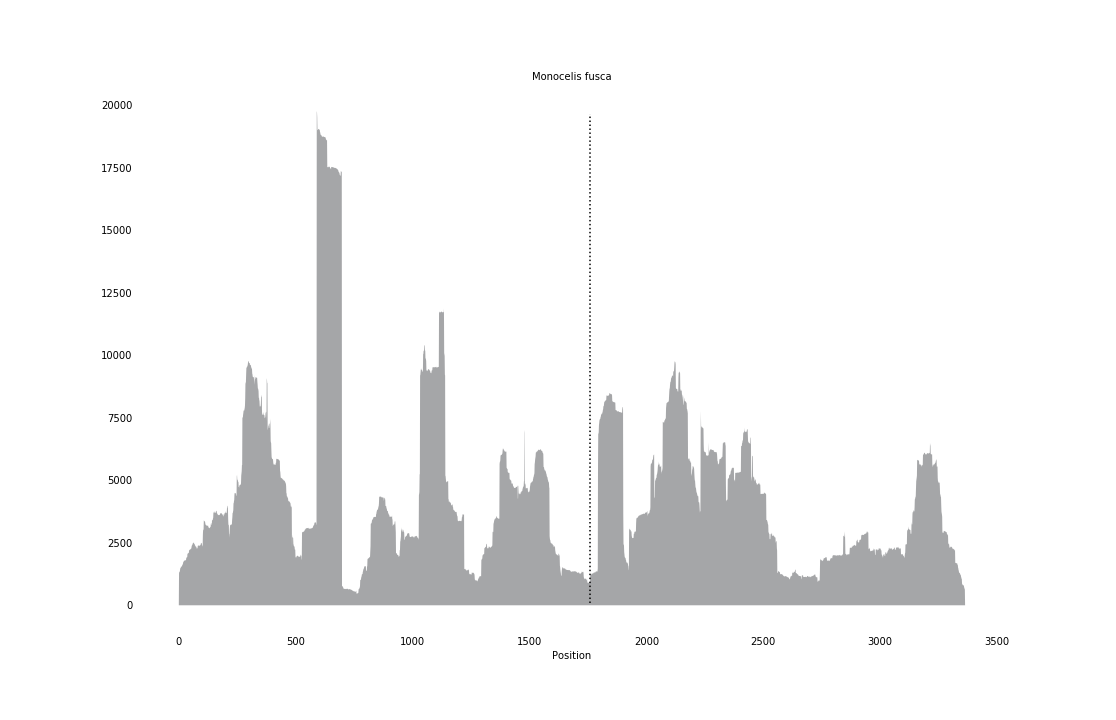

Supplement: Supplementary file 2 — Supplementary information [file 41598_2019_55573_MOESM2_ESM.zip › SupplementaryFile1/Metazoa/Protostomia/Platyhelminthes/Monocelis_fusca_coverage.png]

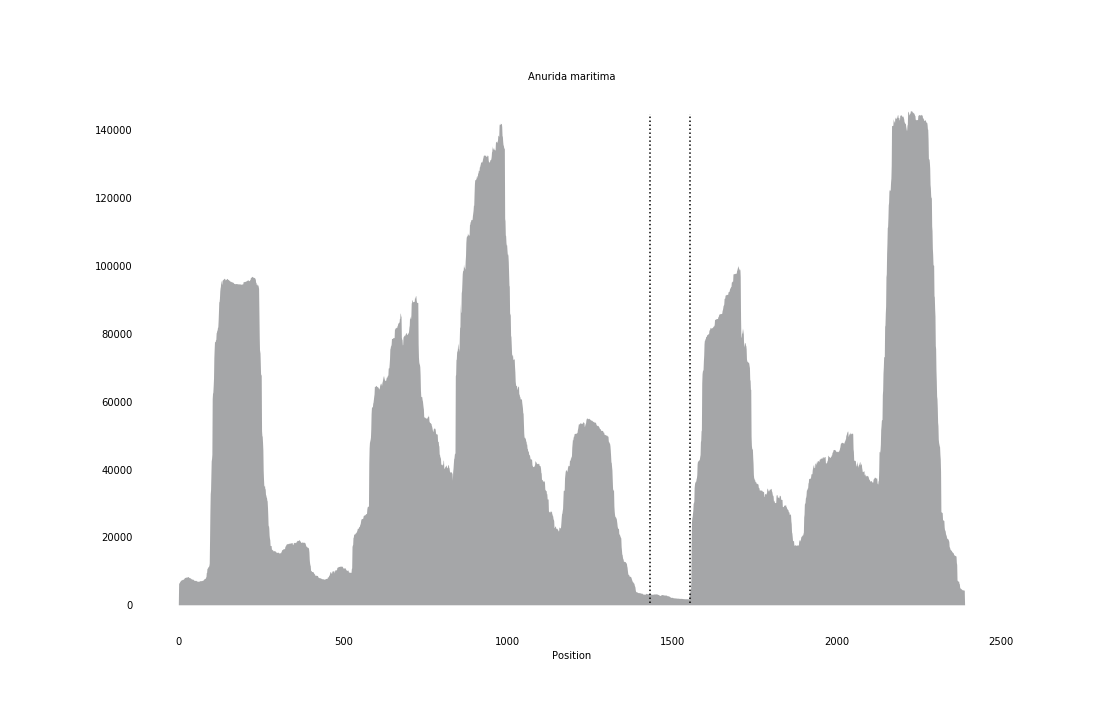

Supplement: Supplementary file 2 — Supplementary information [file 41598_2019_55573_MOESM2_ESM.zip › SupplementaryFile1/Metazoa/Protostomia/Arthropoda/Collembola/Anurida_maritima_coverage_correct.png]

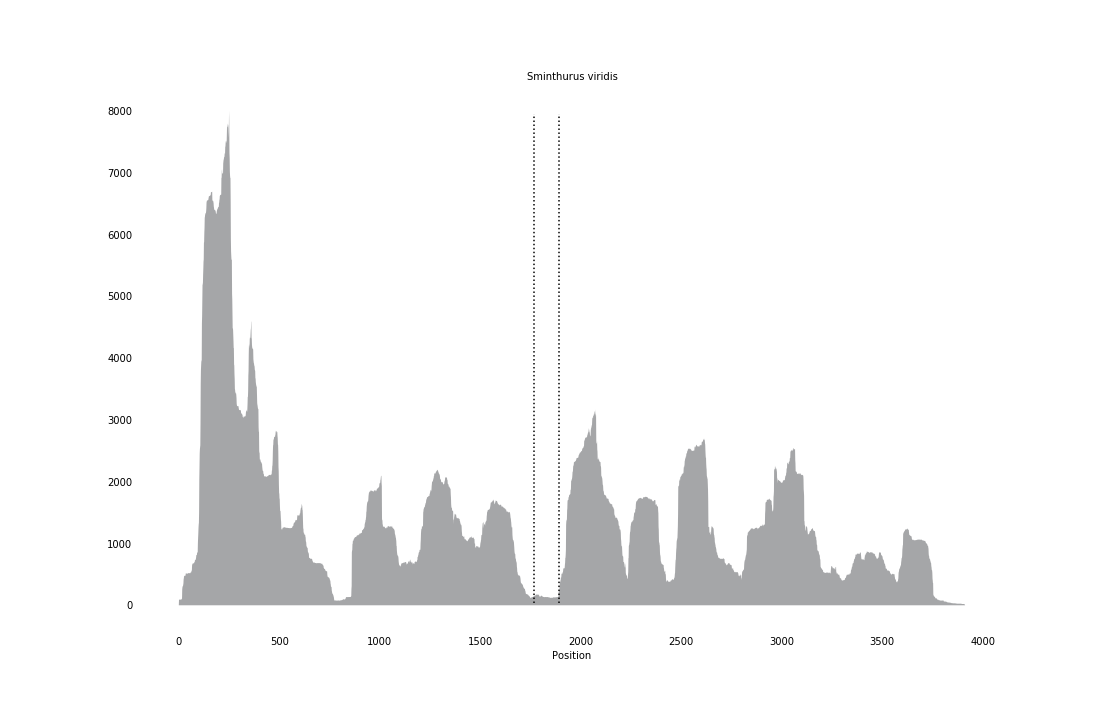

Supplement: Supplementary file 2 — Supplementary information [file 41598_2019_55573_MOESM2_ESM.zip › SupplementaryFile1/Metazoa/Protostomia/Arthropoda/Collembola/Sminthurus_viridis_coverage_correct.png]

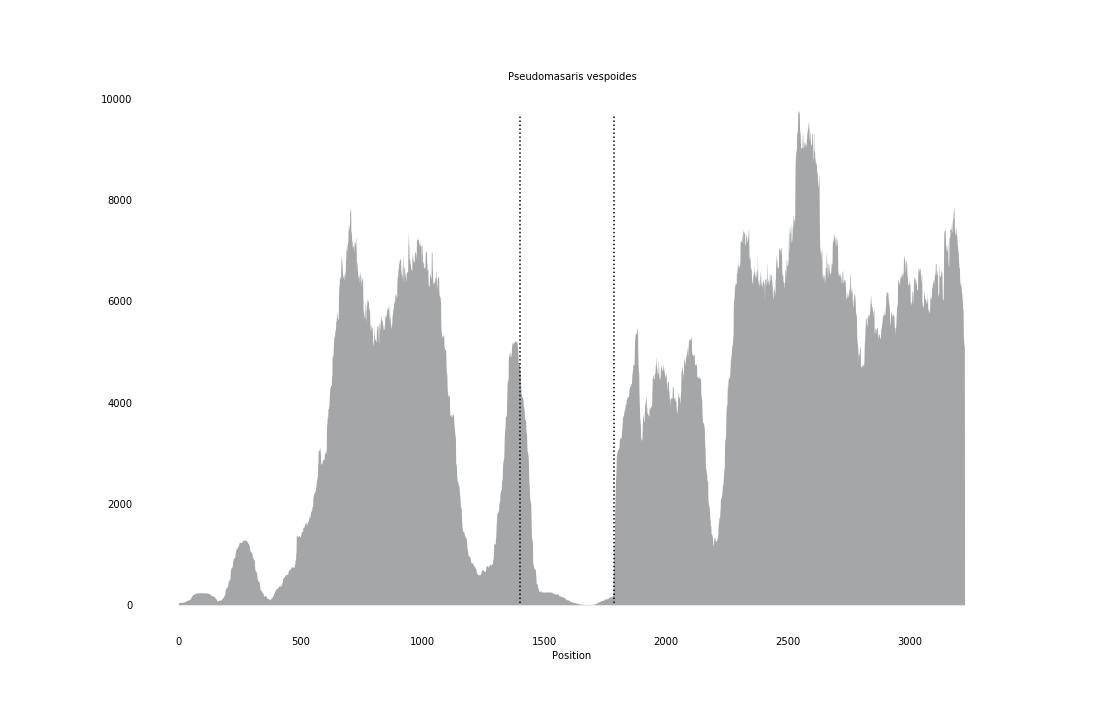

Supplement: Supplementary file 2 — Supplementary information [file 41598_2019_55573_MOESM2_ESM.zip › SupplementaryFile1/Metazoa/Protostomia/Arthropoda/Insecta/Pseudomasaris_vespoides_coverage_correct.png]

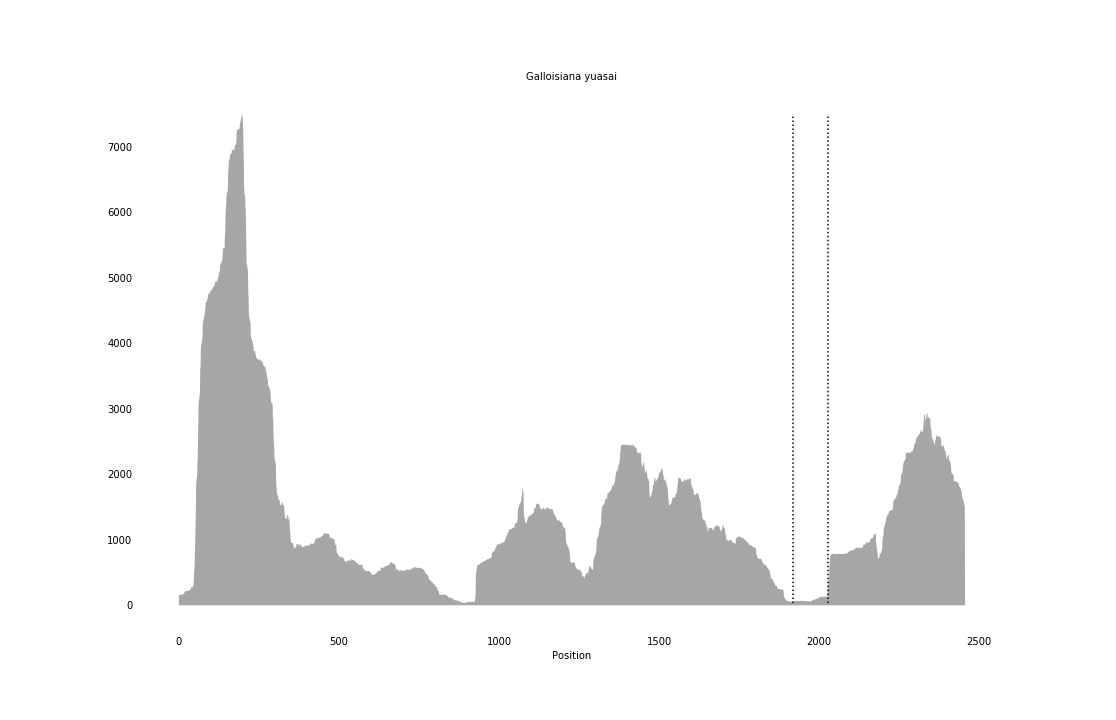

Supplement: Supplementary file 2 — Supplementary information [file 41598_2019_55573_MOESM2_ESM.zip › SupplementaryFile1/Metazoa/Protostomia/Arthropoda/Insecta/Galloisiana_yuasai_coverage_correct.png]

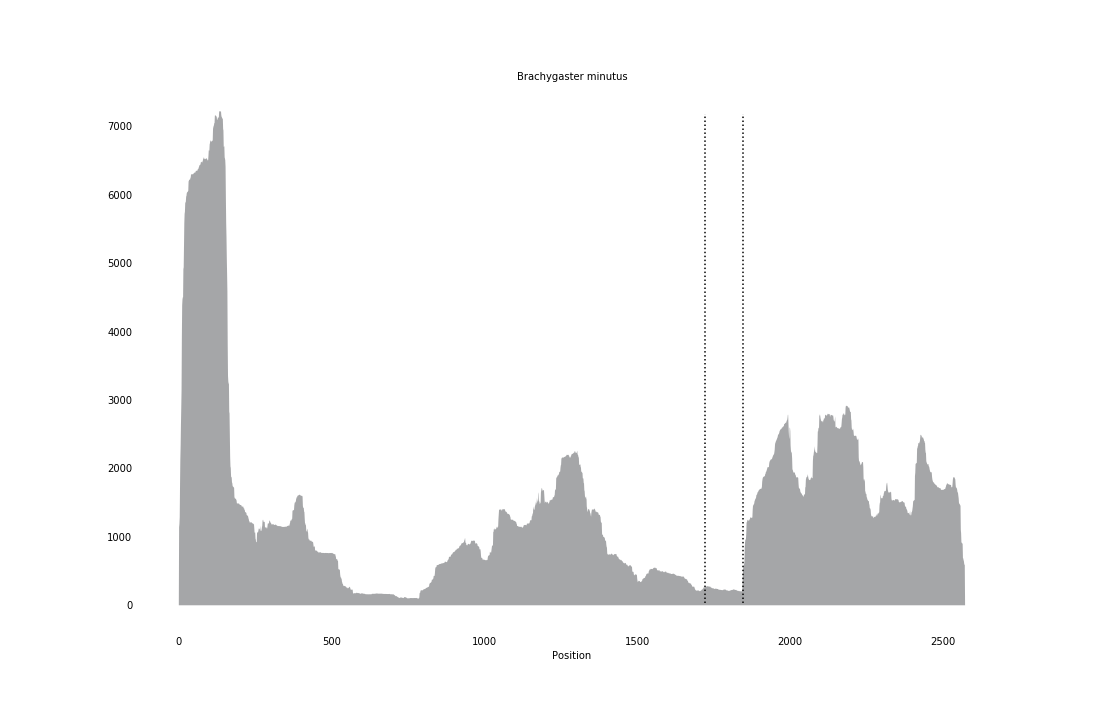

Supplement: Supplementary file 2 — Supplementary information [file 41598_2019_55573_MOESM2_ESM.zip › SupplementaryFile1/Metazoa/Protostomia/Arthropoda/Insecta/Brachygaster_minutus_coverage_correct.png]

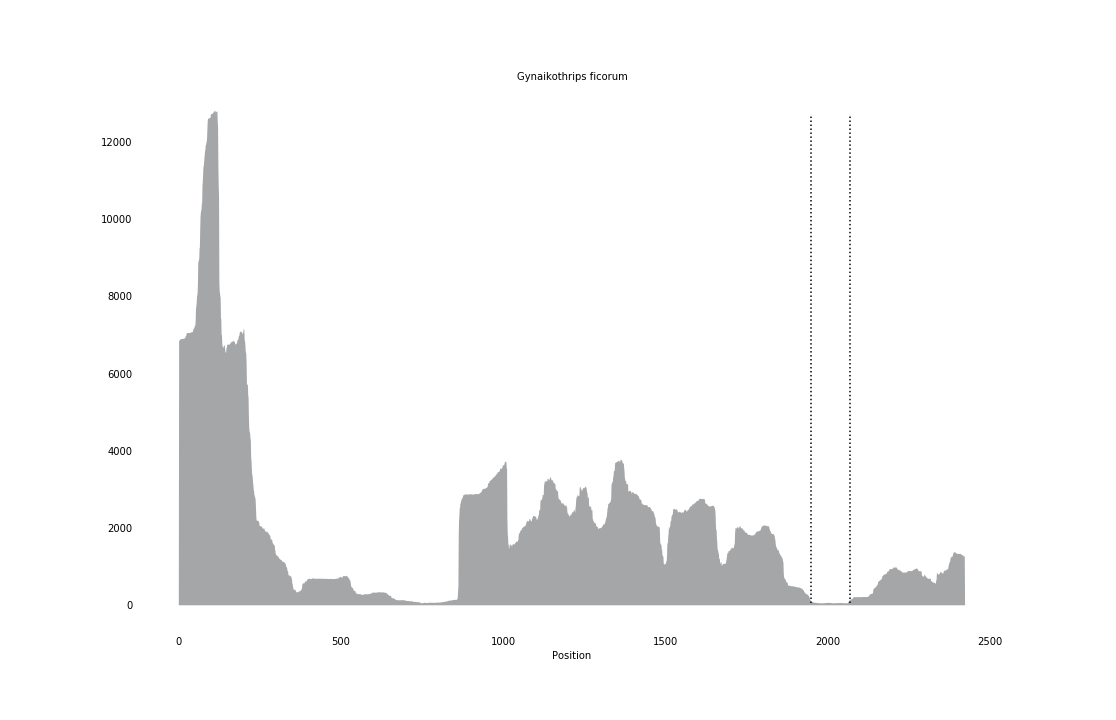

Supplement: Supplementary file 2 — Supplementary information [file 41598_2019_55573_MOESM2_ESM.zip › SupplementaryFile1/Metazoa/Protostomia/Arthropoda/Insecta/Gynaikothrips_ficorum_coverage_correct.png]

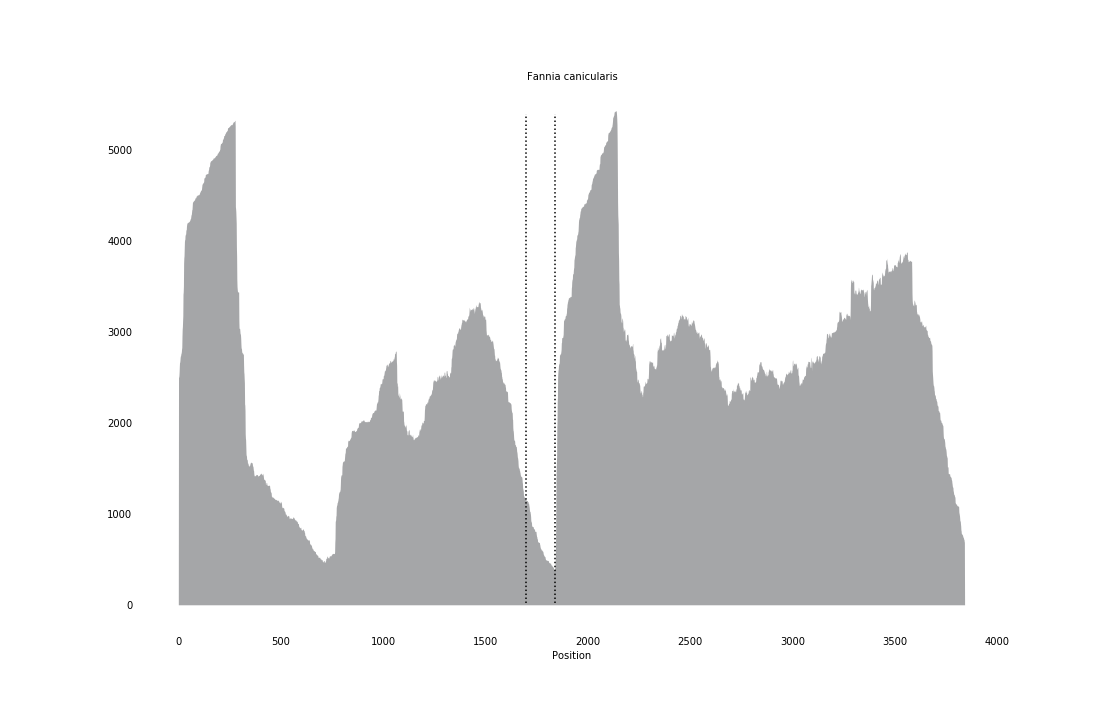

Supplement: Supplementary file 2 — Supplementary information [file 41598_2019_55573_MOESM2_ESM.zip › SupplementaryFile1/Metazoa/Protostomia/Arthropoda/Insecta/Fannia_canicularis_coverage_correct.png]

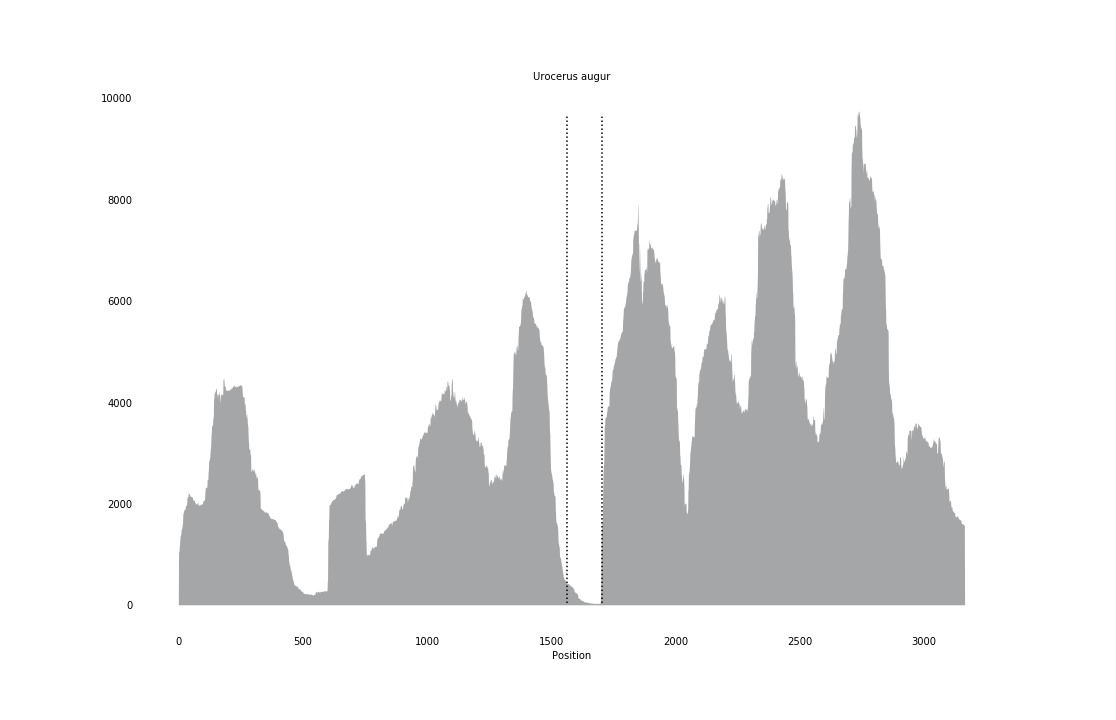

Supplement: Supplementary file 2 — Supplementary information [file 41598_2019_55573_MOESM2_ESM.zip › SupplementaryFile1/Metazoa/Protostomia/Arthropoda/Insecta/Urocerus_augur_coverage_correct.png]

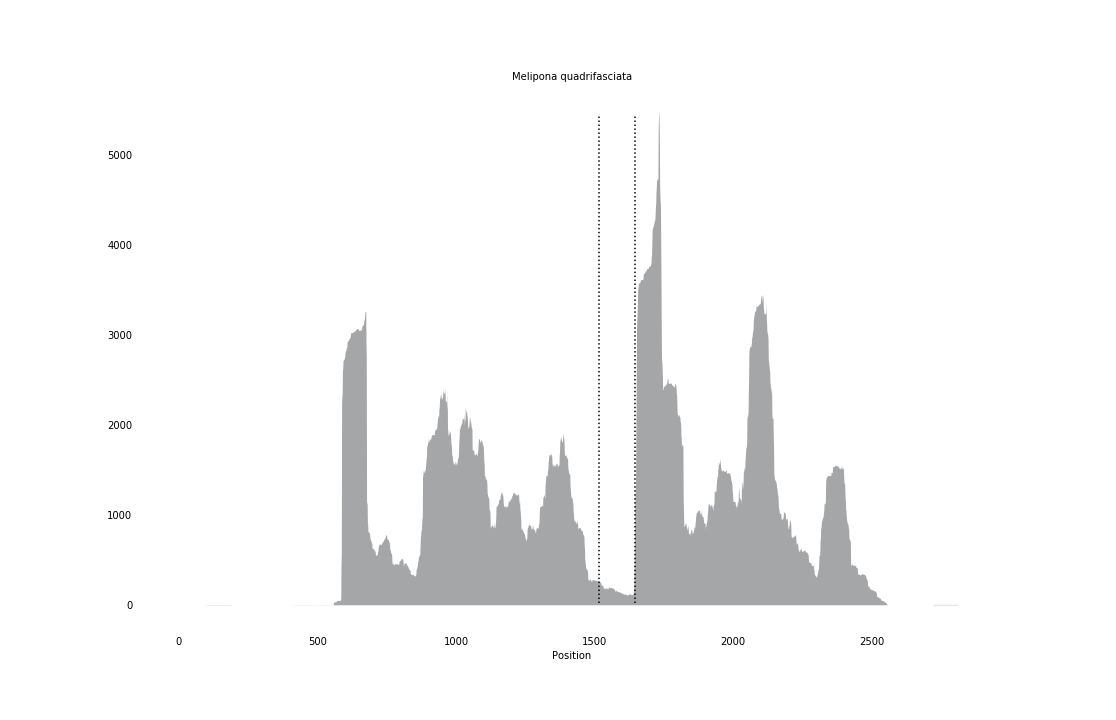

Supplement: Supplementary file 2 — Supplementary information [file 41598_2019_55573_MOESM2_ESM.zip › SupplementaryFile1/Metazoa/Protostomia/Arthropoda/Insecta/Melipona_quadrifasciata_coverage_correct.png]

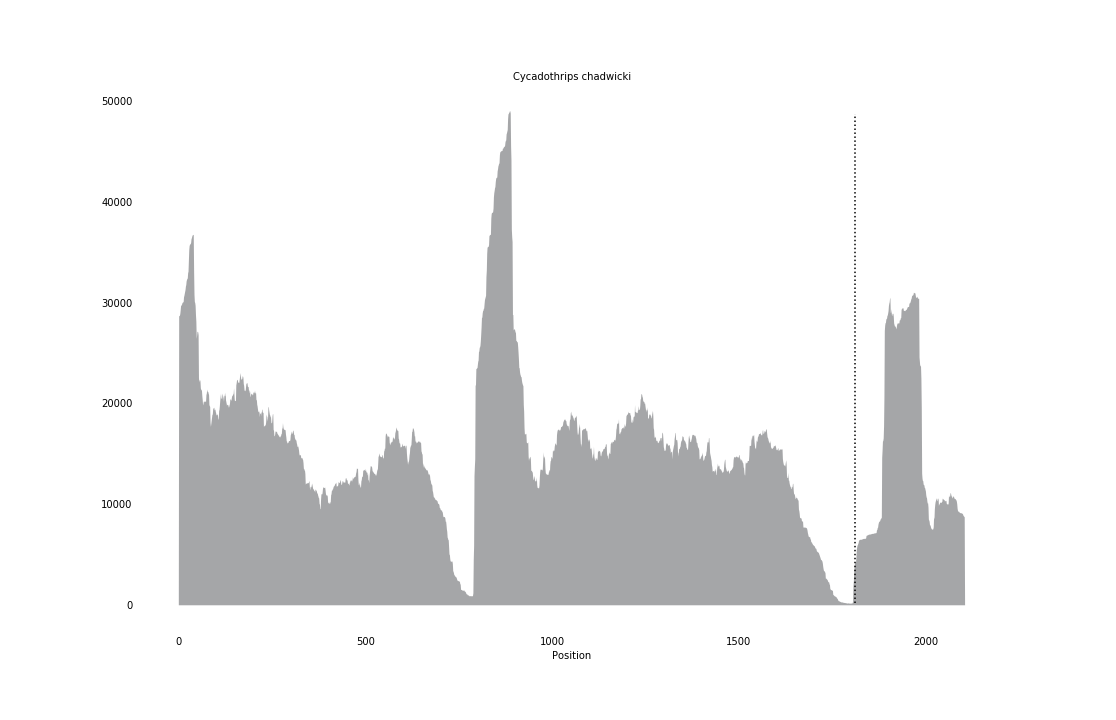

Supplement: Supplementary file 2 — Supplementary information [file 41598_2019_55573_MOESM2_ESM.zip › SupplementaryFile1/Metazoa/Protostomia/Arthropoda/Insecta/Cycadothrips_chadwicki_coverage.png]

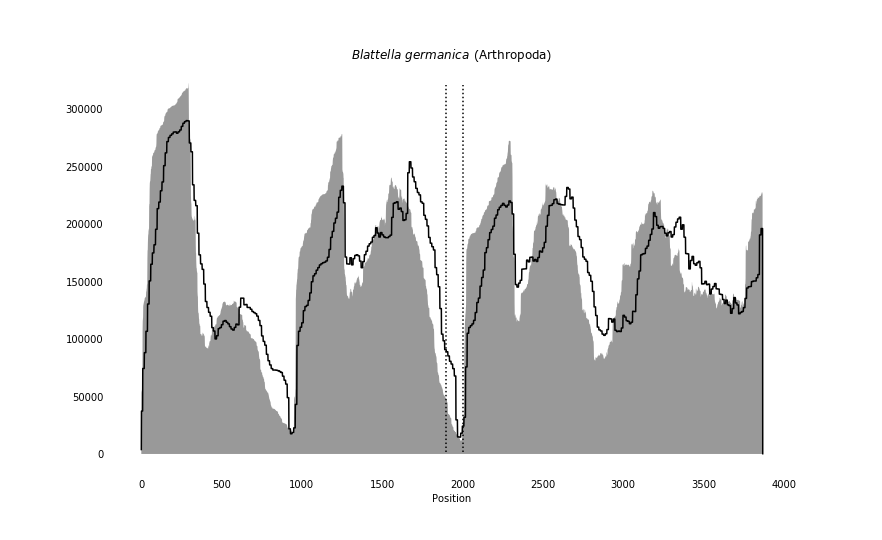

Supplement: Supplementary file 2 — Supplementary information [file 41598_2019_55573_MOESM2_ESM.zip › SupplementaryFile1/Metazoa/Protostomia/Arthropoda/Insecta/Blattella_germanica_overage.png]

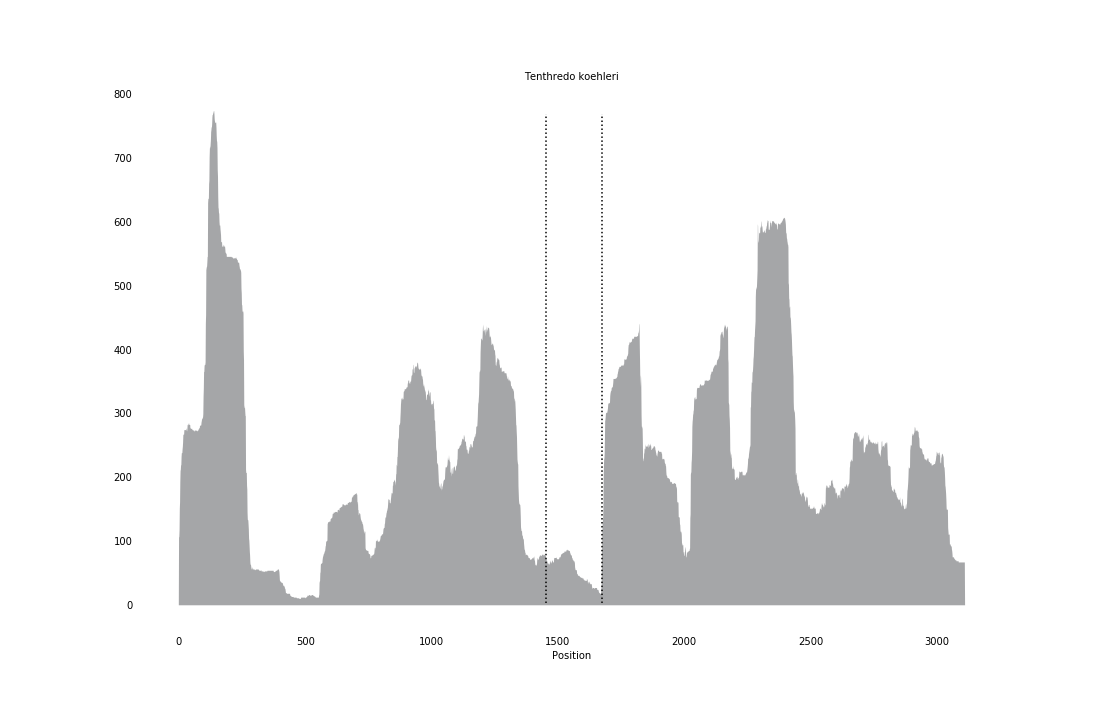

Supplement: Supplementary file 2 — Supplementary information [file 41598_2019_55573_MOESM2_ESM.zip › SupplementaryFile1/Metazoa/Protostomia/Arthropoda/Insecta/Tenthredo_koehleri_coverage_correct.png]

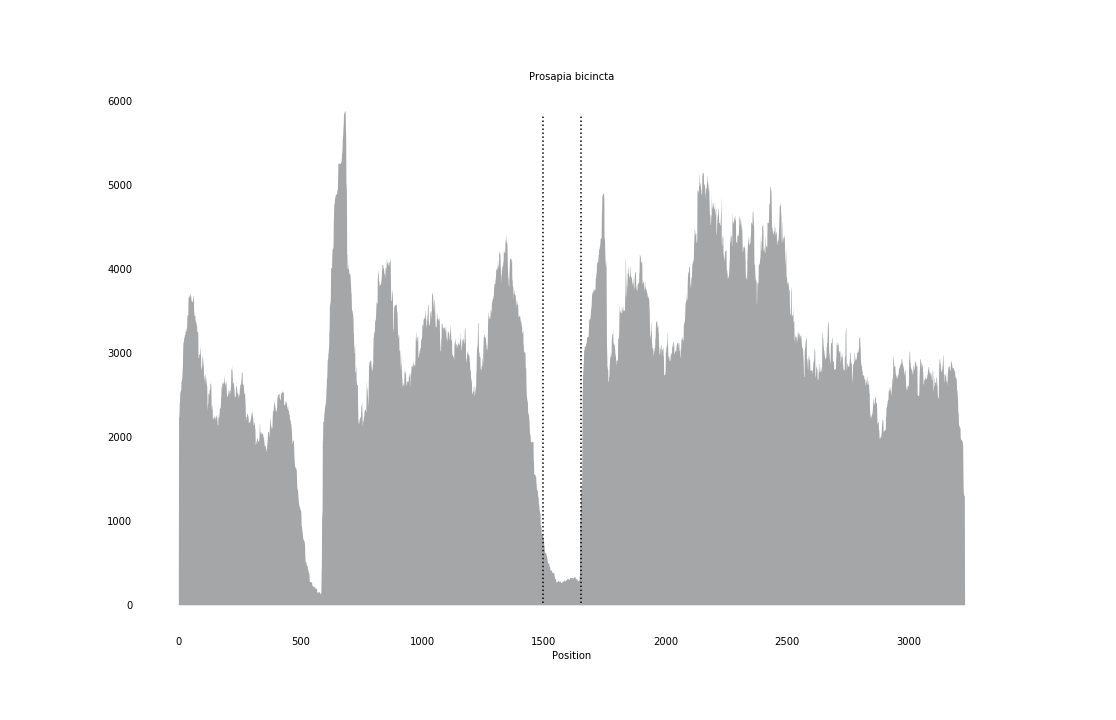

Supplement: Supplementary file 2 — Supplementary information [file 41598_2019_55573_MOESM2_ESM.zip › SupplementaryFile1/Metazoa/Protostomia/Arthropoda/Insecta/Prosapia_bicincta_coverage_correct.png]

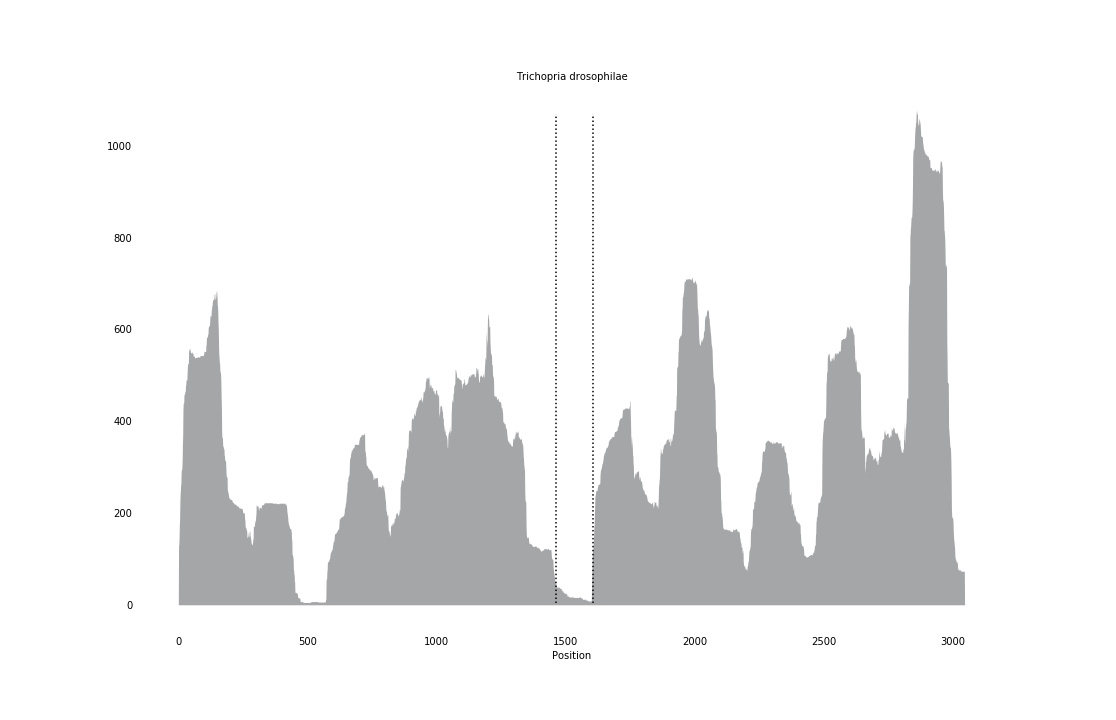

Supplement: Supplementary file 2 — Supplementary information [file 41598_2019_55573_MOESM2_ESM.zip › SupplementaryFile1/Metazoa/Protostomia/Arthropoda/Insecta/Trichopria_drosophilae_coverage_correct.png]

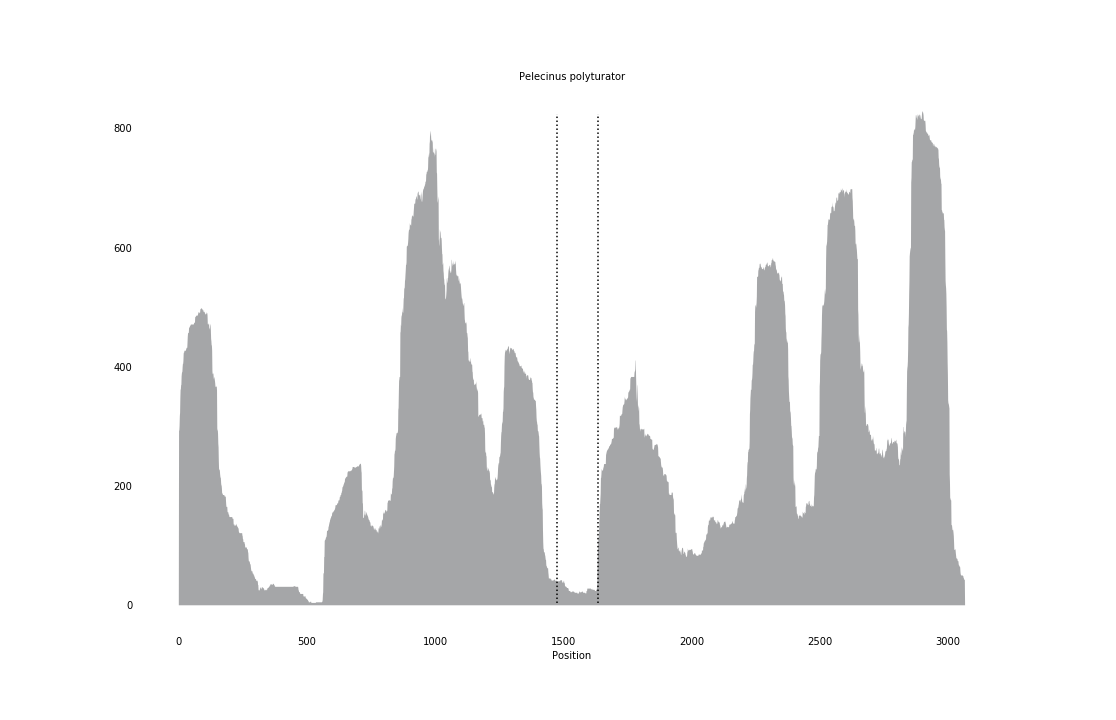

Supplement: Supplementary file 2 — Supplementary information [file 41598_2019_55573_MOESM2_ESM.zip › SupplementaryFile1/Metazoa/Protostomia/Arthropoda/Insecta/Pelecinus_polyturator_coverage_correct.png]

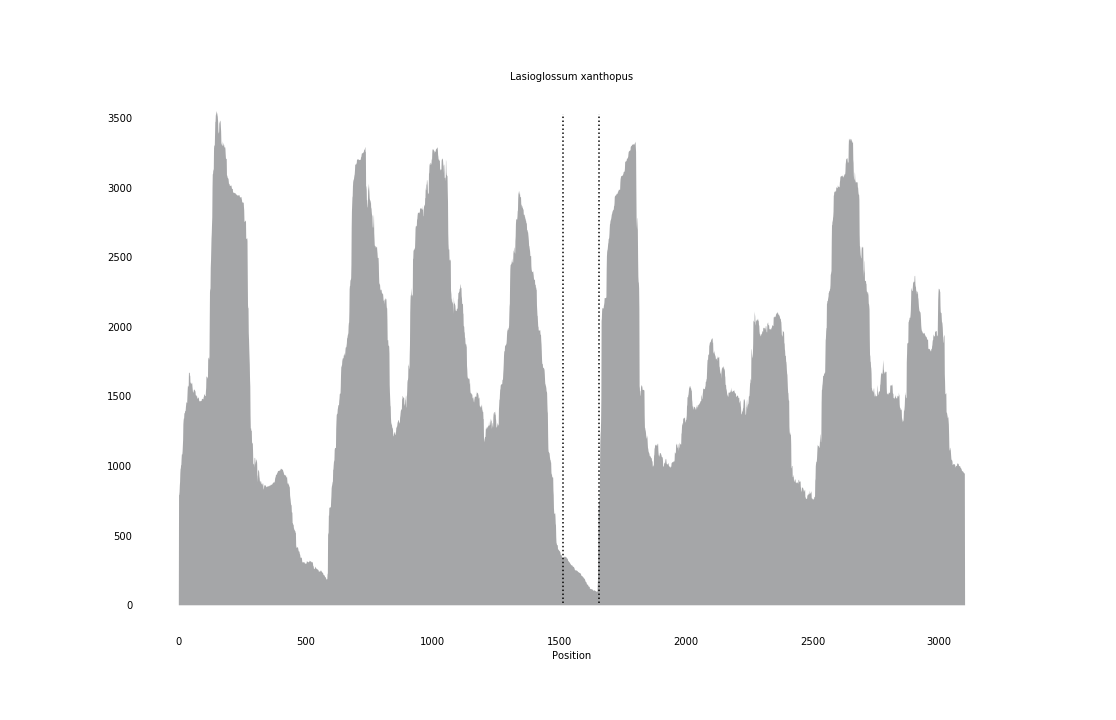

Supplement: Supplementary file 2 — Supplementary information [file 41598_2019_55573_MOESM2_ESM.zip › SupplementaryFile1/Metazoa/Protostomia/Arthropoda/Insecta/Lasioglossum_xanthopus_coverage_correct.png]

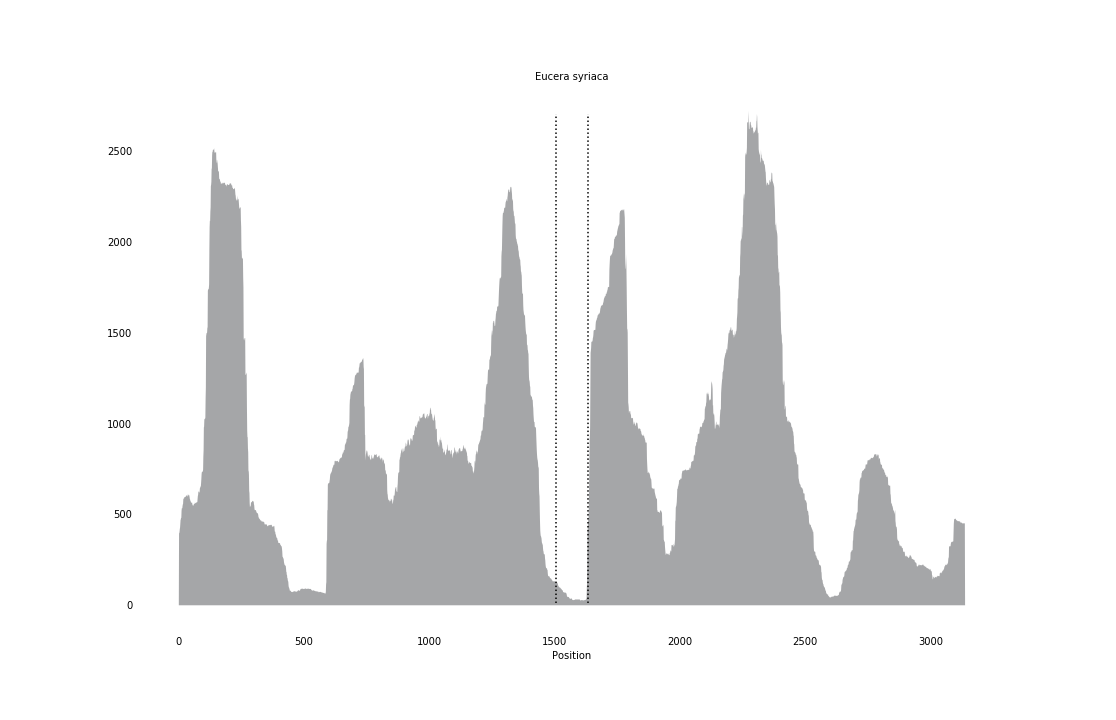

Supplement: Supplementary file 2 — Supplementary information [file 41598_2019_55573_MOESM2_ESM.zip › SupplementaryFile1/Metazoa/Protostomia/Arthropoda/Insecta/Eucera_syriaca_coverage_correct.png]

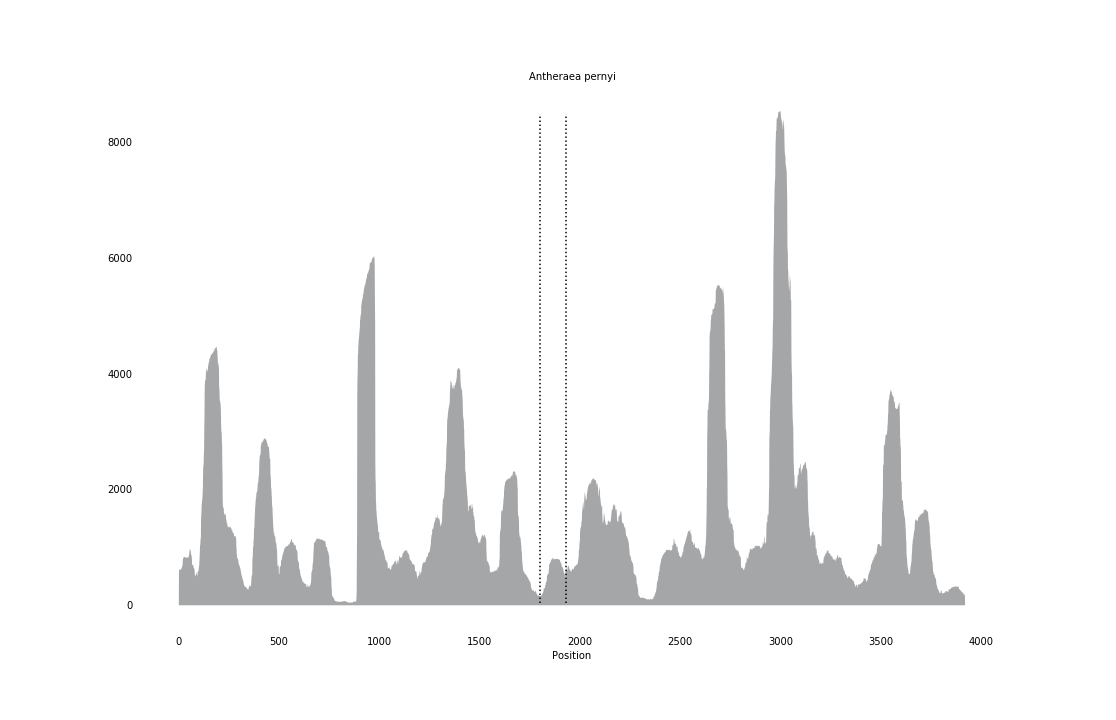

Supplement: Supplementary file 2 — Supplementary information [file 41598_2019_55573_MOESM2_ESM.zip › SupplementaryFile1/Metazoa/Protostomia/Arthropoda/Insecta/Antheraea_pernyi_coverage_correct.png]

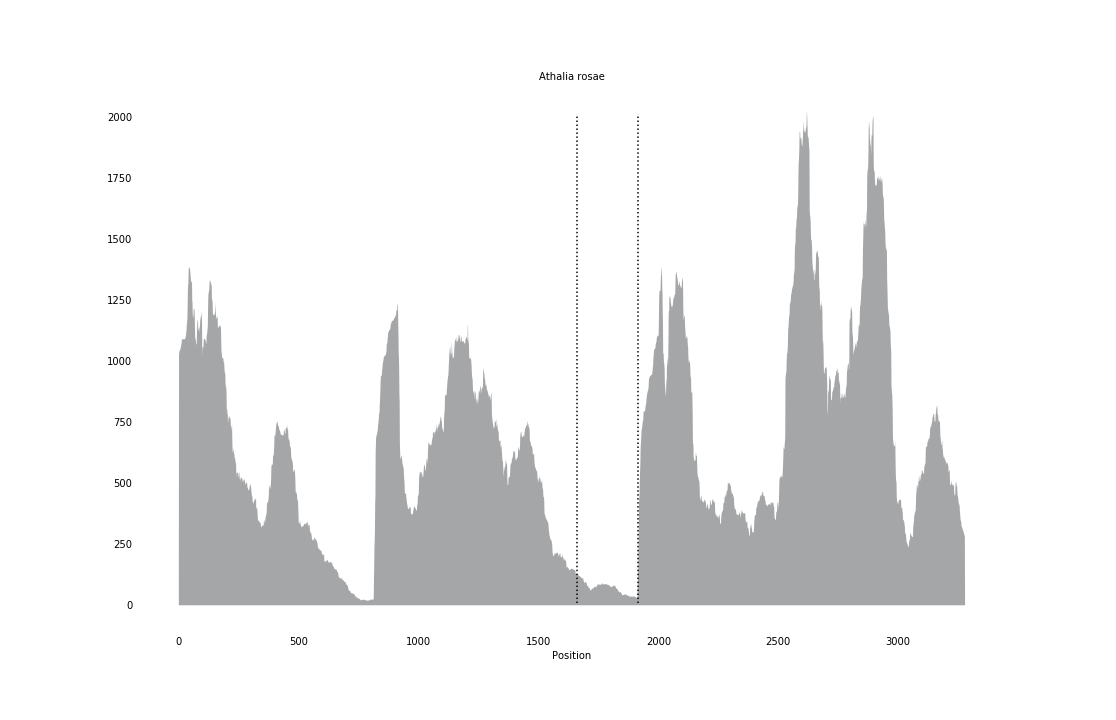

Supplement: Supplementary file 2 — Supplementary information [file 41598_2019_55573_MOESM2_ESM.zip › SupplementaryFile1/Metazoa/Protostomia/Arthropoda/Insecta/Athalia_rosae_coverage_correct.png]

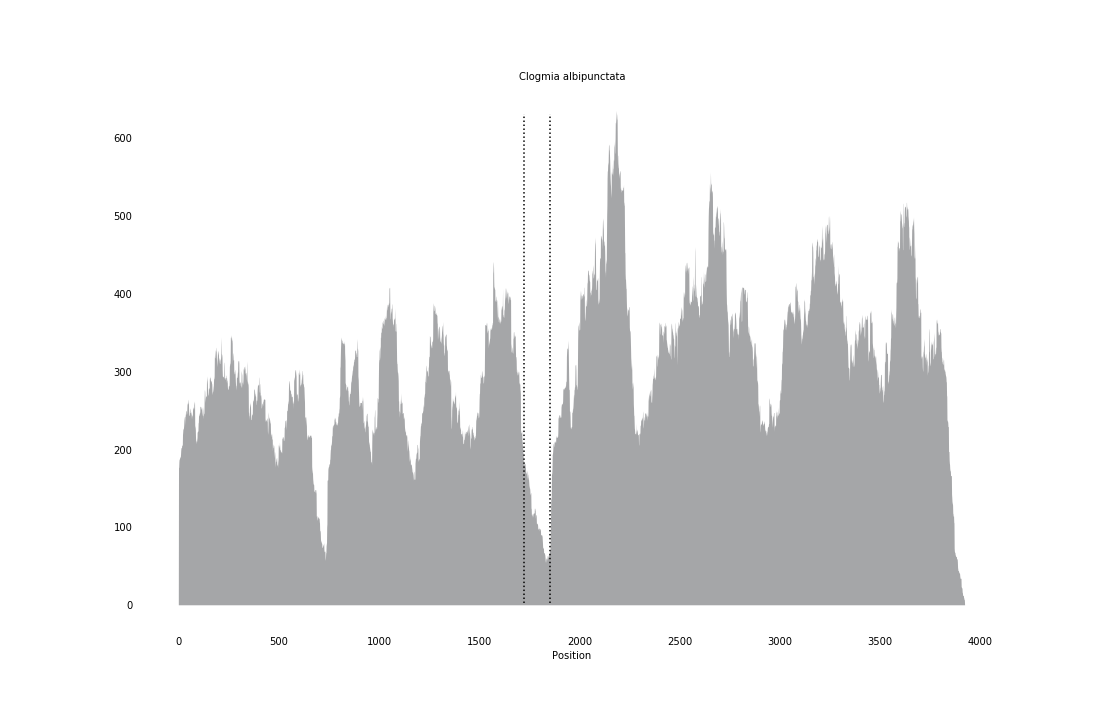

Supplement: Supplementary file 2 — Supplementary information [file 41598_2019_55573_MOESM2_ESM.zip › SupplementaryFile1/Metazoa/Protostomia/Arthropoda/Insecta/Clogmia_albipunctata_coverage_correct.png]

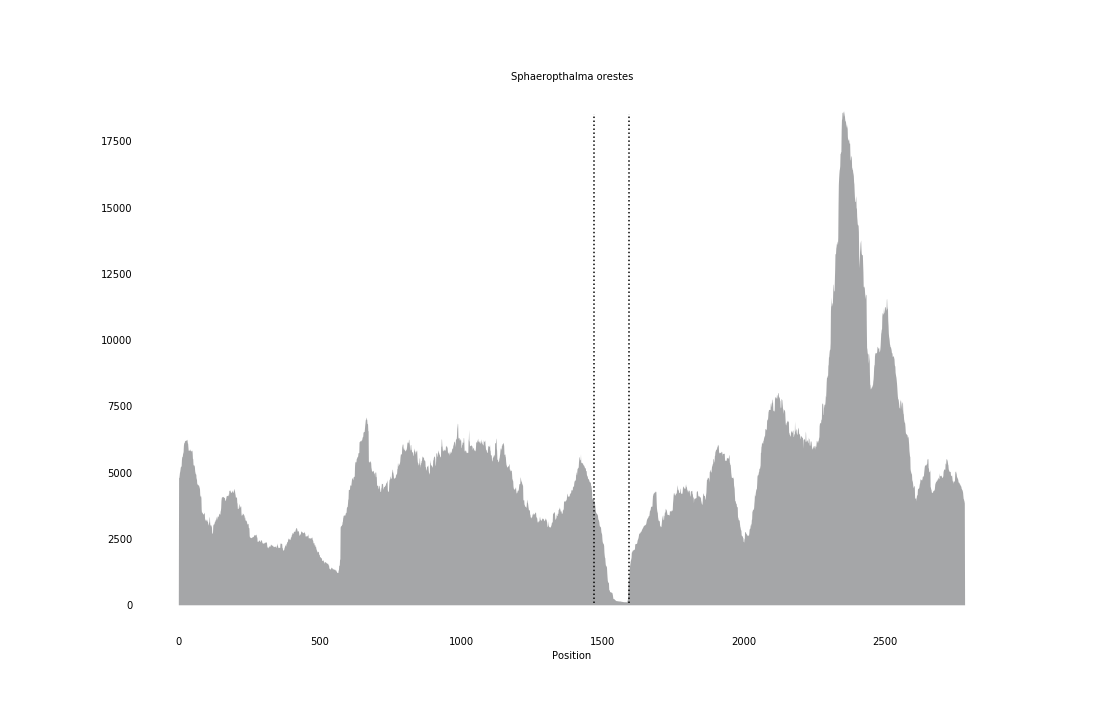

Supplement: Supplementary file 2 — Supplementary information [file 41598_2019_55573_MOESM2_ESM.zip › SupplementaryFile1/Metazoa/Protostomia/Arthropoda/Insecta/Sphaeropthalma_orestes_coverage_correct.png]

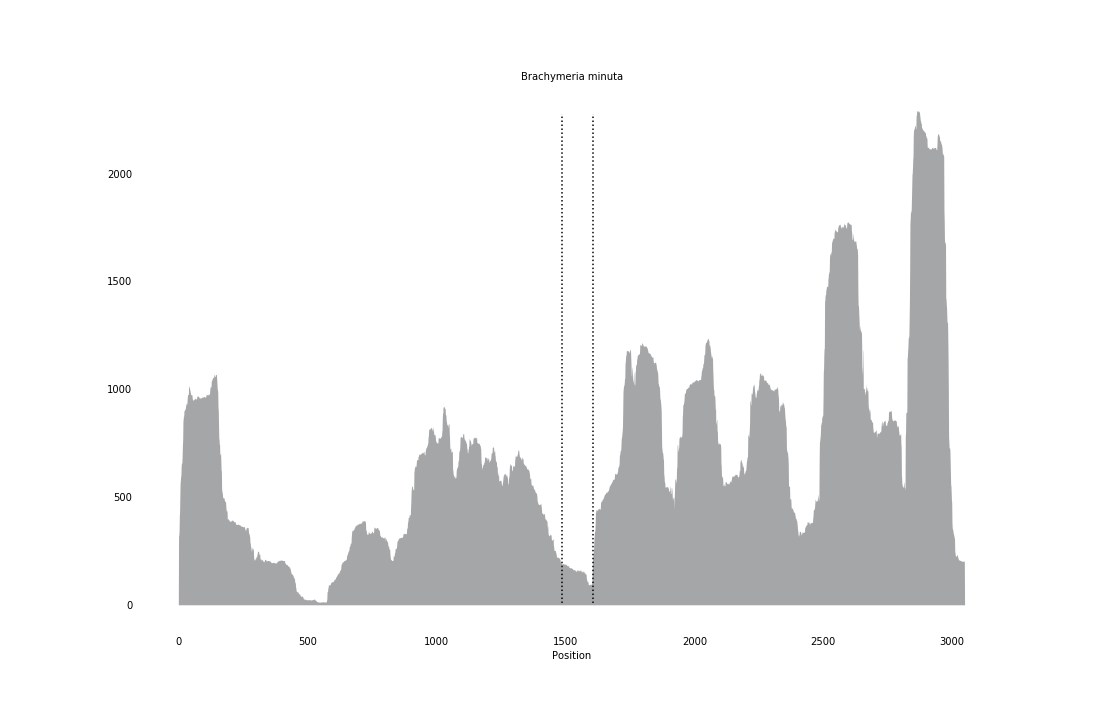

Supplement: Supplementary file 2 — Supplementary information [file 41598_2019_55573_MOESM2_ESM.zip › SupplementaryFile1/Metazoa/Protostomia/Arthropoda/Insecta/Brachymeria_minuta_coverage_correct.png]

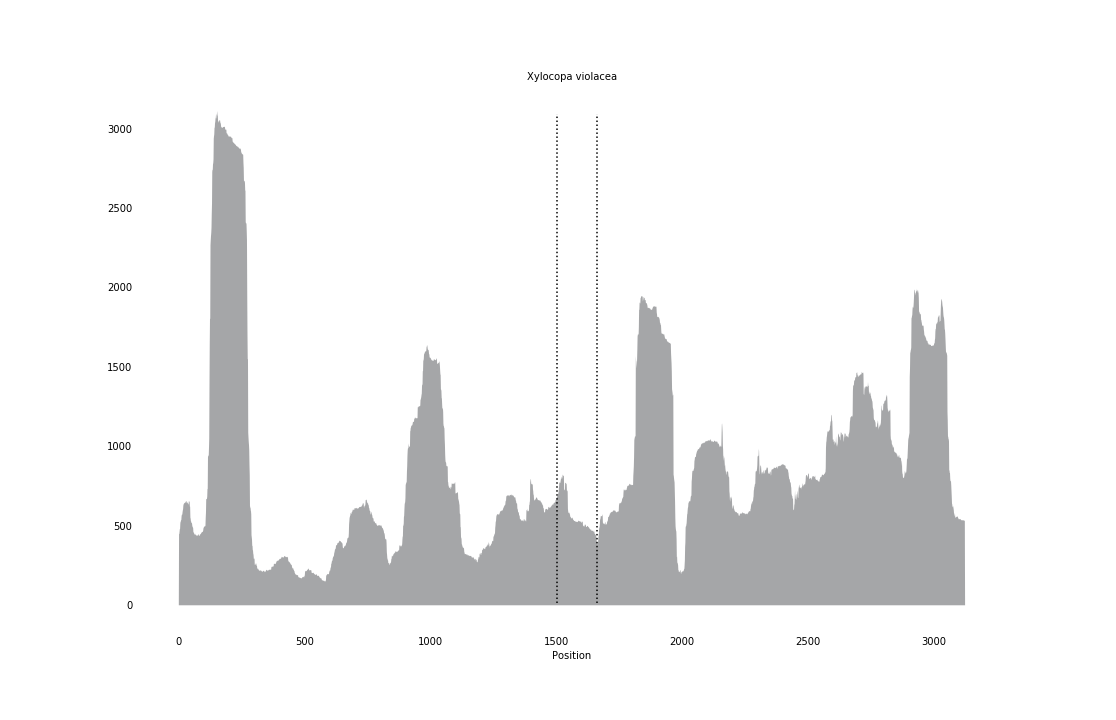

Supplement: Supplementary file 2 — Supplementary information [file 41598_2019_55573_MOESM2_ESM.zip › SupplementaryFile1/Metazoa/Protostomia/Arthropoda/Insecta/Xylocopa_violacea_coverage_correct.png]

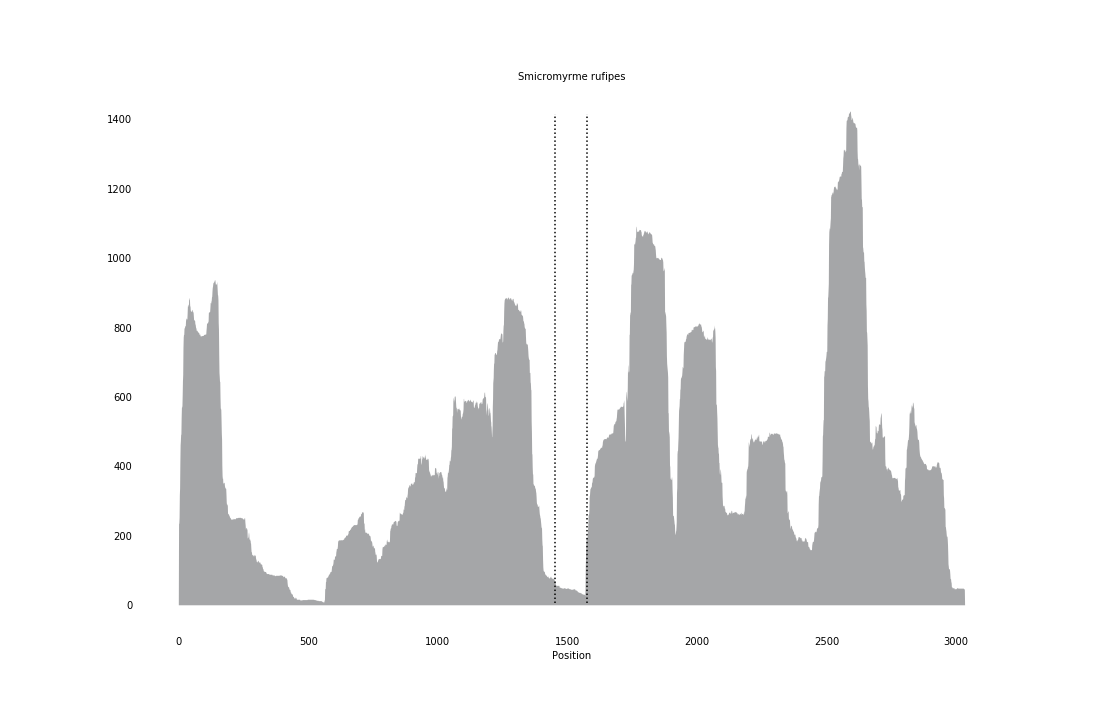

Supplement: Supplementary file 2 — Supplementary information [file 41598_2019_55573_MOESM2_ESM.zip › SupplementaryFile1/Metazoa/Protostomia/Arthropoda/Insecta/Smicromyrme_rufipes_coverage_correct.png]

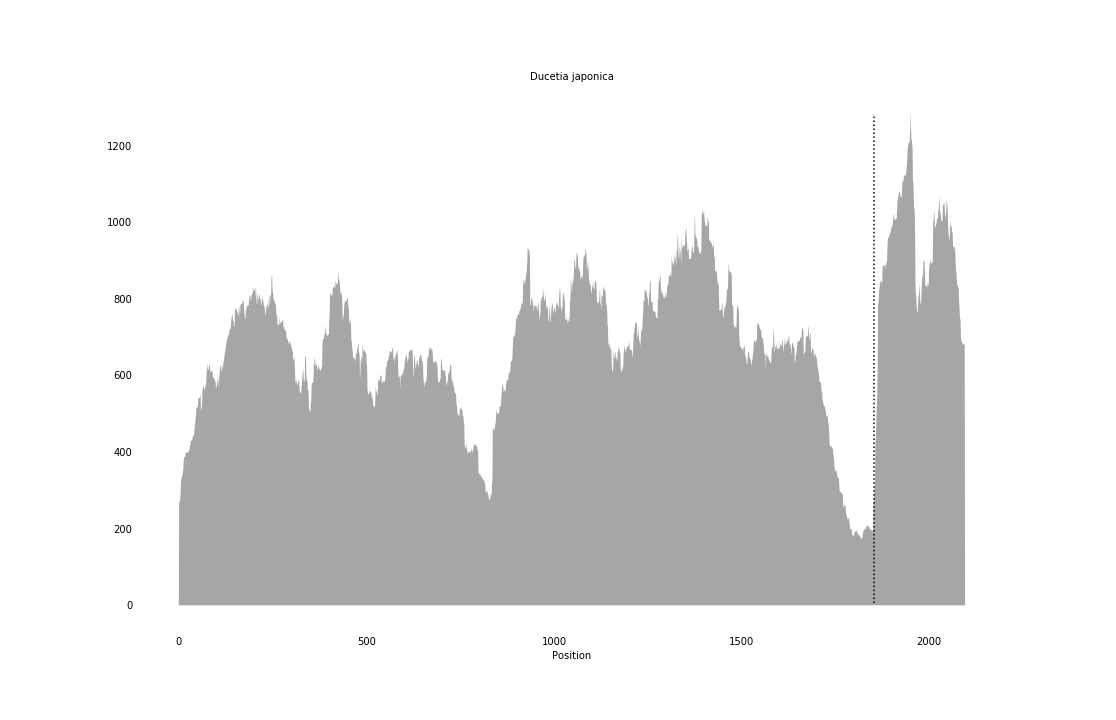

Supplement: Supplementary file 2 — Supplementary information [file 41598_2019_55573_MOESM2_ESM.zip › SupplementaryFile1/Metazoa/Protostomia/Arthropoda/Insecta/Ducetia_japonica_coverage.png]

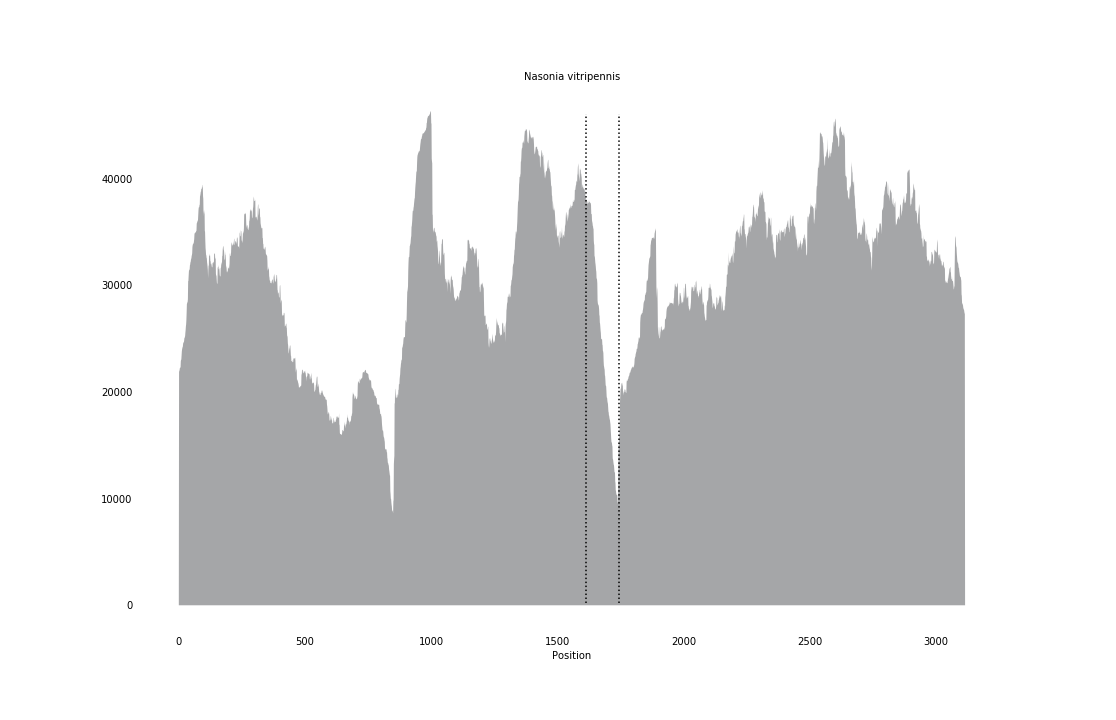

Supplement: Supplementary file 2 — Supplementary information [file 41598_2019_55573_MOESM2_ESM.zip › SupplementaryFile1/Metazoa/Protostomia/Arthropoda/Insecta/Nasonia_vitripennis_coverage_correct.png]

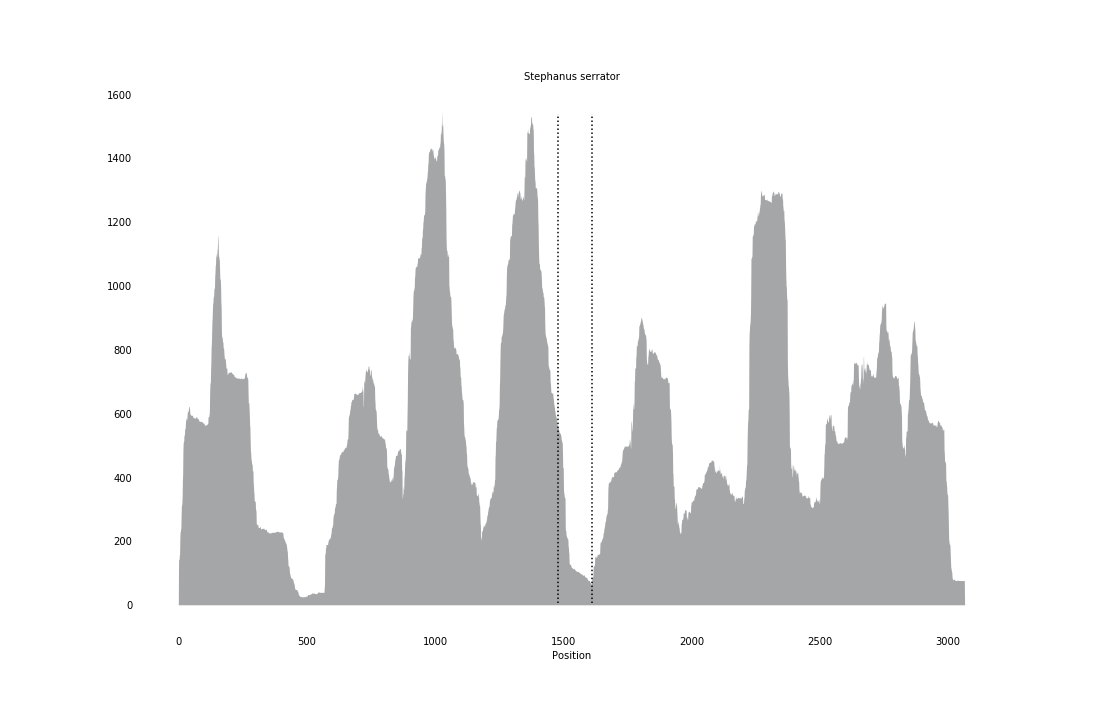

Supplement: Supplementary file 2 — Supplementary information [file 41598_2019_55573_MOESM2_ESM.zip › SupplementaryFile1/Metazoa/Protostomia/Arthropoda/Insecta/Stephanus_serrator_coverage_correct.png]

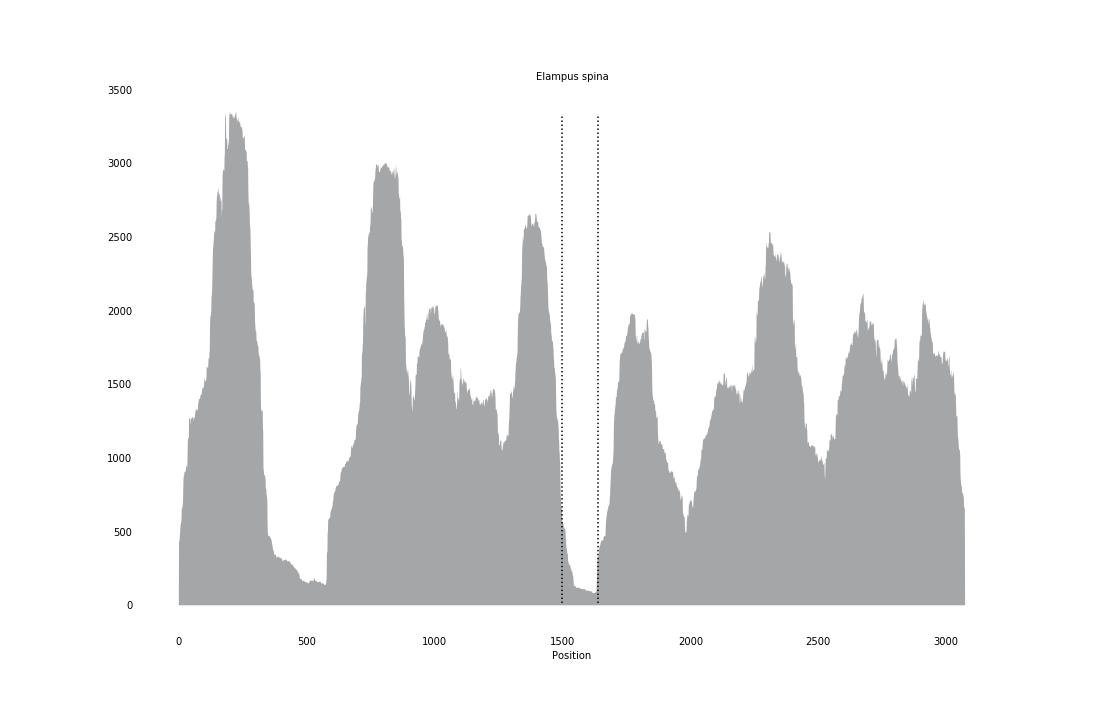

Supplement: Supplementary file 2 — Supplementary information [file 41598_2019_55573_MOESM2_ESM.zip › SupplementaryFile1/Metazoa/Protostomia/Arthropoda/Insecta/Elampus_spina_coverage_correct.png]

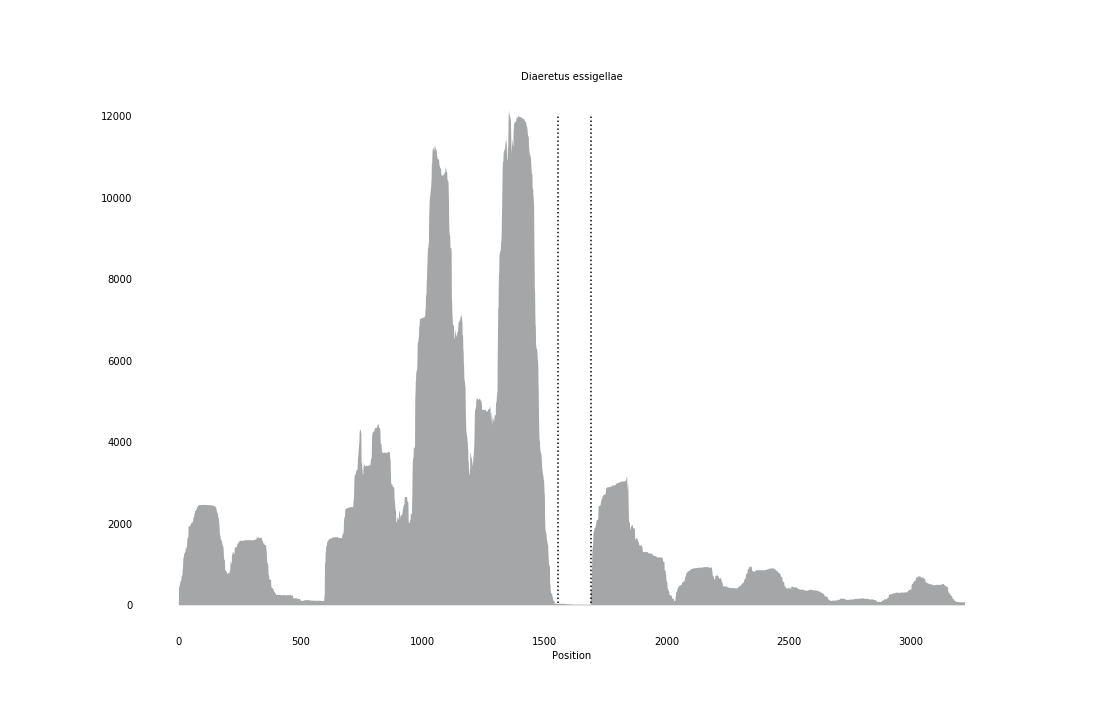

Supplement: Supplementary file 2 — Supplementary information [file 41598_2019_55573_MOESM2_ESM.zip › SupplementaryFile1/Metazoa/Protostomia/Arthropoda/Insecta/Diaeretus_essigellae_coverage_correct.png]

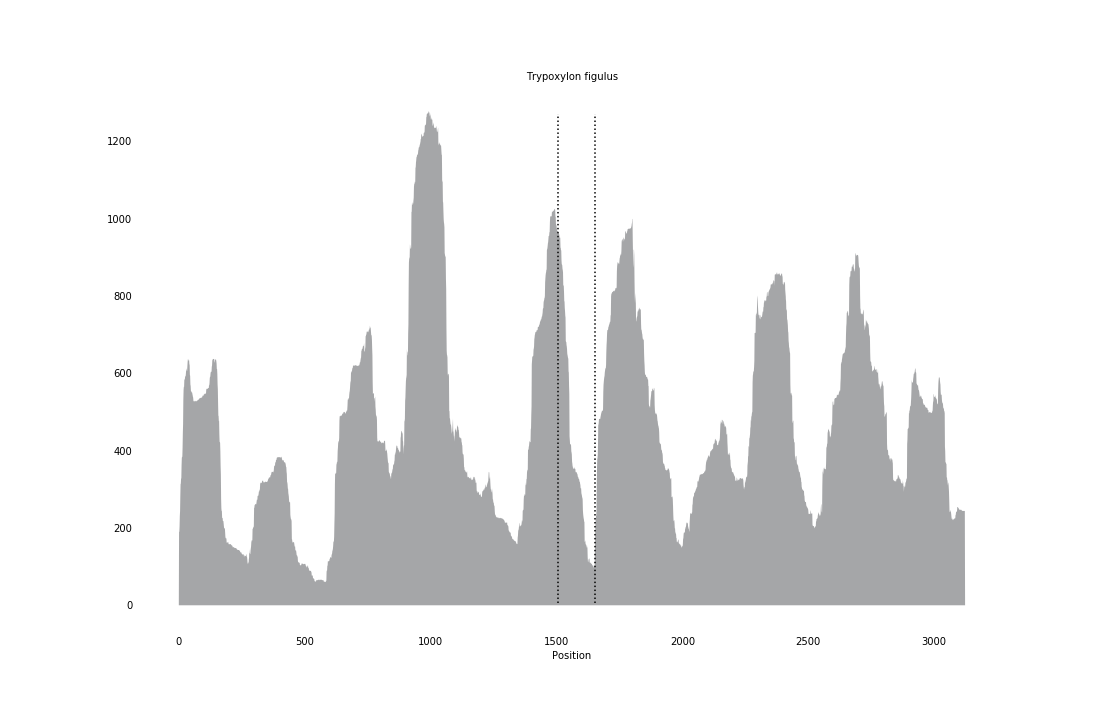

Supplement: Supplementary file 2 — Supplementary information [file 41598_2019_55573_MOESM2_ESM.zip › SupplementaryFile1/Metazoa/Protostomia/Arthropoda/Insecta/Trypoxylon_figulus_coverage_correct.png]

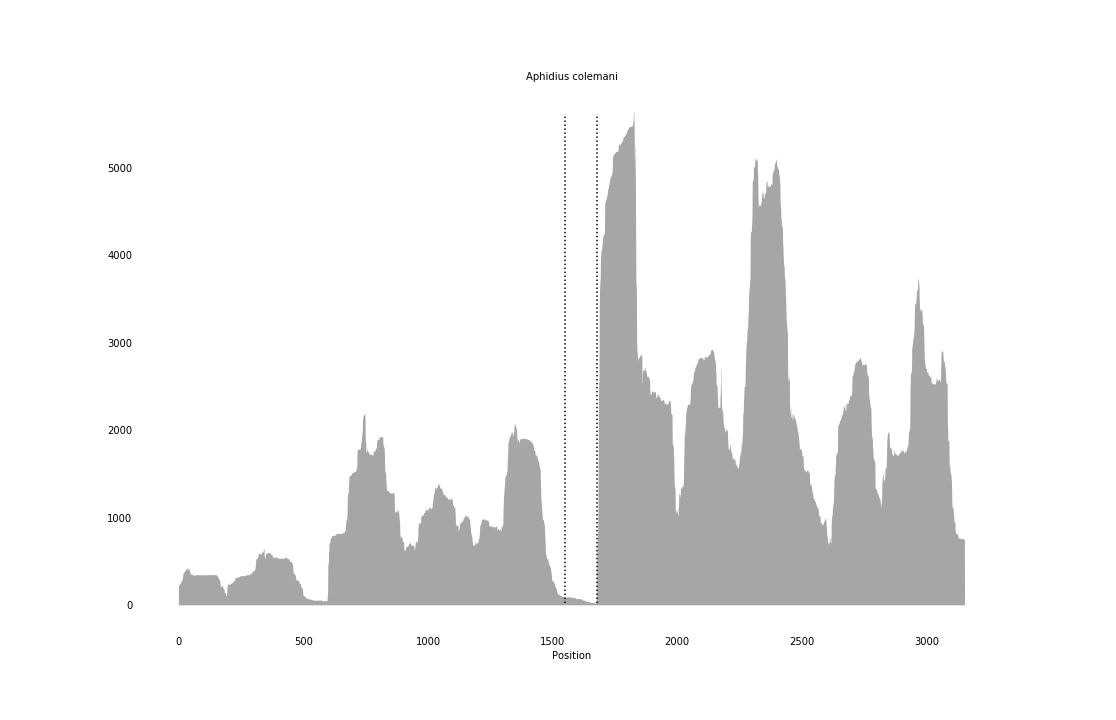

Supplement: Supplementary file 2 — Supplementary information [file 41598_2019_55573_MOESM2_ESM.zip › SupplementaryFile1/Metazoa/Protostomia/Arthropoda/Insecta/Aphidius_colemani_coverage_correct.png]

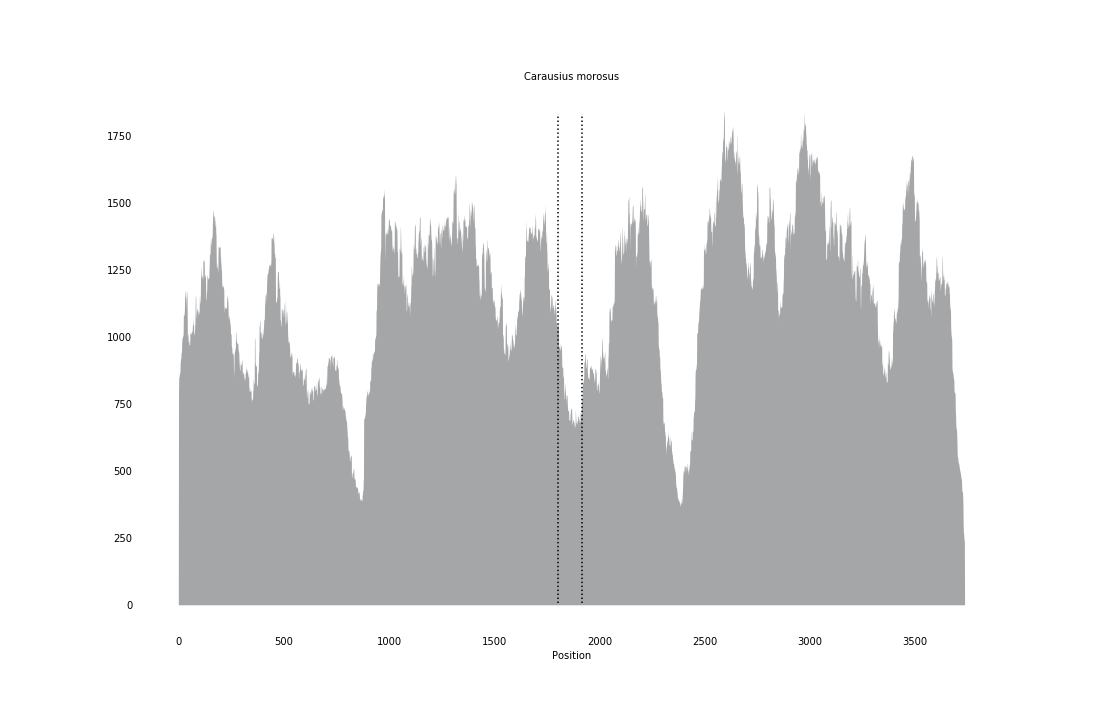

Supplement: Supplementary file 2 — Supplementary information [file 41598_2019_55573_MOESM2_ESM.zip › SupplementaryFile1/Metazoa/Protostomia/Arthropoda/Insecta/Carausius_morosus_coverage_correct.png]

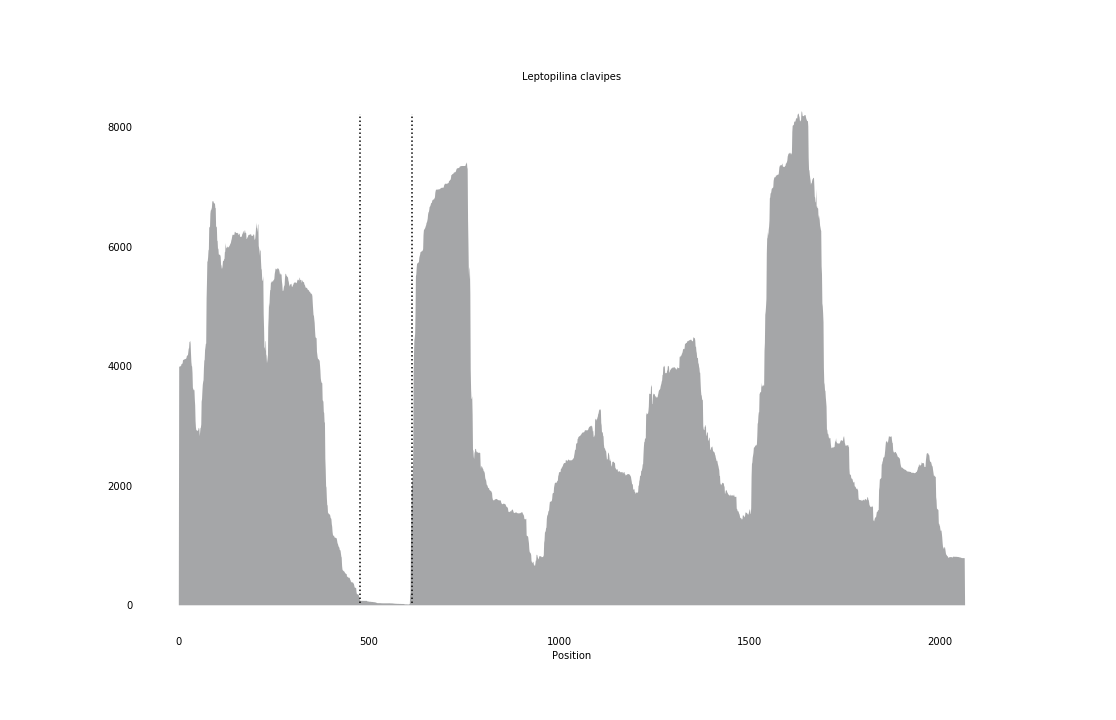

Supplement: Supplementary file 2 — Supplementary information [file 41598_2019_55573_MOESM2_ESM.zip › SupplementaryFile1/Metazoa/Protostomia/Arthropoda/Insecta/Leptopilina_clavipes_coverage_correct.png]

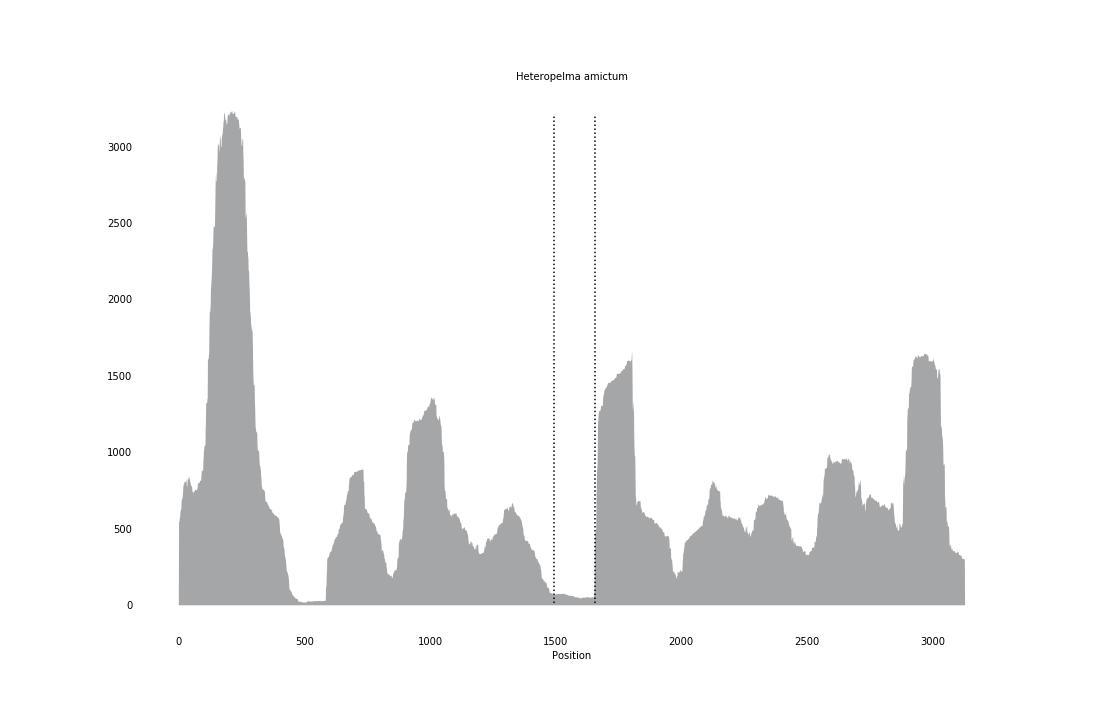

Supplement: Supplementary file 2 — Supplementary information [file 41598_2019_55573_MOESM2_ESM.zip › SupplementaryFile1/Metazoa/Protostomia/Arthropoda/Insecta/Heteropelma_amictum_coverage_correct.png]

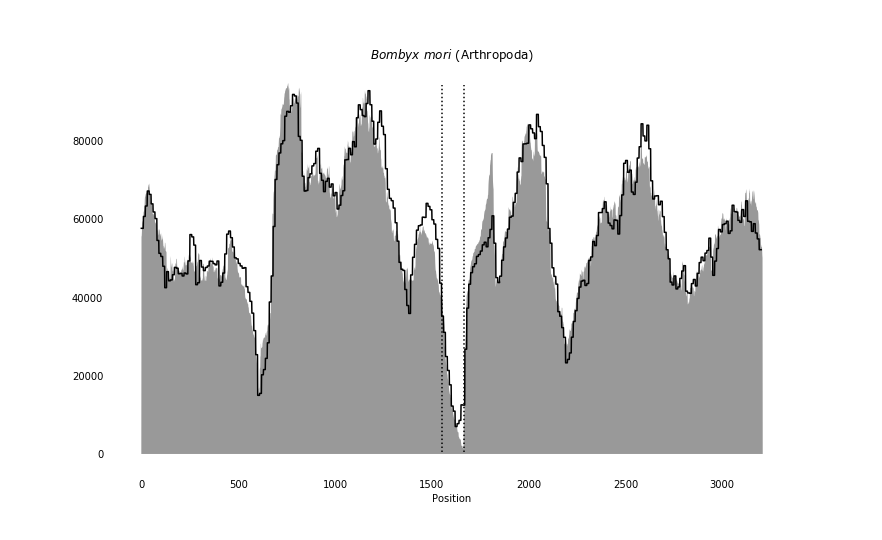

Supplement: Supplementary file 2 — Supplementary information [file 41598_2019_55573_MOESM2_ESM.zip › SupplementaryFile1/Metazoa/Protostomia/Arthropoda/Insecta/Bombyx_mori_overage.png]

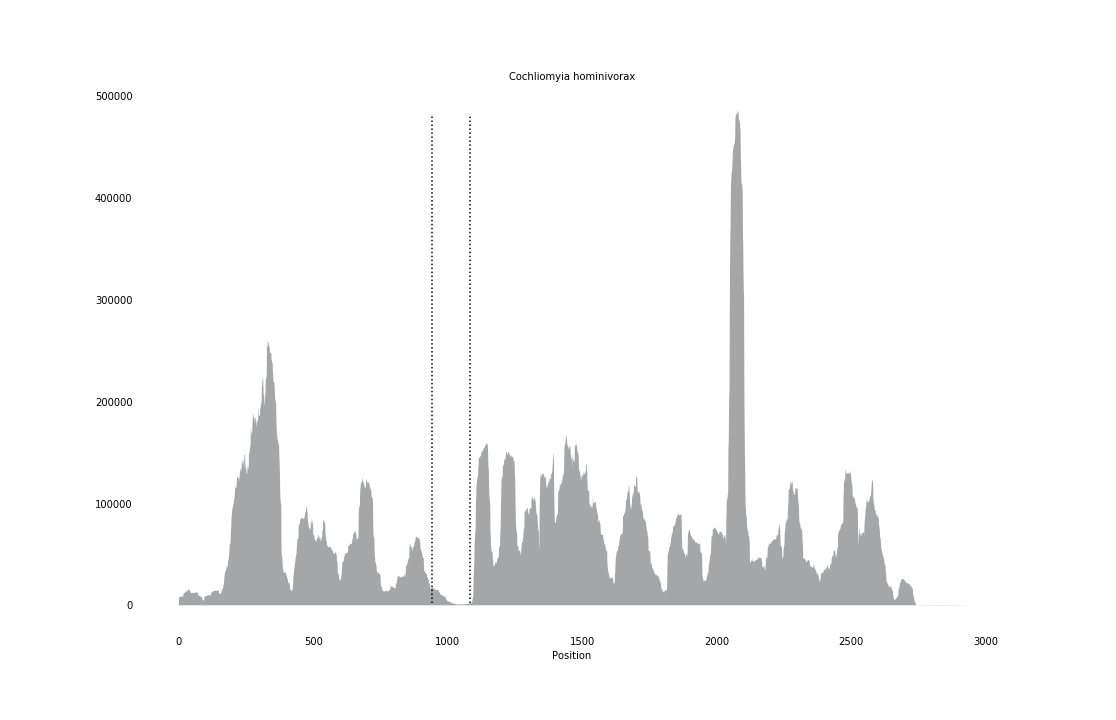

Supplement: Supplementary file 2 — Supplementary information [file 41598_2019_55573_MOESM2_ESM.zip › SupplementaryFile1/Metazoa/Protostomia/Arthropoda/Insecta/Cochliomyia_hominivorax_coverage_correct.png]

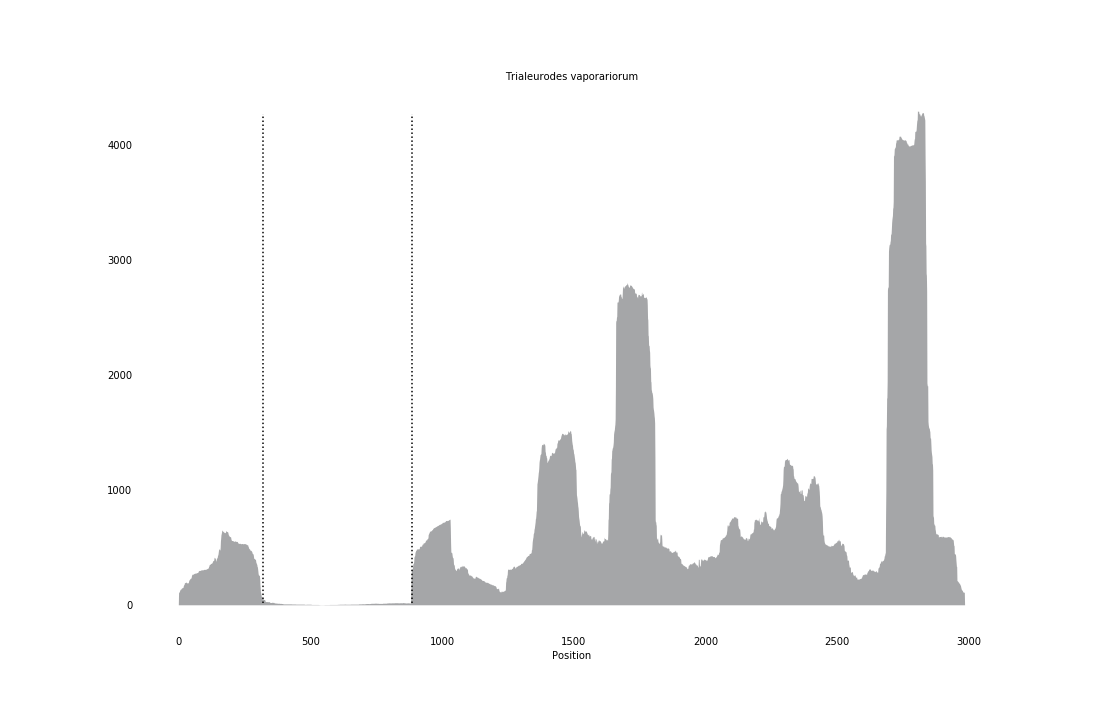

Supplement: Supplementary file 2 — Supplementary information [file 41598_2019_55573_MOESM2_ESM.zip › SupplementaryFile1/Metazoa/Protostomia/Arthropoda/Insecta/Trialeurodes_vaporariorum_coverage_correct.png]

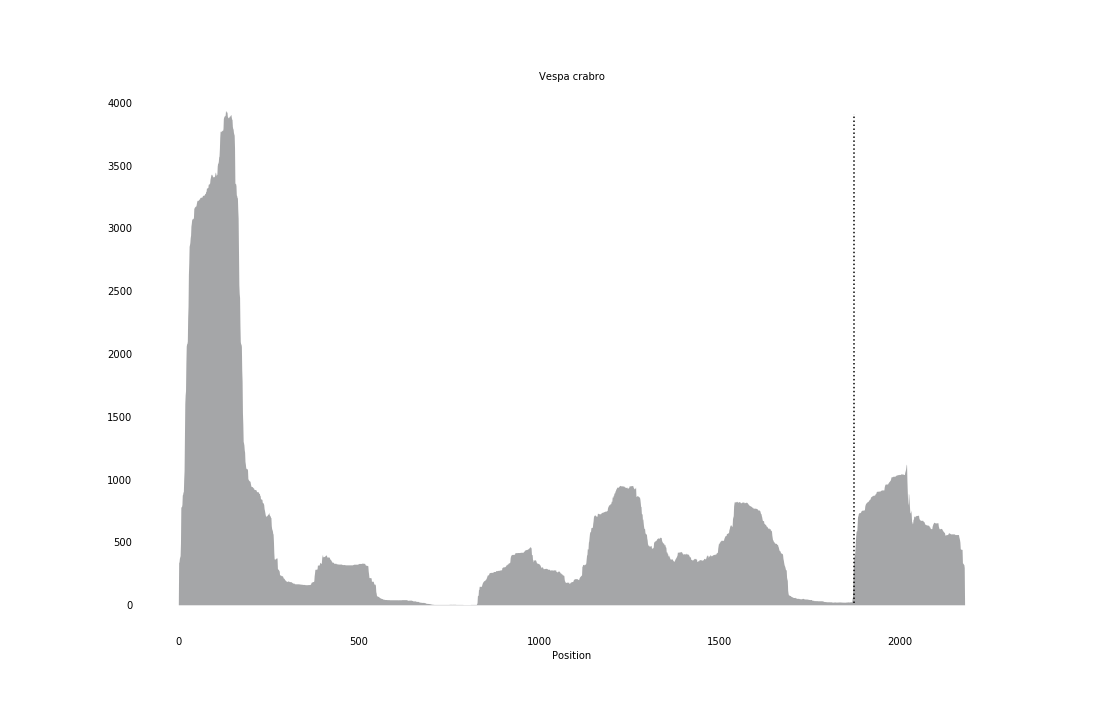

Supplement: Supplementary file 2 — Supplementary information [file 41598_2019_55573_MOESM2_ESM.zip › SupplementaryFile1/Metazoa/Protostomia/Arthropoda/Insecta/Vespa_crabro_coverage.png]

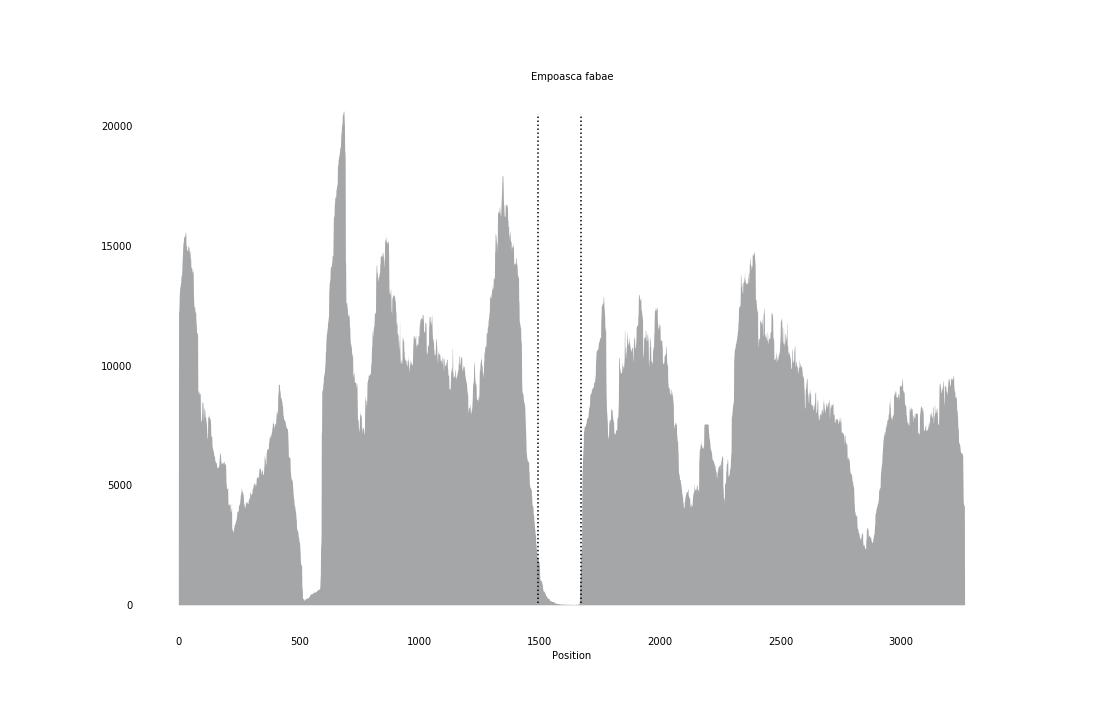

Supplement: Supplementary file 2 — Supplementary information [file 41598_2019_55573_MOESM2_ESM.zip › SupplementaryFile1/Metazoa/Protostomia/Arthropoda/Insecta/Empoasca_fabae_coverage_correct.png]

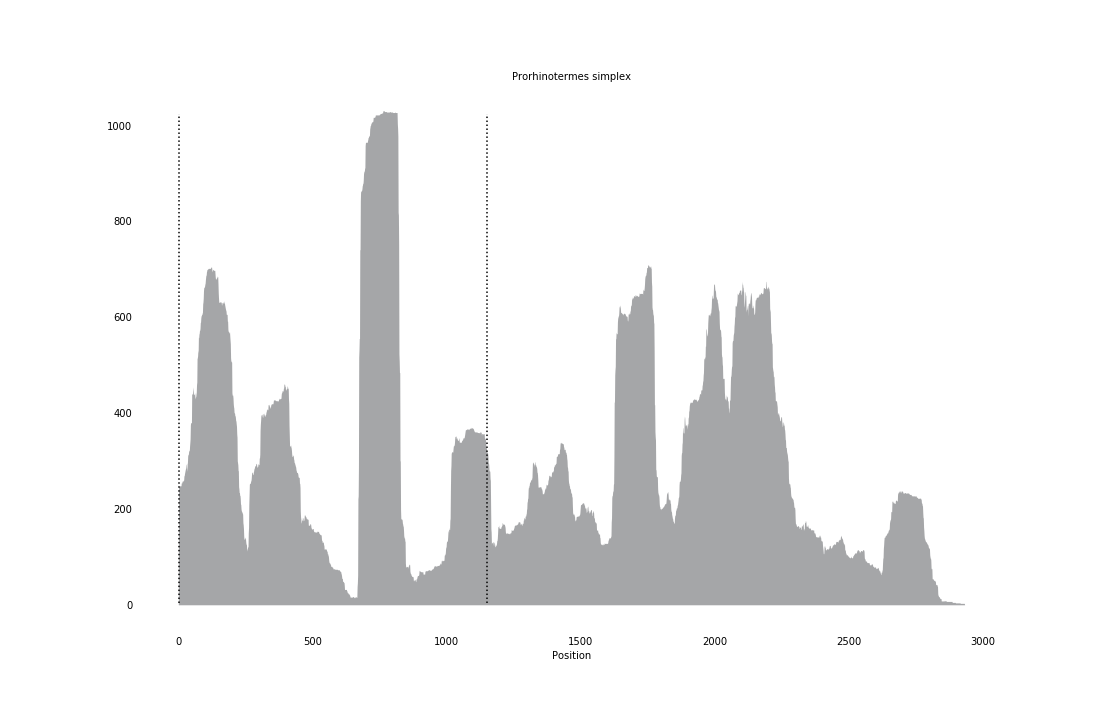

Supplement: Supplementary file 2 — Supplementary information [file 41598_2019_55573_MOESM2_ESM.zip › SupplementaryFile1/Metazoa/Protostomia/Arthropoda/Insecta/Prorhinotermes_simplex_coverage_correct.png]

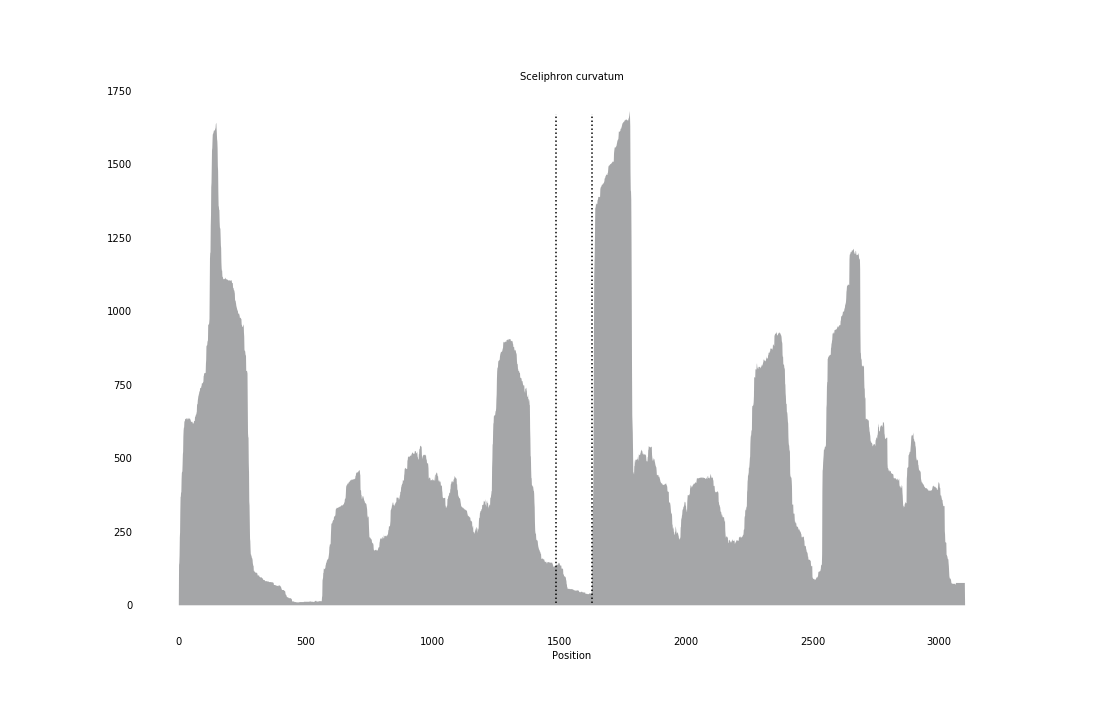

Supplement: Supplementary file 2 — Supplementary information [file 41598_2019_55573_MOESM2_ESM.zip › SupplementaryFile1/Metazoa/Protostomia/Arthropoda/Insecta/Sceliphron_curvatum_coverage_correct.png]

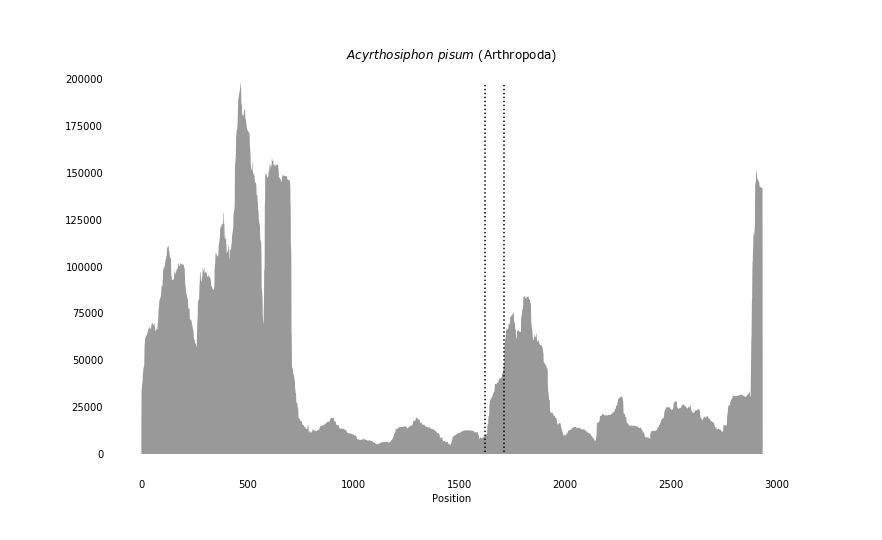

Supplement: Supplementary file 2 — Supplementary information [file 41598_2019_55573_MOESM2_ESM.zip › SupplementaryFile1/Metazoa/Protostomia/Arthropoda/Insecta/Acyrthosiphon_pisum_coverage.png]

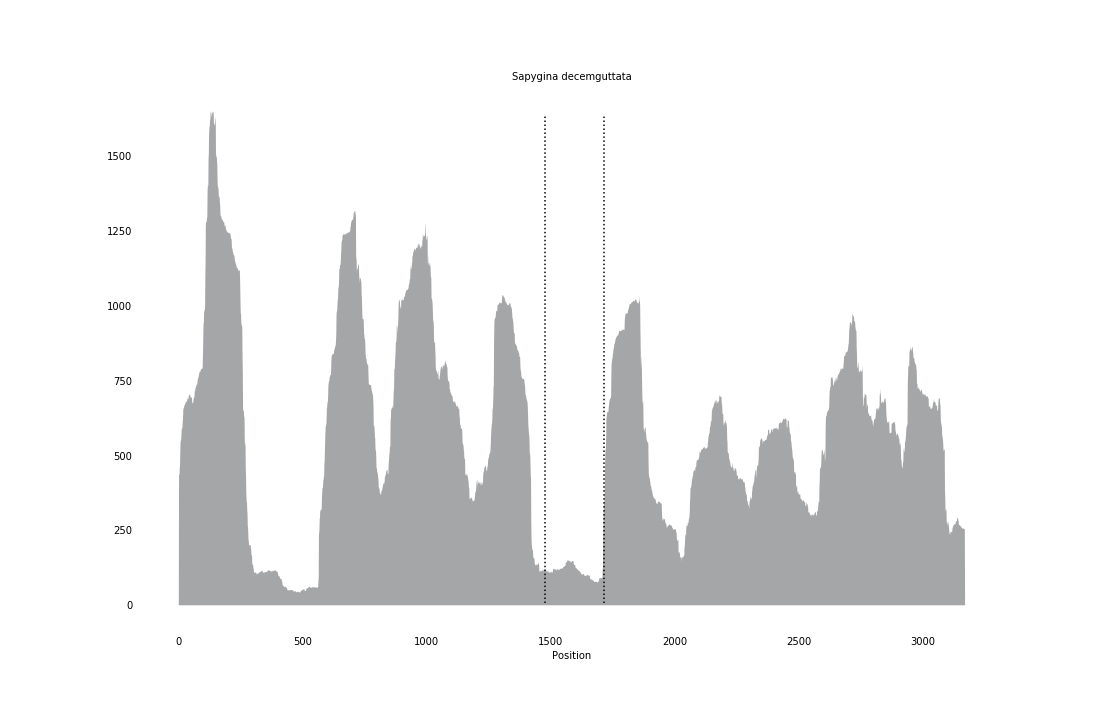

Supplement: Supplementary file 2 — Supplementary information [file 41598_2019_55573_MOESM2_ESM.zip › SupplementaryFile1/Metazoa/Protostomia/Arthropoda/Insecta/Sapygina_decemguttata_coverage_correct.png]

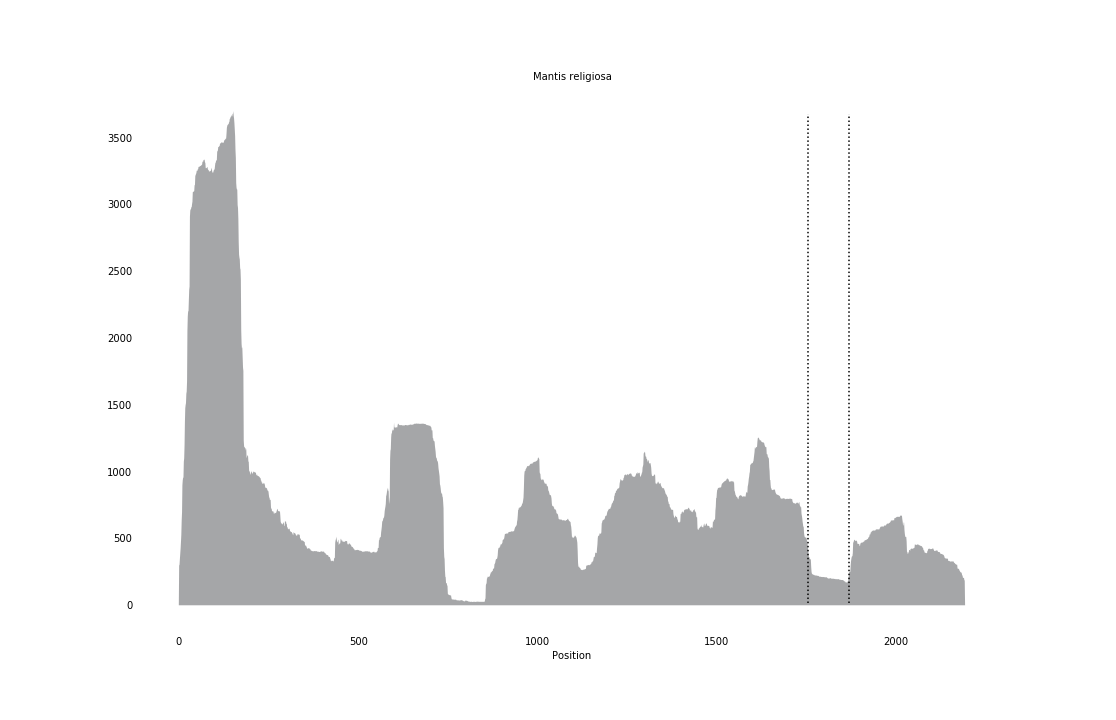

Supplement: Supplementary file 2 — Supplementary information [file 41598_2019_55573_MOESM2_ESM.zip › SupplementaryFile1/Metazoa/Protostomia/Arthropoda/Insecta/Mantis_religiosa_coverage_correct.png]

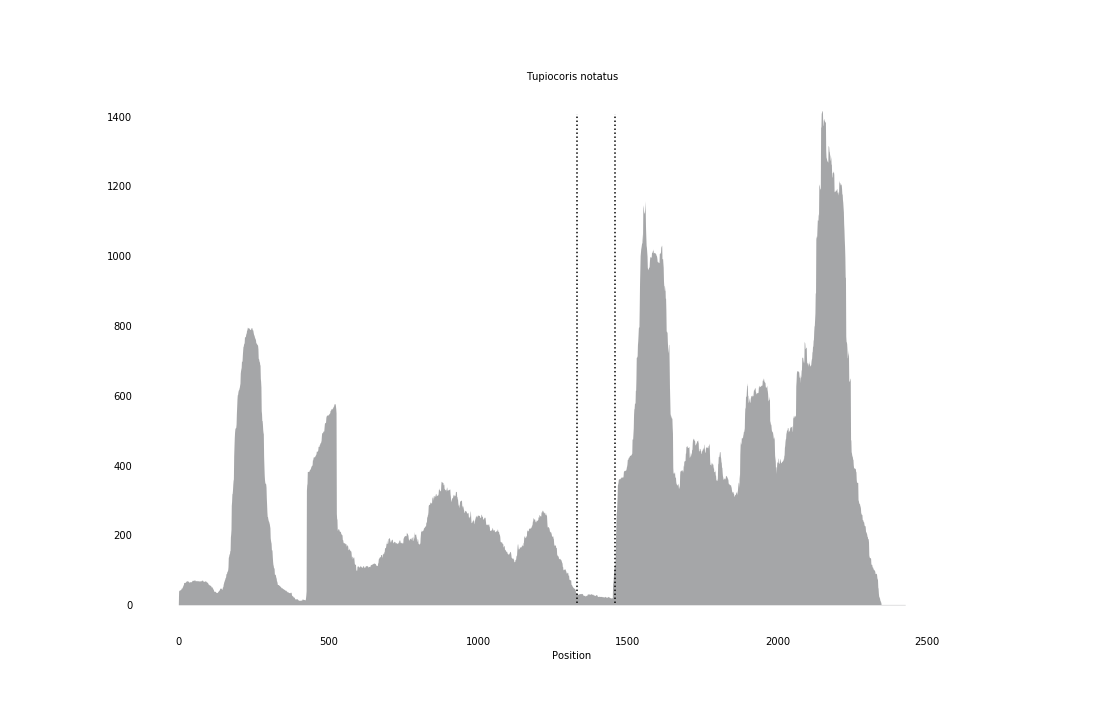

Supplement: Supplementary file 2 — Supplementary information [file 41598_2019_55573_MOESM2_ESM.zip › SupplementaryFile1/Metazoa/Protostomia/Arthropoda/Insecta/Tupiocoris_notatus_coverage_correct.png]

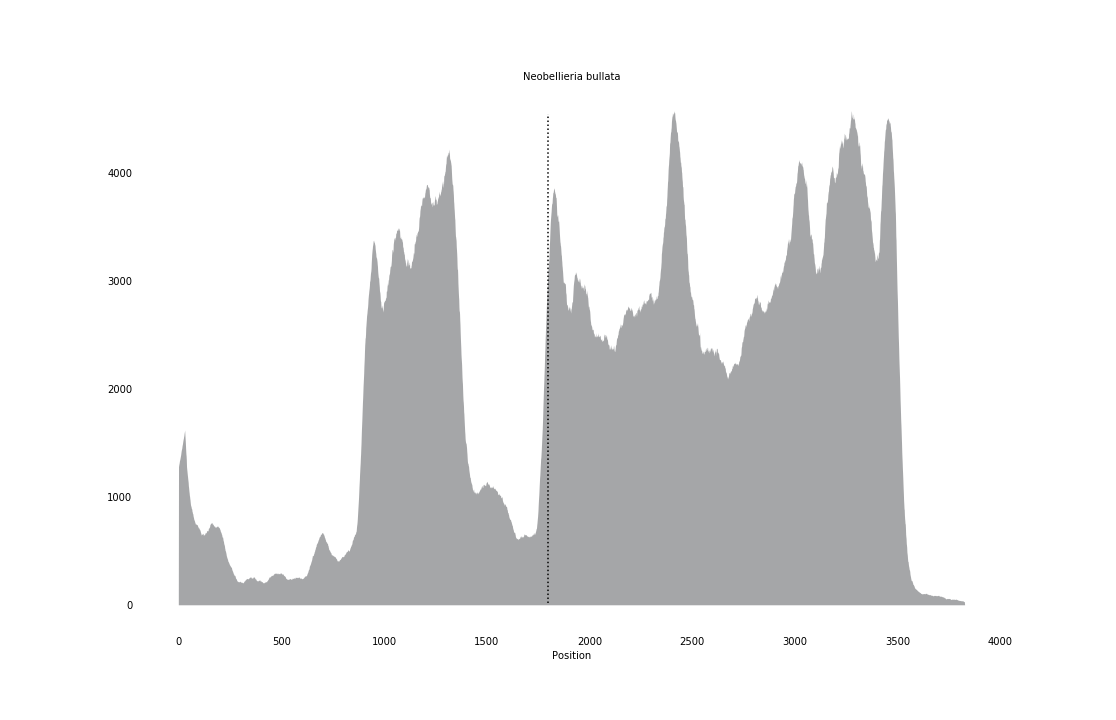

Supplement: Supplementary file 2 — Supplementary information [file 41598_2019_55573_MOESM2_ESM.zip › SupplementaryFile1/Metazoa/Protostomia/Arthropoda/Insecta/Neobellieria_bullata_coverage.png]

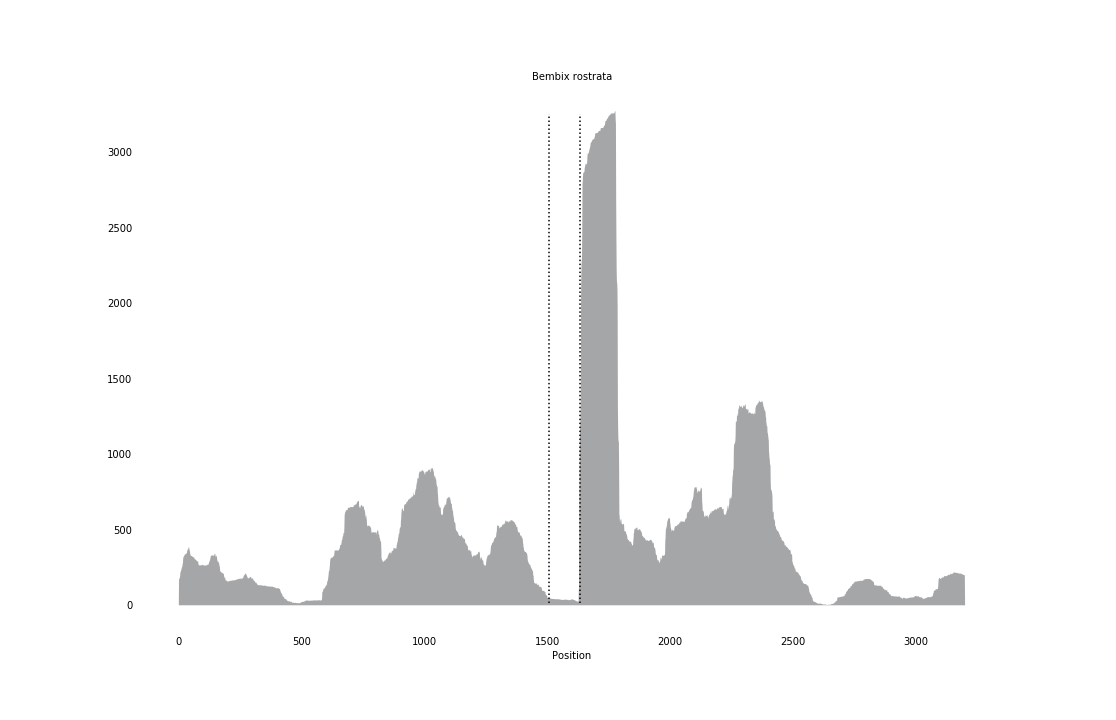

Supplement: Supplementary file 2 — Supplementary information [file 41598_2019_55573_MOESM2_ESM.zip › SupplementaryFile1/Metazoa/Protostomia/Arthropoda/Insecta/Bembix_rostrata_coverage_correct.png]

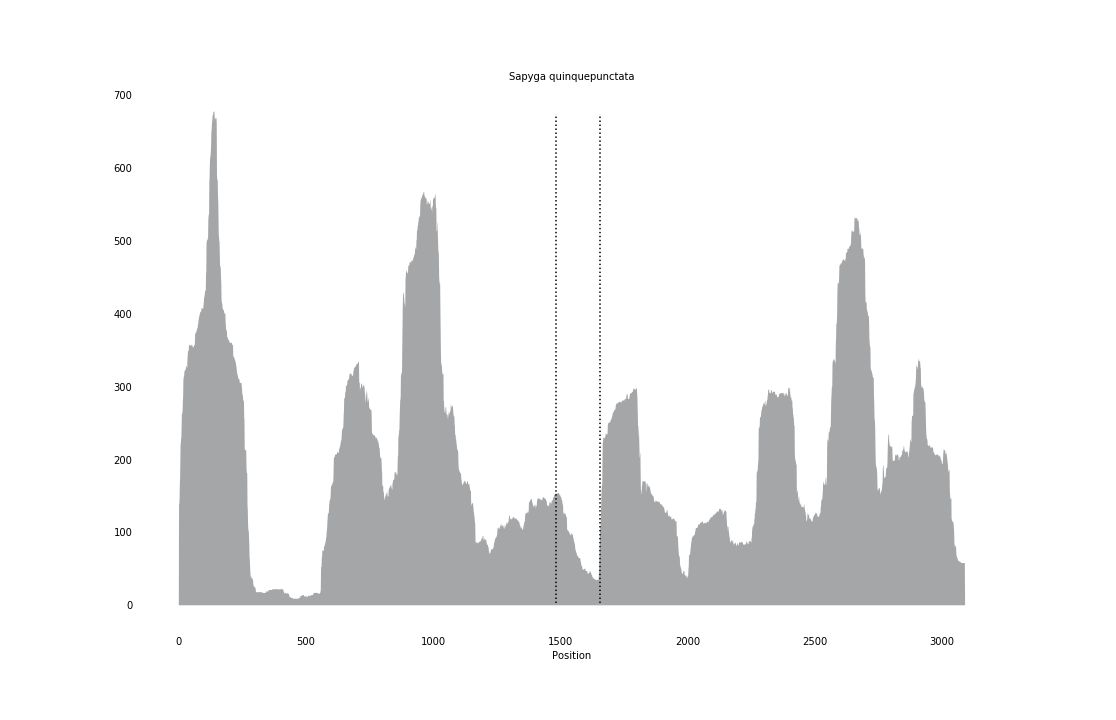

Supplement: Supplementary file 2 — Supplementary information [file 41598_2019_55573_MOESM2_ESM.zip › SupplementaryFile1/Metazoa/Protostomia/Arthropoda/Insecta/Sapyga_quinquepunctata_coverage_correct.png]

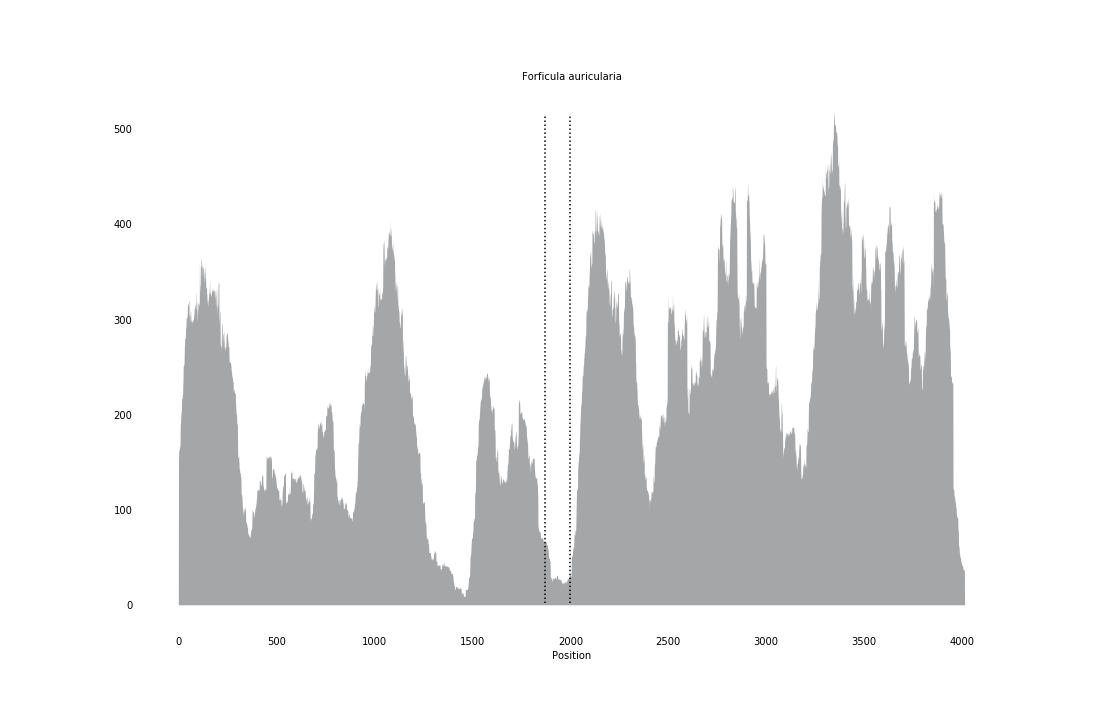

Supplement: Supplementary file 2 — Supplementary information [file 41598_2019_55573_MOESM2_ESM.zip › SupplementaryFile1/Metazoa/Protostomia/Arthropoda/Insecta/Forficula_auricularia_coverage_correct.png]

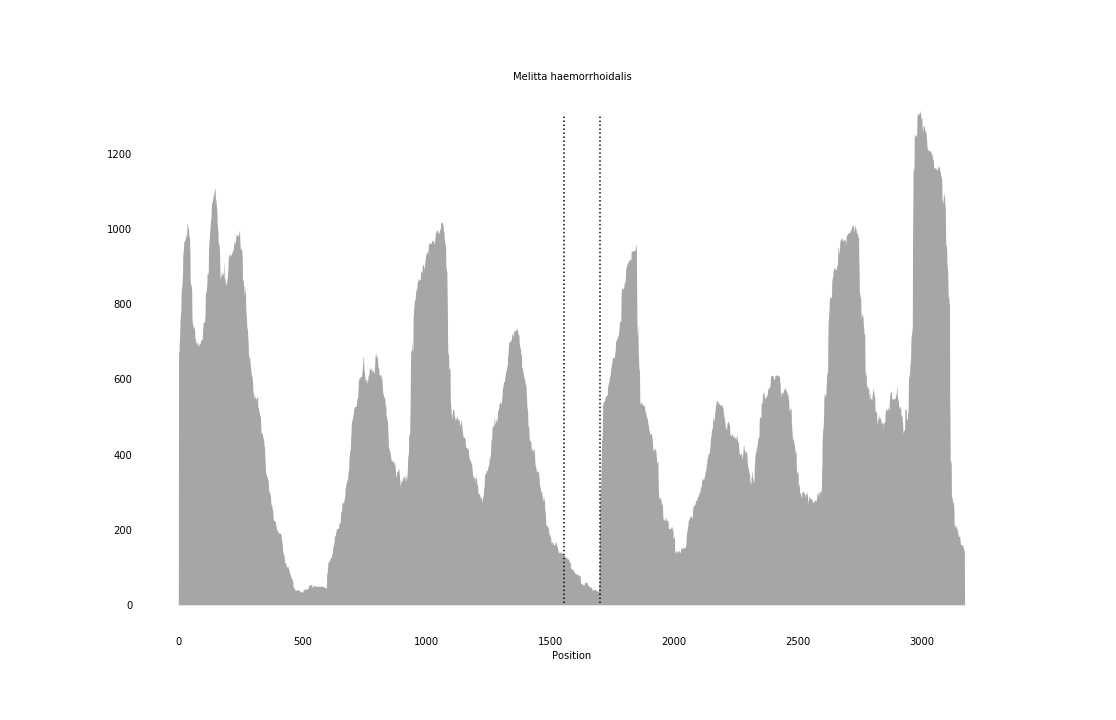

Supplement: Supplementary file 2 — Supplementary information [file 41598_2019_55573_MOESM2_ESM.zip › SupplementaryFile1/Metazoa/Protostomia/Arthropoda/Insecta/Melitta_haemorrhoidalis_coverage_correct.png]

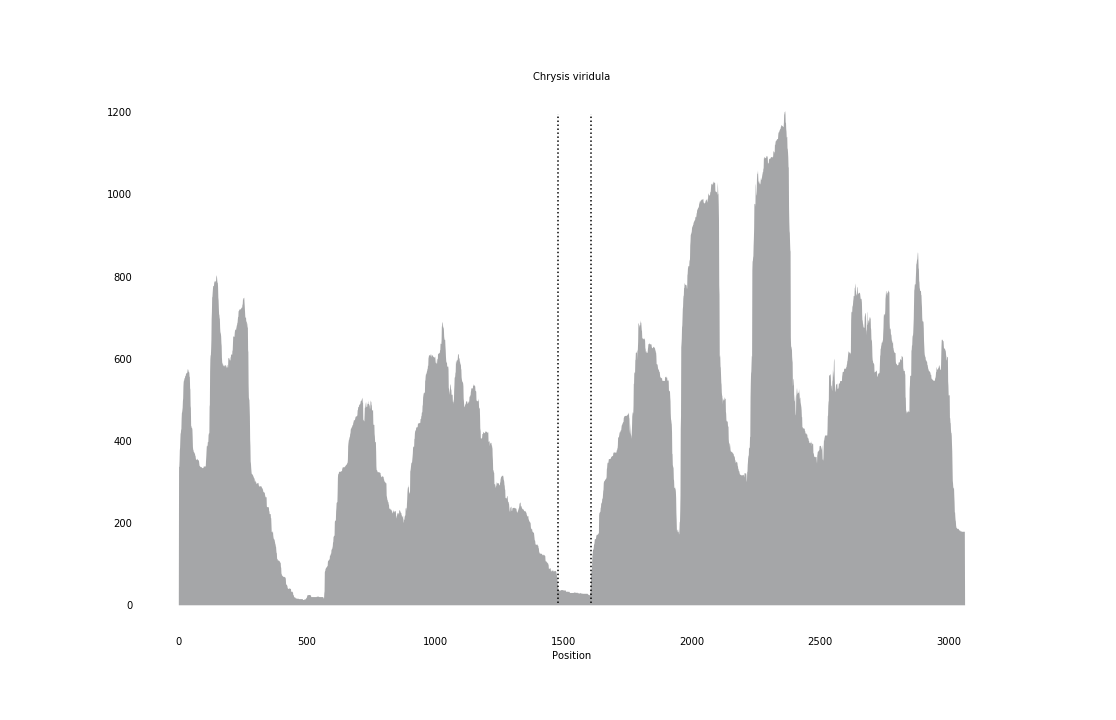

Supplement: Supplementary file 2 — Supplementary information [file 41598_2019_55573_MOESM2_ESM.zip › SupplementaryFile1/Metazoa/Protostomia/Arthropoda/Insecta/Chrysis_viridula_coverage_correct.png]

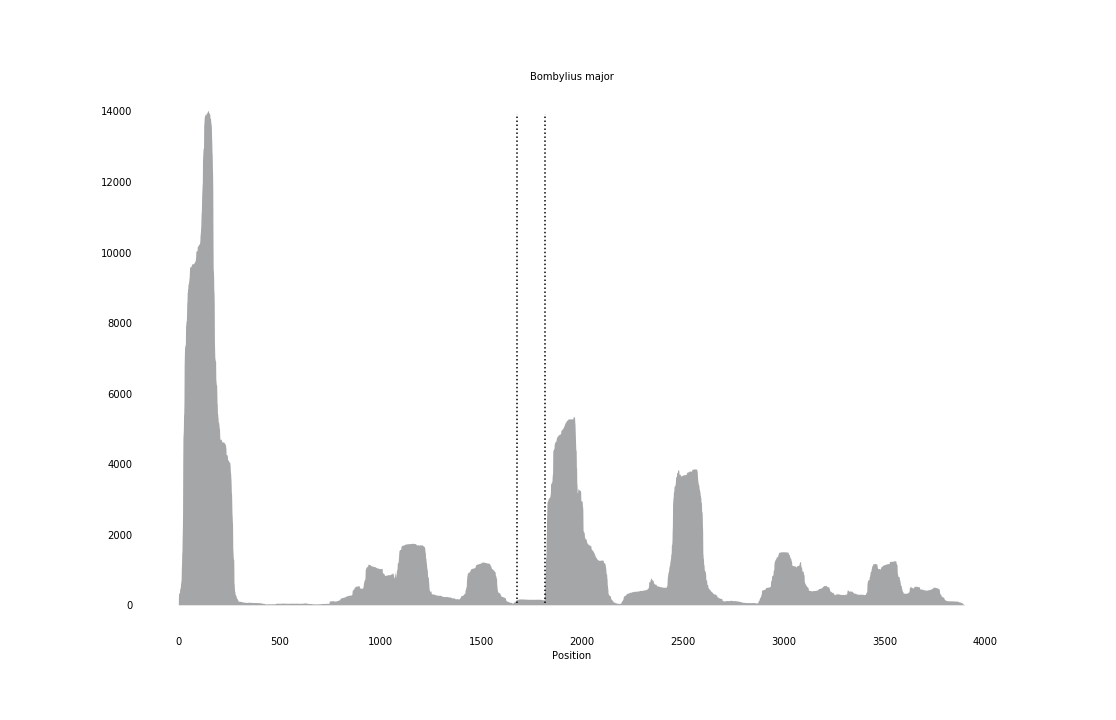

Supplement: Supplementary file 2 — Supplementary information [file 41598_2019_55573_MOESM2_ESM.zip › SupplementaryFile1/Metazoa/Protostomia/Arthropoda/Insecta/Bombylius_major_coverage_correct.png]

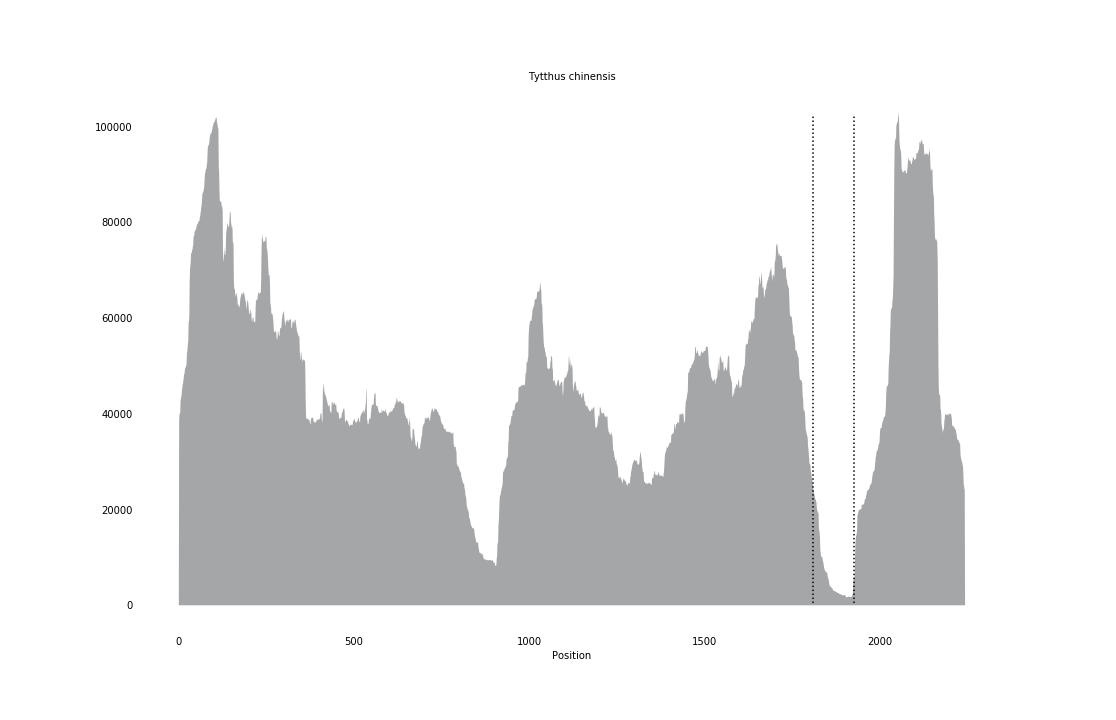

Supplement: Supplementary file 2 — Supplementary information [file 41598_2019_55573_MOESM2_ESM.zip › SupplementaryFile1/Metazoa/Protostomia/Arthropoda/Insecta/Tytthus_chinensis_coverage_correct.png]

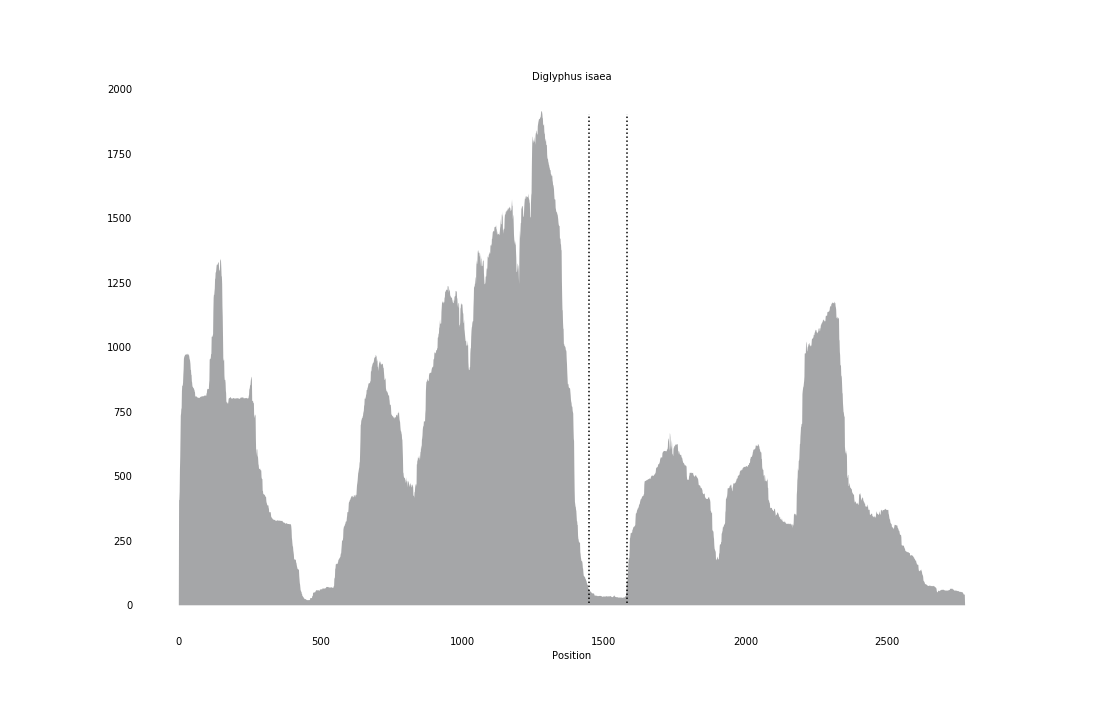

Supplement: Supplementary file 2 — Supplementary information [file 41598_2019_55573_MOESM2_ESM.zip › SupplementaryFile1/Metazoa/Protostomia/Arthropoda/Insecta/Diglyphus_isaea_coverage_correct.png]

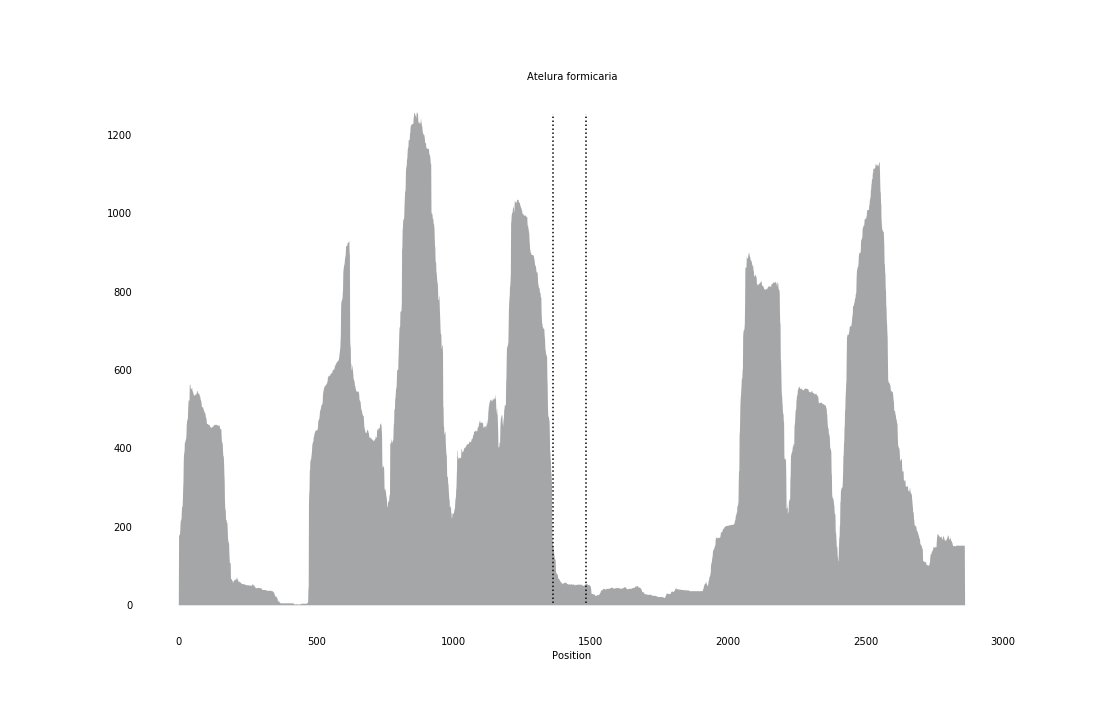

Supplement: Supplementary file 2 — Supplementary information [file 41598_2019_55573_MOESM2_ESM.zip › SupplementaryFile1/Metazoa/Protostomia/Arthropoda/Insecta/Atelura_formicaria_coverage_correct.png]

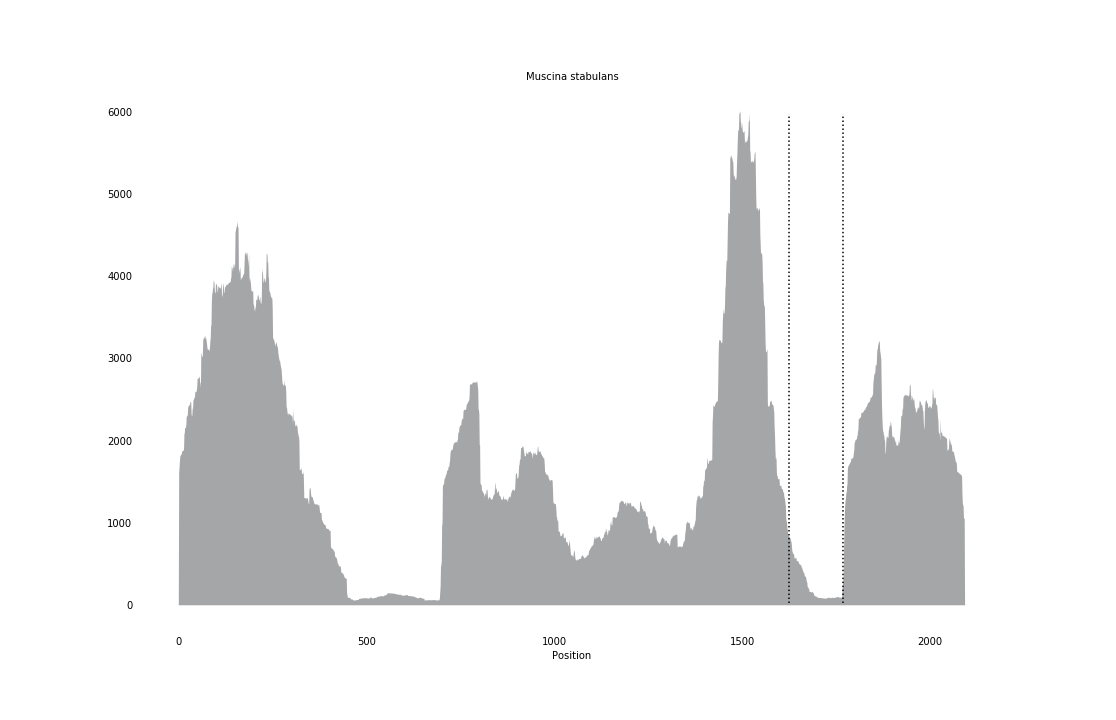

Supplement: Supplementary file 2 — Supplementary information [file 41598_2019_55573_MOESM2_ESM.zip › SupplementaryFile1/Metazoa/Protostomia/Arthropoda/Insecta/Muscina_stabulans_coverage_correct.png]

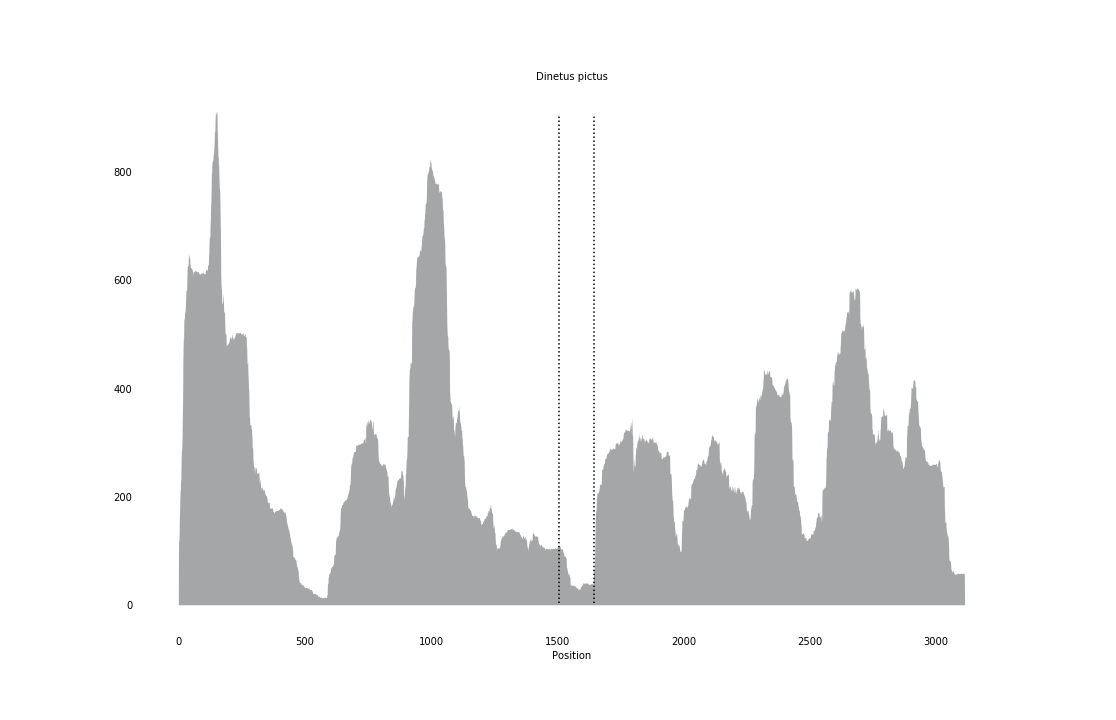

Supplement: Supplementary file 2 — Supplementary information [file 41598_2019_55573_MOESM2_ESM.zip › SupplementaryFile1/Metazoa/Protostomia/Arthropoda/Insecta/Dinetus_pictus_coverage_correct.png]

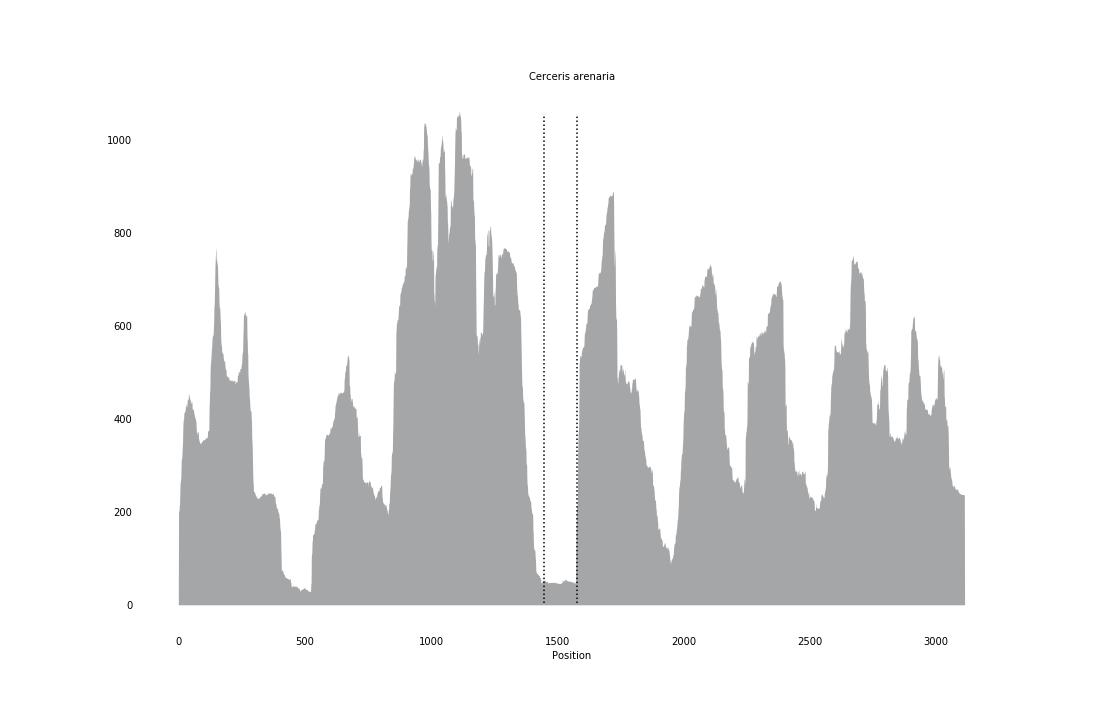

Supplement: Supplementary file 2 — Supplementary information [file 41598_2019_55573_MOESM2_ESM.zip › SupplementaryFile1/Metazoa/Protostomia/Arthropoda/Insecta/Cerceris_arenaria_coverage_correct.png]

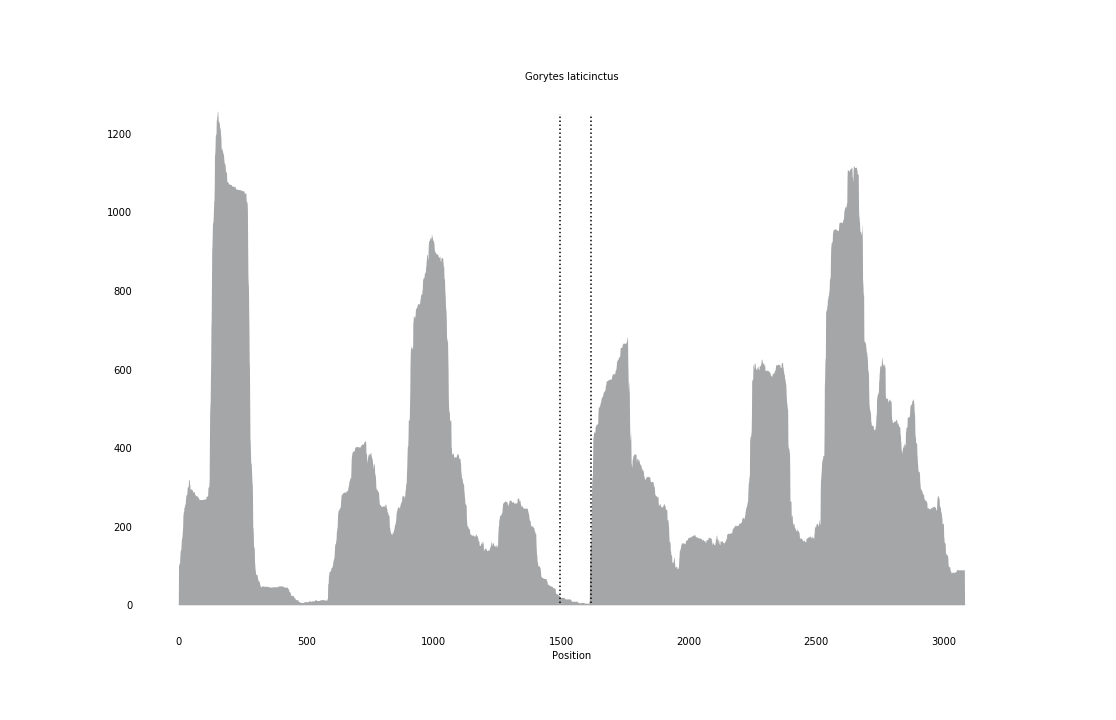

Supplement: Supplementary file 2 — Supplementary information [file 41598_2019_55573_MOESM2_ESM.zip › SupplementaryFile1/Metazoa/Protostomia/Arthropoda/Insecta/Gorytes_laticinctus_coverage_correct.png]

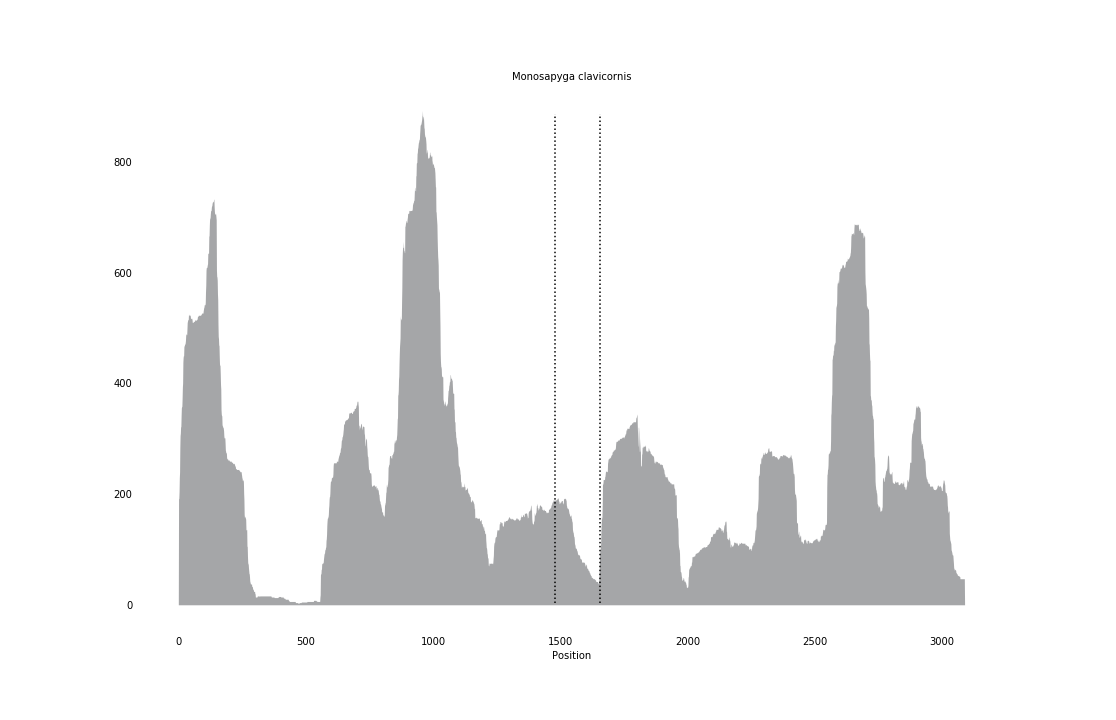

Supplement: Supplementary file 2 — Supplementary information [file 41598_2019_55573_MOESM2_ESM.zip › SupplementaryFile1/Metazoa/Protostomia/Arthropoda/Insecta/Monosapyga_clavicornis_coverage_correct.png]

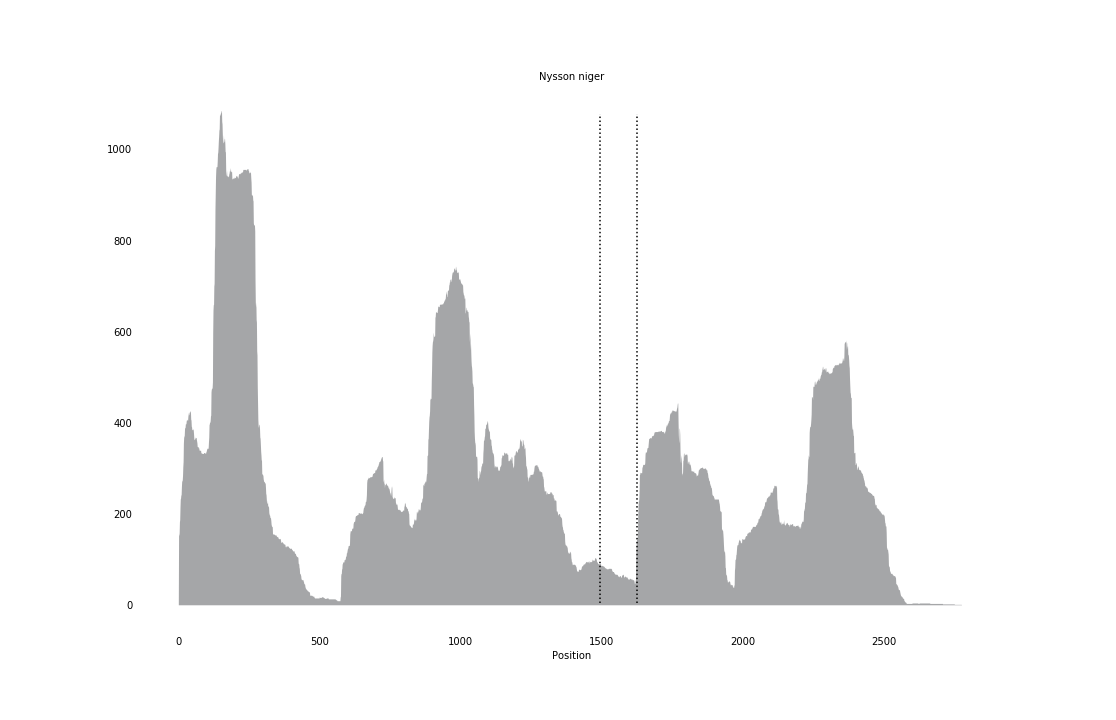

Supplement: Supplementary file 2 — Supplementary information [file 41598_2019_55573_MOESM2_ESM.zip › SupplementaryFile1/Metazoa/Protostomia/Arthropoda/Insecta/Nysson_niger_coverage_correct.png]
